# Supplementary material for: Mining and quantitative evaluation of the laboratory biosafety policy in China
Source: PLoS One. 2025 Aug 12;20(8):e0328923. doi: 10.1371/journal.pone.0328923 (PMC12342282; doi:10.1371/journal.pone.0328923)
Supplement: S1 Appendix — (DOCX) [file pone.0328923.s001.docx]

**实验室生物安全相关政策**

1. 全国人民代表大会常务委员会：中华人民共和国动物防疫法

采集、保存、运输动物病料或者病原微生物以及从事病原微生物研究、教学、检测、诊断等活动，应当遵守国家有关病原微生物实验室管理的规定。

1. 国务院：病原微生物实验室生物安全管理条例

第一章　总则

　　第一条　为了加强病原微生物实验室(以下称实验室)生物安全管理，保护实验室工作人员和公众的健康，制定本条例。

　　第二条　对中华人民共和国境内的实验室及其从事实验活动的生物安全管理，适用本条例。

　　本条例所称病原微生物，是指能够使人或者动物致病的微生物。

　　本条例所称实验活动，是指实验室从事与病原微生物菌(毒)种、样本有关的研究、教学、检测、诊断等活动。

　　第三条　国务院卫生主管部门主管与人体健康有关的实验室及其实验活动的生物安全监督工作。

　　国务院兽医主管部门主管与动物有关的实验室及其实验活动的生物安全监督工作。

　　国务院其他有关部门在各自职责范围内负责实验室及其实验活动的生物安全管理工作。

　　县级以上地方人民政府及其有关部门在各自职责范围内负责实验室及其实验活动的生物安全管理工作。

　　第四条　国家对病原微生物实行分类管理，对实验室实行分级管理。

　　第五条　国家实行统一的实验室生物安全标准。实验室应当符合国家标准和要求。

　　第六条　实验室的设立单位及其主管部门负责实验室日常活动的管理，承担建立健全安全管理制度，检查、维护实验设施、设备，控制实验室感染的职责。

第二章　病原微生物的分类和管理

　　第七条　国家根据病原微生物的传染性、感染后对个体或者群体的危害程度，将病原微生物分为四类：

　　第一类病原微生物，是指能够引起人类或者动物非常严重疾病的微生物，以及我国尚未发现或者已经宣布消灭的微生物。

　　第二类病原微生物，是指能够引起人类或者动物严重疾病，比较容易直接或者间接在人与人、动物与人、动物与动物间传播的微生物。

　　第三类病原微生物，是指能够引起人类或者动物疾病，但一般情况下对人、动物或者环境不构成严重危害，传播风险有限，实验室感染后很少引起严重疾病，并且具备有效治疗和预防措施的微生物。

　　第四类病原微生物，是指在通常情况下不会引起人类或者动物疾病的微生物。

　　第一类、第二类病原微生物统称为高致病性病原微生物。

　　第八条　人间传染的病原微生物名录由国务院卫生主管部门商国务院有关部门后制定、调整并予以公布；动物间传染的病原微生物名录由国务院兽医主管部门商国务院有关部门后制定、调整并予以公布。

　　第九条　采集病原微生物样本应当具备下列条件：

　　(一)具有与采集病原微生物样本所需要的生物安全防护水平相适应的设备；

　　(二)具有掌握相关专业知识和操作技能的工作人员；

　　(三)具有有效的防止病原微生物扩散和感染的措施；

　　(四)具有保证病原微生物样本质量的技术方法和手段。

　　采集高致病性病原微生物样本的工作人员在采集过程中应当防止病原微生物扩散和感染，并对样本的来源、采集过程和方法等作详细记录。

　　第十条　运输高致病性病原微生物菌(毒)种或者样本，应当通过陆路运输；没有陆路通道，必须经水路运输的，可以通过水路运输；紧急情况下或者需要将高致病性病原微生物菌(毒)种或者样本运往国外的，可以通过民用航空运输。

　　第十一条　运输高致病性病原微生物菌(毒)种或者样本，应当具备下列条件：

　　(一)运输目的、高致病性病原微生物的用途和接收单位符合国务院卫生主管部门或者兽医主管部门的规定；

　　(二)高致病性病原微生物菌(毒)种或者样本的容器应当密封，容器或者包装材料还应当符合防水、防破损、防外泄、耐高(低)温、耐高压的要求；

　　(三)容器或者包装材料上应当印有国务院卫生主管部门或者兽医主管部门规定的生物危险标识、警告用语和提示用语。

　　运输高致病性病原微生物菌(毒)种或者样本，应当经省级以上人民政府卫生主管部门或者兽医主管部门批准。在省、自治区、直辖市行政区域内运输的，由省、自治区、直辖市人民政府卫生主管部门或者兽医主管部门批准；需要跨省、自治区、直辖市运输或者运往国外的，由出发地的省、自治区、直辖市人民政府卫生主管部门或者兽医主管部门进行初审后，分别报国务院卫生主管部门或者兽医主管部门批准。

　　出入境检验检疫机构在检验检疫过程中需要运输病原微生物样本的，由国务院出入境检验检疫部门批准，并同时向国务院卫生主管部门或者兽医主管部门通报。

　　通过民用航空运输高致病性病原微生物菌(毒)种或者样本的，除依照本条第二款、第三款规定取得批准外，还应当经国务院民用航空主管部门批准。

　　有关主管部门应当对申请人提交的关于运输高致病性病原微生物菌(毒)种或者样本的申请材料进行审查，对符合本条第一款规定条件的，应当即时批准。

　　第十二条　运输高致病性病原微生物菌(毒)种或者样本，应当由不少于2人的专人护送，并采取相应的防护措施。

　　有关单位或者个人不得通过公共电(汽)车和城市铁路运输病原微生物菌(毒)种或者样本。

　　第十三条　需要通过铁路、公路、民用航空等公共交通工具运输高致病性病原微生物菌(毒)种或者样本的，承运单位应当凭本条例第十一条规定的批准文件予以运输。

　　承运单位应当与护送人共同采取措施，确保所运输的高致病性病原微生物菌(毒)种或者样本的安全，严防发生被盗、被抢、丢失、泄漏事件。

　　第十四条　国务院卫生主管部门或者兽医主管部门指定的菌(毒)种保藏中心或者专业实验室(以下称保藏机构)，承担集中储存病原微生物菌(毒)种和样本的任务。

　　保藏机构应当依照国务院卫生主管部门或者兽医主管部门的规定，储存实验室送交的病原微生物菌(毒)种和样本，并向实验室提供病原微生物菌(毒)种和样本。

　　保藏机构应当制定严格的安全保管制度，作好病原微生物菌(毒)种和样本进出和储存的记录，建立档案制度，并指定专人负责。对高致病性病原微生物菌(毒)种和样本应当设专库或者专柜单独储存。

　　保藏机构储存、提供病原微生物菌(毒)种和样本，不得收取任何费用，其经费由同级财政在单位预算中予以保障。

　　保藏机构的管理办法由国务院卫生主管部门会同国务院兽医主管部门制定。

　　第十五条　保藏机构应当凭实验室依照本条例的规定取得的从事高致病性病原微生物相关实验活动的批准文件，向实验室提供高致病性病原微生物菌(毒)种和样本，并予以登记。

　　第十六条　实验室在相关实验活动结束后，应当依照国务院卫生主管部门或者兽医主管部门的规定，及时将病原微生物菌(毒)种和样本就地销毁或者送交保藏机构保管。

　　保藏机构接受实验室送交的病原微生物菌(毒)种和样本，应当予以登记，并开具接收证明。

　　第十七条　高致病性病原微生物菌(毒)种或者样本在运输、储存中被盗、被抢、丢失、泄漏的，承运单位、护送人、保藏机构应当采取必要的控制措施，并在2小时内分别向承运单位的主管部门、护送人所在单位和保藏机构的主管部门报告，同时向所在地的县级人民政府卫生主管部门或者兽医主管部门报告，发生被盗、被抢、丢失的，还应当向公安机关报告；接到报告的卫生主管部门或者兽医主管部门应当在2小时内向本级人民政府报告，并同时向上级人民政府卫生主管部门或者兽医主管部门和国务院卫生主管部门或者兽医主管部门报告。

　　县级人民政府应当在接到报告后2小时内向设区的市级人民政府或者上一级人民政府报告；设区的市级人民政府应当在接到报告后2小时内向省、自治区、直辖市人民政府报告。省、自治区、直辖市人民政府应当在接到报告后1小时内，向国务院卫生主管部门或者兽医主管部门报告。

　　任何单位和个人发现高致病性病原微生物菌(毒)种或者样本的容器或者包装材料，应当及时向附近的卫生主管部门或者兽医主管部门报告；接到报告的卫生主管部门或者兽医主管部门应当及时组织调查核实，并依法采取必要的控制措施。

第三章　实验室的设立与管理

　　第十八条　国家根据实验室对病原微生物的生物安全防护水平，并依照实验室生物安全国家标准的规定，将实验室分为一级、二级、三级、四级。

　　第十九条　新建、改建、扩建三级、四级实验室或者生产、进口移动式三级、四级实验室应当遵守下列规定：

　　(一)符合国家生物安全实验室体系规划并依法履行有关审批手续；

　　(二)经国务院科技主管部门审查同意；

　　(三)符合国家生物安全实验室建筑技术规范；

　　(四)依照《中华人民共和国环境影响评价法》的规定进行环境影响评价并经环境保护主管部门审查批准；

　　(五)生物安全防护级别与其拟从事的实验活动相适应。

　　前款规定所称国家生物安全实验室体系规划，由国务院投资主管部门会同国务院有关部门制定。制定国家生物安全实验室体系规划应当遵循总量控制、合理布局、资源共享的原则，并应当召开听证会或者论证会，听取公共卫生、环境保护、投资管理和实验室管理等方面专家的意见。

　　第二十条　三级、四级实验室应当通过实验室国家认可。

　　国务院认证认可监督管理部门确定的认可机构应当依照实验室生物安全国家标准以及本条例的有关规定，对三级、四级实验室进行认可；实验室通过认可的，颁发相应级别的生物安全实验室证书。证书有效期为5年。

　　第二十一条　一级、二级实验室不得从事高致病性病原微生物实验活动。三级、四级实验室从事高致病性病原微生物实验活动，应当具备下列条件：

　　(一)实验目的和拟从事的实验活动符合国务院卫生主管部门或者兽医主管部门的规定；

　　(二)通过实验室国家认可；

　　(三)具有与拟从事的实验活动相适应的工作人员；

　　(四)工程质量经建筑主管部门依法检测验收合格。

　　第二十二条　三级、四级实验室，需要从事某种高致病性病原微生物或者疑似高致病性病原微生物实验活动的，应当依照国务院卫生主管部门或者兽医主管部门的规定报省级以上人民政府卫生主管部门或者兽医主管部门批准。实验活动结果以及工作情况应当向原批准部门报告。

　　实验室申报或者接受与高致病性病原微生物有关的科研项目，应当符合科研需要和生物安全要求，具有相应的生物安全防护水平。与动物间传染的高致病性病原微生物有关的科研项目，应当经国务院兽医主管部门同意；与人体健康有关的高致病性病原微生物科研项目，实验室应当将立项结果告知省级以上人民政府卫生主管部门。

　　第二十三条　出入境检验检疫机构、医疗卫生机构、动物防疫机构在实验室开展检测、诊断工作时，发现高致病性病原微生物或者疑似高致病性病原微生物，需要进一步从事这类高致病性病原微生物相关实验活动的，应当依照本条例的规定经批准同意，并在具备相应条件的实验室中进行。

　　专门从事检测、诊断的实验室应当严格依照国务院卫生主管部门或者兽医主管部门的规定，建立健全规章制度，保证实验室生物安全。

　　第二十四条　省级以上人民政府卫生主管部门或者兽医主管部门应当自收到需要从事高致病性病原微生物相关实验活动的申请之日起15日内作出是否批准的决定。

　　对出入境检验检疫机构为了检验检疫工作的紧急需要，申请在实验室对高致病性病原微生物或者疑似高致病性病原微生物开展进一步实验活动的，省级以上人民政府卫生主管部门或者兽医主管部门应当自收到申请之时起2小时内作出是否批准的决定；2小时内未作出决定的，实验室可以从事相应的实验活动。

　　省级以上人民政府卫生主管部门或者兽医主管部门应当为申请人通过电报、电传、传真、电子数据交换和电子邮件等方式提出申请提供方便。

　　第二十五条　新建、改建或者扩建一级、二级实验室，应当向设区的市级人民政府卫生主管部门或者兽医主管部门备案。设区的市级人民政府卫生主管部门或者兽医主管部门应当每年将备案情况汇总后报省、自治区、直辖市人民政府卫生主管部门或者兽医主管部门。

　　第二十六条　国务院卫生主管部门和兽医主管部门应当定期汇总并互相通报实验室数量和实验室设立、分布情况，以及三级、四级实验室从事高致病性病原微生物实验活动的情况。

　　第二十七条　已经建成并通过实验室国家认可的三级、四级实验室应当向所在地的县级人民政府环境保护主管部门备案。环境保护主管部门依照法律、行政法规的规定对实验室排放的废水、废气和其他废物处置情况进行监督检查。

　　第二十八条　对我国尚未发现或者已经宣布消灭的病原微生物，任何单位和个人未经批准不得从事相关实验活动。

　　为了预防、控制传染病，需要从事前款所指病原微生物相关实验活动的，应当经国务院卫生主管部门或者兽医主管部门批准，并在批准部门指定的专业实验室中进行。

　　第二十九条　实验室使用新技术、新方法从事高致病性病原微生物相关实验活动的，应当符合防止高致病性病原微生物扩散、保证生物安全和操作者人身安全的要求，并经国家病原微生物实验室生物安全专家委员会论证；经论证可行的，方可使用。

　　第三十条　需要在动物体上从事高致病性病原微生物相关实验活动的，应当在符合动物实验室生物安全国家标准的三级以上实验室进行。

　　第三十一条　实验室的设立单位负责实验室的生物安全管理。

　　实验室的设立单位应当依照本条例的规定制定科学、严格的管理制度，并定期对有关生物安全规定的落实情况进行检查，定期对实验室设施、设备、材料等进行检查、维护和更新，以确保其符合国家标准。

　　实验室的设立单位及其主管部门应当加强对实验室日常活动的管理。

　　第三十二条　实验室负责人为实验室生物安全的第一责任人。

　　实验室从事实验活动应当严格遵守有关国家标准和实验室技术规范、操作规程。实验室负责人应当指定专人监督检查实验室技术规范和操作规程的落实情况。

　　第三十三条　从事高致病性病原微生物相关实验活动的实验室的设立单位，应当建立健全安全保卫制度，采取安全保卫措施，严防高致病性病原微生物被盗、被抢、丢失、泄漏，保障实验室及其病原微生物的安全。实验室发生高致病性病原微生物被盗、被抢、丢失、泄漏的，实验室的设立单位应当依照本条例第十七条的规定进行报告。

　　从事高致病性病原微生物相关实验活动的实验室应当向当地公安机关备案，并接受公安机关有关实验室安全保卫工作的监督指导。

　　第三十四条　实验室或者实验室的设立单位应当每年定期对工作人员进行培训，保证其掌握实验室技术规范、操作规程、生物安全防护知识和实际操作技能，并进行考核。工作人员经考核合格的，方可上岗。

　　从事高致病性病原微生物相关实验活动的实验室，应当每半年将培训、考核其工作人员的情况和实验室运行情况向省、自治区、直辖市人民政府卫生主管部门或者兽医主管部门报告。

　　第三十五条　从事高致病性病原微生物相关实验活动应当有2名以上的工作人员共同进行。

　　进入从事高致病性病原微生物相关实验活动的实验室的工作人员或者其他有关人员，应当经实验室负责人批准。实验室应当为其提供符合防护要求的防护用品并采取其他职业防护措施。从事高致病性病原微生物相关实验活动的实验室，还应当对实验室工作人员进行健康监测，每年组织对其进行体检，并建立健康档案；必要时，应当对实验室工作人员进行预防接种。

　　第三十六条　在同一个实验室的同一个独立安全区域内，只能同时从事一种高致病性病原微生物的相关实验活动。

　　第三十七条　实验室应当建立实验档案，记录实验室使用情况和安全监督情况。实验室从事高致病性病原微生物相关实验活动的实验档案保存期，不得少于20年。

　　第三十八条　实验室应当依照环境保护的有关法律、行政法规和国务院有关部门的规定，对废水、废气以及其他废物进行处置，并制定相应的环境保护措施，防止环境污染。

　　第三十九条　三级、四级实验室应当在明显位置标示国务院卫生主管部门和兽医主管部门规定的生物危险标识和生物安全实验室级别标志。

　　第四十条　从事高致病性病原微生物相关实验活动的实验室应当制定实验室感染应急处置预案，并向该实验室所在地的省、自治区、直辖市人民政府卫生主管部门或者兽医主管部门备案。

　　第四十一条　国务院卫生主管部门和兽医主管部门会同国务院有关部门组织病原学、免疫学、检验医学、流行病学、预防兽医学、环境保护和实验室管理等方面的专家，组成国家病原微生物实验室生物安全专家委员会。该委员会承担从事高致病性病原微生物相关实验活动的实验室的设立与运行的生物安全评估和技术咨询、论证工作。

　　省、自治区、直辖市人民政府卫生主管部门和兽医主管部门会同同级人民政府有关部门组织病原学、免疫学、检验医学、流行病学、预防兽医学、环境保护和实验室管理等方面的专家，组成本地区病原微生物实验室生物安全专家委员会。该委员会承担本地区实验室设立和运行的技术咨询工作。

第四章　实验室感染控制

　　第四十二条　实验室的设立单位应当指定专门的机构或者人员承担实验室感染控制工作，定期检查实验室的生物安全防护、病原微生物菌(毒)种和样本保存与使用、安全操作、实验室排放的废水和废气以及其他废物处置等规章制度的实施情况。

　　负责实验室感染控制工作的机构或者人员应当具有与该实验室中的病原微生物有关的传染病防治知识，并定期调查、了解实验室工作人员的健康状况。

　　第四十三条　实验室工作人员出现与本实验室从事的高致病性病原微生物相关实验活动有关的感染临床症状或者体征时，实验室负责人应当向负责实验室感染控制工作的机构或者人员报告，同时派专人陪同及时就诊；实验室工作人员应当将近期所接触的病原微生物的种类和危险程度如实告知诊治医疗机构。接诊的医疗机构应当及时救治；不具备相应救治条件的，应当依照规定将感染的实验室工作人员转诊至具备相应传染病救治条件的医疗机构；具备相应传染病救治条件的医疗机构应当接诊治疗，不得拒绝救治。

　　第四十四条　实验室发生高致病性病原微生物泄漏时，实验室工作人员应当立即采取控制措施，防止高致病性病原微生物扩散，并同时向负责实验室感染控制工作的机构或者人员报告。

　　第四十五条　负责实验室感染控制工作的机构或者人员接到本条例第四十三条、第四十四条规定的报告后，应当立即启动实验室感染应急处置预案，并组织人员对该实验室生物安全状况等情况进行调查；确认发生实验室感染或者高致病性病原微生物泄漏的，应当依照本条例第十七条的规定进行报告，并同时采取控制措施，对有关人员进行医学观察或者隔离治疗，封闭实验室，防止扩散。

　　第四十六条　卫生主管部门或者兽医主管部门接到关于实验室发生工作人员感染事故或者病原微生物泄漏事件的报告，或者发现实验室从事病原微生物相关实验活动造成实验室感染事故的，应当立即组织疾病预防控制机构、动物防疫监督机构和医疗机构以及其他有关机构依法采取下列预防、控制措施：

　　(一)封闭被病原微生物污染的实验室或者可能造成病原微生物扩散的场所；

　　(二)开展流行病学调查；

　　(三)对病人进行隔离治疗，对相关人员进行医学检查；

　　(四)对密切接触者进行医学观察；

　　(五)进行现场消毒；

　　(六)对染疫或者疑似染疫的动物采取隔离、扑杀等措施；

　　(七)其他需要采取的预防、控制措施。

　　第四十七条　医疗机构或者兽医医疗机构及其执行职务的医务人员发现由于实验室感染而引起的与高致病性病原微生物相关的传染病病人、疑似传染病病人或者患有疫病、疑似患有疫病的动物，诊治的医疗机构或者兽医医疗机构应当在2小时内报告所在地的县级人民政府卫生主管部门或者兽医主管部门；接到报告的卫生主管部门或者兽医主管部门应当在2小时内通报实验室所在地的县级人民政府卫生主管部门或者兽医主管部门。接到通报的卫生主管部门或者兽医主管部门应当依照本条例第四十六条的规定采取预防、控制措施。

　　第四十八条　发生病原微生物扩散，有可能造成传染病暴发、流行时，县级以上人民政府卫生主管部门或者兽医主管部门应当依照有关法律、行政法规的规定以及实验室感染应急处置预案进行处理。

第五章　监督管理

　　第四十九条　县级以上地方人民政府卫生主管部门、兽医主管部门依照各自分工，履行下列职责：

　　(一)对病原微生物菌(毒)种、样本的采集、运输、储存进行监督检查；

　　(二)对从事高致病性病原微生物相关实验活动的实验室是否符合本条例规定的条件进行监督检查；

　　(三)对实验室或者实验室的设立单位培训、考核其工作人员以及上岗人员的情况进行监督检查；

　　(四)对实验室是否按照有关国家标准、技术规范和操作规程从事病原微生物相关实验活动进行监督检查。

　　县级以上地方人民政府卫生主管部门、兽医主管部门，应当主要通过检查反映实验室执行国家有关法律、行政法规以及国家标准和要求的记录、档案、报告，切实履行监督管理职责。

　　第五十条　县级以上人民政府卫生主管部门、兽医主管部门、环境保护主管部门在履行监督检查职责时，有权进入被检查单位和病原微生物泄漏或者扩散现场调查取证、采集样品，查阅复制有关资料。需要进入从事高致病性病原微生物相关实验活动的实验室调查取证、采集样品的，应当指定或者委托专业机构实施。被检查单位应当予以配合，不得拒绝、阻挠。

　　第五十一条　国务院认证认可监督管理部门依照《中华人民共和国认证认可条例》的规定对实验室认可活动进行监督检查。

　　第五十二条　卫生主管部门、兽医主管部门、环境保护主管部门应当依据法定的职权和程序履行职责，做到公正、公平、公开、文明、高效。

　　第五十三条　卫生主管部门、兽医主管部门、环境保护主管部门的执法人员执行职务时，应当有2名以上执法人员参加，出示执法证件，并依照规定填写执法文书。

　　现场检查笔录、采样记录等文书经核对无误后，应当由执法人员和被检查人、被采样人签名。被检查人、被采样人拒绝签名的，执法人员应当在自己签名后注明情况。

　　第五十四条　卫生主管部门、兽医主管部门、环境保护主管部门及其执法人员执行职务，应当自觉接受社会和公民的监督。公民、法人和其他组织有权向上级人民政府及其卫生主管部门、兽医主管部门、环境保护主管部门举报地方人民政府及其有关主管部门不依照规定履行职责的情况。接到举报的有关人民政府或者其卫生主管部门、兽医主管部门、环境保护主管部门，应当及时调查处理。

　　第五十五条　上级人民政府卫生主管部门、兽医主管部门、环境保护主管部门发现属于下级人民政府卫生主管部门、兽医主管部门、环境保护主管部门职责范围内需要处理的事项的，应当及时告知该部门处理；下级人民政府卫生主管部门、兽医主管部门、环境保护主管部门不及时处理或者不积极履行本部门职责的，上级人民政府卫生主管部门、兽医主管部门、环境保护主管部门应当责令其限期改正；逾期不改正的，上级人民政府卫生主管部门、兽医主管部门、环境保护主管部门有权直接予以处理。

第六章　法律责任

　　第五十六条　三级、四级实验室未经批准从事某种高致病性病原微生物或者疑似高致病性病原微生物实验活动的，由县级以上地方人民政府卫生主管部门、兽医主管部门依照各自职责，责令停止有关活动，监督其将用于实验活动的病原微生物销毁或者送交保藏机构，并给予警告；造成传染病传播、流行或者其他严重后果的，由实验室的设立单位对主要负责人、直接负责的主管人员和其他直接责任人员，依法给予撤职、开除的处分；构成犯罪的，依法追究刑事责任。

　　第五十七条　卫生主管部门或者兽医主管部门违反本条例的规定，准予不符合本条例规定条件的实验室从事高致病性病原微生物相关实验活动的，由作出批准决定的卫生主管部门或者兽医主管部门撤销原批准决定，责令有关实验室立即停止有关活动，并监督其将用于实验活动的病原微生物销毁或者送交保藏机构，对直接负责的主管人员和其他直接责任人员依法给予行政处分；构成犯罪的，依法追究刑事责任。

　　因违法作出批准决定给当事人的合法权益造成损害的，作出批准决定的卫生主管部门或者兽医主管部门应当依法承担赔偿责任。

　　第五十八条　卫生主管部门或者兽医主管部门对出入境检验检疫机构为了检验检疫工作的紧急需要，申请在实验室对高致病性病原微生物或者疑似高致病性病原微生物开展进一步检测活动，不在法定期限内作出是否批准决定的，由其上级行政机关或者监察机关责令改正，给予警告；造成传染病传播、流行或者其他严重后果的，对直接负责的主管人员和其他直接责任人员依法给予撤职、开除的行政处分；构成犯罪的，依法追究刑事责任。

　　第五十九条　违反本条例规定，在不符合相应生物安全要求的实验室从事病原微生物相关实验活动的，由县级以上地方人民政府卫生主管部门、兽医主管部门依照各自职责，责令停止有关活动，监督其将用于实验活动的病原微生物销毁或者送交保藏机构，并给予警告；造成传染病传播、流行或者其他严重后果的，由实验室的设立单位对主要负责人、直接负责的主管人员和其他直接责任人员，依法给予撤职、开除的处分；构成犯罪的，依法追究刑事责任。

　　第六十条　实验室有下列行为之一的，由县级以上地方人民政府卫生主管部门、兽医主管部门依照各自职责，责令限期改正，给予警告；逾期不改正的，由实验室的设立单位对主要负责人、直接负责的主管人员和其他直接责任人员，依法给予撤职、开除的处分；有许可证件的，并由原发证部门吊销有关许可证件：

　　(一)未依照规定在明显位置标示国务院卫生主管部门和兽医主管部门规定的生物危险标识和生物安全实验室级别标志的；

　　(二)未向原批准部门报告实验活动结果以及工作情况的；

　　(三)未依照规定采集病原微生物样本，或者对所采集样本的来源、采集过程和方法等未作详细记录的；

　　(四)新建、改建或者扩建一级、二级实验室未向设区的市级人民政府卫生主管部门或者兽医主管部门备案的；

　　(五)未依照规定定期对工作人员进行培训，或者工作人员考核不合格允许其上岗，或者批准未采取防护措施的人员进入实验室的；

　　(六)实验室工作人员未遵守实验室生物安全技术规范和操作规程的；

　　(七)未依照规定建立或者保存实验档案的；

　　(八)未依照规定制定实验室感染应急处置预案并备案的。

　　第六十一条　经依法批准从事高致病性病原微生物相关实验活动的实验室的设立单位未建立健全安全保卫制度，或者未采取安全保卫措施的，由县级以上地方人民政府卫生主管部门、兽医主管部门依照各自职责，责令限期改正；逾期不改正，导致高致病性病原微生物菌(毒)种、样本被盗、被抢或者造成其他严重后果的，责令停止该项实验活动，该实验室2年内不得申请从事高致病性病原微生物实验活动；造成传染病传播、流行的，该实验室设立单位的主管部门还应当对该实验室的设立单位的直接负责的主管人员和其他直接责任人员，依法给予降级、撤职、开除的处分；构成犯罪的，依法追究刑事责任。

　　第六十二条　未经批准运输高致病性病原微生物菌(毒)种或者样本，或者承运单位经批准运输高致病性病原微生物菌(毒)种或者样本未履行保护义务，导致高致病性病原微生物菌(毒)种或者样本被盗、被抢、丢失、泄漏的，由县级以上地方人民政府卫生主管部门、兽医主管部门依照各自职责，责令采取措施，消除隐患，给予警告；造成传染病传播、流行或者其他严重后果的，由托运单位和承运单位的主管部门对主要负责人、直接负责的主管人员和其他直接责任人员，依法给予撤职、开除的处分；构成犯罪的，依法追究刑事责任。

　　第六十三条　有下列行为之一的，由实验室所在地的设区的市级以上地方人民政府卫生主管部门、兽医主管部门依照各自职责，责令有关单位立即停止违法活动，监督其将病原微生物销毁或者送交保藏机构；造成传染病传播、流行或者其他严重后果的，由其所在单位或者其上级主管部门对主要负责人、直接负责的主管人员和其他直接责任人员，依法给予撤职、开除的处分；有许可证件的，并由原发证部门吊销有关许可证件；构成犯罪的，依法追究刑事责任：

　　(一)实验室在相关实验活动结束后，未依照规定及时将病原微生物菌(毒)种和样本就地销毁或者送交保藏机构保管的；

　　(二)实验室使用新技术、新方法从事高致病性病原微生物相关实验活动未经国家病原微生物实验室生物安全专家委员会论证的；

　　(三)未经批准擅自从事在我国尚未发现或者已经宣布消灭的病原微生物相关实验活动的；

　　(四)在未经指定的专业实验室从事在我国尚未发现或者已经宣布消灭的病原微生物相关实验活动的；

　　(五)在同一个实验室的同一个独立安全区域内同时从事两种或者两种以上高致病性病原微生物的相关实验活动的。

　　第六十四条　认可机构对不符合实验室生物安全国家标准以及本条例规定条件的实验室予以认可，或者对符合实验室生物安全国家标准以及本条例规定条件的实验室不予认可的，由国务院认证认可监督管理部门责令限期改正，给予警告；造成传染病传播、流行或者其他严重后果的，由国务院认证认可监督管理部门撤销其认可资格，有上级主管部门的，由其上级主管部门对主要负责人、直接负责的主管人员和其他直接责任人员依法给予撤职、开除的处分；构成犯罪的，依法追究刑事责任。

　　第六十五条　实验室工作人员出现该实验室从事的病原微生物相关实验活动有关的感染临床症状或者体征，以及实验室发生高致病性病原微生物泄漏时，实验室负责人、实验室工作人员、负责实验室感染控制的专门机构或者人员未依照规定报告，或者未依照规定采取控制措施的，由县级以上地方人民政府卫生主管部门、兽医主管部门依照各自职责，责令限期改正，给予警告；造成传染病传播、流行或者其他严重后果的，由其设立单位对实验室主要负责人、直接负责的主管人员和其他直接责任人员，依法给予撤职、开除的处分；有许可证件的，并由原发证部门吊销有关许可证件；构成犯罪的，依法追究刑事责任。

　　第六十六条　拒绝接受卫生主管部门、兽医主管部门依法开展有关高致病性病原微生物扩散的调查取证、采集样品等活动或者依照本条例规定采取有关预防、控制措施的，由县级以上人民政府卫生主管部门、兽医主管部门依照各自职责，责令改正，给予警告；造成传染病传播、流行以及其他严重后果的，由实验室的设立单位对实验室主要负责人、直接负责的主管人员和其他直接责任人员，依法给予降级、撤职、开除的处分；有许可证件的，并由原发证部门吊销有关许可证件；构成犯罪的，依法追究刑事责任。

　　第六十七条　发生病原微生物被盗、被抢、丢失、泄漏，承运单位、护送人、保藏机构和实验室的设立单位未依照本条例的规定报告的，由所在地的县级人民政府卫生主管部门或者兽医主管部门给予警告；造成传染病传播、流行或者其他严重后果的，由实验室的设立单位或者承运单位、保藏机构的上级主管部门对主要负责人、直接负责的主管人员和其他直接责任人员，依法给予撤职、开除的处分；构成犯罪的，依法追究刑事责任。

　　第六十八条　保藏机构未依照规定储存实验室送交的菌(毒)种和样本，或者未依照规定提供菌(毒)种和样本的，由其指定部门责令限期改正，收回违法提供的菌(毒)种和样本，并给予警告；造成传染病传播、流行或者其他严重后果的，由其所在单位或者其上级主管部门对主要负责人、直接负责的主管人员和其他直接责任人员，依法给予撤职、开除的处分；构成犯罪的，依法追究刑事责任。

　　第六十九条　县级以上人民政府有关主管部门，未依照本条例的规定履行实验室及其实验活动监督检查职责的，由有关人民政府在各自职责范围内责令改正，通报批评；造成传染病传播、流行或者其他严重后果的，对直接负责的主管人员，依法给予行政处分；构成犯罪的，依法追究刑事责任。

第七章　附则

　　第七十条　军队实验室由中国人民解放军卫生主管部门参照本条例负责监督管理。

　　第七十一条　本条例施行前设立的实验室，应当自本条例施行之日起6个月内，依照本条例的规定，办理有关手续。

　　第七十二条　本条例自公布之日起施行。

1. 科学技术部：高等级病原微生物实验室建设审查办法

第一章 总 则

第一条 为规范三级、四级病原微生物实验室（以下简称实验室）建设审查，根据《病原微生物实验室生物安全管理条例》（国务院令第424号）的有关规定，制定本办法。

第二条 新建、改建、扩建实验室或者生产、进口移动式实验室应当报科学技术部审查同意。

第二章 申 请

第三条 向科学技术部申请建设实验室，应当符合下列条件：

（一）符合国家生物安全实验室体系建设规划要求；

（二）开展实验室建设确属必要；

（三）具备保障实验室规范安全运行的能力和机制；

（四）符合法律法规规定的其他条件。

第四条 符合第三条规定的申请单位向科学技术部提交《高等级病原微生物实验室建设审查申请书》（以下简称《申请书》）。《申请书》应当经其所在地的省级人民政府或按照行政隶属关系经国务院有关部门盖章同意。

第三章 审 查

第五条 科学技术部设立实验室建设审查专家委员会（以下简称专家委员会）。专家委员会由从事实验室管理及相关领域科研工作的专家组成，任期5年。

专家委员会对实验室建设审查工作提供咨询意见。

专家委员会专家受科学技术部委托组成专家组，对每个具体实验室建设申请进行审查并提出专家组审查意见。专家组一般不少于5人。

第六条 科学技术部将组织专家组对实验室建设申请进行专家审查。基本程序包括：

（一）资料审核；

（二）申请单位陈述；

（三）现场勘查（现场审查方式）；

（四）专家提问；

（五）讨论形成专家组审查意见。

第七条 专家组成员应遵守诚信和回避制度，客观、公正地开展工作。

第八条 科学技术部根据专家组审查意见，经科学技术部部务会审定同意后形成科学技术部审查决定。

第九条 科学技术部自收到申请单位报送的申请材料后，应当在5个工作日内完成形式审查。申请材料不齐全或不符合规定形式的，科学技术部应当在5个工作日内退回申请单位。申请材料齐全并符合规定形式的，科学技术部自受理之日起20个工作日内作出批准或者不予批准的决定；不予批准的应当说明理由。因特殊原因无法在规定期限内做出审查决定的，经科学技术部负责人批准可以延长10个工作日，并将延长期限的理由告知申请单位。

第十条 申请单位对科学技术部审查决定有异议的，可自收到通知之日起15个工作日内以书面形式向科学技术部申请复核。科学技术部将组织专家进行复核审查。

第十一条 实验室建设申请单位在实验室建设审查过程中存在弄虚作假等行为的，科学技术部将终止对其申请的审查或撤销已作出的审查决定，书面通知申请单位，并根据情节轻重决定在1-3年内不再受理其申请。

第四章 附 则

第十二条 实验室建设申请与审查过程中涉及国家秘密的，应严格按照有关保密规定执行。

第十三条 通过建设审查的实验室建成后，依据《病原微生物实验室生物安全管理条例》，由有关部门根据相关规定进行建筑质量验收、建设项目竣工环境保护验收、实验室国家认可和实验活动审批及监管等，确保实验室安全。

第十四条 实验室建设申请与审查工作不收取任何费用。

第十五条 本办法自2018年10月31日起施行。

第十六条 本办法由科学技术部负责解释。

1. 全国人民代表大会常务委员会：中华人民共和国生物安全法

病原微生物实验室生物安全

国家加强对病原微生物实验室生物安全的管理，制定统一的实验室生物安全标准。病原微生物实验室应当符合生物安全国家标准和要求。

从事病原微生物实验活动，应当严格遵守有关国家标准和实验室技术规范、操作规程，采取安全防范措施。

国家根据病原微生物的传染性、感染后对人和动物的个体或者群体的危害程度，对病原微生物实行分类管理。

从事高致病性或者疑似高致病性病原微生物样本采集、保藏、运输活动，应当具备相应条件，符合生物安全管理规范。具体办法由国务院卫生健康、农业农村主管部门制定。

设立病原微生物实验室，应当依法取得批准或者进行备案。

个人不得设立病原微生物实验室或者从事病原微生物实验活动。

国家根据对病原微生物的生物安全防护水平，对病原微生物实验室实行分等级管理。

从事病原微生物实验活动应当在相应等级的实验室进行。低等级病原微生物实验室不得从事国家病原微生物目录规定应当在高等级病原微生物实验室进行的病原微生物实验活动。

高等级病原微生物实验室从事高致病性或者疑似高致病性病原微生物实验活动，应当经省级以上人民政府卫生健康或者农业农村主管部门批准，并将实验活动情况向批准部门报告。

对我国尚未发现或者已经宣布消灭的病原微生物，未经批准不得从事相关实验活动。

病原微生物实验室应当采取措施，加强对实验动物的管理，防止实验动物逃逸，对使用后的实验动物按照国家规定进行无害化处理，实现实验动物可追溯。禁止将使用后的实验动物流入市场。

病原微生物实验室应当加强对实验活动废弃物的管理，依法对废水、废气以及其他废弃物进行处置，采取措施防止污染。

病原微生物实验室的设立单位负责实验室的生物安全管理，制定科学、严格的管理制度，定期对有关生物安全规定的落实情况进行检查，对实验室设施、设备、材料等进行检查、维护和更新，确保其符合国家标准。

病原微生物实验室设立单位的法定代表人和实验室负责人对实验室的生物安全负责。

病原微生物实验室的设立单位应当建立和完善安全保卫制度，采取安全保卫措施，保障实验室及其病原微生物的安全。

国家加强对高等级病原微生物实验室的安全保卫。高等级病原微生物实验室应当接受公安机关等部门有关实验室安全保卫工作的监督指导，严防高致病性病原微生物泄漏、丢失和被盗、被抢。

国家建立高等级病原微生物实验室人员进入审核制度。进入高等级病原微生物实验室的人员应当经实验室负责人批准。对可能影响实验室生物安全的，不予批准；对批准进入的，应当采取安全保障措施。

病原微生物实验室的设立单位应当制定生物安全事件应急预案，定期组织开展人员培训和应急演练。发生高致病性病原微生物泄漏、丢失和被盗、被抢或者其他生物安全风险的，应当按照应急预案的规定及时采取控制措施，并按照国家规定报告。

病原微生物实验室所在地省级人民政府及其卫生健康主管部门应当加强实验室所在地感染性疾病医疗资源配置，提高感染性疾病医疗救治能力。

企业对涉及病原微生物操作的生产车间的生物安全管理，依照有关病原微生物实验室的规定和其他生物安全管理规范进行。

涉及生物毒素、植物有害生物及其他生物因子操作的生物安全实验室的建设和管理，参照有关病原微生物实验室的规定执行。

1. 生态环境部（原国家环境保护总局）：病原微生物实验室生物安全环境管理办法

第一条 为规范病原微生物实验室(以下简称“实验室”)生物安全环境管理工作，根据《病原微生物实验室生物安全管理条例》和有关环境保护法律和行政法规，制定本办法。

第二条 本办法适用于中华人民共和国境内的实验室及其从事实验活动的生物安全环境管理。

本办法所称的病原微生物，是指能够使人或者动物致病的微生物。

本办法所称的实验活动，是指实验室从事与病原微生物菌(毒)种、样品有关的研究、教学、检测、诊断等活动。

第三条 国家根据实验室对病原微生物的生物安全防护水平，并依照实验室生物安全国家标准的规定，将实验室分为一级、二级、三级和四级。

一级、二级实验室不得从事高致病性病原微生物实验活动。

第四条 国家环境保护总局制定并颁布实验室污染控制标准、环境管理技术规范和环境监督检查制度。

第五条 国家环境保护总局设立病原微生物实验室生物安全环境管理专家委员会。专家委员会主要由环境保护、病原微生物以及实验室管理方面的专家组成。

病原微生物实验室生物安全环境管理专家委员会的主要职责是：审议有关实验室污染控制标准和环境管理技术规范，提出审议建议；审查有关实验室环境影响评价文件，提出审查建议。

第六条 新建、改建、扩建实验室，应当按照国家环境保护规定,执行环境影响评价制度。

实验室环境影响评价文件应当对病原微生物实验活动对环境可能造成的影响进行分析和预测，并提出预防和控制措施。

第七条 新建、改建、扩建三级、四级实验室或者生产、进口移动式三级、四级实验室，应当编制环境影响报告书，并按照规定程序报国家环境保护总局审批。

承担三级、四级实验室环境影响评价工作的环境影响评价机构，应当具备甲级评价资质和相应的评价范围。

第八条 实验室应当按照国家环境保护规定、经审批的环境影响评价文件以及环境保护行政主管部门批复文件的要求，安装或者配备污染防治设施、设备。

污染防治设施、设备必须经环境保护行政主管部门验收合格后，实验室方可投入运行或者使用。

第九条 建成并通过国家认可的三级、四级实验室，应当在取得生物安全实验室证书后15日内填报三级、四级病原微生物实验室备案表（见附表），报所在地的县级人民政府环境保护行政主管部门。

第十条 县级人民政府环境保护行政主管部门应当自收到三级、四级病原微生物实验室备案表之日起10日内，报设区的市级人民政府环境保护行政主管部门；设区的市级人民政府环境保护行政主管部门应当自收到三级、四级病原微生物实验室备案表之日起10日内，报省级人民政府环境保护行政主管部门；省级人民政府环境保护行政主管部门应当自收到三级、四级病原微生物实验室备案表之日起10日内，报国家环境保护总局。

第十一条 实验室的设立单位对实验活动产生的废水、废气和危险废物承担污染防治责任。

实验室应当依照国家环境保护规定和实验室污染控制标准、环境管理技术规范的要求，建立、健全实验室废水、废气和危险废物污染防治管理的规章制度，并设置专（兼）职人员，对实验室产生的废水、废气及危险废物处置是否符合国家法律、行政法规及本办法规定的情况进行检查、督促和落实。

第十二条 实验室排放废水、废气的，应当按照国家环境保护总局的有关规定，执行排污申报登记制度。

实验室产生危险废物的，必须按照危险废物污染环境防治的有关规定，向所在地县级以上地方人民政府环境保护行政主管部门申报危险废物的种类、产生量、流向、贮存、处置等有关资料。

第十三条 实验室对其产生的废水，必须按照国家有关规定进行无害化处理；符合国家有关排放标准后，方可排放。

第十四条 实验室进行实验活动时，必须按照国家有关规定保证大气污染防治设施的正常运转；排放废气不得违反国家有关标准或者规定。

第十五条 实验室必须按照下列规定，妥善收集、贮存和处置其实验活动产生的危险废物，防止环境污染：

（一）建立危险废物登记制度，对其产生的危险废物进行登记。登记内容应当包括危险废物的来源、种类、重量或者数量、处置方法、最终去向以及经办人签名等项目。登记资料至少保存3年。

（二）及时收集其实验活动中产生的危险废物，并按照类别分别置于防渗漏、防锐器穿透等符合国家有关环境保护要求的专用包装物、容器内，并按国家规定要求设置明显的危险废物警示标识和说明。

（三）配备符合国家法律、行政法规和有关技术规范要求的危险废物暂时贮存柜（箱）或者其他设施、设备。

（四）按照国家有关规定对危险废物就地进行无害化处理，并根据就近集中处置的原则，及时将经无害化处理后的危险废物交由依法取得危险废物经营许可证的单位集中处置。

（五）转移危险废物的，应当按照《固体废物污染环境防治法》和国家环境保护总局的有关规定，执行危险废物转移联单制度。

（六）不得随意丢弃、倾倒、堆放危险废物，不得将危险废物混入其他废物和生活垃圾中。

（七）国家环境保护法律、行政法规和规章有关危险废物管理的其他要求。

第十六条 实验室建立并保留的实验档案应当如实记录与生物安全相关的实验活动和设施、设备工作状态情况，以及实验活动产生的废水、废气和危险废物无害化处理、集中处置以及检验的情况。

第十七条 实验室应当制定环境污染应急预案，报所在地县级人民政府环境保护行政主管部门备案，并定期进行演练。

实验室产生危险废物的，应当按照国家危险废物污染环境防治的规定，制定意外事故的防范措施和应急预案，并向所在地县级以上地方人民政府环境保护行政主管部门备案。

《病原微生物实验室生物安全管理条例》施行前已经投入使用的三级实验室,应当按照所在地县级人民政府环境保护行政主管部门的要求,限期制定环境污染应急预案和监测计划,并报环境保护行政主管部门备案。

第十八条 实验室发生泄露或者扩散，造成或者可能造成严重环境污染或者生态破坏的，应当立即采取应急措施，通报可能受到危害的单位和居民，并向当地人民政府环境保护行政主管部门和有关部门报告，接受调查处理。

当地人民政府环境保护行政主管部门应当按照国家环境保护总局污染事故报告程序规定报告上级人民政府环境保护行政主管部门。

第十九条 县级以上人民政府环境保护行政主管部门应当定期对管辖范围内的实验室废水、废气和危险废物的污染防治情况进行监督检查。发现有违法行为的，应当责令其限期整改。检查情况和处理结果应当予以记录，由检查人员签字后归档并反馈被检查单位。

第二十条 县级以上人民政府环境保护行政主管部门在履行监督检查职责时，有权进入被检查单位和病原微生物泄漏或者扩散现场调查取证，采集样品，查阅、复制有关资料，被检查单位应当予以配合，不得拒绝、阻挠。

需要进入三级或者四级实验室调查取证、采集样品的，应当指定或者委托专业机构实施。

环境保护行政主管部门应当为实验室保守技术秘密和业务秘密。

第二十一条 违反本办法有关规定，有下列情形之一的，由县级以上人民政府环境保护行政主管部门责令限期改正,给予警告;逾期不改正的,处1000元以下罚款：

（一）未建立实验室污染防治管理的规章制度，或者未设置专（兼）职人员的；

（二）未对产生的危险废物进行登记或者未保存登记资料的；

（三）未制定环境污染应急预案的。

违反本办法规定的其他行为，环境保护法律、行政法规已有处罚规定的,适用其规定。

第二十二条 环境保护行政主管部门应当及时向社会公告依据本办法被予以处罚的实验室名单，并将受到处罚的实验室名单通报中国实验室国家认可委员会。

1. 农业农村部：高致病性动物病原微生物实验室生物安全管理审批办法

第一章 总 则

第一条 为了规范高致病性动物病原微生物实验室生物安全管理的审批工作，根据《病原微生物实验室生物安全管理条例》，制定本办法。

第二条 高致病性动物病原微生物的实验室资格、实验活动和运输的审批，适用本办法。

第三条 本办法所称高致病性动物病原微生物是指来源于动物的、《动物病原微生物分类名录》中规定的第一类、第二类病原微生物。

《动物病原微生物分类名录》由农业部商国务院有关部门后制定、调整并予以公布。

第四条 农业部主管全国高致病性动物病原微生物实验室生物安全管理工作。

县级以上地方人民政府兽医行政管理部门负责本行政区域内高致病性动物病原微生物实验室生物安全管理工作。

第二章 实验室资格审批

第五条 实验室从事高致病性动物病原微生物实验活动，应当取得农业部颁发的《高致病性动物病原微生物实验室资格证书》。

第六条 实验室申请《高致病性动物病原微生物实验室资格证书》，应当具备下列条件：

（一）依法从事动物疫病的研究、检测、诊断，以及菌（毒）种保藏等活动；

（二）符合农业部颁发的《兽医实验室生物安全管理规范》；

（三）取得国家生物安全三级或者四级实验室认可证书；

（四）从事实验活动的工作人员具备兽医相关专业大专以上学历或中级以上技术职称，受过生物安全知识培训；

（五）实验室工程质量经依法检测验收合格。

第七条 符合前条规定条件的，申请人应当向所在地省、自治区、直辖市人民政府兽医行政管理部门提出申请，并提交下列材料：

（一）高致病性动物病原微生物实验室资格申请表一式两份；

（二）实验室管理手册；

（三）国家实验室认可证书复印件；

（四）实验室设立单位的法人资格证书复印件；

（五）实验室工作人员学历证书或者技术职称证书复印件；

（六）实验室工作人员生物安全知识培训情况证明材料；

（七）实验室工程质量检测验收报告复印件。

省、自治区、直辖市人民政府兽医行政管理部门应当自收到申请之日起10日内，将初审意见和有关材料报送农业部。

农业部收到初审意见和有关材料后，组织专家进行评审，必要时可到现场核实和评估。农业部自收到专家评审意见之日起10日内作出是否颁发《高致病性动物病原微生物实验室资格证书》的决定；不予批准的，及时告知申请人并说明理由。

第八条 《高致病性动物病原微生物实验室资格证书》有效期为5年。有效期届满，实验室需要继续从事高致病性动物病原微生物实验活动的，应当在届满6个月前，按照本办法的规定重新申请《高致病性动物病原微生物实验室资格证书》。

第三章 实验活动审批

第九条 一级、二级实验室不得从事高致病性动物病原微生物实验活动。三级、四级实验室需要从事某种高致病性动物病原微生物或者疑似高致病性动物病原微生物实验活动的，应当经农业部或者省、自治区、直辖市人民政府兽医行政管理部门批准。

第十条 三级、四级实验室从事某种高致病性动物病原微生物或者疑似高致病性动物病原微生物实验活动的，应当具备下列条件：

（一）取得农业部颁发的《高致病性动物病原微生物实验室资格证书》，并在有效期内；

（二）实验活动限于与动物病原微生物菌（毒）种、样本有关的研究、检测、诊断和菌（毒）种保藏等。

农业部对特定高致病性动物病原微生物或疑似高致病性动物病原微生物实验活动的实验单位有明确规定的，只能在规定的实验室进行。

第十一条 符合前条规定条件的，申请人应当向所在地省、自治区、直辖市人民政府兽医行政管理部门提出申请，并提交下列材料：

（一）高致病性动物病原微生物实验活动申请表一式两份；

（二）高致病性动物病原微生物实验室资格证书复印件；

（三）从事与高致病性动物病原微生物有关的科研项目的，还应当提供科研项目立项证明材料。

从事我国尚未发现或者已经宣布消灭的动物病原微生物有关实验活动的，或者从事国家规定的特定高致病性动物病原微生物病原分离和鉴定、活病毒培养、感染材料核酸提取、动物接种试验等有关实验活动的，省、自治区、直辖市人民政府兽医行政管理部门应当自收到申请之日起7日内，将初审意见和有关材料报送农业部。农业部自收到初审意见和有关材料之日起8日内作出是否批准的决定；不予批准的，及时通知申请人并说明理由。

从事前款规定以外的其他高致病性动物病原微生物或者疑似高致病性动物病原微生物实验活动的，省、自治区、直辖市人民政府兽医行政管理部门应当自收到申请之日起15日内作出是否批准的决定，并自批准之日起10日内报农业部备案；不予批准的，应当及时通知申请人并说明理由。

第十二条 实验室申报或者接受与高致病性动物病原微生物有关的科研项目前，应当向农业部申请审查，并提交以下材料：

（一）高致病性动物病原微生物科研项目生物安全审查表一式两份；

（二）科研项目建议书；

（三）科研项目研究中采取的生物安全措施。

农业部自收到申请之日起20日内作出是否同意的决定。

科研项目立项后，需要从事与高致病性动物病原微生物有关的实验活动的，应当按照本办法第十条、第十一条的规定，经农业部或者省、自治区、直辖市人民政府兽医行政管理部门批准。

第十三条 出入境检验检疫机构、动物防疫机构在实验室开展检测、诊断工作时，发现高致病性动物病原微生物或疑似高致病性动物病原微生物，需要进一步从事这类高致病性动物病原微生物病原分离和鉴定、活病毒培养、感染材料核酸提取、动物接种试验等相关实验活动的，应当按照本办法第十条、第十一条的规定，经农业部或者省、自治区、直辖市人民政府兽医行政管理部门批准。

第十四条 出入境检验检疫机构为了检验检疫工作的紧急需要，申请在实验室对高致病性动物病原微生物或疑似高致病性动物病原微生物开展病原分离和鉴定、活病毒培养、感染材料核酸提取、动物接种试验等进一步实验活动的，应当具备下列条件，并按照本办法第十一条的规定提出申请。

（一）实验目的仅限于检疫；

（二）实验活动符合法定检疫规程；

（三）取得农业部颁发的《高致病性动物病原微生物实验室资格证书》，并在有效期内。

农业部或者省、自治区、直辖市人民政府兽医行政管理部门自收到申请之时起2小时内作出是否批准的决定；不批准的，通知申请人并说明理由。2小时内未作出决定的，出入境检验检疫机构实验室可以从事相应的实验活动。

第十五条 实验室在实验活动期间，应当按照《病原微生物实验室生物安全管理条例》的规定，做好实验室感染控制、生物安全防护、病原微生物菌（毒）种保存和使用、安全操作、实验室排放的废水和废气以及其他废物处置等工作。

第十六条 实验室在实验活动结束后，应当及时将病原微生物菌（毒）种、样本就地销毁或者送交农业部指定的保藏机构保藏，并将实验活动结果以及工作情况向原批准部门报告。

第四章 运输审批

第十七条 运输高致病性动物病原微生物菌（毒）种或者样本的，应当经农业部或者省、自治区、直辖市人民政府兽医行政管理部门批准。

第十八条 运输高致病性动物病原微生物菌（毒）种或者样本的，应当具备下列条件：

（一）运输的高致病性动物病原微生物菌（毒）种或者样本仅限用于依法进行的动物疫病的研究、检测、诊断、菌（毒）种保藏和兽用生物制品的生产等活动；

（二）接收单位是研究、检测、诊断机构的，应当取得农业部颁发的《高致病性动物病原微生物实验室资格证书》，并取得农业部或者省、自治区、直辖市人民政府兽医行政管理部门颁发的从事高致病性动物病原微生物或者疑似高致病性动物病原微生物实验活动批准文件；接收单位是兽用生物制品研制和生产单位的，应当取得农业部颁发的生物制品批准文件；接收单位是菌（毒）种保藏机构的，应当取得农业部颁发的指定菌（毒）种保藏的文件；

（三）盛装高致病性动物病原微生物菌（毒）种或者样本的容器或者包装材料应当符合农业部制定的《高致病性动物病原微生物菌（毒）种或者样本运输包装规范》。

第十九条 符合前条规定条件的，申请人应当向出发地省、自治区、直辖市人民政府兽医行政管理部门提出申请，并提交以下材料：

（一）运输高致病性动物病原微生物菌（毒）种（样本）申请表一式两份;

（二）前条第二项规定的有关批准文件复印件；

（三）接收单位同意接收的证明材料，但送交菌（毒）种保藏的除外。

在省、自治区、直辖市人民政府行政区域内运输的，省、自治区、直辖市人民政府兽医行政管理部门应当对申请人提交的申请材料进行审查，符合条件的，即时批准，发给《高致病性动物病原微生物菌（毒）种、样本准运证书》；不予批准的，应当即时告知申请人。

需要跨省、自治区、直辖市运输或者运往国外的，由出发地省、自治区、直辖市人民政府兽医行政管理部门进行初审，并将初审意见和有关材料报送农业部。农业部应当对初审意见和有关材料进行审查，符合条件的，即时批准，发给《高致病性动物病原微生物菌（毒）种、样本准运证书》；不予批准的，应当即时告知申请人。

第二十条 申请人凭《高致病性动物病原微生物菌（毒）种、样本准运证书》运输高致病性动物病原微生物菌（毒）种或者样本；需要通过铁路、公路、民用航空等公共交通工具运输的，凭《高致病性动物病原微生物菌（毒）种、样本准运证书》办理承运手续；通过民航运输的，还需经过国务院民用航空主管部门批准。

第二十一条 出入境检验检疫机构在检疫过程中运输动物病原微生物样本的，由国务院出入境检验检疫部门批准，同时向农业部通报。

1. 国家卫生健康委员会：人间传染的高致病性病原微生物实验室和实验活动生物安全审批管理办法

第一章　总则

第一条　为加强实验室生物安全管理，规范高致病性病原微生物实验活动，依据《病原微生物实验室生物安全管理条例》，制定本办法。

第二条　本办法适用于三级、四级生物安全实验室从事与人体健康有关的高致病性病原微生物实验活动资格的审批，及其从事高致病性病原微生物或者疑似高致病性病原微生物实验活动的审批。

第三条　本办法所称高致病性病原微生物是指国家卫生计生委颁布的《人间传染的病原微生物名录》（以下简称《名录》）中公布的第一类、第二类病原微生物和按照第一类、第二类管理的病原微生物，以及其他未列入《名录》的与人体健康有关的高致病性病原微生物或者疑似高致病性病原微生物。

第四条　国家卫生计生委负责三级、四级生物安全实验室从事高致病性病原微生物实验活动资格的审批工作。

国家卫生计生委和省级卫生计生行政部门负责高致病性病原微生物或者疑似高致病性病原微生物实验活动的审批工作。

县级以上地方卫生计生行政部门负责本行政区域内高致病性病原微生物实验室及其实验活动的生物安全监督管理工作。

第二章　高致病性病原微生物实验室资格的审批

第五条　三级、四级生物安全实验室从事高致病性病原微生物实验活动，必须取得国家卫生计生委颁发的《高致病性病原微生物实验室资格证书》。

第六条　三级、四级生物安全实验室申请《高致病性病原微生物实验室资格证书》，应当具备以下条件：

（一）根据实验室所属法人机构的职能，合法从事与病原微生物菌（毒）种、样本有关的研究、教学、检测、诊断、保藏及生物制品生产等活动，并符合有关主管部门的相关规定；

（二）实验室的生物安全防护水平与所从事的病原微生物实验活动相适应，符合《名录》对生物安全防护水平的要求；

（三）工程质量依法验收合格；通过实验室国家认可，取得相应级别的生物安全实验室认可证书；

（四）实验室应当具备与所从事的实验活动相适应的实验设施、设备及防护措施；

（五）从事实验室活动的人员应当参加生物安全培训并取得上岗资格；

（六）应当明确实验室的职能、工作范围、工作内容和所从事的病原微生物种类；对所从事的病原微生物应当进行危害性评估，制订生物安全防护方案、实验方法及相应标准操作程序（SOP）、意外事故应急预案及感染监测方案等；

（七）应当建立持续有效的实验室生物安全管理体系及完善的管理制度。

第七条　申请《高致病性病原微生物实验室资格证书》，应当向省级卫生计生行政部门提交以下资料：

（一）高致病性病原微生物实验室资格申请表；

（二）实验室所属法人机构的法人资格证书；

（三）实验室认可证书；

（四）工程质量依法验收合格的相关证明材料；

（五）实验室人员名单，实验室人员取得的生物安全岗位培训证书及所在单位颁发的上岗证书；

（六）实验室职能报告（包括工作范围、工作内容等），拟从事实验活动的高致病性病原微生物名单及危害性评估报告、实验内容及相应标准操作程序（SOP）、生物安全防护方案、意外事故应急预案、暴露及暴露后监测和处理方案等；

（七）实验室的生物安全管理文件、实验室安全手册和其他相关文件；

（八）实验设施、设备清单；

（九）个体防护设备、用品清单；

（十）国家卫生计生委规定的其他相关资料。

第八条　高致病性病原微生物实验室资格审批程序：

（一）省级卫生计生行政部门应当对申请单位提交的申请材料及时审查，对申请材料不齐全或者不符合法定形式的，应当在5日内出具申请材料补正通知书；申请材料齐全或者符合法定形式的，应当在15个工作日内提出初审意见；并将初审意见和有关资料报国家卫生计生委；

（二）国家卫生计生委对申报资料进行审查后，应当组织专家进行生物安全评估和技术论证，并组织专家到现场进行评估和论证；专家评估和论证所需时限应当书面告知申请人；

（三）国家卫生计生委应当自收到专家评估论证意见之日起20个工作日内做出是否批准的决定。对于批准的，由国家卫生计生委颁发《高致病性病原微生物实验室资格证书》，并附批准该实验室从事的病原微生物名单和项目范围；对不予批准的，由国家卫生计生委书面通知申请者并说明理由。

第九条　取得《高致病性病原微生物实验室资格证书》的三级、四级生物安全实验室软件、硬件系统发生较大变化时，应当按照程序报国家卫生计生委进行评价和审批。

第十条　《高致病性病原微生物实验室资格证书》有效期5年。实验室需要继续从事高致病性病原微生物实验活动的，应当在有效期届满前6个月按照本办法的规定重新申请《高致病性病原微生物实验室资格证书》。

第三章　高致病性病原微生物实验活动的审批

第十一条　取得《高致病性病原微生物实验活动资格证书》的三级、四级生物安全实验室，需要从事某种高致病性病原微生物或者疑似高致病性病原微生物实验活动的，应当报省级以上卫生计生行政部门批准。

第十二条　实验室申请从事《名录》规定在四级生物安全实验室进行的实验活动或者申请从事该实验室病原微生物名单和项目范围外的实验活动的，由国家卫生计生委审批；申请从事该实验室病原微生物名单和项目范围内且在三级生物安全实验室进行的实验活动，由省级卫生计生行政部门审批，并报国家卫生计生委备案。

第十三条　为了预防、控制传染病，需要对我国尚未发现或者已经宣布消灭的病原微生物从事相关实验活动的，应当经国家卫生计生委批准，并在国家卫生计生委指定的实验室中进行。

拟从事未列入《名录》的高致病性病原微生物或者疑似高致病性病原微生物实验活动的实验室应当先进行危害性评估，提出实验室生物安全防护级别，并按照程序报国家卫生计生委审批。

第十四条　取得高致病性病原微生物实验室资格的三级、四级生物安全实验室，申请开展某种高致病性病原微生物或者疑似高致病性病原微生物实验活动，应当具备以下条件：

（一）实验活动是以依法从事检测检验、诊断、科学研究、教学、菌（毒）种保藏、生物制品生产等为目的；

（二）实验室的生物安全防护级别应当与其拟从事的实验活动相适应；

（三）实验室应当具备与所从事的实验活动相适应的人员、设备等；

（四）实验室应当根据《名录》，对拟从事实验活动的高致病性病原微生物或者疑似高致病性病原微生物的危害性进行评估，并制定切实可行的生物安全防护措施、意外事故应急预案及标准操作程序。

第十五条　国家对从事特定的高致病性病原微生物或者疑似高致病性病原微生物实验活动的单位有明确规定的，由国家指定的实验室开展有关实验活动。

第十六条　申请开展高致病性病原微生物或者疑似高致病性病原微生物实验活动，应当向省级卫生计生行政部门提交以下资料：

（一）高致病性病原微生物实验活动申请表（一式二份）；

（二）《高致病性病原微生物实验室资格证书》；

（三）实验室所属法人机构生物安全委员会审查意见；

（四）实验活动的主要内容和技术方法报告；

（五）实验室人员名单，实验室人员取得的生物安全岗位培训证书及所在单位颁发的上岗证书；

（六）省级以上卫生计生行政部门规定的其他有关资料。

第十七条　高致病性病原微生物实验活动审批程序：

（一）按照本办法第十二条、第十三条的规定，需要由国家卫生计生委批准的，省级卫生计生行政部门应当在受理申请材料之日起5个工作日内进行初审，提出初审意见；并将初审意见和有关资料报国家卫生计生委；

（二）国家卫生计生委对申报材料进行审查，并组织专家委员会对申报材料进行评估和论证，提出评估论证意见；专家评估和论证所需时限应当书面告知申请人；

（三）国家卫生计生委自收到专家评估论证意见之日起10个工作日内，做出是否批准的决定。对于批准实验活动的，由国家卫生计生委颁发《高致病性病原微生物实验活动批准证书》。对不予批准的，由国家卫生计生委书面通知申请者并说明理由。对于批准的实验活动，国家卫生计生委应当告知实验室所在地省级卫生计生行政部门进行监督检查；

（四）按照本办法第十二条的规定，需要由省级卫生计生行政部门批准的，其申报和审批程序由省级卫生计生行政部门参照本办法的有关要求制定。需要向国家卫生计生委备案的实验活动，应当在批准之日起10个工作日内报国家卫生计生委备案；

（五）在突发公共卫生事件应急状态下，省级以上卫生计生行政部门可以根据疾病预防控制和医疗救治工作的紧急需要，简化审批程序，临时指定合格的实验室开展相应的实验活动。

省级以上卫生计生行政部门应当为申请人通过电报、电传、传真、电子数据交换和电子邮件等方式提出申请提供方便。

第十八条　实验室申报或者接受与高致病性病原微生物有关的科研项目，应当符合科研需要和生物安全要求，具有相应的生物安全防护水平。按照本办法第十二条和第十三条规定，科研项目涉及到应由国家卫生计生委审批的实验活动的，承担单位应当将立项结果报国家卫生计生委；科研项目涉及到应由省级卫生计生行政部门审批的实验活动的，承担单位应当将立项结果报省级卫生计生行政部门。

科研项目立项后所从事的实验活动，应当按照本办法第十四条、第十五条、第十六条的规定报批。

第十九条　出入境检验检疫机构、医疗卫生机构等在实验室开展检测、诊断工作时，发现高致病性病原微生物或者疑似高致病性病原微生物，需要进一步从事相关实验活动的，应当依照本办法的规定报批或者转运至具备资格的实验室开展实验活动。

第二十条　出入境检验检疫机构为了检疫工作的紧急需要，申请在实验室对高致病性病原微生物或者疑似高致病性病原微生物开展进一步实验活动的，应当获得所在地省级出入境检验检疫主管部门的同意，并按照本办法第十四条、第十五条、第十六条的规定向所在地省级卫生计生行政部门提出申请。省级卫生计生行政部门应当自收到申请之时起2小时内做出是否批准的决定。省级卫生计生行政部门在做出批准决定的同时，应当通知实验室所在地卫生计生行政部门进行生物安全监督。在规定的时间内未做出决定的，出入境检验检疫机构实验室可以从事相应的实验活动。

第二十一条　需要在动物体上从事高致病性病原微生物相关实验活动的，应当在符合动物实验室生物安全国家标准的生物安全三级以上实验室进行，并按照本办法的规定报批。

第四章　监督管理

第二十二条　实验室的设立单位及其主管部门应当按照《病原微生物实验室生物安全管理条例》的有关规定，加强对高致病性病原微生物实验室的生物安全防护和实验活动的管理。

第二十三条　高致病性病原微生物实验室应当在明显位置标示生物危险标识和生物安全实验室级别标志。

高致病性病原微生物实验室应当制定科学、严格的管理制度并认真贯彻执行。

第二十四条　高致病性病原微生物实验室应当每年定期对工作人员进行培训，并对实验室工作人员进行健康监测。

第二十五条　高致病性病原微生物实验室应当建立完备的实验记录和档案，做好实验室感染控制工作，制定实验室感染应急处置预案。

第二十六条　高致病性病原微生物实验室从事的高致病性病原微生物实验活动结束后，应当及时将病原微生物菌（毒）种和样本就地销毁或者送交保藏机构保管，并及时将实验活动结果以及工作情况向原批准部门报告。

第二十七条　高致病性病原微生物实验室使用新技术、新方法从事高致病性病原微生物相关实验活动的，应当符合防止高致病性病原微生物扩散、保证生物安全和操作者人身安全的要求。

高致病性病原微生物实验室所属法人机构生物安全委员会应对其进行评价，并报国家病原微生物实验室生物安全专家委员会论证；经论证可行的，方可使用。

第二十八条　各级卫生计生行政部门应当按照《病原微生物实验室生物安全管理条例》的规定，对高致病性病原微生物实验室及其实验活动进行监督检查。

第二十九条　对于违反本办法规定的行为，依照《病原微生物实验室生物安全管理条例》第五十六条、第五十七条、第五十八条、第五十九条、第六十条、第六十三条的有关规定予以处罚。

1. 国务院办公厅：国务院办公厅关于印发“十四五”国民健康规划的通知

国办发〔2022〕11号

完善审批程序，加强实验室生物安全管理，强化运行评估和监管。完善高级别病原微生物实验室运行评价和保障体系，完善国家病原微生物菌（毒）种和实验细胞等可培养物保藏体系。

1. 农业农村部：动物病原微生物菌（毒）种保藏管理办法

第一条 为了加强动物病原微生物菌（毒）种和样本保藏管理，依据《中华人民共和国动物防疫法》、《病原微生物实验室生物安全管理条例》和《兽药管理条例》等法律法规，制定本办法。

第二条 本办法适用于中华人民共和国境内菌（毒）种和样本的保藏活动及其监督管理。

第三条 本办法所称菌（毒）种，是指具有保藏价值的动物细菌、真菌、放线菌、衣原体、支原体、立克次氏体、螺旋体、病毒等微生物。

本办法所称样本，是指人工采集的、经鉴定具有保藏价值的含有动物病原微生物的体液、组织、排泄物、分泌物、污染物等物质。

本办法所称保藏机构，是指承担菌（毒）种和样本保藏任务，并向合法从事动物病原微生物相关活动的实验室或者兽用生物制品企业提供菌（毒）种或者样本的单位。

菌（毒）种和样本的分类按照《动物病原微生物分类名录》的规定执行。

第四条 农业农村部主管全国菌（毒）种和样本保藏管理工作。

县级以上地方人民政府畜牧兽医主管部门负责本行政区域内的菌（毒）种和样本保藏监督管理工作。

第五条 国家对实验活动用菌（毒）种和样本实行集中保藏，保藏机构以外的任何单位和个人不得保藏菌（毒）种或者样本。

第二章 保藏机构

第六条 保藏机构分为国家级保藏中心和省级保藏中心。保藏机构由农业农村部指定。

保藏机构保藏的菌（毒）种和样本的种类由农业农村部核定。

第七条 保藏机构应当具备以下条件：

（一）符合国家关于保藏机构设立的整体布局和实际需要；

（二）有满足菌（毒）种和样本保藏需要的设施设备；保藏高致病性动物病原微生物菌（毒）种或者样本的，应当具有相应级别的高等级生物安全实验室，并依法取得《高致病性动物病原微生物实验室资格证书》；

（三）有满足保藏工作要求的工作人员；

（四）有完善的菌（毒）种和样本保管制度、安全保卫制度；

（五）有满足保藏活动需要的经费。

第八条 保藏机构的职责：

（一）负责菌（毒）种和样本的收集、筛选、分析、鉴定和保藏；

（二）开展菌（毒）种和样本的分类与保藏新方法、新技术研究；

（三）建立菌（毒）种和样本数据库；

（四）向合法从事动物病原微生物实验活动的实验室或者兽用生物制品生产企业提供菌（毒）种或者样本。

第三章 菌（毒）种和样本的收集

第九条 从事动物疫情监测、疫病诊断、检验检疫和疫病研究等活动的单位和个人，应当及时将研究、教学、检测、诊断等实验活动中获得的具有保藏价值的菌（毒）种和样本，送交保藏机构鉴定和保藏，并提交菌（毒）种和样本的背景资料。

保藏机构可以向国内有关单位和个人索取需要保藏的菌（毒）种和样本。

第十条 保藏机构应当向提供菌（毒）种和样本的单位和个人出具接收证明。

第十一条 保藏机构应当在每年年底前将保藏的菌（毒）种和样本的种类、数量报农业农村部。

第四章 菌（毒）种和样本的保藏、供应

第十二条 保藏机构应当设专库保藏一、二类菌（毒）种和样本，设专柜保藏三、四类菌（毒）种和样本。

保藏机构保藏的菌（毒）种和样本应当分类存放，实行双人双锁管理。

第十三条 保藏机构应当建立完善的技术资料档案，详细记录所保藏的菌（毒）种和样本的名称、编号、数量、来源、病原微生物类别、主要特性、保存方法等情况。

技术资料档案应当永久保存。

第十四条 保藏机构应当对保藏的菌（毒）种按时鉴定、复壮，妥善保藏，避免失活。

保藏机构对保藏的菌（毒）种开展鉴定、复壮的，应当按照规定在相应级别的生物安全实验室进行。

第十五条 保藏机构应当制定实验室安全事故处理应急预案。发生保藏的菌（毒）种或者样本被盗、被抢、丢失、泄漏和实验室人员感染的，应当按照《病原微生物实验室生物安全管理条例》的规定及时报告、启动预案，并采取相应的处理措施。

第十六条 实验室和兽用生物制品生产企业需要使用菌（毒）种或者样本的，应当向保藏机构提出申请。

第十七条 保藏机构应当按照以下规定提供菌（毒）种或者样本：

（一）提供高致病性动物病原微生物菌（毒）种或者样本的，查验从事高致病性动物病原微生物相关实验活动的批准文件；

（二）提供兽用生物制品生产和检验用菌（毒）种或者样本的，查验兽药生产批准文号文件；

（三）提供三、四类菌（毒）种或者样本的，查验实验室所在单位出具的证明。

保藏机构应当留存前款规定的证明文件的原件或者复印件。

第十八条 保藏机构提供菌（毒）种或者样本时，应当进行登记，详细记录所提供的菌（毒）种或者样本的名称、数量、时间以及发放人、领取人、使用单位名称等。

第十九条 保藏机构应当对具有知识产权的菌（毒）种承担相应的保密责任。

保藏机构提供具有知识产权的菌（毒）种或者样本的，应当经原提供者或者持有人的书面同意。

第二十条 保藏机构提供的菌（毒）种或者样本应当附有标签，标明菌（毒）种名称、编号、移植和冻干日期等。

第二十一条 保藏机构保藏菌（毒）种或者样本所需费用由同级财政在单位预算中予以保障。

第五章 菌（毒）种和样本的销毁

第二十二条 有下列情形之一的，保藏机构应当组织专家论证，提出销毁菌（毒）种或者样本的建议：

（一）国家规定应当销毁的；

（二）有证据表明已丧失生物活性或者被污染，已不适于继续使用的；

（三）无继续保藏价值的。

第二十三条 保藏机构销毁一、二类菌（毒）种和样本的，应当经农业农村部批准；销毁三、四类菌（毒）种和样本的，应当经保藏机构负责人批准，并报农业农村部备案。

保藏机构销毁菌（毒）种和样本的，应当在实施销毁30日前书面告知原提供者。

第二十四条 保藏机构销毁菌（毒）种和样本的，应当制定销毁方案，注明销毁的原因、品种、数量，以及销毁方式方法、时间、地点、实施人和监督人等。

第二十五条 保藏机构销毁菌（毒）种和样本时，应当使用可靠的销毁设施和销毁方法，必要时应当组织开展灭活效果验证和风险评估。

第二十六条 保藏机构销毁菌（毒）种和样本的，应当做好销毁记录，经销毁实施人、监督人签字后存档，并将销毁情况报农业农村部。

第二十七条 实验室在相关实验活动结束后，应当按照规定及时将菌（毒）种和样本就地销毁或者送交保藏机构保管。

第六章 菌（毒）种和样本的对外交流

第二十八条 国家对菌（毒）种和样本对外交流实行认定审批制度。

第二十九条 从国外引进和向国外提供菌（毒）种或者样本的，应当报农业农村部批准。

第三十条 从国外引进菌（毒）种或者样本的单位，应当在引进菌（毒）种或者样本后6个月内，将备份及其背景资料，送交保藏机构。

引进单位应当在相关活动结束后，及时将菌（毒）种和样本就地销毁。

第三十一条 出口《生物两用品及相关设备和技术出口管制清单》所列的菌（毒）种或者样本的，还应当按照《生物两用品及相关设备和技术出口管制条例》的规定取得生物两用品及相关设备和技术出口许可证件。

第七章 罚则

第三十二条 违反本办法规定，保藏或者提供菌（毒）种或者样本的，由县级以上地方人民政府畜牧兽医主管部门责令其将菌（毒）种或者样本销毁或者送交保藏机构；拒不销毁或者送交的，对单位处一万元以上三万元以下罚款，对个人处五百元以上一千元以下罚款。

第三十三条 违反本办法规定，未及时向保藏机构提供菌（毒）种或者样本的，由县级以上地方人民政府畜牧兽医主管部门责令改正；拒不改正的，对单位处一万元以上三万元以下罚款，对个人处五百元以上一千元以下罚款。

第三十四条 违反本办法规定，未经农业农村部批准，从国外引进或者向国外提供菌（毒）种或者样本的，由县级以上地方人民政府畜牧兽医主管部门责令其将菌（毒）种或者样本销毁或者送交保藏机构，并对单位处一万元以上三万元以下罚款，对个人处五百元以上一千元以下罚款。

第三十五条 保藏机构违反本办法规定的，由农业农村部责令限期改正，并给予警告；造成严重后果的，由其所在单位或者其上级主管部门对主要负责人、直接负责的主管人员和其他直接责任人员依法予以处理。

第八章 附则

第三十六条 本办法自2009年1月1日起施行。1980年11月25日农业部发布的《兽医微生物菌种保藏管理试行办法》（农〔牧〕字第181号）同时废止。

1. 国务院办公厅：国务院办公厅关于改革完善医疗卫生行业综合监管制度的指导意见

国办发〔2018〕63号

依法加强对环境保护、食品安全、职业卫生、精神卫生、放射卫生、传染病防治、实验室生物安全、公共场所卫生、饮用水卫生、学校卫生等公共卫生服务的监管。

1. 国务院办公厅：国务院办公厅关于加强传染病防治人员安全防护的意见

国务院办公厅关于加强传染病防治人员安全防护的意见

"加强实验室生物安全条件建设和管理：建立和完善生物安全实验室网络，提升高致病性病原微生物实验室检测能力和防护水平，降低标本转运、保藏、检测等环节的感染风险。科学规划和布局高等级生物安全实验室，每个省份应当设有生物安全三级实验室，推进国家生物安全四级实验室建设。发展改革、财政等部门和地方要做好高等级生物安全实验室建设的投资安排。实验室建设要依法开展环境影响评价。各地和有关单位要加强生物安全三级实验室的使用管理和维护，确保其有效运转、发挥作用。进一步加强实验室装备建设，逐步使省、市、县级疾病预防控制机构仪器配备达到《疾病预防控制中心建设标准》规定要求。切实落实重大科研基础设施和大型科研仪器向社会开放的规定，建立高等级生物安全实验室共享机制，满足传染病防控、医疗、科研等工作需要。卫生计生、农业、质检、林业等部门要完善实验室生物安全、菌毒种保藏、储存运输相关规范和操作流程，制定实验室生物安全事故应对和处置预案，完善应对准备和相关设备、设施、技术储备。要健全生物安全实验室管理体系，加强对实验室生物安全防护的质量控制和全过程监管，做好样本采集、运输、保存、检测等环节的人员防护，明确行政管理和技术责任人，有效预防实验室生物安全事故发生。

做好医疗废物处置、患者遗体处理及相关人员防护

严格落实《医疗废物管理条例》规定，切实做好医疗废物集中无害化处置，落实医疗废物收集、运送、贮存、处置的全过程管理。各地要加强医疗废物集中处置单位建设，确保医疗废物出口通畅。医疗卫生机构和医疗废物集中处置单位要建立健全医疗废物管理责任制，严格执行转移联单制度，防止医疗废物流失。禁止任何单位和个人非法转让、买卖医疗废物。按规定对传染病患者遗体进行卫生处理，对死者生前居住场所进行消毒，对确诊或疑似传染病患者尸体解剖查验过程中产生的医疗废物进行规范处理，并做好工作人员的安全防护。"

1. 国务院办公厅：国务院办公厅关于印发促进生物产业加快发展若干政策的通知

国办发〔2009〕45号

加强生物安全管理。认真履行生物安全有关国际公约，依据有关法律法规健全生物安全特别是转基因生物安全技术标准、安全评价、检测监测和监督管理体系，提高安全监管能力。加强防范外来有害生物入侵的防御体系建设，完善进境生物安全防范体系，防范转基因生物、微生物菌剂非法越境转移和无意越境转移。依法限制或禁止影响国家安全或公共利益的生物技术和产品进出口。建立健全生物安全风险分析和信息交换机制，强化风险预警和应急反应机制，提高防范与应对外来有害生物入侵、生物恐怖袭击的能力。加强实验室生物安全监督管理，健全实验室生物安全体系，保护实验室工作人员和公众安全。

1. 国家卫生健康委员会：人间传染的病原微生物菌（毒）种保藏机构管理办法

　　第一条　为加强人间传染的病原微生物菌（毒）种（以下称菌（毒）种）保藏机构的管理，保护和合理利用我国菌（毒）种或样本资源，防止菌（毒）种或样本在保藏和使用过程中发生实验室感染或者引起传染病传播，依据《中华人民共和国传染病防治法》、《病原微生物实验室生物安全管理条例》（以下称《条例》）的规定制定本办法。

　　第二条　卫生部主管全国人间传染的菌（毒）种保藏机构（以下称保藏机构）的监督管理工作。

　　县级以上人民政府卫生行政部门负责本行政区域内保藏机构的监督管理工作。

　　第三条　本办法所称的菌（毒）种是指可培养的，人间传染的真菌、放线菌、细菌、立克次体、螺旋体、支原体、衣原体、病毒等具有保存价值的，经过保藏机构鉴定、分类并给予固定编号的微生物。

　　本办法所称的病原微生物样本（以下称样本）是指含有病原微生物的、具有保存价值的人和动物体液、组织、排泄物等物质，以及食物和环境样本等。

　　可导致人类传染病的寄生虫不同感染时期的虫体、虫卵或样本按照本办法进行管理。

　　编码产物或其衍生物对人体有直接或潜在危害的基因（或其片段）参照本办法进行管理。

　　菌（毒）种的分类按照《人间传染的病原微生物名录》（以下简称《名录》）的规定执行。

　　菌（毒）种或样本的保藏是指保藏机构依法以适当的方式收集、检定、编目、储存菌（毒）种或样本，维持其活性和生物学特性，并向合法从事病原微生物相关实验活动的单位提供菌（毒）种或样本的活动。

　　保藏机构是指由卫生部指定的，按照规定接收、检定、集中储存与管理菌（毒）种或样本，并能向合法从事病原微生物实验活动的单位提供菌（毒）种或样本的非营利性机构。

　　第四条　保藏机构以外的机构和个人不得擅自保藏菌（毒）种或样本。

　　必要时，卫生部可以根据疾病控制和科研、教学、生产的需要，指定特定机构从事保藏活动。

　　第五条　国家病原微生物实验室生物安全专家委员会卫生专业委员会负责保藏机构的生物安全评估和技术咨询、论证等工作。

　　第六条　菌( 毒)种或样本有关保密资料、信息的管理和使用必须严格遵守国家保密工作的有关法律、法规和规定。信息及数据的相关主管部门负责确定菌( 毒)种或样本有关资料和信息的密级、保密范围、保密期限、管理责任和解密。各保藏机构应当根据菌( 毒)种信息及数据所定密级和保密范围制定相应的保密制度，履行保密责任。

　　未经批准，任何组织和个人不得以任何形式泄漏涉密菌( 毒)种或样本有关的资料和信息，不得使用个人计算机、移动储存介质储存涉密菌( 毒)种或样本有关的资料和信息。

第二章　保藏机构的职责

　　第七条　保藏机构分为菌（毒）种保藏中心和保藏专业实验室。菌（毒）种保藏中心分为国家级和省级两级。

　　保藏机构的设立及其保藏范围应当根据国家在传染病预防控制、医疗、检验检疫、科研、教学、生产等方面工作的需要，兼顾各地实际情况，统一规划、整体布局。

　　国家级菌（毒）种保藏中心和保藏专业实验室根据工作需要设立。省级菌（毒）种保藏中心根据工作需要设立，原则上各省、自治区、直辖市只设立一个。

　　第八条　国家级菌（毒）种保藏中心的职责为：

　　（一）负责菌（毒）种或样本的收集、选择、鉴定、复核、保藏、供应和依法进行对外交流；

　　（二）出具国家标准菌（毒）株证明；

　　（三）从国际菌（毒）种保藏机构引进标准或参考菌（毒）种，供应国内相关单位使用；

　　（四）开展菌（毒）种或样本分类、保藏新方法、新技术的研究和应用；

　　（五）负责收集和提供菌（毒）种或样本的信息，编制菌（毒）种或样本目录和数据库；

　　（六）组织全国学术交流和培训；

　　（七）对保藏专业实验室和省级菌（毒）种保藏中心进行业务指导。

　　第九条　省级菌（毒）种保藏中心的职责：

　　（一）负责本行政区域内菌（毒）种或样本的收集、选择、鉴定、分类、保藏、供应和依法进行对外交流；

　　（二）向国家级保藏机构提供国家级保藏机构所需的菌（毒）种或样本；

　　（三）从国家或者国际菌（毒）种保藏机构引进标准或参考菌（毒）种，供应辖区内相关单位使用；

　　（四）开展菌（毒）种或样本分类、保藏新方法、新技术的研究和应用；

　　（五）负责收集和提供本省（自治区、直辖市）菌（毒）种或样本的各种信息，编制地方菌（毒）种或样本目录和数据库。

　　第十条　保藏专业实验室的职责：

　　（一）负责专业菌（毒）种或样本的收集、选择、鉴定、复核、保藏、供应和依法进行对外交流；

　　（二）开展菌（毒）种或样本分类、保藏新方法、新技术的研究和应用；

　　（三）负责提供专业菌（毒）种或样本的各种信息，建立菌（毒）种或样本数据库；

　　（四）向国家级和所属行政区域内省级保藏中心提供菌（毒）种代表株。

　　第十一条　下列菌（毒）种或样本必须由国家级保藏中心或专业实验室进行保藏：

　　（一）我国境内未曾发现的高致病性病原微生物菌（毒）种或样本和已经消灭的病原微生物菌（毒）种或样本；

　　（二）《名录》规定的第一类病原微生物菌（毒）种或样本；

　　（三）卫生部规定的其他菌（毒）种或样本。

第三章　保藏机构的指定

　　第十二条　保藏机构及其保藏范围由卫生部组织专家评估论证后指定，并由卫生部颁发《人间传染的病原微生物菌（毒）种保藏机构证书》。

　　第十三条　申请保藏机构应当具备以下条件：

　　（一）符合国家关于保藏机构设立的整体布局（规划）和实际需要；

　　（二）依法从事涉及菌（毒）种或样本实验活动，并符合有关主管部门的相关规定；

　　（三）符合卫生部公布的《人间传染的病原微生物菌（毒）种保藏机构设置技术规范》的要求，具备与所从事的保藏工作相适应的保藏条件；

　　（四）生物安全防护水平与所保藏的病原微生物相适应，符合《名录》对生物安全防护水平的要求。高致病性菌（毒）种保藏机构还必须具备获得依法开展实验活动资格的相应级别的高等级生物安全实验室；

　　（五）工作人员具备与拟从事保藏活动相适应的能力；

　　（六）明确保藏机构的职能、工作范围、工作内容和所保藏的病原微生物种类。在对所保藏的病原微生物进行风险评估的基础上，制订可靠、完善的生物安全防护方案、相应标准操作程序、意外事故应急预案及感染监测方案等；

　　（七）建立持续有效的保藏机构实验室生物安全管理体系及完善的管理制度；

　　（八）具备开展保藏活动所需的经费支持。

　　第十四条　拟申请保藏机构的法人单位应当向所在地省、自治区、直辖市人民政府卫生行政部门提交下列资料：

　　（一）《人间传染的病原微生物菌（毒）种保藏机构申请表》；

　　（二）保藏机构所属法人机构的法人资格证书（复印件）；

　　（三）保藏机构生物安全实验室的相关批准或者证明文件（复印件）；

　　（四）保藏工作的内容、范围，拟保藏菌（毒）种及样本的清单；

　　（五）保藏机构的组织结构、管理职责、硬件条件、基本建设条件等文件，并提供设施、设备、用品清单；

　　（六）生物安全管理文件、生物安全手册、风险评估报告、相应标准操作程序、生物安全防护方案、意外事故和安全保卫应急预案、暴露及暴露后监测和处理方案等；

　　（七）保藏机构人员名单、生物安全培训证明及所在单位颁发的上岗证书；

　　（八）卫生部规定的其他相关资料。

　　省、自治区、直辖市人民政府卫生行政部门收到材料后，在15个工作日内进行审核，审核同意的报卫生部。卫生部在收到省、自治区、直辖市人民政府卫生行政部门报告后60个工作日内组织专家进行评估和论证，对于符合本办法第十三条所列条件的，颁发《人间传染的病原微生物菌（毒）种保藏机构证书》。

　　第十五条　取得《人间传染的病原微生物菌（毒）种保藏机构证书》的保藏机构发生以下变化时，应当及时向省、自治区、直辖市人民政府卫生行政部门报告，省、自治区、直辖市人民政府卫生行政部门经核查后报卫生部：

　　（一）实验室生物安全级别发生变化；

　　（二）实验室增加高致病性菌（毒）种或样本保藏内容；

　　（三）保藏场所和空间发生变化；

　　（四）实验室存在严重安全隐患、发生生物安全事故；

　　（五）管理体系文件换版或者进行较大修订；

　　（六）保藏机构应报告的其他重大事项。

　　第十六条　《人间传染的病原微生物菌（毒）种保藏机构证书》有效期5年。保藏机构需要继续从事保藏工作的，应当在有效期届满前6个月按照本办法的规定重新申请《人间传染的病原微生物菌（毒）种保藏机构证书》。

第四章　保藏活动

　　第十七条　各实验室应当将在研究、教学、检测、诊断、生产等实验活动中获得的有保存价值的各类菌（毒）株或样本送交保藏机构进行鉴定和保藏。保藏机构对送交的菌（毒）株或样本，应当予以登记，并出具接收证明。

　　国家级保藏中心、专业实验室和省级保藏中心应当定期向卫生部指定的机构申报保藏入库菌（毒）种目录。

　　国家级保藏中心可根据需要选择收藏省级保藏中心保藏的有价值的菌（毒）种。

　　第十八条　保藏机构有权向有关单位收集和索取所需要保藏的菌（毒）种，相关单位应当无偿提供。

　　第十九条　保藏机构对专用和专利菌（毒）种要承担相应的保密责任，依法保护知识产权和物权。

　　样本等不可再生资源所有权属于提交保藏的单位，其他单位需要使用，必须征得所有权单位的书面同意。根据工作需要，卫生部和省、自治区、直辖市人民政府卫生行政部门依据各自权限可以调配使用。

　　第二十条　申请使用菌（毒）种或样本的实验室，应当向保藏机构提供从事病原微生物相关实验活动的批准或证明文件。保藏机构应当核查登记后无偿提供菌（毒）种或样本。

　　非保藏机构实验室在从事病原微生物相关实验活动结束后，应当在6个月内将菌（毒）种或样本就地销毁或者送交保藏机构保藏。

　　医疗卫生、出入境检验检疫、教学和科研机构按规定从事临床诊疗、疾病控制、检疫检验、教学和科研等工作，在确保安全的基础上，可以保管其工作中经常使用的菌（毒）种或样本，其保管的菌（毒）种或样本名单应当报当地卫生行政部门备案。但涉及高致病性病原微生物及行政部门有特殊管理规定的菌（毒）种除外。

　　第二十一条　实验室从事实验活动，使用涉及本办法第十一条规定的菌（毒）种或样本，应当经卫生部批准；使用其他高致病性菌（毒）种或样本，应当经省级人民政府卫生行政部门批准；使用第三、四类菌（毒）种或样本，应当经实验室所在法人机构批准。

　　第二十二条　保藏机构储存、提供菌（毒）种和样本，不得收取任何费用。

　　第二十三条　保藏机构保藏的菌（毒）种或样本符合下列条件之一的可以销毁：

　　（一）国家规定必须销毁的；

　　（二）有证据表明保藏物已丧失生物活性或被污染已不适于继续使用的；

　　（三）保藏机构认为无继续保存价值且经送保藏单位同意的。

　　销毁的菌（毒）种或样本属于本办法第十一条规定的应当经卫生部批准；销毁其他高致病性菌（毒）种或样本，应当经省级人民政府卫生行政部门批准；销毁第三、四类菌（毒）种或样本的，应当经保藏机构负责人批准。

　　第二十四条　销毁高致病性病原微生物菌（毒）种或样本必须采用安全可靠的方法，并应当对所用方法进行可靠性验证。

　　销毁应当在与拟销毁菌（毒）种相适应的生物安全防护水平的实验室内进行，由两人共同操作，并应当对销毁过程进行严格监督。

　　销毁后应当作为医疗废物送交具有资质的医疗废物集中处置单位处置。

　　销毁的全过程应当有详细记录，相关记录保存不得少于20年。

　　第二十五条　保藏机构应当制定严格的安全保管制度，做好菌（毒）种或样本的出入库、储存和销毁等原始记录，建立档案制度，并指定专人负责。所有档案保存不得少于20年。

　　保藏机构对保藏的菌（毒）种或样本应当设专库储存。建立严格的菌（毒）种库人员管理制度，保（监）管人应当为本单位正式员工并不少于2人。

　　保藏环境和设施应当符合有关规范，具有防盗设施并向公安机关备案。保藏机构应当制定应急处置预案，并具备相关的应急设施设备，对储存库应当实行24小时监控。

　　第二十六条　对从事菌（毒）种或样本实验活动的专业人员，保藏机构应当按照国家规定采取有效的安全防护和医疗保障措施。

　　第二十七条　菌（毒）种或样本的国际交流应当符合本办法第十九条的规定，并参照《中华人民共和国生物两用品及相关设备和技术出口管制条例》、《出口管制清单》、《卫生部和国家质检总局关于加强医用特殊物品出入境管理卫生检疫的通知》等规定办理出入境手续。

第五章　监督管理与处罚

　　第二十八条　卫生部主管保藏机构生物安全监督工作。地方人民政府卫生行政部门应当按照属地化管理的原则对所辖区域内的保藏机构依法进行监督管理。保藏机构的设立单位及上级主管部门应当加强对保藏机构的建设及监督管理，建立明确的责任制和责任追究制度，确保实验室生物安全。

　　第二十九条　保藏机构应当加强自身管理工作，完善并执行下列要求：

　　（一）主管领导负责菌（毒）种或样本保藏工作；

　　（二）建立菌（毒）种或样本安全保管、使用和销毁制度，标准操作程序和监督保障体系；

　　（三）建立菌（毒）种或样本的出入库记录、相关生物学和鉴定、复核等信息档案；

　　（四）必须保持与其所保藏菌（毒）种或样本危害程度相适应的生物安全防护和储存条件的工作状态；

　　（五）工作人员必须经过生物安全和专业知识培训，考核合格后上岗；

　　（六）建立相关人员健康监测制度，制定保藏机构相关人员感染应急处置预案，并向实验活动批准机构备案。

　　第三十条　保藏机构每年年底应向卫生部报送所保藏的高致病性菌（毒）种或样本的种类、数量、使用、发放及变化等情况。

　　第三十一条　保藏机构在保藏过程中发生菌（毒）种或样本被盗、被抢、丢失、泄露以及实验室感染时，应当按照《条例》第十七条、第四十二条、第四十三条、第四十四条、第四十五条、第四十六条、第四十七条、第四十八条规定及时报告和处理，做好感染控制工作。

　　第三十二条　保藏机构未依照规定储存实验室送交的菌（毒）种和样本，或者未依照规定提供菌（毒）种和样本的，按照《条例》第六十八条规定，由卫生部责令限期改正，收回违法提供的菌（毒）种和样本，并给予警告；造成传染病传播、流行或者其他严重后果的，由其所在单位或者其上级主管部门对主要负责人、直接负责的主管人员和其他直接责任人员，依法予以处理；构成犯罪的，依法追究刑事责任。

第六章　附　　则

　　第三十三条　军队菌（毒）种保藏机构的管理由中国人民解放军主管部门负责。

　　第三十四条　本办法施行前设立的菌（毒）种保藏机构，应当自本办法施行之日起2年内，依照本办法申请《人间传染的病原微生物菌（毒）种保藏机构证书》。

　　第三十五条　本办法自2009年10月1日起施行。

1. 国务院办公厅：国务院办公厅关于转发发展改革委生物产业发展 “十一五” 规划的通知

国办发〔2007〕23号

加快制定生物安全法。建立病原微生物实验室生物安全监测与评价体系，建立我国统一的、与国际接轨的国家生物产业认证认可体系。

1. 国务院办公厅：国务院办公厅关于印发国家卫生和计划生育委员会主要职责内设机构和人员编制规定的通知

国办发〔2013〕50号

（十三）科技教育司。

承担医药卫生实验室生物安全的监督管理工作

1. 国务院：关于印发卫生事业发展“十二五”规划的通知

国发〔2012〕57号

加强实验室生物安全能力建设。

1. 国务院办公厅：国务院办公厅关于转发发展改革委等部门疫苗供应体系建设规划的通知

国办发〔2011〕62号

加强病原学和免疫学应用基础技术研究，加快疫苗相关实验室生物安全能力建设。

1. 国家卫生健康委员会：可感染人类的高致病性病原微生物菌（毒）种或样本运输管理规定

第一条　为加强可感染人类的高致病性病原微生物菌（毒）种或样本运输的管理，保障人体健康和公共卫生，依据《中华人民共和国传染病防治法》、《病原微生物实验室生物安全管理条例》等法律、行政法规的规定，制定本规定。

第二条　本规定所称可感染人类的高致病性病原微生物菌（毒）种或样本是指在《人间传染的病原微生物名录》中规定的第一类、第二类病原微生物菌（毒）种或样本。

第三条　本规定适用于可感染人类的高致病性病原微生物菌（毒）种或样本的运输管理工作。

《人间传染的病原微生物名录》中第三类病原微生物运输包装分类为A类的病原微生物菌（毒）种或样本，以及疑似高致病性病原微生物菌（毒）种或样本，按照本规定进行运输管理。

第四条　运输第三条规定的菌（毒）种或样本（以下统称高致病性病原微生物菌（毒）种或样本），应当经省级以上卫生行政部门批准。未经批准，不得运输。

第五条　从事疾病预防控制、医疗、教学、科研、菌（毒）种保藏以及生物制品生产的单位，因工作需要，可以申请运输高致病性病原微生物菌（毒）种或样本。

第六条　申请运输高致病性病原微生物菌（毒）种或样本的单位（以下简称申请单位），在运输前应当向省级卫生行政部门提出申请，并提交以下申请材料（原件一份，复印件三份）：

（一）可感染人类的高致病性病原微生物菌（毒）种或样本运输申请表；

（二）法人资格证明材料（复印件）；

（三）接收高致病性病原微生物菌（毒）种或样本的单位（以下简称接收单位）同意接收的证明文件；

（四）本规定第七条第（二）、（三）项所要求的证明文件（复印件）；

（五）容器或包装材料的批准文号、合格证书（复印件）或者高致病性病原微生物菌（毒）种或样本运输容器或包装材料承诺书；

（六）其他有关资料。

第七条　接收单位应当符合以下条件：

（一）具有法人资格；

（二）具备从事高致病性病原微生物实验活动资格的实验室；

国务院办公厅关于印发卫生部主要职责内设机构和人员编制规定的通知种或样本保藏、生物制品生产等的批准文件。

第八条　在固定的申请单位和接收单位之间多次运输相同品种高致病性病原微生物菌（毒）种或样本的，可以申请多次运输。多次运输的有效期为6个月；期满后需要继续运输的，应当重新提出申请。

第九条　申请在省、自治区、直辖市行政区域内运输高致病性病原微生物菌（毒）种或样本的，由省、自治区、直辖市卫生行政部门审批。

省级卫生行政部门应当对申请单位提交的申请材料及时审查，对申请材料不齐全或者不符合法定形式的，应当即时出具申请材料补正通知书；对申请材料齐全或者符合法定形式的，应当即时受理，并在5个工作日内做出是否批准的决定；符合法定条件的，颁发《可感染人类的高致病性病原微生物菌（毒）种或样本准运证书》；不符合法定条件的，应当出具不予批准的决定并说明理由。

第十条　申请跨省、自治区、直辖市运输高致病性病原微生物菌（毒）种或样本的，应当将申请材料提交运输出发地省级卫生行政部门进行初审；对符合要求的，省级卫生行政部门应当在3个工作日内出具初审意见，并将初审意见和申报材料上报卫生部审批。

卫生部应当自收到申报材料后3个工作日内做出是否批准的决定。符合法定条件的，颁发《可感染人类的高致病性病原微生物菌（毒）种或样本准运证书》；不符合法定条件的，应当出具不予批准的决定并说明理由。

第十一条　对于为控制传染病暴发、流行或者突发公共卫生事件应急处理的高致病性病原微生物菌（毒）种或样本的运输申请，省级卫生行政部门与卫生部之间可以通过传真的方式进行上报和审批；需要提交有关材料原件的，应当于事后尽快补齐。

根据疾病控制工作的需要，应当向中国疾病预防控制中心运送高致病性病原微生物菌（毒）种或样本的，向中国疾病预防控制中心直接提出申请，由中国疾病预防控制中心审批；符合法定条件的，颁发《可感染人类的高致病性病原微生物菌（毒）种或样本准运证书》；不符合法定条件的，应当出具不予批准的决定并说明理由。中国疾病预防控制中心应当将审批情况于3日内报卫生部备案。

第十二条　运输高致病性病原微生物菌（毒）种或样本的容器或包装材料应当达到国际民航组织《危险物品航空安全运输技术细则》（Doc9284包装说明PI602）规定的A类包装标准，符合防水、防破损、防外泄、耐高温、耐高压的要求，并应当印有卫生部规定的生物危险标签、标识、运输登记表、警告用语和提示用语。

第十三条　运输高致病性病原微生物菌（毒）种或样本，应当有专人护送，护送人员不得少于两人。申请单位应当对护送人员进行相关的生物安全知识培训，并在护送过程中采取相应的防护措施。

第十四条　申请单位应当凭省级以上卫生行政部门或中国疾病预防控制中心核发的《可感染人类的高致病性病原微生物菌（毒）种或样本准运证书》到民航等相关部门办理手续。

通过民航运输的，托运人应当按照《中国民用航空危险品运输管理规定》（CCAR276）和国际民航组织文件《危险物品航空安全运输技术细则》（Doc9284）的要求，正确进行分类、包装、加标记、贴标签并提交正确填写的危险品航空运输文件，交由民用航空主管部门批准的航空承运人和机场实施运输。如需由未经批准的航空承运人和机场实施运输的，应当经民用航空主管部门批准。

第十五条　高致病性病原微生物菌（毒）种或样本在运输之前的包装以及送达后包装的开启，应当在符合生物安全规定的场所中进行。

申请单位在运输前应当仔细检查容器和包装是否符合安全要求，所有容器和包装的标签以及运输登记表是否完整无误，容器放置方向是否正确。

第十六条　在运输结束后，申请单位应当将运输情况向原批准部门书面报告。

第十七条　对于违反本规定的行为，依照《病原微生物实验室生物安全管理条例》第六十二条、六十七条的有关规定予以处罚。

第十八条　高致病性病原微生物菌（毒）种或样本的出入境，按照卫生部和国家质检总局《关于加强医用特殊物品出入境管理卫生检疫的通知》进行管理。

1. 国务院办公厅：国务院办公厅关于印发卫生部主要职责内设机构和人员编制规定的通知

国办发〔2008〕81号

（十三）科技教育司。承担医药卫生实验室生物安全的监督管理工作；

1. 国务院：国务院关于印发生物产业发展规划的通知

国发〔2012〕65号

加强实验室生物安全监督管理，健全实验室生物安全体系。

1. 农业农村部：农业农村部办公厅关于加强一级、二级动物病原微生物实验室备案工作的指导意见

农办牧〔2024〕2号

为加强动物病原微生物实验室（以下简称“实验室”）生物安全管理，完善备案工作制度，规范实验室备案内容与程序，现就做好一级、二级实验室备案工作提出如下指导意见。

一、总体要求

贯彻落实习近平总书记关于生物安全的重要指示精神，严格遵守《中华人民共和国生物安全法》、《病原微生物实验室生物安全管理条例》等有关规定，完善一级、二级实验室备案制度，督促实验室设立单位主动备案；落实农业农村部门属地管理责任，做到应备尽备、底数明晰，确保实验室生物安全。

二、统一备案内容

（一）明确备案范围。从事与动物病原微生物菌（毒）种、样本有关的研究、教学、检测、诊断等活动的一级、二级实验室（含移动式实验室），均应当向农业农村部门备案。

（二）统一备案部门。设区的市级人民政府农业农村部门负责本辖区内实验室的备案登记工作。

（三）严格备案标准。备案实验室应当符合《中华人民共和国生物安全法》、《病原微生物实验室生物安全管理条例》、《实验室生物安全通用要求》（GB19489–2008）、《生物安全实验室建筑技术规范》（GB50346–2011）、《兽医实验室生物安全要求通则》（NY/T1948–2010）、《移动式实验室生物安全要求》（GB27421–2015）等规定，生物安全防护水平应当与其拟开展的实验活动相匹配。同一套生物安全管理体系下的不同实验间，作为一个实验室备案；采用不同生物安全管理体系的实验室，分别单独备案。

（四）确定备案要素。备案内容主要包括实验室及其设立单位基本情况、实验室负责人情况、工作人员情况、平面布局、主要设施设备、生物安全管理体系、拟从事的实验活动范围等。

三、规范备案程序

（一）自我评估。实验室备案前，实验室设立单位应就实验室生物安全防护水平、实验室布局合理性、安全管理体系文件内容的完整性与规范性、实验室拟从事的动物病原微生物有关实验活动的生物安全风险等进行自我评估。

（二）提交材料。新建、改建或者扩建一级、二级实验室（含已建成未备案的一级、二级实验室），实验室设立单位应及时向实验室所在地（移动式实验室设立单位所在地）设区的市级人民政府农业农村部门提交以下备案材料：一级、二级动物病原微生物实验室备案信息表（参考附件1）、实验室或实验室设立单位的法人资格证明（复印件）、实验室设立单位的生物安全组织管理框架图、实验室平面布局图、实验室主要设施设备信息和检测报告、实验室自我评估意见等。

（三）材料审核。设区的市级人民政府农业农村部门在收到实验室备案材料后，应当及时审核，对材料齐全且备案信息完整的予以备案，发放一级、二级实验室备案凭证（参考附件2）。材料不齐全或者备案信息不完整的，应在及时补正后，再予备案。

四、强化监管保障

（一）加强组织领导。农业农村部负责指导全国一级、二级实验室备案工作。省级人民政府农业农村部门负责本辖区内的实验室备案管理工作，按照法律法规规定和本指导意见要求，制定完善实验室备案管理办法。已出台的备案管理办法不符合本指导意见要求的，要组织修订完善。各级农业农村部门要加强与教育、科技、海关、林草、市场监管等部门沟通协调，共同做好实验室备案工作。水生动物病原微生物实验室备案指导意见另行制定。根据《病原微生物实验室生物安全管理条例》，军队实验室由中国人民解放军卫生主管部门负责监督管理。

（二）强化备案管理。省级人民政府农业农村部门要落实属地管理责任，建立健全备案工作机制，指导辖区内各级农业农村部门做好备案宣传，督促实验室落实生物安全主体责任。设区的市级人民政府农业农村部门应当建立监督检查制度，在发放备案凭证后，组织县级人民政府农业农村部门对备案实验室进行监督检查，发现实验室备案材料信息与实际情况不符的，应当通知实验室及时更新备案信息。设区的市级人民政府农业农村部门应当每年将备案情况汇总后报省级人民政府农业农村部门。

实验室设立单位法定代表人、实验室负责人、实验室平面布局、重要设施设备（包括生物安全柜、压力蒸汽灭菌器、生物安全型离心机等）、实验活动范围等与生物安全相关的重大事项发生变更时，应及时向原备案部门更新备案信息。其中，涉及实验室平面布局、重要设施设备、实验活动范围发生变更的，实验室设立单位应当再次自我评估（必要时可邀请专家）并提交评估意见及相关材料。移动式一级、二级实验室需异地使用的，应当提前将实验室原备案材料、工作地点、时间安排、实验活动内容、实验室负责人、工作人员等信息向原备案部门和使用地设区的市级人民政府农业农村部门报告，接受使用地人民政府农业农村部门的监督管理。实验室不再从事实验活动的，应由原备案部门注销备案。

（三）推动信息化建设。中国动物疫病预防控制中心承担全国动物病原微生物实验室信息平台建设运维工作，并制定信息填报指南。省级人民政府农业农村部门每年按指南要求组织辖区内备案实验室信息的收集、汇总、审核、填报，逐步完善全国动物病原微生物实验室信息系统数据。

1. 农业农村部办公厅、教育部办公厅、科学技术部办公厅、国家卫生健康委办公厅、海关总署办公厅、国家林业与草原局办公室、中国科学院办公厅：关于加强动物病原微生物实验室生物安全管理的通知

农办牧〔2020〕15号

为深入贯彻落实总体国家安全观，切实推进国家生物安全，进一步加强动物病原微生物实验室生物安全管理，依据《中华人民共和国动物防疫法》（以下简称《动物防疫法》）《中华人民共和国传染病防治法》（以下简称《传染病防治法》）、《病原微生物实验室生物安全管理条例》（以下简称《条例》）等有关法律法规和规章，现就做好动物病原微生物实验室生物安全管理工作有关事项通知如下。

一、充分认识做好病原微生物实验室生物安全管理工作的重要性

病原微生物实验室生物安全是国家生物安全的重要组成部分，事关养殖业生产安全、动物源性食品安全和公共卫生安全，事关国家经济发展和社会稳定。近年来，各地各有关部门认真贯彻落实《动物防疫法》《传染病防治法》《条例》等有关法律法规，密切合作，不断加强病原微生物实验室生物安全管理工作，取得积极成效，实验室生物安全水平明显提高。但是，部分实验室生物安全管理工作仍存在一些问题和隐患。各地各有关部门要切实增强做好病原微生物实验室生物安全管理的责任感和使命感，强化安全意识，健全管理措施，落实管理责任，有效防范和化解实验室生物安全风险。

二、加强动物病原微生物实验室设立与备案管理

各地畜牧兽医主管部门要加强部门合作，切实做好包括新建、改建、扩建生物安全一级、二级实验室在内的动物病原微生物实验室（含第三方实验室）的备案管理，建立完善实验室电子备案及信息化系统。配合做好新建、改建、扩建生物安全三级、四级实验室审查。建立和完善部门间生物安全三级、四级实验室管理信息交流机制，共同推动实验室依法依规建设。实验室设立的法人单位在实验室建设项目开工前，应当依法开展环境影响评价。

三、加强动物病原微生物实验活动监管

一是规范实验活动行政许可。生物安全三级、四级实验室需要从事某种高致病性动物病原微生物或者疑似高致病性动物病原微生物实验活动的，应当按照《条例》《高致病性动物病原微生物实验室生物安全管理审批办法》《农业部关于进一步规范高致病性动物病原微生物实验活动审批工作的通知》等规定，报省级以上畜牧兽医主管部门批准。严格实验活动生物安全承诺制度和实验活动情况报告制度。

任何单位和个人未经批准不得从事相关实验活动。对海关为了检验检疫工作紧急需要从事相关实验活动的，按照《条例》第二十四条规定执行。紧急实验活动结束后，应依法停止开展相关实验活动；拟继续从事相关实验活动的，应依法获得相应实验活动行政许可。二是加强实验活动监督检查。各地畜牧兽医主管部门要加强对辖区内动物病原微生物实验室及其实验活动的生物安全监督管理。严格监督执法，对未经批准从事高致病性动物病原微生物实验活动的，要依法严肃查处，对由此产生的任何科研成果均不予认可。对实验室能力条件发生变化，不再符合国家标准或有关规定的，要及时暂停或取消实验活动许可。三是加强相关科研成果发表管理。各有关部门要按照《科技部教育部农业部卫生部中科院中国科协关于加强我国病毒研究成果发表管理的通知》（国科发社〔2012〕921号）要求，加强对所属科研院所、大专院校、出版机构有关高致病性病原微生物研究成果发表的管理，将实验室生物安全管理情况纳入相关绩效评价工作。

四、加强动物病原微生物菌（毒）种保藏保存管理

依据《条例》《动物病原微生物菌（毒）种保藏管理办法》（以下简称《办法》）等规定，国家对具有保藏价值的实验活动用动物病原微生物菌（毒）种和样本实行集中保藏。除农业农村部指定的菌（毒）种保藏机构和相关专业实验室外，其他单位和个人不得保藏高致病性病原微生物菌（毒）种和样本。各保藏机构和相关专业实验室要严格按照《办法》规定要求，做好菌（毒）种和样本的收集、保藏、供应、销毁管理，建立健全生物安全和安保管理制度，确保菌（毒）种和样本安全。对于违规保存菌（毒）种和样本的，当地畜牧兽医主管部门应当监督其就地销毁或送农业农村部指定的保藏机构保存。

从事有关动物疫情监测、疫病检测诊断、检验检疫和疫病研究等相关实验室及其设立单位要切实履行实验室生物安全管理主体责任，加强菌（毒）种和样本的采集、运输、接收、使用、保存、销毁的全链条安全管理和对外交流管理。其中，对于运输高致病性动物病原微生物菌（毒）种或者样本的，应依据《条例》严格实施调运审批制度。需要运往国外的，由出发地省级畜牧兽医主管部门进行初审后，报农业农村部批准。其中，涉及《生物两用品及相关设备和技术出口管制清单》所列的菌（毒）种和样品的，申请单位还应依据《中华人民共和国生物两用品及相关设备和技术出口管制条例》及相关规章规定，获得商务部相应行政许可。从境外引进动物病原微生物菌（毒）种或者样本的，引进单位应当报农业农村部批准。其中，因科学研究等特殊需要，引进《中华人民共和国进出境动植物检疫法》第五条第一款所列禁止进境物的，还应向海关申请办理禁止进境物特许检疫审批手续。

五、加强动物病料采集和使用监管

各有关单位和个人采集病原微生物样本，应当具备《条例》第九条规定的相应设备、人员、措施、技术方法和手段等条件。对于重大动物疫病或疑似重大动物疫病，应当由动物防疫监督机构采集病料。其他单位和个人采集病料的，应当具备《重大动物疫情应急条例》第二十一条第一款规定的相应条件。采集高致病性病原微生物样本的工作人员在采集过程中应当防止病原微生物扩散和感染，并对样本的来源、采集过程和方法等作详细记录。各地畜牧兽医主管部门要加强病料采集和使用的安全监管。各实验室及其设立单位应加强相关实验活动废弃物的处置监管，保证灭菌有效、流向可追溯。

六、加强相关科研项目审查管理

各地畜牧兽医主管部门要切实做好高致病性动物病原微生物科研项目生物安全审查工作。实验室申报或者接受与高致病性动物病原微生物有关的科研项目，应当符合科研需要和生物安全要求，具备相应生物安全防护水平，并经农业农村部审查同意。教育、科技等有关部门在设立、申报和审批与高致病性动物病原微生物相关科研项目时，应当将实验室生物安全管理要求纳入相关内容。

各地各有关部门要加强协调配合，按照职责分工，切实做好动物病原微生物实验室生物安全管理工作。一是加强组织领导。按照《条例》等规定要求，切实履行好实验室生物安全管理责任，在各自职责范围内抓好实验室及其实验活动的生物安全管理工作，确保生物安全责任落实到部门、落实到单位、落实到具体人员。二是加强信息化管理。建立健全协调机制，进一步完善实验室生物安全管理信息通报制度，强化资源信息共享。三是加强宣传培训。广泛宣传病原微生物实验室的生物安全政策法规和标准，加强技术指导与服务，督促实验室健全规章制度，强化实验室人员生物安全意识，提高生物安全防范能力。四是加强实验室安全保卫工作。督促生物安全三级、四级实验室向当地公安机关备案，并接受公安机关有关实验室安全保卫工作的监督指导。

1. 国家卫生健康委办公厅、农业农村部：关于组建第四届国家病原微生物实验室生物安全专家委员会的通知

国卫办科教函〔2021〕489号

根据《生物安全法》《病原微生物实验室生物安全管理条例》有关规定，在部门推荐的基础上，经过遴选，现组建第四届国家病原微生物实验室生物安全专家委员会（以下简称专家委员会）。专家委员会由46位专家组成，设顾问3人、主任委员1人、常务副主任委员1人、副主任委员3人、秘书2人。专家委员会每届任期3年。

专家委员会受国家卫生健康委、农业农村部、海关总署等相关部门委托，承担从事高致病性病原微生物相关实验活动的实验室设立与运行的生物安全评估和技术咨询、论证工作，并对全国病原微生物实验室生物安全管理提供咨询意见。主要包括下列事项的咨询评估论证：

一、病原微生物名录（目录）；

二、病原微生物实验活动生物安全防护要求；

三、新发突发传染病病原实验室生物安全管理要求；

四、使用新技术、新方法从事高致病性病原微生物实验活动；

五、高致病性病原微生物菌（毒）种或者样本出入境生物安全风险及管理要求；

六、相关部门委托的其他事项。

1. 农业农村部：农业农村部办公厅关于进一步加强动物病原微生物实验室生物安全管理工作的通知

农办牧〔2021〕23号

为加强动物病原微生物实验室生物安全管理，有效防范化解实验室生物安全风险，依据《中华人民共和国生物安全法》（以下简称《生物安全法》）《中华人民共和国动物防疫法》《病原微生物实验室生物安全管理条例》等法律法规，现就进一步做好动物病原微生物实验室生物安全管理工作通知如下。

一、深化对动物病原微生物实验室生物安全工作的认识

动物病原微生物实验室生物安全是国家生物安全的重要组成部分，事关养殖业生产安全、动物源性食品安全和公共卫生安全。近年来，各地持续加强管理，动物病原微生物实验室生物安全水平明显提高，但新建、改建或者扩建一级、二级实验室未及时进行备案、违规开展高致病性动物病原微生物实验活动等问题时有发生。要以高度的政治责任感，深入贯彻落实《生物安全法》等法律法规要求，积极开展宣传培训，掌握核心要义，指导做好实验室生物安全管理工作，督促健全实验室管理制度，增强生物安全意识，提高生物安全风险防范能力。

二、强化动物病原微生物实验室备案管理

各地要依照相关法律法规要求，做好新建、改建或者扩建一级、二级实验室在内的动物病原微生物实验室的备案管理。要积极探索制定备案管理办法，建立完善实验室电子备案及信息化系统，做到底数清、情况明，应备尽备，不留死角，不落下一个动物病原微生物实验室。

三、进一步规范高致病性动物病原微生物行政审批

各地要落实“放管服”要求，进一步规范高致病性动物病原微生物行政审批，参照我部相关行政审批办事指南，编制省级办事指南，细化审批条件，严格审批要求，规范审批流程，按时办结并及时将申请材料和初审意见报送我部，不得由申请人代为邮寄。

四、严格动物病原微生物菌（毒）种和样本保藏管理

国家对具有保藏价值的实验活动用动物病原微生物菌（毒）种和样本实行集中保藏，除我部指定的菌（毒）种保藏机构和相关专业实验室外，其他单位和个人不得保藏菌（毒）种和样本。各地要切实履行监督职责，对违规保存菌（毒）种和样本的实验室，监督其就地销毁或送我部指定的保藏机构保存。

五、加强动物病原微生物实验室及实验活动常态化监督检查

各地要落实属地管理责任，加强对辖区内动物病原微生物实验室及其实验活动的生物安全监督管理，做好常态化监管工作。要严格监督执法，对未经批准从事高致病性动物病原微生物实验活动的，坚决依法严肃查处，对由此产生的科研成果不予认可。对实验室能力条件发生变化，不再符合国家标准或相关规定的，要及时暂停或取消实验活动许可。

六、严格科研成果发表管理

各地要按照《科技部教育部农业部卫生部中科院中国科协关于加强我国病毒研究成果发表管理的通知》（国科发社〔2012〕921号）要求，加强对所属科研院所、大专院校、出版机构高致病性病原微生物研究成果发表的管理，将实验室生物安全管理情况纳入相关绩效评价工作。

1. 农业农村部：农业农村部办公厅关于开展2020年度全国动物病原微生物实验室生物安全专项检查的通知

农办牧〔2020〕24号

为进一步加强动物病原微生物实验室生物安全管理，有效防范和化解生物安全风险，根据《病原微生物实验室生物安全管理条例》《高致病性动物病原微生物实验室生物安全管理审批办法》等法规规章，以及农业农村部等七部门联合印发《关于加强动物病原微生物实验室生物安全管理工作的通知》（农办牧〔2020〕15号），我部决定组织开展2020年度全国动物病原微生物实验室生物安全专项检查。现将有关事宜通知如下。

一、检查范围

检查对象为从事动物病原微生物实验活动的实验室，包括畜牧兽医系统、高等院校、科研院所、海关、林草、兽用生物制品生产企业、大型养殖企业、动物诊疗机构等单位相关实验室，重点检查高级别动物病原微生物实验室和各省畜牧兽医主管部门授权从事非洲猪瘟检测任务的实验室。

二、检查内容

检查内容主要包括：实验室生物安全组织机构情况；实验室生物安全管理责任制、有关规章制度制定落实情况；实验室生物安全防护措施落实情况；实验室从事动物病原微生物实验活动情况；实验室应急预案制定实施情况；菌（毒）种和样本保存、使用、销毁情况；实验室工作人员生物安全知识培训情况；实验室各类记录和档案；实验室近3年（2018—2020年）涉及高致病性动物病原微生物相关实验活动研究论文发表情况。

1. 国家卫生健康委：国家卫生健康委办公厅关于国家人间传染的病原微生物实验室生物安全评审专家委员会换届的通知

国卫办科教函〔2021〕14号

为适应病原微生物实验室生物安全工作形势，切实发挥专家在实验室生物安全监管和行政审批中的技术支撑作用，按照《生物安全法》《病原微生物实验室生物安全管理条例》等有关规定，在有关部门和单位推荐的基础上，经过培训、考核等程序，我委组织对国家人间传染的病原微生物实验室生物安全评审专家委员会进行了换届。

第三届国家人间传染的病原微生物实验室生物安全评审专家委员会由176位专家组成，设主任1人、副主任3人，并设专家委员会秘书长1人、评审秘书20人。专家委员会办公室设立在中国疾病预防控制中心，负责专家委员会日常管理，并组织开展我委职责范围内的实验室相关审批的专家评审工作。

1. 国家卫生健康委：国家卫生健康委办公厅关于印发《新型冠状病毒实验室生物安全指南（第二版）》的通知

国卫办科教函〔2020〕70号

根据目前掌握的新型冠状病毒生物学特点、流行病学特征、致病性、临床表现等信息，该病原体暂按照病原微生物危害程度分类中第二类病原微生物进行管理。

一、实验活动生物安全要求

（一）病毒培养：指病毒的分离、培养、滴定、中和试验、活病毒及其蛋白纯化、病毒冻干以及产生活病毒的重组实验等操作。上述操作应当在生物安全三级实验室内进行。使用病毒培养物提取核酸，裂解剂或灭活剂的加入必须在与病毒培养等同级别的实验室和防护条件下进行，裂解剂或灭活剂加入后可比照未经培养的感染性材料的防护等级进行操作。实验室开展相关活动前，应当报经国家卫生健康委批准，取得开展相应活动的资质。

（二）动物感染实验：指以活病毒感染动物、感染动物取样、感染性样本处理和检测、感染动物特殊检查、感染动物排泄物处理等实验操作，应当在生物安全三级实验室操作。实验室开展相关活动前，应当报经国家卫生健康委批准，取得开展相应活动的资质。

（三）未经培养的感染性材料的操作：指未经培养的感染性材料在采用可靠的方法灭活前进行的病毒抗原检测、血清学检测、核酸提取、生化分析，以及临床样本的灭活等操作，应当在生物安全二级实验室进行，同时采用生物安全三级实验室的个人防护。

（四）灭活材料的操作：感染性材料或活病毒在采用可靠的方法灭活后进行的核酸检测、抗原检测、血清学检测、生化分析等操作应当在生物安全二级实验室进行。分子克隆等不含致病性活病毒的其他操作，可以在生物安全一级实验室进行。

二、病原体及样本运输和管理

（一）国内运输：新型冠状病毒毒株或其他潜在感染性生物材料的运输包装分类属于A类，对应的联合国编号为UN2814，包装符合国际民航组织文件Doc9284《危险品航空安全运输技术细则》的PI602分类包装要求；环境样本属于B类，对应的联合国编号为UN3373，包装符合国际民航组织文件Doc9284《危险品航空安全运输技术细则》的PI650分类包装要求；通过其他交通工具运输的可参照以上标准包装。

新型冠状病毒毒株或其他潜在感染性材料运输应当按照《可感染人类的高致病性病原微生物菌（毒）种或样本运输管理规定》（卫生部令第45号）办理《准运证书》。

（二）国际运输：新型冠状病毒毒株或样本在国际间运输的，应当规范包装，按照《出入境特殊物品卫生检疫管理规定》办理相关手续，并满足相关国家和国际相关要求。

（三）毒株和样本管理：新型冠状病毒毒株和相关样本应当由专人管理，准确记录毒株和样本的来源、种类、数量、编号登记，采取有效措施确保毒株和样本的安全，严防发生误用、恶意使用、被盗、被抢、丢失、泄露等事件。

三、废弃物管理

（一）开展新型冠状病毒相关实验活动的实验室应当制定废弃物处置程序文件及污物、污水处理操作程序。

（二）所有的危险性废弃物必须依照统一规格化的容器和标示方式，完整并且合规地标示废弃物内容。

（三）应当由经过适当培训的人员使用适当的个人防护装备和设备处理危险废弃物。

（四）废弃物的处理措施：废弃物的处理是控制实验室生物安全的关键环节，切实安全地处理感染性废弃物，必须充分掌握生物安全废弃物的分类，并严格执行相应的处理程序。

1.废液的处理：实验室产生的废液可分为普通污水和感染性废液。

（1）普通污水产生于洗手池等设备，对此类污水应当单独收集，排入实验室水处理系统，经处理达标后方可排放。

（2）感染性废液即在实验操作过程中产生的废水，采用化学消毒或物理消毒方式处理，并对消毒效果进行验证，确保彻底灭活。

（3）工作人员应当及时处理废弃物，不得将废弃物带出实验区。

2.固体废物的处理：

（1）固体废物分类收集，固体废物的收集容器应当具有不易破裂、防渗漏、耐湿耐热、可密封等特性。实验室内的感染性垃圾不允许堆积存放，应当及时压力蒸汽灭菌处理。废物处置之前，应当存放在实验室内指定的安全地方。

（2）小型固体废物如组织标本、耗材、个人防护装备等均需经过压力蒸汽灭菌处理，再沿废弃物通道移出实验室。

（3）体积较大的固体废物如HEPA过滤器，应当由专业人士进行原位消毒后，装入安全容器内进行消毒灭菌。不能进行压力蒸汽灭菌的物品如电子设备可以采用环氧乙烷熏蒸消毒处理。

（4）经消毒灭菌处理后移出实验室的固体废物，集中交由固体废物处理单位处置。

（5）实验过程如使用锐器（包括针头、小刀、金属和玻璃等）要直接弃置于锐器盒内，高压灭菌后，再做统一处理。

（五）建立废弃物处理记录：定期对实验室排风HEPA过滤器进行检漏和更换，定期对处理后的污水进行监测，采用生物指示剂监测压力蒸汽灭菌效果。

四、实验室生物安全操作失误或意外的处理

（一）新型冠状病毒毒株或其他潜在感染性材料污染生物安全柜的操作台造成局限污染：使用有效氯含量为0.55%消毒液，消毒液需要现用现配，24小时内使用。此后内容中有效氯含量参照此浓度。

（二）含病毒培养器皿碎裂或倾覆造成实验室污染：保持实验室空间密闭，避免污染物扩散，使用0.55%有效氯消毒液的毛巾覆盖污染区。必要时(大量溢撒时)可用过氧乙酸加热熏蒸实验室，剂量为2g/m3，熏蒸过夜；或20g/L过氧乙酸消毒液用气溶胶喷雾器喷雾，用量8ml/m3，作用1～2小时；必要时或用高锰酸钾-甲醛熏蒸：高锰酸钾8g/m3，放入耐热耐腐蚀容器（陶罐或玻璃容器），后加入甲醛（40%）10ml/m3，熏蒸4小时以上。熏蒸时室内湿度60%-80%。

（三）清理污染物严格遵循活病毒生物安全操作要求，采用压力蒸汽灭菌处理，并进行实验室换气等，防止次生危害。

1. 农业农村部：农业农村部办公厅关于成立农业农村部动物病原微生物实验室生物安全评审专家委员会的通知

农办牧〔2019〕81号

为加强动物病原微生物实验室生物安全管理工作，2009年1月我部成立了动物病原微生物实验室生物安全评审专家库（以下简称“专家库”）。专家库成立以来，在推动实验室生物安全管理技术标准规范制修订、实验活动生物安全技术评审、支持动物疫病防控及其技术研发、有效防范和化解实验室生物安全风险等方面发挥了重要作用。为进一步加强实验室生物安全管理，我部决定成立农业农村部动物病原微生物实验室生物安全评审专家委员会（以下简称“委员会”），并按程序确定了委员会章程及委员会委员名单，现予以公布。

1. 农业农村部：农业部办公厅关于开展2016年动物病原微生物实验室生物安全专项检查工作的通知

农办医〔2016〕38号

一、检查范围

从事动物病原微生物实验活动的实验室，包括兽医系统、教学科研单位、出入境检验检疫部门、兽用生物制品生产企业、大型养殖企业及动物诊疗机构等单位的相关实验室。

二、检查形式

（一）生物安全自查。各有关实验室应自行开展生物安全自查。各省级兽医主管部门负责组织本地相关实验室生物安全自查工作。

（二）生物安全抽查。各省级兽医主管部门根据辖区内各实验室设立单位报送的自查报告，在不同领域选取一定数量的实验室开展生物安全抽查。农业部兽医局将根据工作需要，派员参加相关省份生物安全抽查活动。

三、检查时间

（一）生物安全自查。2016年7月31日前完成；

（二）生物安全抽查。2016年10月31日前完成。

四、检查内容

（一）动物病原微生物实验室建设情况；

（二）动物病原微生物实验室生物安全组织机构情况；

（三）动物病原微生物实验室从事高致病性动物病原微生物实验活动情况；

（四）动物病原微生物实验室生物安全防护措施落实情况；

（五）动物病原微生物实验室生物安全管理责任制和有关规章制度落实情况；

（六）动物病原微生物实验室应急预案制定和实施情况；

（七）动物病原微生物菌（毒）种或者样本保存、销毁情况；

（八）动物病原微生物实验室各类记录和档案；

（九）动物病原微生物实验室工作人员生物安全培训情况；

详细检查内容见《动物病原微生物实验室生物安全检查表》。

五、工作要求

（一）加强组织领导。各级兽医主管部门要高度重视，切实加强组织领导，把实验室生物安全工作纳入重要议事日程。要明确专人负责上述检查工作，6月25日前将联系人有关信息（附件2）报我部兽医局，同时抄送中国动物疫病预防控制中心。

（二）精心组织实施。各级兽医主管部门要在我部制定的检查方案基础上，结合本地区实际情况，进一步细化检查方案，确保检查工作顺利开展。自查内容可在本通知所列内容上适当增加。

（三）做好总结分析。各地要及时总结生物安全检查情况，分析存在问题，提出工作建议，分别于8月15日和11月10日前将专项自查情况总结（包括辖区内生物安全自查汇总表）（附件3）和生物安全专项抽查情况总结报我部兽医局，同时抄送中国动物疫病预防控制中心。

（四）及时更新填报数据。各地要根据本次生物安全专项自查和抽查情况，及时更新全国兽医实验室信息管理系统数据。

1. 科学技术部：关于加强高等级病原微生物实验室建设审查工作的通知

国科办社〔2011〕50号

根据《病原微生物实验室生物安全管理条例》（国务院令第424号，以下简称《条例》）第十九条“新建、改建、扩建三级、四级实验室或者生产、进口移动式三级、四级实验室应当...经国务院科技主管部门审查同意”的规定，科学技术部研究制定了《高等级病原微生物实验室建设审查办法》（以下简称《办法》），对新建、改建、扩建三级、四级实验室或者生产、进口移动式三级、四级实验室应当具备的条件及申请审查程序作出了规定，已于2011年6月24日以科学技术部令第15号公布，自2011年8月1日起施行。

有关单位在新建、改建、扩建三级、四级实验室或者生产、进口移动式三级、四级实验室时均应按照《条例》和《办法》的规定执行。未按《条例》要求已经建设和运行的高等级病原微生物实验室，请于2012年1月31日之前按照《条例》和《办法》的规定报科学技术部进行审查，补充办理相关手续。

1. 农业农村部：农业部办公厅关于进一步做好动物病原微生物实验室生物安全监管有关工作的通知

农办医〔2016〕15号

“十三五”期间，动物疫病科研、监测等工作要求更高、任务更重。为进一步做好动物病原微生物实验室生物安全监管工作，保障动物病原微生物实验室生物安全，根据有关法律法规和我部相关规定，现将有关事宜通知如下。

一、充分认识做好动物病原微生物实验室生物安全监管工作的重要性

动物病原微生物实验室生物安全是生物安全的重要组成部分，不仅直接关系到动物疫病防控和公众健康，而且关系到社会稳定和国家安全。近年来，各级兽医主管部门认真贯彻落实《病原微生物实验室生物安全管理条例

》（以下简称《条例》）等法律法规规定和我部要求，严格依法开展动物病原微生物实验室生物安全监管工作，督促有关实验室落实生物安全责任制、完善内部管理制度，有力保障了公共卫生安全和生物安全。但个别实验室仍存在未经批准擅自开展高致病性动物病原微生物实验活动、申报或接受科研项目甚至擅自保存高致病性动物病原微生物菌（毒）种的情况。各级兽医主管部门要从维护公共卫生安全和国家生物安全的高度，充分认识进一步好动物病原微生物实验室生物安全监管有关工作的重要性和紧迫性，严格依法做好有关工作。

二、依法严格做好动物病原微生物实验室生物安全审批有关工作

为落实好《条例》规定的审批和监管职责，我部先后印发《高致病性动物病原微生物实验室生物安全管理审批办法

》等十余个规章和文件，对高致病性动物病原微生物实验室生物安全管理审批工作做了明确规定。各省级兽医主管部门要严格依据《条例》和上述规章、文件，以及国务院行政审批改革要求，切实做好高致病性动物病原微生物菌（毒）种或者样本省内运输、部分高致病性动物病原微生物实验活动的行政审批工作，及时将批准的实验活动报我部备案；做好高致病性动物病原微生物菌（毒）种或者样本跨省或向境外运输、高致病性动物病原微生物实验室资格、有关高致病性动物病原微生物实验活动审批的初审工作，同步做好网上办理；督促辖区内有关科研单位，在申报或接受与高致病性动物病原微生物有关的科研项目前，向我部申请审查。

三、加强动物病原微生物实验室及实验活动的日常监管

各级兽医部门要严格依法加强对辖区内动物病原微生物实验室及其实验活动的生物安全监督检查。进一步摸清辖区内动物病原微生物实验室底数；制定生物安全监督检查计划，加强日常监管，加强对实验室生物安全重点环节、重点部位的监督检查，根据问题开展专项检查；按照《科技部 教育部 农业部 卫生部 中科院 中国科协关于加强我国病毒研究成果发表管理的通知》（国科发社〔2012〕921号）规定，做好有关机构病毒研究成果发表审查工作。要监督有关实验室完善并切实执行实验室生物安全管理、安全防护、感染控制和安全事故应急、人员考核培训、档案管理等工作制度；严格按照批复内容开展实验活动，认真执行实验活动定期报告制度；严格按要求，及时销毁高致病性动物病原微生物菌（毒）种、样本或送我部指定的菌（毒）种保藏机构保藏。我部将根据需要，及时组织开展监督抽查，重点检查高致病性病原微生物科研项目审查申请、实验活动开展等情况。

1. 生态环境部：国家环境保护总局办公厅关于组织开展病原微生物实验室环境现场检查工作的通知

环办〔2007〕71号

为认真贯彻落实《病原微生物实验室生物安全管理条例》（国务院令第424号，以下简称《条例》）和《病原微生物实验室生物安全环境管理办法》（国家环境保护总局令第32号，以下简称《办法》），加强对病原微生物实验室(以下简称实验室)的环境监管，各级环境保护部门要认真开展实验室环境现场检查工作。现将有关事项通知如下：

一、充分认识加强实验室环境监管工作的重要性。2003年SARS疫情特别是实验室SARS病毒泄露事件发生后，党中央、国务院对实验室生物安全管理工作高度重视。保护实验室工作人员和公众健康已经摆上重要议事日程，加强实验室环境现场检查工作是实现实验室生物安全管理的重要条件，也是日常环境监察的重要内容。各地要高度重视，认真组织开展。

二、明确现场检查权限，依法处理环境违法行为。县（区）级环境保护行政主管部门要负责检查当地的一级、二级实验室，市（地）级环境保护行政主管部门要负责检查当地的三级、四级实验室。需要进入从事高致病性病原微生物实验活动的实验室进行调查取证、采集样品的，应当委托专业机构实施。

三、加强业务培训，确保执法人员的安全。对三级、四级实验室的环境执法要有专人负责，执法人员要经过业务和安全培训。进入实验室的现场执法人员必须严格遵守实验室的安全要求。

四、根据实验室的具体情况，确定主要检查内容。一般包括四个方面：

（一）实验室环保审批手续办理情况

1、实验室是否依法履行环境影响评价审批手续，实验室从事的实验活动是否符合环境影响评价报告书的内容。

2、实验室是否依法办理“三同时”竣工环保验收手续。未投入使用的实验室，污染防治设施与主体工程是否同时施工；投入使用的实验室，污染防治设施与主体工程是否同时建成并投入运营。

（二）实验室有关环境管理措施落实情况

1、三级、四级实验室是否按照《病原微生物实验室生物安全环境管理办法》第九条的规定，向所在地县级人民政府环境保护行政主管部门备案。

2、实验室是否按照《病原微生物实验室生物安全环境管理办法》第十一条、第十六条和第十七条的规定，落实实验室污染防治责任、建立实验档案、编写环境应急预案和定期进行环境应急演练。

（三）实验室对实验活动产生的废水、废气和危险废物处置情况

1、实验室废水处理、消毒与排放是否符合有关标准规定的污水排放限值和控制要求。

2、实验室废气处理、消毒与排放是否符合有关标准规定的废气排放限值和控制要求，大气污染防治设施是否按规定正常运转。

3、危险废物处置是否符合有关标准规定的固体废物和污泥控制要求，是否符合《病原微生物实验室生物安全环境管理办法》第十五条的规定。

4、高致病性病原微生物菌（毒）种或者样品的运送方式是否符合环境影响评价文件的要求，是否采取了相应的环境保护措施。

五、将实验室环境监管纳入日常环境监察范围。对新建、改建、扩建实验室，要检查其环保“三同时”执行情况。对已建成的实验室，要纳入排污申报范围，依法征收排污费，并定期开展检查。上级环保部门要检查下级环保部门对三级、四级实验室的备案情况。

1. 农业农村部：农业农村部办公厅关于开展2018年全国动物病原微生物实验室生物安全专项检查工作的通知

农办医[2018]12号

为切实做好动物病原微生物实验室生物安全管理，维护养殖业生产安全、动物源性食品安全、公共卫生安全和生态安全，根据《病原微生物实验室生物安全管理条例》《高致病性动物病原微生物实验室生物安全管理审批办法》《兽医实验室生物安全要求通则》等法规规章和我部《2018年兽医工作要点》，决定组织开展动物病原微生物实验室生物安全专项检查。现将有关事宜通知如下。

一、原则与目标

深入贯彻党的十九大精神，紧紧围绕“防风险、保安全、促发展”目标，按照“统一标准、属地负责、全面排查、重点管控”的工作原则，落实各级兽医主管部门的监管责任，强化各级各类动物病原微生物实验室设立单位及上级主管部门的日常管理责任，对动物病原微生物实验室特别是高致病性动物病原微生物实验室开展生物安全专项检查，排除实验室生物安全隐患，进一步提高实验室生物安全管理水平。

二、检查范围

从事动物病原微生物实验活动的实验室，包括兽医系统、教学科研单位、出入境检验检疫部门、兽用生物制品生产企业、大型养殖企业及动物诊疗机构等单位的相关实验室。

三、检查内容

实验室建设和运行情况；实验室生物安全组织机构情况；实验室生物安全管理责任制和有关规章制度落实情况；实验室生物安全防护措施落实情况；实验室从事高致病性动物病原微生物实验活动情况；实验室应急预案制定和实施情况；菌（毒）种和样本保存、销毁情况；实验室工作人员生物安全知识培训情况；实验室各类记录和档案。

1. 农业农村部：兽医实验室生物安全管理规范

2003年农业部公告第302号

1 适用范围

本规范规定了兽医实验室生物安全防护的基本原则、实验室的分级、各级实验室的基本要求和管理。本规范为最低要求。

本规范适用于各级兽医实验室的建设、使用和管理。

2 引用标准

本规范引用下列文件中的条款作为本规范的条款。凡注日期的引用文件，其随后所有的修改（不包括勘误的内容）或修订版均不适用于本规范。凡不注日期的引用文件，其最新版本适用于本规范。

《中华人民共和国动物防疫法》（1997）

《中华人民共和国进出境动植物检疫法》（1992）

《中华人民共和国进出境动植物检疫法实施条例》（1995）

《农业转基因生物安全管理条例》（2001国务院304号令）

《农业生物基因工程安全管理实施办法》（1996农业部7号令）

《实验动物管理条例》(1988国家科委2号令)

GB 14925-2001 实验动物 环境与设施

GB/T 15481-2000 检测和校准实验室能力的通用要求

GB/T 16803-1997 采暖、通风、空调、净化设备术语

GB/T 14295-93 空气过滤器

GB/13554-92 高效空气过滤器

GB 50155-92 采暖通风与空气调节术语标准

GBJ 19-87 采暖通风与空气调节设计规范

WS 233-2002 微生物和生物医学实验室生物安全通用准则

OIE 2002 国际动物卫生法典

JCJ 71-90 洁净室施工及验收规范

NF EN 12021 可呼吸空气生产标准

3 定义

本规范采用下列定义：

兽医实验室(Veterinary LaboratorY)：一切从事兽医病原微生物、寄生虫研究与使用，以及兽医临床诊疗和疫病检疫监测的实验室。

动物(Animal)：本规范涉及的动物是指家畜家禽和人工饲养、合法捕获其他动物。

兽医微生物(Veterinary Microorganisms)：一切能引起动物传染病或人畜共患病的细菌、病毒和真菌等病原体。

人畜共患病(Zoonosis)：可以由动物传播给人并引起人类发病的传染性疾病。

外来病(Exotic Diseases)：在国外存在或流行的，但在国内尚未证实存在或已消灭的动物疫病。

实验室生物安全防护（Biosafety Containment of Laboratories）： 实验室工作人员在处理病原微生物、含有病原微生物的实验材料或寄生虫时，为确保实验对象不对人和动物造成生物伤害，确保周围环境不受其污染，在实验室和动物实验室的设计与建造、使用个体防护装置、严格遵守标准化的工作及操作程序和规程等方面所采取的综合防护措施。

微生物危害评估（Hazard Assessment of Microbes）：对病原微生物或寄生虫可能给人、动物和环境带来的危害所进行的评估。

气溶胶（Aerosol）：悬浮于气体介质中粒径为0.001-100μm的固体、液体微小粒子形成的胶溶状态分散体系。

通风橱（Chemical Hood）：是通过管道直接排出操作化学药品时所产生的有害或挥发性气体、气溶胶和微粒的通风装置。

高效空气过滤器(HEPA, High Efficiency Particulate Air-filter)： 在额定风量下，对粒径大于等于0.3μm的粒子捕集效率在99.97%以上及气流阻力在245Pa以下的空气过滤器。

物理防护设备（Physical Containment Device）：是用于防止病原微生物逸出和对操作者实施防护的物理或机械设备。

生物安全柜（Biosafety Cabinet）：处理危险性微生物时所用的箱形负压空气净化安全设备。分为Ⅰ、Ⅱ和Ⅲ级。

生物安全柜的简单分类及应用

柜子 应用

类型 面速度（英尺/分） 气流方式 放射性元素/有毒化学物操作 生物安全水平 产品防护

Ⅰ级 前开门式75 前面进，后面出，顶部通过HEPA过滤器 不能 2,3 无

Ⅱ级 A型75 70%通过HEPA循环，通过HEPA排出 不能 2,3 有

B1型100 30%通过HEPA循环，通过HEPA和严格管道排出 能（低水平/挥发性） 2,3 有

B2型100 无循环，全部通过HEPA和严格管道排出 能 2,3 有

B3型100 同ⅡA，但箱内呈负压和管道排气 能 2,3 有

Ⅲ级无要求 供气进口和排气通过两道HEPA过滤器 能 3,4 有

4 实验室生物安全防护的基本原则

4.1总则

4.1.1兽医实验室生物安全防护内容包括安全设备、个体防护装置和措施（一级防护），实验室的特殊设计和建设要求（二级防护），严格的管理制度和标准化的操作程序与规程。

4.1.2兽医实验室除了防范病原体对实验室工作人员的感染外，还必须采取相应措施防止病原体的逃逸。

4.1.3对每一特定实验室，应制定有关生物安全防护综合措施，编写各实验室的生物安全管理手册，并有专人负责生物安全工作。

4.1.4生物安全水平根据微生物的危害程度和防护要求分为4个等级，即Ⅰ、Ⅱ、Ⅲ、Ⅳ级。

4.1.5有关DNA重组操作和遗传工程体的生物安全应参照《农业生物基因工程安全管理实施办法》执行。

4.2安全设备和个体防护

确保实验室工作人员不与病原微生物直接接触的初级屏障。

4.2.1实验室必须配备相应级别的生物安全设备。所有可能使病原微生物逸出或产生气溶胶的操作，必须在相应等级的生物安全控制条件下进行。

4.2.2实验室工作人员必须配备个体防护用品（防护帽、护目镜、口罩、工作服、手套等）。

4.3 实验室选址、设计和建造的要求

实验室的选址、设计和建造应考虑对周围环境的影响。

4.3.1实验室必须依据所需要的防护级别和标准进行设计和建造，并满足本规范中的最低设计要求和运行条件。

4.3.2 动物实验室除满足相应生物安全级别要求外，还应隔离，并根据其相应生物安全级别，保持与中心实验室的相应压差。

4.4生物安全操作规程

4.4.1本规范规定了不同级别的兽医实验室生物安全操作规程，必须在各实验室的生物安全管理手册中明列，并结合实际制定相应的实施方案。

4.4.2本规范对各种病原微生物均有明确的生物危害分类，各实验室应根据其操作的对象，制定相应的特殊生物安全操作规程，并列入其生物安全管理手册。

4.5 危害性微生物及其毒素样品的引进、采集、包装、标识、传递和保存

4.5.1采集的样品应放入安全的防漏容器内，传递时必须包装结实严密，标识清楚牢固，容器表面消毒后由专人送递或邮寄至相应实验室。

4.5.2 进口危害性微生物及其毒素样品时，申请者必须要有与该微生物危害等级相应的生物安全实验室，并经国务院畜牧兽医行政管理部门批准。

4.5.3 危害性微生物及其毒素样品的保存应根据其危害等级分级保存。

4.6 使用放射性同位素的生物安全防护要求参照《放射性同位素与射线装置放射防护条例》执行。

4.7去污染与废弃物（废气、废液和固形物）处理

4.7.1 去污染包括灭菌（彻底杀灭所有微生物）和消毒（杀灭特殊种类的病原体），是防止病原体扩散造成生物危害的重要防护屏障。

4.7.2被污染的废弃物或各种器皿在废弃或清洗前必须进行灭菌处理；实验室在病原体意外泄漏、重新布置或维修、可疑污染设备的搬运以及空气过滤系统检修时，均应对实验室设施及仪器设备进行消毒处理。

4.7.3 根据被处理物的性质选择适当的处理方法，如高压灭菌、化学消毒、熏蒸、γ-射线照射或焚烧等。

4.7.4对实验动物尸体及动物产品应按规定作无害化处理。

4.7.5实验室应尽量减少用水，污染区、半污染区产生的废水必须排入专门配备的废水处理系统，经处理达标后方可排放。

4.8管理制度 兽医实验室必须建立健全管理制度。

4.9微生物危害评估

按照微生物危害分为4级。在建设实验室之前，必须对拟操作的病原微生物进行危害评估，结合人和动物对其易感性、气溶胶传播的可能性、预防和治疗的获得性等因素，确定相应生物安全水平等级。

5 微生物危害分级

5.1. 微生物危害通常分为以下4级

生物危害1级：对个体和群体危害程度低，已知的不能对健康成年人和动物致病的微生物。

生物危害2级：对个体危害程度为中度，对群体危害较低，主要通过皮肤、粘膜、消化道传播。对人和动物有致病性，但对实验人员、动物和环境不会造成严重危害的动物致病微生物，具有有效的预防和治疗措施。

生物危害3级：对个体危害程度高，对群体危害程度较高。能通过气溶胶传播的，引起严重或致死性疫病，导致严重经济损失的动物致病微生物，或外来的动物致病微生物。对人引发的疾病具有有效的预防和治疗措施。

生物危害4级：对个体和群体的危害程度高，通常引起严重疫病的、暂无有效预防和治疗措施的动物致病微生物。通过气溶胶传播的，有高度传染性、致死性的动物致病微生物；或未知的危险的动物致病微生物。

5.2 根据对象微生物本身的致病特征确定微生物的危害等级时必须考虑下列因素

●微生物的致病性和毒力

●宿主范围

●所引起疾病的发病率和死亡率

●疾病的传播媒介

●动物体内或环境中病原的量和浓度

●排出物传播的可能性

●病原在自然环境中的存活时间

●病原的地方流行特性

●交叉污染的可能性

●获得有效疫苗、预防和治疗药物的程度

5.3 除考虑特定微生物固有的致病危害外，危害评估还应包括

●产生气溶胶的可能性

●操作方法（体外、体内或攻毒）

●对重组微生物还应评估其基因特征（毒力基因和毒素基因）、宿主适应性改变、基因整合、增殖力和回复野生型的能力等。

6 兽医实验室的分类、分级及其适用范围

6.1分类：兽医实验室分两类。

6.1.1生物安全实验室

是指对病原微生物进行试验操作时所产生的生物危害具有物理防护能力的兽医实验室。适用于兽医微生物的临床检验检测、分离培养、鉴定以及各种生物制剂的研究等工作。

6.1.2 生物安全动物实验室

是指对病原微生物的动物生物学试验研究时所产生的生物危害具有物理防护能力的兽医实验室。也适用于动物传染病临床诊断、治疗、预防研究等工作。

6.2分级

上述两类实验室，根据所用病原微生物的危害程度、对人和动物的易感性、气溶胶传播的可能性、预防和治疗的可行性等因素，其实验室生物安全水平各分为四级，一级最低，四级最高。

6.2.1.生物安全水平分级依据

一级生物安全水平（BSL-1）：能够安全操作，对实验室工作人员和动物无明显致病性的，对环境危害程度微小的，特性清楚的病原微生物的生物安全水平。

二级生物安全水平（BSL-2）：能够安全操作，对实验室工作人员和动物致病性低的，对环境有轻微危害的病原微生物的生物安全水平。

三级生物安全水平（BSL-3）：能够安全地从事国内和国外的，可能通过呼吸道感染，引起严重或致死性疾病的病原微生物工作的生物安全水平。与上述相近的或有抗原关系的，但尚未完全认知的病原体，也应在此种水平条件下进行操作，直到取得足够的数据后，才能决定是继续在此种安全水平下工作还是在其它等级生物安全水平下工作。

四级生物安全水平（BSL-4）：能够安全地从事国内和国外的，能通过气溶胶传播，实验室感染高度危险，严重危害人和动物生命和环境的，没有特效预防和治疗方法的微生物工作的生物安全水平。与上述相近的或有抗原关系的，但尚未完全认识的病原体也应在此种水平条件下进行操作，直到取得足够的数据后，才能决定是继续在此种安全水平下工作还是在低一级安全水平下工作。

6.2.2 动物实验生物安全水平（ABSL）

一级动物实验生物安全水平（ABSL-1）:能够安全地进行没有发现肯定能引起健康成人发病的,对实验室工作人员、动物和环境危害微小的、特性清楚的病原微生物感染动物工作的生物安全水平。

二级动物实验生物安全水平（ABSL-2）:能够安全地进行对工作人员、动物和环境有轻微危害的病原微生物感染动物的生物安全水平。这些病原微生物通过消化道和皮肤、粘膜暴露而产生危害。

三级动物实验生物安全水平（ABSL-3）:能够安全地从事国内和国外的，可能通过呼吸道感染、引起严重或致死性疾病的病原微生物感染动物工作的生物安全水平。与上述相近的或有抗原关系的但尚未完全认识的病原体感染，也应在此种水平条件下进行操作，直到取得足够的数据后，才能决定是继续在此种安全水平下工作还是在低一级安全水平下工作。

四级动物实验生物安全水平（ABSL-4）:能够安全地从事国内和国外的，能通过气溶胶传播，实验室感染高度危险、严重危害人和动物生命和环境的，没有特效预防和治疗方法的微生物感染动物工作的生物安全水平。与上述相近的或有抗原关系的，但尚未完全认知的病原体动物试验也应在此种水平条件下进行操作，直到取得足够的数据后，才能决定是继续在此种安全水平下工作还是在低一级安全水平下工作。

6.3 实验室致病微生物的生物安全等级见附表一

7 实验室生物安全的物理防护分级和组合

7.1初级物理防护屏障

实验室生物安全必须配备初级物理防护屏障，它包括各级生物安全设备和个人防护器具。

7.2次级物理防护屏障

实验室的设施结构和通风设计构成次级物理防护屏障。次级物理防护的能力取决于实验室分区和室内气压，要根据实验室的安全要求进行设计。一般把实验室分为洁净、半污染和污染三个区。实验室保持密闭，通风的气流方向始终保持：外界→HEPA→洁净区→半污染区→污染区→HEPA→外界。三级和四级生物安全水平的实验室中，污染区和半污染区的气压相对于大气压的压差分别不应小于-50Pa和-30Pa.

7.3 生物安全水平（BSL）的构成

生物安全水平依赖于初级防护屏障、次级防护屏障和操作规程。三者不同形式的组合构成了4个级别生物安全水平，Ⅰ、Ⅱ、Ⅲ、Ⅳ级安全水平逐级提高，从而构成Ⅰ、Ⅱ、Ⅲ、Ⅳ级实验室生物安全。应根据实验的生物安全要求进行各种组合的设计。

7.4 各级生物安全实验室要求

7.4.1一级生物安全实验室

指按照BSL-1标准建造的实验室，也称基础生物实验室。在建筑物中,实验室无需与一般区域隔离。实验室人员需经一般生物专业训练。其具体标准、微生物操作、安全设备、实验室设施要求如下。

7.4.1.1标准操作

●工作一般在桌面上进行,采用微生物的常规操作。工作台面至少每天消毒一次。

●工作区内不准吃、喝、抽烟、用手接触隐形眼镜、存放个人物品（化妆品、食品等）。

●严禁用嘴吸取试验液体,应该使用专用的移液管。

●防止皮肤损伤。

●所有操作均需小心,避免外溢和气溶胶的产生。

●所有废弃物在处理之前用公认有效的方法灭菌消毒。从实验室拿出消毒后的废弃物应放在一个牢固不漏的容器内,并按照国家或地方法规进行处理。

●昆虫和啮齿类动物控制方案应参照其它有关规定进行。

7.4.1.2特殊操作：无。

7.4.1.3.安全设备(初级防护屏障)

●BSL-1实验室可不配置特殊的物理防护设备。

●工作时应穿着实验室专用长工作服。

●戴乳胶手套。

●可佩戴防护眼镜或面罩。

7.4.1.4实验室设施(次级防护屏障)

●实验室有控制进出的门。

●每个实验室应有一个洗手池。

●室内装饰便于打扫卫生，不用地毯和垫子。

●工作台面不漏水、耐酸碱和中等热度、抗化学物质的腐蚀。

●实验室内器具安放稳妥,器具之间留有一定的距离,方便清扫。

●实验室的窗户,必须安纱窗。

7.4.2 二级生物安全实验室

指按照BSL-2标准建造的实验室，也称为基础生物实验室。在建筑物中,实验室无需与一般区域隔离。实验室人员需经一般生物专业训练。其具体标准微生物操作、特殊操作、安全设备、实验室设施要求如下。

7.4.2.1标准操作

●工作一般在桌面上进行,采用微生物的常规操作和特殊操作。

●工作区内禁止吃、喝、抽烟、用手接触隐形眼镜和使用化妆品。食物贮藏在专门设计的工作区外的柜内或冰箱内。

●使用移液管吸取液体,禁止用嘴吸取。

●操作传染性材料后要洗手,离开实验室前脱掉手套并洗手。

●制定对利器的安全操作对策（见7.4.3.2的避免利器感染）。

●所有操作均须小心,以减少实验材料外溢、飞溅、产生气溶胶。

●每天完成实验后对工作台面进行消毒。实验材料溅出时，要用有效的消毒剂消毒。

●所有培养物和废弃物在处理前都要用高压蒸汽灭菌器消毒。消毒后的物品要放入牢固不漏的容器内, 按照国家法规进行包装，密闭传出处理。

●昆虫和啮齿类动物的控制应参照其它有关规定进行。

●妥善保管菌、毒种，使用要经负责人批准并登记使用量。

7.4.2.2特殊操作

●操作传染性材料的人员，由负责人指定。一般情况下受感染概率增加或受感染后后果严重的人不允许进入实验室。例如,免疫功能低下或缺陷的人受感染危险增加。

●负责人要告知工作人员工作中的潜在危险和所需的防护措施(如免疫接种),否则不能进入实验室工作。

●操作病原微生物期间，在实验室入口必须标记生物危险信号，其内容包括微生物种类、生物安全水平、是否需要免疫接种、研究者的姓名和电话号码、进入人员必须佩戴的防护器具、遵守退出实验室的程序。

●实验室人员需操作某些人畜共患病病原体时应接受相应的疫苗免疫或检测试验(如狂犬病疫苗和TB皮肤试验)。

●应收集和保存实验室人员和其他受威胁人的基础血清，进行试验病原微生物抗体水平的测定，以后定期或不定期收取血清样本进行监测。

●实验室负责人应制定具体的生物安全规则和标准操作程序,或制定实验室特殊的安全手册。

●实验室负责人对实验人员和辅助人员要进行针对性的生物危害防护的专业训练, 定期培训。必须防止微生物暴露、学会评价暴露危害的方法。

●必须高度重视污染利器包括针头、注射器、玻璃片、吸管、毛细管和手术刀的安全对策（见7.4.3.2的避免利器感染）。

●培养物、组织或体液标本的收集、处理、加工、储存、运输过程，应放在防漏的容器内进行。

●操作传染性材料后,应对使用的仪器表面和工作台面进行有效的消毒,特别是发生传染性材料外溢、溅出,或其它污染时更要严格消毒。污染的仪器在送出设施检修、打包、运输之前都要给予消毒。

●发生传染性材料溅出或其它事故要立即报告负责人,负责人要进行恰当的危害评价、监督、处理,并记录存档。

●非本实验所需动物不允许进入实验室。

7.4.2.3安全设备(初级防护屏障)

●实验室内工作必需穿防护工作服。离开实验室到非工作区(如餐厅、图书室和办公室)之前要脱掉工作服。所有工作服或在实验室处理或由洗衣房清洗，不准带回家。

●可能接触传染性材料和接触污染表面时要戴乳胶手套。完成传染性材料工作之后需经过消毒处理，方可脱掉手套。待处理的手套不能接触清洁表面（微机键盘、电话等），不能丢弃至实验室外面。脱掉手套后要洗手。如果手套破损，先消毒后脱掉。

●能产生传染物外溢、溅出和气溶胶的操作,包括离心、研磨、搅拌、强力震荡混合、超声波破碎、打开装有传染性材料的容器、动物鼻腔注射、收取感染动物和孵化卵的组织等，都要使用Ⅱ级生物安全柜和物理防护设备。

●离心高浓度和大容量的传染性材料时，如果使用密闭转头、带有安全帽的离心机可在开放的实验室内进行,否则只能在生物安全柜内进行。

●当操作(微生物)不得不在安全柜外面进行时,应采取严格的面部安全防护措施(护目镜、口罩、面罩或其它设施),并防止气溶胶发生。

7.4.2.4实验室设施（次级屏障）

●设施门要加锁，限制人员进入。

●实验设施地点离开公共区。

●每个实验室设一个洗手池。要求设置非手动或自动开关。

●实验室结构要便于清洁卫生，禁止使用地毯和垫子。

●工作台面不渗水，应耐酸、碱、耐热和有机溶剂等。

●实验室家具应预先设计，便于摆放和使用，表面应便于消毒，并在其间留有空隙便于清洁。

●生物安全柜的安装，室内的送、排风要符合物理防护参数要求。远离门口、风口和能开的窗户，远离室内人员经常走动的地方，远离其它可能干扰的仪器，以保证生物安全柜的气流参数和物理防护功能。

●建立冲洗眼睛的紧急救护点。

●照明适合于室内一切活动，避免反射和耀眼，以免干扰视线。

●只要求一般舒适空调，没有特殊通风要求。但是，新设施应该考虑机械通风系统能够提供通向室内的单向气流。如果有通向室外的窗户，必须安装纱窗。

7.4.3 三级生物安全实验室

指按照BSL-3标准建造的实验室，也称为生物安全实验室。实验室需与建筑物中的一般区域隔离。其具体标准微生物操作、特殊操作、安全设备、实验室设施要求如下。

7.4.3.1标准操作

●完成传染性材料操作后，对手套进行消毒冲洗，离开实验室之前，脱掉手套并洗手。

●设施内禁止吃、喝、抽烟，不准触摸隐形眼镜和使用化妆品。戴隐形眼镜的人也要佩戴防护镜或面罩。食物只能存放在工作区以外的地方。

●禁止用嘴吸取试验液体，要使用专用的移液管。

●一切操作均要小心，以减少和避免产生气溶胶。

●实验室卫生至少每天清洁一次，工作后随时消毒工作台面，传染性材料外溢、溅出污染时要立即消毒处理。

●所有培养物、储存物和其它日常废弃物在处理之前都要用高压灭菌器进行有效地灭菌处理。需要在实验室外面处理的材料，要装入牢固不漏的容器内，加盖密封后传出实验室。实验室的废弃物在送到处理地点之前应消毒、包装，避免污染环境。

●对BSL-3内操作的菌、毒种必须由两人保管，保存在安全可靠的设施内，使用前应办理批准手续，说明使用剂量，并详细登记，两人同时到场方能取出。试验要有详细使用和销毁记录。

●昆虫和啮齿类动物控制应参照其它有关规定执行。

7.4.3.2特殊操作

●制定安全细则

实验室负责人要根据实际情况制定本实验室特殊而全面的生物安全规则和具体的操作规程，以补充和细化本规范的操作要求，并报请生物安全委员会批准。工作人员必须了解细则，认真贯彻执行。

●生物危害标志

要在实验室入口的门上标记国际通用生物危害标志。实验室门口标记实验微生物种类、实验室负责人的名单和电话号码，指明进入本实验室的特殊要求，诸如需要免疫接种、佩戴防护面具或其它个人防护器具等。

实验室使用期间，谢绝无关人员参观。如参观必须经过批准并

在个体条件和防护达到要求时方能进入。

●生物危害警告

实验过程中实验室或物理防护设备里放有传染性材料或感染动物时，实验室的门必须保持紧闭，无关人员一律不得进入。

门口要示以危害警告标志，如挂红牌或文字说明实验的状态，禁止进入或靠近。

●进入实验室的条件

实验室负责人要指定、控制或禁止进入实验室的实验人员和辅助人员。

未成年人不允许进入实验室。

受感染概率增加或感染后果严重的实验室工作人员不允许进入实验室。

只有了解实验室潜在的生物危害和特殊要求并能遵守有关规定合乎条件的人才能进入实验室。

与工作无关的动植物和其它物品不允许带入实验室。

●工作人员的培训

对实验室工作人员和辅助人员要进行与工作有关的定期和不定期的生物安全防护专业培训。实验人员需经专门生物专业训练和生物安全训练，并由有经验的专家指导，或在生物安全委员会指导监督下工作。

必须学会气溶胶暴露危害的评价和预防方法。

在BSL-3实验室做传染性工作之前，实验室负责人要保证和证明，所有工作人员熟练掌握了微生物标准操作和特殊操作，熟练掌握本实验室设备、设施的特殊操作运转技术。包括操作致病因子和细胞培养的技能，或实验室负责人特殊培训的内容，或包括在安全微生物工作方面具有丰富经验的专家和安全委员会指导下规定的内容。

避免气溶胶暴露：一切传染性材料的操作不可直接暴露于空气之中，不能在开放的台面上和开放的容器内进行，都应在生物安全柜内或其它物理防护设备内进行。

需要保护人体和样品的操作可在室内排放式2A型生物安全柜内进行。

只保护人体不保护样品的操作可在Ⅰ级生物安全柜内进行。

如果操作带有放射性或化学性有害物时应在2B2型生物安全柜。

禁止使用超净工作台。

避免利器的感染：对可能污染的利器，包括针头、注射器、刀片、玻璃片、吸管、毛细吸管和解剖刀等，必须经常地采取高度有效的防范措施，必须预防经皮肤的实验室感染。

在BSL-3实验室工作，尽量不使用针头、注射器和其它锐利的器件。只有在必要时，如实质器官的注射、静脉切开、或从动物体内和瓶子（密封胶盖）里吸取液体时才能使用,尽量用塑料制品代替玻璃制品。

在注射和抽取传染性材料时，使用一次性注射器（针头与注射器一体的）。使用过的针头在消毒之前避免不必要的操作，如不可折弯、折断、破损，不要用手直接盖上原来的针头帽；要小心地把其放在固定方便且不会刺破的处理利器的容器里，然后进行高压消毒灭菌。

破损的玻璃不能用手直接操作，必须用机械的方法清除，如刷子、夹子和镊子等。

●污染的清除和消毒

传染性材料操作完成之后，实验室设备和工作台面应用有效的消毒剂进行常规消毒，特别是传染材料溢出、溅出其它污染，更要及时消毒。

溅出的传染性材料的消毒由适合的专业人员处理和清除，或由其它经过训练和有使用高浓度传染物工作经验的人处理。

一切废弃物处理之前都要高压灭菌，一切潜在的实验室污物（如，手套、工作服等）均需在处理或丢弃之前消毒。

需要修理、维护的仪器，在包装运输之前要进行消毒。

●感染性样品的储藏运输

一切感染性样品如培养物、组织材料和体液样品等在储藏、搬动、运输过程中都要放在不泄漏的容器内,容器外表面要彻底消毒，包装要有明显、牢固的标记。

●病原体痕迹的监测

采集所有实验室工作人员和其他有关人员的本底血清样品，进行病原体痕迹跟踪检测。依据被操作病原体和设施功能情况或实际中发生的事件，定期、不定期采集血清样本，进行特异性检测。

●医疗监督与保健

在BSL-3实验室工作期间对工作者进行医疗监督和保健，对于实验室操作的病原体，工作人员要接受相应的试验或免疫接种（如狂犬病疫苗，TB皮肤试验）。

●暴露事故的处理

当生物安全柜或实验室出现持续正压时，室内人员应立即停止操作并戴上防护面具，采取措施恢复负压。如不能及时恢复和保持负压，应停止实验，及早按规程退出。

发生此类事故或具有传染性暴露潜在危险的其它事故和污染，当事者除了采取紧急措施外，应立即向实验室负责人报告，听候指示，同时报告国家兽医实验室生物安全管理委员会。负责人和当事人应对其事故进行紧急科学、合理的处理。事后，当事人和负责人应提供切合实际的医学危害评价，进行医疗监督和预防治疗。

实验室负责人对事件的过程要予以调查和公布，写出书面报告呈报国家兽医实验室生物安全管理委员会同时抄报实验室安全委员会并保留备份。

7.4.3.3安全设备（初级防护屏障）

●防护服装

实验室内，工作人员要穿防护性实验服，如长服装、短套装，或有护胸的工作服装。消毒后清洗，如有明显的污染应及时换掉，作为污弃物处理。

在实验室外面不能穿工作服。

●防护手套

在操作传染性材料、感染动物和污染的仪器时必须戴手套，戴双层为好，必要时再戴上不易损坏的防护手套。

更换手套前，戴在手上消毒冲洗，一次性手套不得重复使用。

●生物安全柜

感染性材料的操作，如感染动物的解剖，组织培养、鸡胚接种、动物体液的收取等，都应在Ⅱ级以上生物安全柜内进行。

离心、粉碎、搅拌等不能在Ⅱ级生物安全柜内进行的工作可在较大或特制的Ⅰ级生物安全柜内进行。

●其它物理防护

当操作不能在生物安全柜内进行时，个人防护（Ⅲ级以上类似防护设备的具体要求）和其它物理防护设备（离心机安全帽，或密封离心机转头）并用。

●面部保护

污染区、半污染区应备有防护面具以便紧急使用，当房间内有感染动物时要戴面具保护。

建立紧急防护工作点。

●紧急防护用品

污染区或半污染区备用防护面具、冲洗眼睛的器具和药品等，随时可用。

7.4.3.4实验室设施（次级防护屏障）

BSL-3生物安全实验室里所有病原微生物的操作均在Ⅱ级以上（含Ⅱ级）生物安全柜内进行，其次级屏障标准如下：

●建筑结构和平面布局

建筑物抗震能力七级以上，防鼠、防虫、防盗。

实验室内净高应在2.6米以上，管道层净高宜不低于2.0米。

建筑物内实验室应与活动不受限制的公共区域隔开，设置安全门并安装门锁，禁止无关人员进入。

进入设施的通道设带闭门器的双扇门,其后是更衣室，分成一更室（清洁区）和二更室（半污染区），二更室后面为后室或称缓冲室（半污染区），进出缓冲室的门应为自动互锁。如果是多个实验室共用一个公用的走廊（或缓冲室），则进入每个实验室宜经过一个连锁的气闸（锁）门。

实验室应有安全通道和紧急出口，并有明显标识。

半污染区与清洁区之间必须设置传递窗。

洗刷室、机房等附属区域应是清洁区，但应尽量缩短与实验室的距离，方便工作。

实验室内可设密闭观察窗。

●密闭性和内表面

一切设施、设备外表无毛刺、无锐利棱角，尽量减少水平表面面积，便于清洁和消毒。

各种管道通过的孔洞必须密封。

墙和顶棚的表面要光滑，不刺眼、不积尘、不受化学物和常用消毒剂的腐蚀，无渗水、不凝集蒸气。

地表面应该是一体、防滑、耐磨、耐腐、不反光、不积尘、不漏水，如能按污染区划分给予颜色区别更好。

工作台面不能渗水，耐中等热、有机溶剂、酸、碱和常用消毒剂的损害和腐蚀。

实验室必要的桌椅橱柜等用具事先设计，便于稳妥安放和使用，彼此留有一定空间便于清洁卫生，表面消毒方便、耐腐。

●消毒灭菌设施

必须安装双扉式高压蒸汽灭菌器，安装在半污染区与洗刷室之间。灭菌器的两个门应互为连锁，灭菌器应满足生物安全二次灭菌要求。

污染区、半污染区的房间或传递窗内可安装紫外灯。

室内应配制人工或自动消毒器具（如消毒喷雾器、臭氧消毒器）并备有足够的消毒剂。

一切实验室内的废弃物都要分类集中装在可靠的容器内，都要在设施内进行消毒处理（高压、化学、焚化、其它处理），仪器的消毒选择适当的方法，如传递式臭氧消毒柜、环氧乙烷消毒袋等，如果废弃物需要传至实验室外，应该消毒后并装入密封容器、包装。

●净化空调

实验室污染区和半污染区采用负压单向流全新风净化空调系统。

污染区和半污染区不允许安装暖气、分体空调，不可用电风扇。

温度230C±20C、相对湿度40%—70%。

室内噪声不超过60分贝。

气流方向始终保证由清洁区流向污染区，由低污染区流向高污染区。空调系统应安装压力无关装置，以保证系统压力平衡，排风应采用一用一备自动切换系统。发生紧急情况时，应关闭送风系统，维持排风，保证实验室内安全负压。

供气需经HEPA过滤。排出的气体必须经过至少两级HEPA过滤排放，不允许在任何区域循环使用。

室内洁净度高于万级。

实验室送风口应在一侧的棚顶，出风口应在对面墙体的下部，尽量减少室内气流死角。保持单向气流，矢流方式较为合适。

实验室门口安装可视装置，能够确切表明进入实验室的气流方向。

Ⅱ级生物安全柜每年检测一次。2A型的排气可进入室内，2B2型安全柜和Ⅲ级安全柜的排风要通过实验室总排风系统排出。如果Ⅲ级安全柜是带有二次HEPA过滤、移动式，气流亦可在室内排气，但排气口应靠近室内排风口。

如有其它设备如液体消毒传递窗、药物熏蒸消毒器等的抽气系统，必须经过HEPA过滤，并根据需要更换。

●水的净化处理

每个房间出口附近设置一个非手动开关的洗手池。

污染区、半污染区和有可能被污染的供水管道应采取防止回流措施。如有下水，水池或地漏要设置消毒设施。下水下方必须设有水封，并始终充+盈消毒剂，水封的排气应加HEPA过滤装置。可能污染的下水只能排放到消毒装置内，消毒后再排至公共下水道。如没有下水排放，或不外排的所有废水均须收集并高压处理。洁净区域的下水可直接排入公共下水道。

●污染物和废弃物处理

对可能污染的物品和其它废弃物要放在专用的防止污染扩散或可消毒的容器里，以便消毒或高压灭菌处理。

● 实验室监控系统

应对实验室各种状态及设施全面设置监控报警点，构成完善的实验室安全报警系统。

●备用电源

非双路供电情况下，应配有备用电源，在停电时，至少能够保

证空调系统、警铃、灯光、进出控制和生物安全设备的工作。

●照明

照明应适合室内的一切活动，不反射、不刺眼，不影响视线。照明灯最好把灯具的部件装在顶棚里，或采取减少积尘措施。

●通讯

实验室内外应有适合的通讯联系设施（电话、传真、计算机等），

进行无纸化操作。

●验收和年检

BSL-3设施和运行必须是指令性的。

实验室的验收或年检应参考ISO10648标准检测方法进行密封性以保证维护结构的可靠性。

新建设施的功能必须检测验收，确认设计和运作参数合乎要求方能使用。

运行后每年再进行一次检测确认。

7.4.4. 四级生物安全实验室

指按照BSL-4标准建造的实验室，也称为高度实验室生物安全。实验室为独立的建筑物，或在建筑物内一切其它区域相隔离的可控制的区域。

为防止微生物传播和污染环境，BSL-4实验室必须实施特殊的设计和工艺。在此没有提到的BSL-3要求的各条款在BSL-4中都应做到。

其具体的标准微生物操作、特殊操作、安全设备和实验室设施要求如下：

7.4.4.1标准操作

●限制进入实验室的人员数量。

●制定安全操作利器的规程。

●减少或避免气溶胶发生。

●工作台面每天至少消毒一次，任何溅出物都要及时消毒。

●一切废弃物在处理前要高压灭菌。

●昆虫和啮齿类动物控制按有关规定执行。

●严格控制菌、毒种（见前）。

7.4.4.2特殊操作

●人员进入

只有工作需要的人员和设备运转需要的人员经过系统的生物安全培训，并经过批准后方能进入实验室。负责人或监督人有责任慎重处理每一个情况，确定进入实验室工作的人员。

采用门禁系统限制人员进入。

进入人员由实验室负责人、安全控制员管理。

人员进入前要告知他们潜在的生物危险，教会他们使用安全装置。

工作人员要遵守实验室进出程序。

制定应对紧急事件切实可行的对策和预案。

●危害警告 当实验室内有传染性材料或感染动物时，在所有的入口门上展示危险标志和普遍防御信号，说明微生物的种类、实验室负责人和其他责任人的名单和进入此区域特殊的要求。

●负责人职责 实验室负责人有责任保证，在BSL-4内工作之前，所有工作人员已经高度熟练掌握标准微生物操作技术、特殊操作和设施运转的特殊技能。这包括实验室负责人和具有丰富的安全微生物操作和工作经验专家培训时所提供的内容和安全委员会的要求。

●免疫接种 工作人员要接受试验病原体或实验室内潜在病原微生物的免疫注射。

●血清学监督 对实验室所有工作人员和其他有感染危险的人员采集本底血清并保存，再根据操作情况和实验室功能不定期血样采集。进行血清学监督。对致病微生物抗体评价方法要注意适用性。项目进行中，要保证每个阶段血清样本的检测，并把结果通知本人。

●安全手册 制定生物安全手册。告知工作人员特殊的生物危险，要求他们认真阅读并在实际工作当中严格执行。

●技术培训 工作人员必须经过操作最危险病原微生物的全面培训，建立普遍防御意识，学会对暴露危害的评价方法，学习物理防护设备和设施的设计原理和特点。每年训练一次，规程一旦修改要增加训练次数。由对这些病原微生物工作受过严格训练和具有丰富工作经验的专家或安全委员会指导、监督进行工作。

●紧急通道只有在紧急情况下才能经过气闸门进出实验室。实验室内要有紧急通道的明显标识。

●在安全柜型实验室中，工作人员的衣服在外更衣室脱下保存。穿上全套的实验服装（包括外衣、裤子、内衣或者连衣裤、鞋、手套）后进入。在离开实验室进入淋浴间之前，在内更衣室脱下实验服装。服装洗前应高压灭菌。在防护服型实验室中，工作人员必须穿正压防护服方可进入。离开时，必须进入消毒淋浴间消毒。

●实验材料和用品要通过双扉高压灭菌器、熏蒸消毒室或传递

窗送入，每次使用前后对这些传递室进行适当消毒。

●对利器，包括针头、注射器、玻璃片、吸管、毛吸管和解剖

刀，必须采取高度有效的防范措施。

尽量不使用针头、注射器和其它锐利的器具。只有在必要时，如实质器官的注射、静脉切开或从动物体内和瓶子里吸取液体时才能使用，尽量用塑料制品代替玻璃制品。

在注射和抽取传染性材料时，只能使用锁定针头的或一次性的注射器（针头与注射器一体的）。使用过的针头在处理之前，不能折弯、折断、破损，要精心操作，不要盖上原来的针头帽；放在固定方便且不会刺破的用于处理利器的容器里。不能处理的利器，必须放在器壁坚硬的容器内，运输到消毒区，高压消毒灭菌。

可以使用套管针管和套管针头、无针头注射器和其它安全器具。

破损的玻璃不能用手直接操作，必须用机械的方法清除，如刷子、簸萁、夹子和镊子。盛污染针头、锐利器具、碎玻璃等，在处理前一律消毒，消毒后处理按照国家或地方的有关规定实施。

●从BSL-4拿出活的或原封不动的材料时，先将其放在坚固密封的一级容器内，再密封在不能破损的二级容器里，经过消毒剂浸泡或消毒熏蒸后通过专用气闸取出。

●除活体或原封不动的生物材料以外的物品，除非经过消毒灭菌，否则不能从BSL-4拿出。不耐高热和蒸汽的器具物品可在专用消毒通道或小室内用熏蒸消毒。

●完成传染性材料工作之后，特别是有传染性材料溢出、溅出或污染时，都要严格彻底地灭菌。实验室内仪器要进行常规消毒。

●传染性材料溅出的消毒清洁工作，由适宜的专业人员进行。并将事故的经过在实验室内公示。

●建立报告实验室暴露事故、雇员缺勤制度和系统，以便对与实验室潜在危险相关的疾病进行医学监督。对该系统要建造一个病房或观察室，以便需要时，检疫、隔离、治疗与实验室相关的病人。

●与实验无关的物品（植物、动物和衣物）不许进入实验室。

7.4.4.3安全设备（初级防护屏障）

在设施污染和半污染工作区域内的一切操作都应在Ⅲ级生物安全柜内进行。如工作人员穿着具有生命支持通风系统的正压防护服，可在Ⅱ级生物安全柜内进行实验操作。

7.4.4.4实验室设施（次级防护屏障）

BSL-4实验室有两种类型：安全柜型，即所有病原微生物的操作均在Ⅲ级生物安全柜内或隔离器进行；防护服型，即工作人员穿正压防护服工作，操作可在Ⅱ级生物安全柜内进行。也可以在同一设施内穿正压防护服，并使用Ⅲ级生物安全柜。

●安全柜型

BSL-4建筑物或独立，或在系统建筑中由一个清洁区或隔墙把它与其它区域隔离开。

中心实验室（污染区）装有Ⅲ级生物安全柜，实验室周围为足够宽的隔离带，如环形走廊（半污染区）。从隔离带进出实验室必须通过一个缓冲间。

在污染区和半污染区之间，安装两台以上生物安全型高压蒸汽灭菌器（一次灭菌），互为备用。

外更衣室（清洁区）与内更衣室（半污染区）由淋浴间（清洁区）隔开，人员进出经过淋浴间。在清洁区与半污染区之间设置一个通风的双门传递通道，为不可通过更衣室进入实验室的实验材料、实验用品或仪器通过物理屏障时提供通道和消毒。在清洁区与半污染区之间同样安置一台生物安全型高压灭菌器，用于二次消毒。

每天工作开始之前，检查所有物理防护参数（如压差）。

实验区的墙、地和天棚整体密封，便于熏蒸消毒。内表面耐水和化学制剂、便于消毒。实验区任何液体必须排放到有消毒装置的储液罐，经过有效灭菌达标排放。通风口和在线管道都要安装HEPA过滤器。

工作台面不渗水，耐中等热、有机溶剂、酸、碱和常用消毒剂的腐蚀。

实验室用具事先设计，便于安放稳妥和使用，彼此留有一定空间便于清洁卫生，桌椅表面易于消毒。

内外更衣室和实验室进出门附近安装非手动或自动开关的洗手池。

排风经过2个串连的HEPA过滤，送、排风过滤器安装应便于消毒和更换。

供水、供气均安装防止回流的装置加以保护。

如果提供水源（消防喷枪），其开关应该是安装在实验室外面走廊里，开关自动或非手动。此系统与实验室区域供水分配系统分开，配备防止回流装置。

实验室进出门自动锁闭。

实验室内所有窗户都必须是封闭窗。

从Ⅲ级安全柜和实验室传出的材料必须经双扉高压灭菌器灭菌。灭菌器与周围物理屏障的墙之间要密封。灭菌器的门自动连锁控制，以保证只有在灭菌过程全部完成后才能开启外门。

从Ⅲ级安全柜或实验室内要拿出的材料和仪器，不能用高压灭菌消毒的要通过液体浸泡消毒、气体熏蒸消毒或同等效果的消毒装置进行消毒和传递。

来自内更衣室（包括厕所）和实验室内的洗手、地漏、高压灭菌器的废水以及其它废水，在排入公共下水之前，都要使用可靠的方法消毒（热处理比较合适）。淋浴和清洁区一侧厕所的废水不需特殊处理就可排入公共下水。所用废水消毒方法必须具有物理学和生物学的监测措施和法规确认。

非循环的负压通风系统，供、排风系统应采用压力无关装置保持动态平衡，保证气流从最低危险区向最高危险区的方向流动。对相邻区域的压差或气流方向进行监测，能进行系统声光报警。应安装一套能指示和确认实验室压差、适用而可视的气压监测装置，其显示部分安装在外更衣室的进口处。Ⅲ级生物安全柜与排风系统相连。

实验室的供排气都要经过HEPA过滤。为了缩短工作管道潜在的污染，HEPA尽可能安装在靠近工作的地方。所有HEPA每年均须检测一次，同时在靠近HEPA的地方应安装零泄露气密阀，便于过滤器安装与消毒更换。HEPA上游安装预过滤器可延长其使用寿命。

安全柜型生物安全水平Ⅳ级实验室的设计和操作程序是指令性的。实验室必须经过检测、鉴定和验收。只有合乎设计要求和运行标准的才能启用。实验室的验收或年检应参考ISO10648标准检测方法进行密封性测试，其检测压力不低于500Pa，半小时的小时泄漏率不超过10%，以保证维护结构的可靠性。实验室每年必须检测一次，确认合乎设计和运行参数的要求，才能继续运行。

实验室内外应有适合的通讯联系设施（电话、传真、计算机等），进行无纸化操作。

●防护服型

BSL-4建筑物独立，或在系统建筑中由一个清洁区或隔墙把它与建筑物其它区域隔开。

实验室房间的安排与安全柜型基本相同。不同的是在进入实验室（可用Ⅱ级生物安全柜代替Ⅲ级生物安全柜）之前要穿上有生命支持系统的正压防护服。生命支持系统所供气体应满足可呼吸空气生产标准，同时应增加紧急排风设施及配有备用电源。

进入BSL-4实验室之前要设置一个更衣和消毒区（设在实验室的一角或环形走廊内侧）。工作人员离开此区之前应在专用消毒室对防护服表面进行药物喷淋和熏蒸，时间不短于5分钟。

备用电源，在停电时应能够保证排风、生命支持系统、警铃、灯光、进出控制和生物安全柜的应急工作。

所有通向实验区、消毒淋浴室、气闸的空隙都要封闭。

每天实验开始之前，要完成对所有物理防护参数（如压差等）和正压防护服的检测，以保证实验室安全运行。

在实验区跨墙安装双扉高压灭菌器，对从实验区拿出的废弃物进行一次消毒。高压灭菌器与物理防护的壁板间要密闭。

设置渡槽、熏蒸消毒传递小室（柜），供不能通过更衣室进入实验区的实验材料、用品或仪器的消毒和传递使用。这些设施还能用于不能高压的材料、用品和仪器安全地取出。在清洁区与半污染区之间同样安置一台双扉生物安全型高压灭菌器，用于二次消毒

实验区的墙、地和天棚整体密封，便于熏蒸消毒。内表面耐水和化学制剂、便于消毒。实验区任何液体必须排放到有消毒装置的储液罐，经过有效灭菌达标排放。通风口和在线管道都要安装HEPA过滤器。

实验区内部附属设施，如灯的固定、空气管道、功能管道等的安排尽可能减少水平表面面积。

工作台面不渗水，中等耐热、抗有机溶剂、酸、碱和常用消毒剂的腐蚀。

实验用具要简单、分体、适用、牢固，不选用多孔材料。桌、柜、仪器之间保持一定空间，便于清洁和消毒。实验用椅和其它用具的表面应易于消毒。

实验区、内外更衣室的洗手池设非手动开关。

中央真空系统设在实验区内，在线HEPA过滤器靠近每一个使用点或开关。过滤器安装便于消毒和更换。其它进入实验区的供水、供气由防止回流装置加以控制。

实验区的门采用门禁系统。消毒淋浴、气闸室的内外门连锁。

来自污染区内的洗手池、地漏、灭菌器和其它来源的废水必须排放到有消毒装置的储液罐，经过有效灭菌达标排放。来自淋浴和厕所的废水经处理后排入下水道。所用的废水消毒方法的效果要有物理学和生物学的证据。

全新风通风系统。供、排风系统应采用压力无关装置保持动态平衡，保证气流从最低危险区向最高危险区的流动。对相邻区域的压差或气流方向进行监测，能进行系统声光报警。应安装一套能指示和确认实验室压差、适用而可视的气压监测装置，其显示部分安装在外更衣室的进口处。

实验区的供气要通过一个HEPA过滤处理，排气要通过串连的2个HEPA过滤处理。空气向高空排放，远离进气口。为了缩短工作管道潜在的污染，HEPA尽可能安装在靠近工作的地方。所有HEPA每年均须检测一次，同时在靠近HEPA的地方应安装零泄露气密阀，便于过滤器安装与消毒更换。HEPA上游安装预过滤器可延长其使用寿命。

防护服型生物安全Ⅳ级实验室设计和运转要求是指令性的。实验室必须经过检测、鉴定和验收。只有合乎设计要求和运行标准的才能启用。实验室的验收或年检应参考ISO10648标准检测方法进行密封性测试，其检测压力不低于500Pa，半小时内的小时泄漏率不超过10%，以保证维护结构的可靠性。实验室每年必须检测一次，确认合乎设计和运行参数的要求，才能继续运行。

实验室内外应有适合的通讯联系设施（电话、传真、计算机等），进行无纸化操作。

8 动物实验生物安全水平标准

8.1动物实验生物安全实验室分级：

动物实验安全实验室分4级，所配备的动物设施、设备和操作分别适用于生物安全Ⅰ-Ⅳ级的病原微生物感染动物的工作，安全水平逐级提高。

8.2 各级动物生物安全实验室的要求

8.2.1一级动物实验生物安全实验室

指按照ABSL-1标准建造的实验室，也称动物实验基础实验室。

8.2.1.1标准操作

●动物实验室工作人员需经专业培训才能进入实验室。人员进入前,要熟知工作中潜在的危险,并由熟练的安全员指导。

●动物实验室要有适当的医疗监督措施。

●制定安全手册，工作人员要认真贯彻执行，知悉特殊危险

●在动物实验室内不允许吃、喝、抽烟、处理隐形眼镜和使用化妆品、储藏食品等。

●所有实验操作过程均须十分小心,以减少气溶胶的产生和外溢。

●实验中，病原微生物意外溢出及其它污染时要及时消毒处理。

●从动物室取出的所有废弃物,包括动物组织、尸体、垫料,都要放入防漏带盖的容器内,并焚烧或做其它无害化处理，焚烧要合乎环保要求。

●对锋利物要制定安全对策。

●工作人员在操作培养物和动物以后要洗手消毒,离开动物设施之前脱去手套、洗手。

●在动物实验室入口处都要设置生物安全标志，写明病原体名称、动物实验室负责人及其电话号码,指出进入本动物实验室的特殊要求(如需要免疫接种和呼吸道防护)。

8.2.1.2特殊操作 无。

8.2.1.3安全设备(初级防护屏障)

●工作人员在设施内应穿实验室工作服。

●与非人灵长类动物接触时应考虑其粘膜暴露对人的感染危险,要戴保护眼镜和面部防护器具。

●不要使用净化工作台，需要时使用Ⅰ级或2A型生物安全柜。

8.2.1.4设施(次级防护屏障)

●建筑物内动物设施与人员活动不受限制的开放区域用物理屏障分开。

●外面门自关自锁，通向动物室的门向内开并自关,当有实验动物时保持关闭状态，大房间内的小室门可向外开,为水平或垂直滑动拉门。

●动物设施设计防虫、防鼠、防尘，易于保持室内整洁。内表面(墙、地板和天棚)要防水、耐腐蚀。

●内部设施的附属装置,如灯的固定附件、风管和功能管道排列整齐并尽可能减少水平表面。

●建议不设窗户, 如果动物设施内有窗户并需开启,必须安纱窗。所有窗户必须牢固,不易破裂。

●如果有地漏都要始终用水或消毒剂充满水封。

●排风不循环。建议动物室与邻室保持负压.

●动物室门口设有一个洗手水槽。

●人工或机器洗涤动物笼子,最终洗涤温度至少达到82℃。

●照明要适合所有的活动,不反射耀眼以免影响视觉。

8.2.2 二级动物实验生物安全实验室

指按照ABSL-2标准建造的动物实验室。

8.2.2.1标准操作

●设施制度除了制定紧急情况下的标准安全对策、操作程序和规章制度外,还应依据实际需要制定特殊的对策。把特殊危险告知每位工作人员,要求他们认真贯彻执行安全规程。

●尽可能减少非熟练的新成员进入动物室。为了工作或服务必须进入者,要告知其工作潜在的危险。

●动物实验室应有合适的医疗监督,根据试验微生物或潜在微生物的危害程度，决定是否对实验人员进行免疫接种或检验(例如狂犬病疫苗和TB皮试)。如有必要,应该实施血清监测。

●在动物室内不允许吃、喝、抽烟、处理隐形眼镜和使用化妆品、储藏个人食品。

●所有实验操作过程均须十分小心,以减少气溶胶的产生和防止外溢。

●操作传染性材料以后所有设备表面和工作表面用有效的消毒剂进行常规消毒,特别是有感染因子外溢,和其它污染时更要严格消毒。

●所有样品收集放在密闭的容器内并贴标签,避免外漏。所有动物室的废弃物(包括动物尸体、组织、污染的垫料、剩下的饲料、锐利物和其它垃圾)应放入密闭的容器内，高压蒸汽灭菌,然后建议焚烧。焚烧地点应是远离城市、人员稀少、易于空气扩散的地方。

●对锐利物的安全操作（见前面所述）。

●工作人员操作培养物和动物以后要洗手,离开设施之前脱掉手套并洗手。

●当动物室内操作病原微生物时,在入口处必须有生物危害的标志。危害标志应说明使用感染病原微生物的种类,负责人的名单和电话号码。特别要指出对进入动物室人员的特殊要求(如免疫接种和面罩)。

●严格执行菌（毒）种保管制度。

8.2.2.2特殊操作

●对动物管理人员和试验人员应进行与工作有关的专业技术培训,必须避免微生物暴露,了解评价暴露的方法。每年定期培训, 保存培训记录，当安全规程和方法变化时要进行培训。一般来讲,感染危险可能性增加的人和感染后果可能严重的人不允许进入动物设施,除非有办法除去这种危险。

●只允许用做实验的动物进入动物实验室。

●所有设备拿出动物室之前必须消毒。

●造成明显病原微生物暴露的实验材料外溢事故,必须立刻妥善处理并向设施负责人报告，及时进行医学评价、监督和治疗，并保留记录。

8.2.2.3安全设备(初级防护屏障)

●动物室内工作人员穿工作服。在离开动物实验室时脱去工作服。在操作感染动物和传染性材料时要戴手套。

●在评价认定危害的基础上使用个人防护器具。在室内有传染性非人灵长类动物时要戴防护面罩。

●进行容易产生高危险气溶胶的操作时, 包括对感染动物和鸡胚的尸体、体液的收集和动物鼻腔接种，都要同时使用生物安全柜或其它物理防护设备和个人防护器具(例如口罩和面罩)。

●必要时,把感染动物饲养在和动物种类相宜的一级生物安全设施里。建议鼠类实验使用带过滤帽的动物笼具。

8.2.2.4设施(次级防护屏障)

●建筑物内动物设施与开放的人员活动区分开。

●进入设施要经过牢固的气闸门，其外门自关自锁。进入动物室的门应自动关闭,有实验动物时要关紧。

●设施结构易于保持清洁,内表面(墙、地板和天棚)防水、耐腐。

●设施内部附属装置,如灯架、气道、功能管道尽可能整齐并减少水平表面积。

●一般不设窗户，如有窗户必须牢固并设纱窗。

●如果有地漏,管道水封始终充满消毒液。

●人工或冲洗器洗刷动物笼子,冲洗最终温度至少82℃。

●设施内传染性废弃物要高压灭菌。

●在感染动物室内和设施其它地方安装一个洗手池。

●照明要适合于所有室内活动,不反射耀眼。

8.2.3 三级动物实验生物安全实验室

指按照ABSL-3标准建造的实验室，适合于具有气溶胶传播潜在危害和引起致死性疾病的微生物感染动物的工作。

8.2.3.1标准操作

●制定安全手册或手册草案。除了制定紧急情况下的标准安全对策、操作程序和规章制度,还应根据实际需要制定特殊适用的对策。

●限制对工作不熟悉的人员进入动物室。为了工作或服务必须进入者,要告知他们工作中潜在的危险。

●动物室应有合适的医疗监督,根据试验微生物或潜在微生物的危害程度，决定是否对实验人员进行免疫接种或检验(例如狂犬病疫苗和TB皮试)。如有必要,应该实施血清监测。

●不允许在动物室内吃、喝、抽烟、处理隐形眼镜和使用化妆品、储藏人的食品。

●所有实验操作过程均须十分小心,以减少气溶胶的产生和防止外溢。

●操作传染性材料以后所有设备表面和工作台面用适当的消毒剂进行常规消毒,特别是有传染性材料外溢和其它污染时更要严格消毒。

●所有动物室的废弃物(包括动物组织、尸体、污染的垫料、动物饲料、锐利物和其它垃圾)放入密闭的容器内并加盖，容器外表面消毒后进行高压蒸汽灭菌,然后建议焚烧。焚烧要合乎环保要求。

●对锐利物进行安全操作。

●工作人员操作培养物和动物以后要洗手,离开设施之前脱掉手套、洗手。

●动物室的入口处必须有生物危害的标志。危害标志应说明使用病原微生物的种类,负责人的名单和电话号码，特别要指出对进入动物室人员的特殊要求(如免疫接种和面罩)。

●所有收集的样品应贴上标签,放在能防止微生物传播的传递容器内。

●实验和实验辅助人员要经过与工作有关的潜在危害防护的针对性培训。

●建立评估暴露的方法,避免暴露。

●对工作人员进行专业培训,所有培训记录要归档。

●严格执行菌（毒）种保管和使用制度。

8.2.3.2特殊操作

●用过的动物笼具清洗拿出之前要高压蒸汽灭菌或用其它方法消毒。设施内仪器设备拿出检修打包之前必须消毒。

●实验材料发生了外溢,要消毒打扫干净。如果发生传染性材料的暴露必须立刻向设施负责人报告,同时报国家兽医实验室生物安全管理委员会，最后的处理评估报告，也要及时报国家兽医实验室生物安全管理委员会，同时报实验室生物安全委员会回负责人。及时提供正确医疗评价、医疗监督和处理并保存记录。

●所有的动物室内废弃物在焚烧或进行其它最终处理之前必须高压灭菌。

●与实验无关的物品和生物体不允许带入动物实验室。

8.2.3.3安全设备(初级防护屏障)

●在危害评估确认的基础上使用个人防护器具。操作传染性材料和感染动物都要使用个体防护器具。工作人员进入动物实验室前要按规定穿戴工作服,再穿特殊防护服。不得穿前开口的工作服。离开动物室前必须脱掉工作服，并进行适合的包装，消毒后清洗。

●操作感染动物时要戴手套,实验后以正确方式脱掉,在处理之前和动物实验室其它废弃物一同高压灭菌。

●将感染动物饲养放在Ⅱ级生物安全设备中（如负压隔离器）。

●操作具有产生气溶胶危害的感染动物和鸡胚的尸体、收取的组织和体液，或鼻腔接种动物时，应该使用Ⅱ级以上生物安全柜，戴口罩或面具。

8.2.3.4设施(次级防护屏障)

三级动物生物安全实验室的感染动物在Ⅱ级或Ⅱ级以上生物安全设备中（如负压隔离器）饲养，所有操作均在Ⅱ级或Ⅱ级以上生物安全柜内进行，其次级屏障标准如下：

●建筑物中的动物设施与人员活动区分开。

●进入设施的门要安装闭门器。外门可由门禁系统控制。进入后为一更室（清洁区），其后是二更室（半污染区）。传递窗（室）和双扉高压灭菌器设置在清洁区与半污染区之间，为实验用品、设备和废弃物进出设施提供安全通道。从二更室进入动物室（污染区）经过自动互连锁门的缓冲室,进入动物房的门要向外开。

●设施的设计、结构要便于打扫和保持卫生。内表面(墙、地板、天棚)应防水、耐腐。穿过墙、地板和天棚物件的穿孔要密封,管道开口周围要密封,门和门框间也要密封。

●每个动物室靠近出口处设置一个非手动洗手池,每次使用后洗手池水封处用适合的消毒剂充满。

●设施内的附属配件,如灯架、气道和功能管道排列尽可能整齐、减小水平表面。

●所有窗户都要牢固和密封。

●所有地漏的水封始终充以适当的消毒剂。

●气流方向始终保证由清洁区流向污染区，由低污染区流向高污染区。空调系统应安装压力无关装置，以保证系统压力平衡，排风应采用一用一备自动切换系统。发生紧急情况时，应关闭送风系统，维持排风，保证实验室内安全负压。

●供气需经HEPA过滤。排出的气体必须经过两级HEPA过滤排放，不允许在任何区域循环使用。

室内洁净度高于万级。

实验室实验室送风口应在一侧的棚顶，出风口应在对面墙体的下部，尽量减少室内气流死角。保持单向气流，矢流方式较为合适。

实验室门口安装可视装置，能够确切表明进入实验室的气流方向。

Ⅱ级生物安全柜每年检测一次。2A型的排气可进入室内，2B2型安全柜和Ⅲ级安全柜的排风要通过实验室总排风系统排出。如果Ⅲ级安全柜是带有二次HEPA过滤、移动式，气流亦可在室内自循环。

●动物笼在洗刷池内清洗,如用机器清洗最终温度达到82℃。

●感染性废弃物从设施拿出之前必须高压灭菌。

●有真空(抽气)管道(中心或局部)的,每一个管道连接应该安装液体消毒罐和HEPA,安装在靠近使用点或靠近开关处。过滤器安装应易于消毒更换。

●照明要适应所有的活动,不反射耀眼,以免影响视觉。

●上述的3级生物安全设施和操作程序是强制性规定。

实验室的验收或年检应参考ISO10648标准检测方法进行密封性测试，其检测压力不低于250Pa，半小时的小时泄漏率不超过10%，以保证维护结构的可靠性。

新建设施的功能必须检测验收，确认设计和运作参数合乎要求方能使用。

运行后每年进行一次检测确认。

8.2.4 四级动物实验生物安全实验室

指按照ABSL-4标准建造的实验室，适用于本国和外来的、通过气溶胶传播或不知其传播途径的、引起致死性疾病的高度危害病原体的操作。必须使用Ⅲ级生物安全柜系列的特殊操作和正压防护服的操作。

8.2.4.1标准操作

●应该制定特殊的生物安全手册或措施。除了制定紧急情况下的对策、程序和草案外,还要制定适当的针对性对策。

●未经培训的人员不得进入动物实验室。因为工作或实验必须进入者,应对其说明工作的潜在危害。

●所有进入ABSL-4设施的人必须建立医疗监督,监督项目必须包括适当免疫接种、血清收集及暴露危险等有效性协议和潜在危害预防措施。一般而言,感染危险性增加者或感染后果可能严重的人不允许进入动物设施,除非有特殊办法能避免额外危险。这应由专业保健医师做出评价。

●负责人要告知工作人员工作中特殊的危险,让他们熟读安全规程并遵照执行。

●设施内禁止吃、喝、抽烟、处理隐形眼镜、使用化妆品和储藏食品。

●所有操作均须小心,尽量减少气溶胶的产生和外溢。

●传染性工作完成之后,工作台面和仪器表面要用有效的消毒液进行常规消毒,特别是有传染性材料溢出和溅出或其它污染时更要严格消毒。

●外溢污染一旦发生,应由具有从事传染性实验工作训练和有经验的人处理。外溢事故明显造成传染性材料暴露时要立即向设施负责人报告，同时报国家兽医实验室生物安全管理委员会，最后的处理评估报告，也要及时报国家兽医实验室生物安全管理委员会，同时报实验室生物安全委员会回负责人。及时提供正确医疗评价、医疗监督和处理并保存记录。

●全部废弃物(含动物组织、尸体和污染垫料)、其它处理物和需要洗的衣服均需用安装在次级屏障墙壁上的双扉高压蒸汽灭菌器消毒。废弃物要焚烧。

●要制定使用利器的安全对策。

●传染性材料存在时,设施进口处标示生物安全符号,标明病原微生物的种类、实验室负责人的名单和电话号码,说明对进入者的特殊要求(如免疫接种和呼吸道防护)。

●动物实验室工作人员要接受与工作有关的潜在危害的防护培训,懂得避免暴露的措施和暴露评估的方法。每年定期培训,操作程序发生变化时还要增加培训，所有培训都要记录、归档。

●动物笼具在清洗和拿出动物实验室之前要进行高压灭菌或用其它可靠方法消毒。用传染性材料工作之后，对工作台面和仪器应用适当的消毒剂进行常规消毒。特别是传染材料外溅时更要严格消毒。仪器修理和维修拿出之前必须消毒。

●进行传染性实验必须指派2名以上的实验人员。在危害评估的基础上,使用能关紧的笼具,操作动物要对动物麻醉,或者用其它的方法,必须尽可能减少工作中感染因子的暴露。

●与实验无关的材料不许进入动物实验室。

●严格执行菌（毒）种保管和使用制度。

8.2.4.2特殊操作

●必须控制人员进入或靠近设施(24小时监视和登记进出)。人员进出只能经过更衣室和淋浴间,每一次离开设施都要淋浴。除非紧急情况,不得经过气锁门离开设施。

●在安全柜型实验室中，工作人员的衣服在外更衣室脱下保存。穿上全套的实验服装（包括外衣、裤子、内衣或者连衣裤、鞋、手套）后进入。在离开实验室进入淋浴间之前，在内更衣室脱下实验服装。服装洗前应高压灭菌。在防护服型实验室中，工作人员必须穿正压防护服方可进入。离开时，必须进入消毒淋浴间消毒。

●进入设施的实验用品和材料要通过双扉高压锅或传递消毒室。高压灭菌器应双门互连锁，不排蒸汽，冷凝水自动回收灭菌，避免外门处于开启状态。

●建立事故、差错、暴露、雇员缺勤报告制度和动物实验室有关潜在疾病的医疗监督系统，这个系统要附加以潜在的和已知的与动物实验室有关疾病的检疫、隔离和医学治疗设施。

●定期收集血清样品进行检测并把结果通知本人。

8.2.4.3安全设备(初级防护屏障)

●在安全柜型实验室中，感染动物均在Ⅲ级生物安全设备中（如手套箱型隔离器）饲养，所有操作均在Ⅲ级生物安全柜内进行，并配备相应传递和消毒设施。在防护服型实验室中，工作人员必须穿正压防护服方可进入。感染动物可饲养在局部物理防护系统中(如把开放的笼子放在负压层流柜或负压隔离器中)，操作可在Ⅱ级生物安全柜内进行。

●重复使用的物品,包括动物笼在拿出设施前必须消毒。废弃物拿出设施之前必须高压消毒,然后焚烧。焚烧应符合环保要求。

8.2.4.4设施(次级防护屏障)

●ABSL-4与BSL-4的设施要求基本相同,两者必须紧密结合在一起进行统一考虑,或者说,与前面讨论的规定(安全实验室) 相匹配。本节没有提到的均应按Ⅳ级生物安全水平要求执行。

●动物饲养方法要保证动物气溶胶经过高效过滤净化后方可排放至室外，不能进入室内。

●一般情况，操作感染动物，包括接种、取血、解剖、更换垫料、传递等，都要在物理防护条件下进行。能在Ⅲ级安全柜内进行的必须在其内操作。

●根据实验动物的大小、数量，要特殊设计感染动物的消毒和处理设施，保证不危害人员、不污染环境。污染区与半污染区之间的灭菌器（一次灭菌）安装位置、数量和方法见“Ⅳ级生物安全水平”部分。此外，在半污染区与清洁区之间的再安装一台双扉高压蒸汽灭菌器（二次病菌），以便灭菌其他污染物，必要时进行再次高压灭菌。

●特殊情况，不能在Ⅲ级安全柜内饲养的大动物或动物数量较多时，动物实验室要根据情况特殊设计。

确定动物实验室容积，结构密闭合乎要求，设连锁的气闸门。

要有足够的换气次数，负压过滤通风采用矢流方式，避免死角。

高压灭菌的尸体可经二次灭菌传出，亦可密闭包装、表面消毒通过设置在污染区与清洁区之后的气闸门送出、焚烧。

实验室的验收或年检应参考ISO10648标准检测方法进行密封性测试，其检测压力不低于500Pa，半小时的小时泄漏率不超过10%，，以保证维护结构的可靠性。实验室每年必须检测一次，确认合乎设计和运行参数的要求，才能继续运行。

实验室内外应有适合的通讯联系设施（电话、传真、计算机等），进行无纸化操作。

9 生物危害标志及使用

9.1 生物危害标志

9.2 生物危害标志的使用

9.2.1 在BSL-2 / ABSL-2 级兽医生物安全实验室入口的明显位置必须粘贴标有危险级别的生物危害标志。

9.2.2 在BSL-3 / ABSL-3 级及以上级别兽医生物安全实验室所在的建筑物入口、实验室入口及操作间均必须粘贴标有危害级别的生物危害标志，同时应标明正在操作的病原微生物种类。

9.2.3 凡是盛装生物危害物质的容器、运输工具、进行生物危险物质操作的仪器和专用设备等都必须粘贴标有相应危害级别的生物危害标志。

1. 国家卫生健康委员会：卫生部关于进一步做好病原微生物实验室生物安全管理工作的通知

卫科教发〔2006〕352号

一、进一步提高对实验室生物安全工作重要性的认识

病原微生物实验室生物安全工作是关系到实验室工作人员和社会公众健康的大事，各相关部门、机构和从业人员都要进一步提高对于这项工作的认识。各级卫生行政部门要加强对实验室生物安全工作的监督管理，严格按照国家的法规和规章办事，确保实验室生物安全。同时，要正确处理好业务工作与依法行政的关系，确保重大传染病防治工作有效进行。

应积极利用各种形式进行实验室生物安全的宣传工作，要认真做好生物安全实验室工作人员的培训和考核工作，在生物安全实验室从事高致病性病原微生物实验活动的工作人员要持证上岗。

各级疾病预防控制机构、医疗机构、教育机构、科研机构及各级各类相关机构要组织工作人员认真学习有关的法律法规、技术标准和业务知识，不断提高对实验室生物安全的认识和能力。

二、加强实验室生物安全管理的制度建设

各省级卫生主管部门要根据《条例》和卫生部相关文件的要求，制定本地区贯彻执行的配套文件和管理规定。近期要抓紧制定本辖区内开展高致病性病原微生物实验活动审批及监督管理、运输可感染人类的高致病性病原微生物菌(毒)种或样本管理以及生物安全二级实验室监督管理等相关规定。根据《条例》和卫生部有关文件的要求，除在生物安全四级实验室从事高致病性病原微生物实验活动外，其它高致病性病原微生物实验活动均由省级卫生行政部门审批。卫生行政部门要本着服务基层、依法行政的理念，严格按照审批权限、程序、条件和时限，认真履行职责。各级卫生主管部门要依据《条例》赋予的职责，认真做好实验室生物安全的安全管理工作。

从事病原微生物实验活动的实验室要建立健全实验室生物安全管理体系和管理程序，并把各项管理要求落实到日常工作中去。要强化实验室生物安全责任制和责任追究制度。

各级主管部门和卫生机构要关心和爱护从事病原微生物实验室工作的人员，努力为他们创造良好的工作条件和安全的工作环境。

三、认真履行监督管理职责

监督管理是确保实验室生物安全的重要环节，也是《条例》赋予卫生行政部门的一项重要职责。各级卫生行政部门要根据属地化管理的原则，全面了解本辖区病原微生物实验室的基本情况及生物安全工作的状况，加强对于高致病性病原微生物标本采集、运输、保藏、实验活动、废弃物处理的监管，重点是高等级生物安全实验室实验活动及高致病性病原微生物菌(毒)种及样本保藏的监管。对于未获得实验室活动资格或未经批准进行高致病性病原微生物实验活动的，以及其它违反《条例》的行为，要依法进行查处。

四、加强实验室生物安全条件建设

从事高致病性病原微生物实验活动的机构应当设立与所从事工作相适应的生物安全实验室。各地、各单位要制定生物安全实验室建设发展规划，加大投入，力争早日改善实验室生物安全条件。近日，国家发改委发布了《关于认定第一批国家高级别生物安全实验室的通知》(发改高技 [2006]1587号)，确定了第一批列入国家高等级生物安全实验室建设规划的实验室名单，各地、各单位要认真加以落实。特别是列入规划的省级疾病预防控制机构的生物安全三级实验室，要抓紧建设，以保障疾病预防控制工作的有效开展。各级主管部门和单位要重视生物安全实验室国家认可工作，对列入规划并已建成的高等级生物安全实验室，要抓紧申请国家实验室认可。

各级卫生行政部门要按照《条例》、我部配套文件和本通知的要求，近期内对辖区内从事病原微生物实验活动的机构组织一次监督检查。检查的主要内容是相关法律法规、部门规章的执行情况，要着重检查辖区内相关实验室在可感染人的高致病性病原微生物及其样本的采集、运输、保藏、使用和管理方面存在的问题。请于2006年10月底前将检查结果报我部科教司。我部将于年内再组织一次病原微生物实验室生物安全管理专项检查。

1. 科学技术部：关于发布《高等级病原微生物实验室建设审查行政审批事项服务指南》的通知

一、适用范围

本指南适用于高等级病原微生物实验室（以下简称实验室）建设（审批事项）的申请和办理，包括新建、改建、扩建实验室或者生产、进口移动式实验室。

二、项目信息

（一）项目名称：高等级病原微生物实验室建设审查

（二）审批类别：行政许可

（三）项目编码：03001

三、办理依据

（一）《行政许可法》

（二）《病原微生物实验室生物安全管理条例》（国务院令第424号）第十九条：“新建、改建、扩建三级、四级实验室或者生产、进口移动式三级、四级实验室应当经国务院科技主管部门审查同意。”

（三）《高等级病原微生物实验室建设审查办法》（科技部令第15号）第二条：“新建、改建、扩建高等级病原微生物实验室或者生产、进口移动式高等级病原微生物实验室（以下简称建设实验室）应当报科学技术部审查同意。”

四、受理机构

科技部

五、决定机构

科技部

六、审批数量

无数量限制。

七、办事条件

（一）申请人条件

具有法人资格的企事业单位。

（二）具备或符合如下条件的，准予批准：

1.实验室已纳入国家生物安全实验室体系规划；

2.实验室对于开展相关实验活动确属必要；

3.实验室具有从事相关实验活动的职能和工作基础；

4.实验室具有规范的运行管理制度；

5.实验室具有相应的设施设备和专业人才队伍；

6.申请材料及其报送程序符合规定；

7.法律法规规定的其他条件。

（三）有如下情形之一的，不予批准：

1.实验室未纳入国家生物安全实验室体系规划；

2.实验室不具有开展相关实验活动的工作任务；

3.实验室不具有从事相关实验活动的职能和工作基础；

4.实验室不具有规范的运行管理制度；

5.实验室不具有相应的设施设备和专业人才队伍；

6.申请材料及其报送程序不符合规定；

7.法律法规规定的其他情形。

八、申请材料

（一）申请材料清单

序号 提交材料名称 原件/复印件 份数 纸质/电子 要求 备注

1 高等级病原微生物实验室建设审查申请书 原件、复印件 纸质材料一式13份，其中至少2份原件，电子版材料1份 纸质材料、电子版 A4规格纸张打印，中文使用宋体4号字，英文使用Times New Roman 12号字 电子版刻录光盘报送

申请书请在科技部网站“办事指南”专栏下载（网址：http://www.most.gov.cn/fggw/bmgz/201107/t20110727_88574.htm）。申请书示范文本以及常见错误事例见附录A。

（二）申请材料提交

申请人可通过窗口报送或邮寄方式提交材料：

1.窗口报送：科技部行政审批受理窗口（地址：北京市海淀区西四环中路16号院4号楼，科技部中国生物技术发展中心）；

2.邮寄报送：科技部行政审批受理窗口（地址：北京市海淀区西四环中路16号院4号楼，科技部中国生物技术发展中心；邮编：100039；电话：010-88225153）。

九、申请接收

（一）接收方式

1.窗口接收：科技部行政审批受理窗口（地址：北京市海淀区西四环中路16号院4号楼，科技部中国生物技术发展中心）。

2.信函接收：科技部行政审批受理窗口（地址：北京市海淀区西四环中路16号院4号楼，科技部中国生物技术发展中心；邮编：100039；电话：010-88225153）。

（二）办公时间

工作日8:00-11:30，13:30-17:00

十、办理基本流程

十一、办理方式

本行政许可按照一般程序办理，包括申请、受理、技术评审、决定和文书送达等。

（一）申请

申请单位经其所在地省级人民政府或按照业务隶属关系经国务院有关部门（以下简称申请单位省级主管部门）向科技部提交申请材料。

（二）申请单位省级主管部门签署意见

申请单位省级主管部门在收到申请人提交的材料后，签署意见，并于10个工作日内将申请材料经本部门盖章及负责人签章后函报科技部。

（三）形式审查与受理

科技部在收到申请单位省级主管部门函报的相关材料后，在5个工作日内完成形式审查，对申请材料齐全、符合规定形式的申请，予以受理并出具受理单。申请材料不齐全或不符合规定形式的，在5个工作日内一次性书面告知申请单位省级主管部门和申请单位需要补正的全部内容。

（四）审查委员会审查

科技部从高等级病原微生物实验室生物安全审查委员会抽取专家对受理的申请进行会议或现场审查，形成审查委员会审查建议。

（五）部务会研究

科技部根据审查委员会审查建议，经部务会研究形成审批决定。

（六）文件制作与送达

根据部务会审批决定，科技部编制回复申请单位省级主管部门有关审查意见的函。自行政许可决定作出之日起10个工作日内，科技部将审批函送达申请单位省级主管部门和申请单位。

（七）复核、复议与诉讼

申请单位对以上决定有异议的，可以申请复核、复议或诉讼。可在收到通知之日起15个工作日内经其省级主管部门以书面形式向科技部申请复核，也可在收到通知之日起60日内向科技部申请行政复议，也可在收到通知之日起6个月内向人民法院提起诉讼。

（八）结果公开

审批决定不予公开。

十二、审批时限

自受理之日起10个工作日内办结，特殊情况延长10个工作日。办理过程中所需的专家评审时间不计入时限。

十三、审批收费依据及标准

根据《高等级病原微生物实验室建设审查办法》第十五条，本审批事项不收费。

十四、审批结果

科技部关于高等级病原微生物实验室建设申请审查意见的函（样式见附录B）。

十五、结果送达

作出行政许可决定后10个工作日内，科技部通过电话或短信方式告知申请单位省级主管部门和申请单位，并通过现场领取或邮寄方式将审批函送达申请单位省级主管部门和申请单位。

十六、申请人权利和义务

（一）依据《中华人民共和国行政许可法》、《高等级病原微生物实验室建设审查办法》等，申请人依法享有以下权利：

1.对行政机关实施行政许可，享有陈述权、申辩权；其合法权益因行政机关违法实施行政许可受到损害的，有权依法要求赔偿。

2.申请人对实施行政许可行为有异议的，可自收到通知之日起15个工作日内经其省级主管部门以书面形式向科技部申请复核；或者自收到通知之日起60日内向科技部申请行政复议；或者自收到通知之日起6个月内向人民法院提起诉讼。

（二）依据《中华人民共和国行政许可法》、《高等级病原微生物实验室建设审查办法》等，申请人依法履行以下义务：

1.申请人申请行政许可，应当如实向行政机关提交有关材料和反映真实情况，并对其申请材料实质内容的真实性负责。

2.申请人在实验室建设审查过程中存在弄虚作假等行为的，科技部将终止对其申请的审查或撤销已作出的审批决定，书面通知其省级主管部门，并根据情节轻重决定在1-3年内不再受理其申请。

十七、咨询途径

（一）窗口咨询：科技部行政审批受理窗口（地址：北京市海淀区西四环中路16号院4号楼，科技部中国生物技术发展中心）；

（二）电话咨询： 010-58881423 010-88225153；

（三）网上咨询：http://appweblogic.most.gov.cn/gzwd/gzwd_jsjg.jsp；

（四）电子邮件咨询：sfs_zhc@most.cn；

（五）信函咨询：科技部社会发展科技司（地址：北京市海淀区复兴路乙15号，科技部社会发展科技司；邮编：100862）。

十八、监督和投诉渠道

（一）窗口投诉：科技部政策法规与监督司（地址：北京市海淀区复兴路乙15号）；

（二）电话投诉：科技部政策法规与监督司

010-58881765；

（三）网上投诉：http://appweblogic.most.gov.cn/gzwd/gzwd_jsjg.jsp；

（四）电子邮件投诉：zhengj@most.cn；

（五）信函投诉：科技部政策法规与监督司（地址：北京市海淀区复兴路乙15号；邮编：100862）。

1. 国家卫生健康委员会：卫生部关于印发《高致病性病原微生物实验室资格审批工作程序》的通知

第一章 总则

第一条为确保高致病性病原微生物实验室资格审批（以下简称资格审批）工作的公正、公开、公平，依据《病原微生物实验室生物安全管理条例》和《人间传染的高致病性病原微生物实验室和实验活动生物安全审批管理办法》（以下简称《办法 》）等，制定本程序。

第二条高致病性病原微生物实验室是指三级、四级生物安全实验室；高致病性病原微生物是指卫生部颁布的《人间传染的病原微生物名录》中规定的第一类、第二类病原微生物。

第三条资格审批工作包括申报、评估论证、批准。

第四条实验室资格审批的申请资料，经所在地省级卫生行政部门审查同意后，向卫生部申报。

第二章 申报

第五条申请高致病性病原微生物实验室资格的单位应具备《办法 》第六条规定的条件。

第六条申请高致病性病原微生物实验室资格应按照《办法》第七条的规定，提交完整的资料。

《高致病性病原微生物实验室资格申请表》可从卫生部网站下载（网址：www.moh.gov.cn）。

第七条所有申请资料应一式1份。申请资料应当使用A4规格纸张打印（中文使用宋体小4号字，英文使用12号字）。申报的各项内容应当完整、清楚。申请资料的复印件应当足够清楚并与原件一致。所有申请资料应加盖申请单位公章。

第八条申请单位将符合要求的申请资料报送省级卫生行政部门。省级卫生行政部门对申请资料的合法性、完整性及规范性进行审核。对申请材料不齐全或者不符合规定形式的，应当在5日内出具申请材料补正通知书；对申请材料齐全或者符合规定形式的，应当在15日内提出初审意见，并将初审意见和有关资料报卫生部。

省级卫生行政部门应当直接向卫生部报送资料，不得委托申请单位向卫生部报送资料。

第九条卫生部收到申报资料后，对申报资料进行形式审查。符合要求的，在30日内完成现场评估论证工作。

第三章 现场评估论证

第十条卫生部组织专家组进行现场评估论证，有关要求书面告知申请单位。

第十一条专家组由5～7名相关专业的专家组成，专家由卫生部从病原微生物实验室生物安全专家库中选取。专家组组长由卫生部指定，为现场评估论证工作技术总负责人。

卫生部可指派1～2名管理或专业人员以观察员的身份参加现场评估论证工作。

第十二条现场评估论证时间一般为2～3天。在现场评估论证前，专家组长应当提前制定现场技术考核初步计划。

第十三条卫生部应当在专家组到达评估地前3天将其人员组成情况告知申请单位。申请单位如对专家组成员有异议的，应当在接到通知后24小时内提出需回避的专家名单，并说明理由，由卫生部做出是否回避的决定。

第十四条现场评估程序包括：专家组预备会议、首次会议、资料审查、实验室考察、现场模拟操作考核、理论知识测试、专家组内部会议、末次会议等。

专家组依据计划进行现场评估论证，申请单位应积极配合，并提供相应协助。

第十五条专家组在现场评估论证工作开始前召开全体专家组成员参加的预备会议，会议内容包括：

（一）专家组长重申评估论证工作的公正、客观、保密要求，专家组全体人员签署公正性声明和保密协议；

（二）明确评估范围、内容、依据和要求；

（三）明确评估日程和专家组成员分工，确定现场技术考核计划，准备现场评估和论证所需考核试题等有关资料和表格。

第十六条召开首次会议。参加会议人员包括专家组成员、申请单位负责人及相关人员。会议由专家组组长主持，会议程序及内容如下：

（一）介绍专家组成员和分工；

（二）宣布现场评估论证工作安排、要求和时间表；

（三）明确评估的方法、程序和评定原则；

（四）向申请单位做公正和保密的承诺；

（五）申请单位负责人报告工作情况；

（六）与申请单位确认现场评估所需现场操作和面试考核项目以及被考核人员名单。

第十七条资料审查。专家组审查实验室生物安全手册、程序文件、危害评估报告、标准操作程序、相关记录表格以及《高致病性病原微生物实验室从事实验活动资格现场检查表》涉及的其他资料，并对审查情况进行记录。

第十八条实验室考察。由专家组根据《高致病性病原微生物实验室从事实验活动资格现场检查表》的内容对实验室进行实地考察，并对考察情况进行记录。

第十九条现场模拟操作考核。由专家组从实验室操作人员名单中抽取30%的人员（不少于4人）进行现场操作考核，并对考核情况进行记录。

现场模拟操作考核题目由专家组制订，现场操作应涉及申请范围的主要项目，应当覆盖主要仪器设备、主要人员和主要操作技术。

由每名参试人员抽取1个题目进行现场操作，由2位专家组成员进行评判。评判标准依据实验室的标准操作程序。

第二十条理论知识测试。采取面试形式，全体实验室人员均应参加，参加现场模拟操作考核的人员除外。由2位专家组成员组成考核组，对每名被考核人员进行单独面试并进行评判。

面试考核内容及评判标准依据为《传染病防治法 》、《病原微生物实验室生物安全管理条例 》、《人间传染的高致病性病原微生物实验室和实验活动生物安全审批管理办法》（卫生部令第50号）、《人间传染的病原微生物名录》、《可感染人类的高致病性病原微生物菌（毒）种或样本运输管理规定》（卫生部令第45号）、《实验室生物安全通用要求》（GB 19489-2004）、本实验室生物安全手册、程序文件、标准操作程序（SOP）以及WHO生物安全手册第三版等相关内容。

接受现场操作及面试考核的人员中，未合格者应当重新培训，经考核合格后方能上岗。

第二十一条专家组内部会议。由专家组组长主持，全体专家组成员参加，会议程序及内容：

（一）专家组成员分别报告资料审查、现场模拟操作考核、理论和知识测试、实验室检查等结果，讨论并提出评估论证意见；

（二）编写并通过《高致病性病原微生物实验室从事实验活动资格现场评估论证报告》。

第二十二条末次会议。会议由专家组组长主持，参加人员包括专家组成员、申请单位负责人及相关人员。会议程序及内容：

（一）专家组组长宣读评估论证报告及审查结论；

（二）专家组指出存在的问题，提出整改建议；

（三）专家组与申请单位人员沟通交流意见。

第二十三条专家组长应在现场评估论证结束之日起5日内将评估论证报告、原始记录及有关资料移交卫生部。评估论证报告应当由专家组全体成员签字。

申请单位应按照专家组提出的整改意见，在三个月内完成整改工作，并向卫生部提交整改报告。卫生部在收到整改报告之日起的20日内完成整改复核工作。整改复核工作由原现场评估论证专家组成员完成。

第四章 批准

第二十四条卫生部在收到专家组评估论证报告或者整改复核意见之日起20日内，做出是否批准的决定。对予以批准的，由卫生部颁发《高致病性病原微生物实验室资格证书》。对不予批准的，由卫生部书面通知申请单位，并说明理由。申请单位对卫生部不予批准结论有异议的，可自收到通知书之日起15日内书面向卫生部提出复核申请，逾期不予受理。

未通过资格审批的单位，6个月之后可以重新申请。

第二十五条《高致病性病原微生物实验室资格证书》的有效期为五年。实验室需要继续从事高致病性病原微生物实验活动的，应在有效期满前6个月按照本程序规定，重新申请《高致病性病原微生物实验室资格证书》。

第五章 评估论证工作纪律

第二十六条专家组成员不得与申请单位有利害关系，否则应主动提出回避；卫生行政部门工作人员不得作为专家组成员参加评审。

第二十七条专家组成员要严格按照指定的时间到达和离开现场评估目的地。评审期间，专家组成员不得私下与申请单位联系和接触、传递评审相关信息；专家组各项费用由卫生部负责。专家组在评审地的接待工作由省级卫生行政部门安排，严禁申请单位参加接待工作。专家组不得提出任何与评审工作无关的要求。

第二十八条现场评估过程中，严禁申请单位弄虚作假，严禁通过任何形式对评审施加压力，严禁以各种理由不予配合或拒绝检查。如果出现上述问题，专家组有权终止评审，卫生部也将不予批准。

第六章 附则

第二十九条本程序自颁布之日起施行。

1. 农业农村部办公厅：农业农村部办公厅关于开展2019年全国高级别动物病原微生物实验室生物安全专项检查工作的通知

农办牧〔2019〕38号

为切实做好高级别动物病原微生物实验室生物安全管理,维护养殖业生产安全、动物源性食品安全、公共卫生安全和生态安全,根据《高致病性动物病原微生物实验室生物安全管理审批办法》《兽医实验室生物安全要求通则》等法规规章和我部《2019年畜牧兽医工作要点》,决定组织开展高级别动物病原微生物实验室生物安全监督检查。现将有关事宜通知如下。

一、目标与原则

紧紧围绕“优供给、强安全、保生态”目标,按照“统一标准、属地负责、全面排查、重点管控”的工作原则,落实各级畜牧兽医主管部门的监管责任,进一步强化高级别动物病原微生物实验室设立单位及上级主管部门的日常管理责任,对高级别动物病原微生物实验室特别是农业农村部指定的国家非洲猪瘟相关实验室开展生物安全专项检查,以排查实验室生物安全隐患,进一步提高实验室生物安全管理水平。

二、检查范围

目前已正式运行的高级别动物病原微生物实验室和农业农村部指定的国家非洲猪瘟相关实验室。

三、检查内容

实验室建设和运行情况;实验室生物安全组织机构情况;实验室生物安全管理责任制和有关规章制度落实情况;实验室生物安全防护措施落实情况;实验室从事高致病性动物病原微生物实验活动情况,尤其是开展非洲猪瘟实验活动情况;实验室应急预案制定和实施情况;菌(毒)种和样本保存、销毁情况;实验室工作人员生物安全知识培训情况;实验室记录和档案。

1. 科学技术部：科技部关于聘任高等级病原微生物实验室生物安全审查委员会委员的通知

国科发社〔2012〕603号

为落实《病原微生物实验室生物安全管理条例》（中华人民共和国国务院令第424号），做好我国高等级病原微生物实验室生物安全的相关工作，根据《高等级病原微生物实验室建设审查办法》（科技部第15号令），科技部研究决定，聘任夏咸柱等50名同志为高等级病原微生物实验室生物安全审查委员会委员，聘期为五年（名单见附件）。

请各有关单位积极配合和支持高等级病原微生物实验室生物安全审查委员会委员的工作。

1. 国家卫生健康委员会：国家卫生计生委办公厅关于做好“高致病性病原微生物实验室活动资格审批”取消后的生物安全监管工作的通知

国卫办科教函〔2017〕1069号

依据《国务院关于取消一批行政许可事项的决定》（国发〔2017〕46号）要求，我委决定取消高致病性病原微生物实验室活动资格的行政审批事项。为加强事中事后监管，切实保障与人体健康有关的实验室及其实验活动生物安全，按照《病原微生物实验室生物安全管理条例》（国务院令第424号，以下简称《管理条例》）有关规定，现就有关工作通知如下：

一、切实落实相关监管责任

高级别生物安全实验室的依托单位、上级主管部门和属地卫生计生行政部门，应当依据《管理条例》规定，在各自职责范围内切实履行实验室生物安全管理职责，按照“谁建设谁负责、谁主管谁负责”的原则，全面梳理高级别生物安全实验室运行和管理现状，深入查找存在问题，及时排除安全隐患，研究实施强化监管的措施。

二、进一步严格实验活动审批

依据《国务院关于取消一批行政许可事项的决定》，我委将对《人间传染的高致病性病原微生物实验室和实验活动生物安全审批管理办法》（原卫生部令第50号，以下简称《管理办法》）进行相应修订（修订通知另行印发）。各级卫生计生行政部门应当依据《管理办法 》要求，认真履行“高致病性病原微生物实验活动审批”职责，进一步严格审批标准，优化审批流程，切实发挥审批的监管作用。

三、规范实验室备案等工作

各级卫生计生行政部门应当依据《管理条例》，认真开展生物安全实验室备案工作，完善备案标准，规范备案程序，确保辖区相应实验室备案全覆盖。定期组织备案实验室复核，加强实验室人员培训考核，确保备案工作及时规范。

四、制定实验室生物安全保障工作方案

各省级卫生计生行政部门要按照“统一领导、属地负责，全面监测、及时预警”原则，制定实验室生物安全保障工作方案，明确各方职责，分解管理措施，拟定应急处置预案，建立责任追究制度，纳入重大活动安全保障工作方案中统一部署。

五、强化实验室生物安全日常监督检查

各级卫生计生行政部门应当加强实验室生物安全日常监管，会同卫生计生综合监督部门，研究制订实验室生物安全监督检查工作方案，完善检查内容、强化检查组织、创新检查方式、强化检查结果运用，将实验室生物安全监督检查纳入卫生计生综合执法体系，建立长效机制，列入年度常规工作持续开展。

1. 生态环境部：国家环境保护总局关于病原微生物实验室项目环境影响评价资质有关问题的通知

环办〔2006〕14号

为保证病原微生物实验室项目的环境影响评价质量，更好地防范该类项目可能产生的环境风险，根据环境影响评价分类、分级审批的有关规定和《建设项目环境影响评价资质管理办法》（国家环境保护总局令第26号）的要求，自2006年3月1日起，新建、改建、扩建三级、四级病原微生物实验室（P3、P4实验室）以及生产、进口移动式三级、四级病原微生物实验室项目的环境影响评价，一律编制环境影响报告书并报国家环境保护总局审批。凡接受委托为上述项目进行环境影响评价的机构，应当具备甲级建设项目环境影响评价资质和“化工石化医药”类环境影响报告书评价范围。

1. 农业农村部：农业部办公厅关于做好高致病性病原微生物实验活动资格认定取消后事中事后监管工作的通知

农办医〔2017〕40号

一、加强高致病性动物病原微生物实验活动审批

根据《条例》等法律法规规定，实验室申请开展高致病性动物病原微生物实验活动，应取得国家生物安全实验室认可证书且在有效期内，切实具备相应条件和能力，并经省级以上人民政府兽医主管部门批准；建立完善生物安全管理体系、应急处置预案等制度；实验室及其人员达到操作相应高致病性动物病原微生物的标准和条件，实验活动方案符合生物安全要求。各省级兽医主管部门要严格依据上述规定要求，切实做好高致病性动物病原微生物实验活动审批，要组织开展技术评审，严格实验室标准和条件要求。对新取得国家生物安全实验室认可证书、首次申请从事高致病性动物病原微生物实验活动的，在技术评审时要开展现场评审，进行现场核查和人员考核。实验活动审批应确保实验室已取得国家生物安全实验室认可证书且在有效期内，拟开展的高致病性动物病原微生物实验活动符合国家政策，能保证实验室生物安全。

二、严格高致病性动物病原微生物实验活动承诺和情况报告

实验室申请从事高致病性动物病原微生物实验活动时，应提供《高致病性病原微生物实验活动生物安全承诺书》（见附件），承诺严格按照国家有关法律法规和标准规定要求开展相应高致病性动物病原微生物实验活动。承诺书应由实验室主任、实验室所在单位法人签字并加盖公章，承诺书应长期留存。各实验室在开展高致病性动物病原微生物实验活动期间，应当每季度向原批准部门报告相关情况，具体包括实验活动进展情况、出现的问题及处理情况等，重大事项和突发情况应及时报告。

三、加强高致病性动物病原微生物实验活动全程监督检查

从事高致病性动物病原微生物实验活动的实验室应当建立实验档案，真实、完整地记录实验室使用情况和安全监督情况；建立完善相关制度，强化内部管理；按照有关国家标准、技术规范、操作规程及批复要求开展实验室活动；在实验活动结束后，将分离到的有关高致病性动物病原微生物及时送国家指定的菌（毒）种保藏中心保管或者销毁。县级以上地方人民政府兽医主管部门要依据《条例》规定，按照地方政府统一要求，建立“双随机一公开”制度，加强对辖区内有关实验室的监督检查；组织相关单位加强对国内外科技文献及有关数据库的检索，及时发现疑似未经批准开展高致病性动物病原微生物实验活动的线索，及时调查处理；加大对投诉举报的处理力度，严肃查处违法从事高致病性动物病原微生物实验活动的行为。

四、加强相关科研成果发表的生物安全审查

各省级兽医主管部门要根据《科技部、教育部、农业部 卫生部、中国科学院、中国科协关于加强我国病毒研究成果发表管理的通知》（国科发社〔2012〕921号），加强对所属研究机构、出版机构有关高致病性病原微生物研究成果发表的管理，以防范生物安全风险，确保公共安全和社会稳定。

1. 农业农村部：农业部关于进一步规范高致病性动物病原微生物实验活动审批工作的通知

农医发〔2008〕27号

为进一步规范高致病性动物病原微生物实验活动审批行为，加强动物病原微生物实验室生物安全管理，现就有关事项通知如下。

一、严格掌握高致病动物病原微生物实验活动审批条件

高致病性动物病原微生物实验活动，事关重大动物疫病防控，事关实验室工作人员及广大人民群众身体健康和生命安全。省级以上兽医主管部门要高度重视高致病性动物病原微生物实验活动管理，认真贯彻实施《病原微生物实验室生物安全管理条例

》，按照《高致病性动物病原微生物实验室生物安全管理审批办法

》规定的条件，严格高致病性动物病原微生物实验活动审批。

（一）高致病性动物病原微生物实验活动所需实验室生物安全级别。按照《病原微生物实验室生物安全管理条例

》和《高致病性动物病原微生物实验室生物安全管理审批办法

》规定，一级、二级实验室不得从事高致病性动物病原微生物实验活动；三级、四级实验室需要从事某种高致病性动物病原微生物或者疑似高致病性动物病原微生物实验活动的，应当经农业部或者省、自治区、直辖市人民政府兽医主管部门批准。经省级以上兽医主管部门批准的高致病性动物病原微生物实验活动，必须按照《动物病原微生物实验活动生物安全要求细则》（附后）的要求，在相应生物安全级别的实验室内开展有关实验活动。

（二）高致病性动物病原微生物实验活动审批条件。三级、四级实验室从事高致病性动物病原微生物或者疑似高致病性动物病原微生物实验活动的，应当具备下列条件：一是必须取得农业部颁发的《高致病性动物病原微生物实验室资格证书》，并在有效期内；二是实验活动仅限于与动物病原微生物菌（毒）种或者样本有关的研究、检测、诊断和菌（毒）种保藏等；三是科研项目立项前必须经农业部批准。

二、严格规范高致病性动物病原微生物实验活动审批程序

省级以上兽医主管部门应当按照《高致病性动物病原微生物实验室生物安全管理审批办法

》和农业部第898号公告规定的审批主体、审批程序，做好高致病性动物病原微生物实验活动审批工作。

（一）审批主体。从事下列高致病性动物病原微生物实验活动的，应当报农业部审批：一是猪水泡病病毒、非洲猪瘟病毒、非洲马瘟病毒、牛海绵状脑病病原和痒病病原等我国尚未发现的动物病原微生物；二是牛瘟病毒、牛传染性胸膜肺炎丝状支原体等我国已经宣布消灭的动物病原微生物；三是高致病性禽流感病毒、口蹄疫病毒、小反刍兽疫病毒等烈性动物传染病病毒。从事其他高致病性动物病原微生物实验活动的，由省、自治区、直辖市人民政府兽医主管部门审批。

（二）审批程序。实验室申请从事高致病性动物病原微生物实验活动的，应当向所在地省、自治区、直辖市人民政府兽医主管部门提出申请，并提交下列材料：一是高致病性动物病原微生物实验活动申请表一式两份；二是高致病性动物病原微生物实验室资格证书复印件；三是从事与高致病性动物病原微生物有关的科研项目，还应当提供科研项目立项证明材料。省级以上兽医主管部门按照职责分工，应当在收到申请材料之日起15日内做出是否审批的决定。

三、切实加强高致病性动物病原微生物实验活动监督管理

高致病性动物病原微生物实验活动管理是实验室生物安全监管的重点内容。各级兽医主管部门一定要认真贯彻实施《病原微生物实验室生物安全管理条例

》的各项规定，采取切实有效措施，对高致病性动物病原微生物实验活动实行全程监管，确保实验室生物安全，确保实验室工作人员和广大人民群众身体健康。

（一）严肃查处违法从事实验活动的行为。各级兽医主管部门要严格执行高致病性动物病原微生物实验活动事前审批制度。对未经批准从事高致病性动物病原微生物实验活动的，要依法严肃查处，三年内不再批准该实验室从事任何高致病性动物病原微生物实验活动。

（二）加强实验活动监督检查。各级兽医主管部门要定期组织实验活动监督检查。重点检查实验室是否按照有关国家标准、技术规范和操作规程从事实验活动，及时纠正违规操作行为。要督促实验室加强内部管理，制定并落实安全管理、安全防护、感染控制和生物安全事故应急预案等规章制度。

（三）严格执行实验活动报告制度。经批准的实验活动，实验室应当每半年将实验活动情况报原批准机关。实验活动结束后，应当及时将实验结果以及工作总结报原批准机关。未及时报告的，兽医主管部门要责令改正，并给予警告处罚。

1. 农业农村部：农业农村部办公厅关于做好高等级动物生物安全实验室资源共享工作的指导意见

农办牧〔2022〕17号

高等级动物生物安全实验室是做好动物疫病防控工作的重要技术支撑平台，具有较强公益属性。实验室资源共享是推动动物疫病防控技术和产品研究、促进科技成果转化的重要途径。按照《中华人民共和国生物安全法》《病原微生物实验室生物安全管理条例》《动物病原微生物菌（毒）种保藏管理办法》等有关规定，现就做好高等级动物生物安全实验室资源共享工作提出如下意见。

一、总体要求

（一）指导思想

以习近平新时代中国特色社会主义思想为指导，全面贯彻党的十九大和十九届历次全会精神，认真落实习近平总书记关于加强我国生物安全建设的重要指示精神，贯彻落实生物安全法，统筹发展和安全，破除利益藩篱，创新机制、深化协作、推动共享，加快推进生物科技创新和产业化应用，形成统筹协调、竞争有序、共享共赢的科研发展新格局。

（二）主要原则

依法依规。严格按照《中华人民共和国生物安全法》《病原微生物实验室生物安全管理条例》《动物病原微生物菌（毒）种保藏管理办法》相关要求开展实验室资源共享。

合作共赢。牢牢守住安全底线，探索建立“开放、流动、联合、竞争”机制，发掘平台条件，促进资源互补，释放服务潜能，激发创新活力。

二、主要内容

一是共享实验室设施设备。适应我国重大动物疫病防控新形势，促进兽医科技发展，推动生物制品研发，各实验室要科学合理制定实验计划，在确保完成主责主业的基础上，创造条件为有试验需求的单位或企业开展大动物免疫效力评价等开放实验设施设备，为动物防疫科技创新提供保障和支撑。

二是提供流行株菌（毒）种。动物病原微生物流行株菌（毒）种是国家重要战略资源。被指定为菌（毒）种保藏机构的高等级动物生物安全实验室，必须依法依规履行“向合法从事动物病原微生物实验活动的实验室或兽用生物制品企业提供菌（毒）种或样本”的法定职责，满足国家动物疫病防控、科研和疫苗产品检验等需要。

三是强化专业人员及技术支持。高等级动物生物安全实验室设立单位要在特定高致病性动物病原微生物研究和应用方面充分发挥技术平台和专业人才队伍优势，为缺乏高等级动物病原微生物实验活动操作技术及专业人员的单位或企业施以援手，提供人员支持、技术指导，共享管理经验和实验技术，为建设中的高等级动物生物安全实验室提供技术咨询，培养生物安全和动物疫病防控复合型人才。

四是共同组织项目申报。各实验室要以推动国家生物安全能力建设和保障畜牧业发展为导向，立足人畜共患病和重大动物疫病防控需求，充分发挥高等级动物生物安全实验室设施平台和技术人才优势，积极与其他主体合作申报项目，解决行业发展瓶颈问题，推动形成联合协作、联合攻关的共同发展局面。

三、保障措施

一是加强组织领导。各高等级动物生物安全实验室设立单位要切实提高政治责任感，深刻理解实验室资源的公益属性，充分认识实验室资源开放共享的重要性及紧迫性，成立实验室资源共享工作领导小组，明确专人专职负责。

二是建立共享利用机制。各高等级动物生物安全实验室设立单位和菌（毒）种保藏机构，要以构建“一盘棋”的工作格局为目标，在确保实验室生物安全的前提下，按照法律法规相关要求，建立实验资源共享利用机制，制定实验室资源开放共享方案，并于6月30日前报我部畜牧兽医局备案。

1. 国务院应对新型冠状病毒肺炎疫情联防联控机制综合组：关于印发区域新型冠状病毒核酸检测组织实施指南（第三版）的通知

联防联控机制综发〔2022〕28号

七、规范人员等相关管理

（一）规范人员及实验室生物安全管理。各地应按照《中华人民共和国生物安全法》《病原微生物实验室生物安全管理条例》《新型冠状病毒实验室生物安全指南（第二版）》等规定，按要求进行实验室备案，加强实验室生物安全管理，防范生物安全事件发生。加强医务人员及其他非医务人员的感染控制培训、区域核酸检测信息系统应用培训。采样人员、实验室检测人员防护要求按照《医疗机构新型冠状病毒核酸检测工作手册（试行第二版）》执行。当地要做好统筹协调，加强对参与核酸采样、检测人员的关心关爱，做好后勤保障工作。

（二）加强医疗废物处理。各地应规范医疗废物管理，做好医疗废物收集、包装、无害化处理、暂存、交接和转运等工作，使用双层包装袋盛装医疗废物，有效封口，确保封口严密，确保医疗废物包装无破损、无渗漏。医废处置专班要及时协调具有相应资质的医疗废物处置单位，处置转运采样点和核酸检测机构的医疗废物。核酸采样点产生的医疗废物要当日清运；核酸检测机构产生的医疗废物在暂存条件允许时，暂存时间不得超过2天，医疗废物产生量激增无法安全暂存时，要当日清运。核酸检测机构应根据暂存场所、医疗废物贮存情况，与收运单位预约清运时间，清运应避免大风、雷雨天气。收运单位要优化运输车辆调度，合理安排收运路线，做好医疗废物清运保障。

1. 国家卫生健康委员会：国家卫生健康委关于印发《“十四五”卫生健康标准化工作规划》的通知

国卫法规发〔2022〕2号

制定实验室生物安全标准,加强对病原微生物实验室生物安全的管理。结合近年传染病的防控形势和病原微生物实验室的建设与发展，针对细菌、病毒、真菌、寄生虫等病原微生物实验室的风险评估、生物安全与安保、实验活动、设施设备等，建立病原微生物实验室生物安全标准体系，保障实验室生物安全，为传染病防控提供技术支持与保障。做好医疗卫生机构消防、安检、放射卫生防护等重大安全相关标准的制定和实施。加强卫生健康网络安全标准建设。

1. 国家发展和改革委员会：国家发展改革委关于印发《“十四五”生物经济发展规划》的通知

发改高技〔2021〕1850号

集约化建设生物安全基础设施。加快建设生物信息、人类遗传资源保藏、菌（毒）种保藏、动植物遗传资源保藏等国家战略资源平台。围绕人口健康、检验检疫、国防安全等重点领域，坚持总量调控、因需布局、动态调整，统筹布局建设高级别生物安全实验室。加强对国内病原微生物实验室生物安全的管理，严格执行有关标准规范，严格管理实验样本、实验动物、实验活动废弃物。

1. 农业农村部：农业农村部办公厅关于成立全国动物病原微生物菌（毒）种保藏管理专家委员会的通知

农办牧〔2021〕26号

根据《中华人民共和国生物安全法》《病原微生物实验室生物安全管理条例》《动物病原微生物菌（毒）种保藏管理办法 》等规定，为加强动物病原微生物菌（毒）种保藏管理，充分发挥相关领域专家的技术支撑作用，我部决定成立全国动物病原微生物菌（毒）种保藏管理专家委员会，归口我部畜牧兽医局管理，并制定了全国动物病原微生物菌（毒）种保藏管理专家委员会章程，遴选确定了第一届委员会委员名单，现予以公布。请各有关单位积极支持专家委员会工作。

1. 国家卫生健康委员会、中央军委后勤保障部：关于印发《血站新冠肺炎疫情常态化防控工作指引》的通知

国卫办医函〔2021〕155号

四、实验室检测相关要求

（一）加强实验室规范化管理。实验室应当严格遵从《病原微生物实验室生物安全管理条例》《实验室生物安全通用要求》（GB19489-2008）和《病原微生物实验室生物安全通用准则》（WS233-2017）的规定，建立并严格遵守生物安全管理制度与安全操作规程。加强血液检测人员基础知识、基本理论、检测技术和生物安全的培训，正确认识实验室范围内可能存在的风险。

1.实验室工作人员防护。参照血液检测实验室人员防护要求。

2.样本接收与处理。实验室工作人员在收送标本过程中，应当对转运箱和转运标本架进行消毒，建议采用75%乙醇或其他有效的消毒剂擦拭消毒。

标本在运输、接收、离心或检测过程中出现标本管破损或渗漏时应当及时处理，建议使用含有效氯5500mg/L的消毒剂或其他有效的消毒剂喷洒，覆盖足够大的范围，包括喷溅的最远处。作用30分钟后再将标本管及吸水纸等放入医疗废物袋中，注意不要污染地面台面。

检测后样本宜加盖保存，阳性或疑似样本加化学消毒剂或其他有效的消毒剂消毒，放置在双层医疗废物袋内密封，用75%乙醇或其他有效的消毒剂擦拭消毒医疗废物袋表面后按医疗废物处理。应当制定样本储存冰箱清洁消毒操作程序，并有记录。

3.环境清洁消毒。一般情况下使用含有效氯500mg/L的消毒剂，严重污染时（样本有渗漏、溅出时）使用含有效氯5500mg/L的消毒剂。实验室在工作状态时可采用动态空气消毒（如有），非工作状态时开启紫外照射（应当验证紫外照度）。必要时,采用过氧化氢消毒设备开展终末消毒。

（二）加强实验室能力建设。实验室的设备配备应当满足各地检测策略、检测方法、检测样本量的要求，做好设备的维护、校准和性能监测等工作，特别是应当做好备用设备与常规使用设备的性能比对，确保备用设备随时可以启用。有条件的地区可配备常规血液核酸筛查和新冠病毒血液核酸检测通用的设备作为技术储备，在出现新冠肺炎病例社区传播的流行地区，可对献血者进行新冠病毒核酸和/或IgM抗体检测。

1. 科学技术部：科学技术部关于《生物安全三级实验室建设与管理有关事项》的通知

国科发农社字〔2003〕212号

根据当前我国非典型肺炎防治科技攻关的急需和构建我国应对突发性传染病的科技体系的要求，在全国防治非典型肺炎指挥部科技攻关组的领导下，科技部会同有关部门紧急部署了非典型肺炎P3实验室的有关建设管理工作。

P3实验室建设不但投资大，运转和维持费用高，而且对实验人员的条件及管理要求非常严格，盲目建设和一哄而上将造成国家资源极大的浪费，管理不当，还会带来严重安全隐患。国务院领导对此给予了高度重视，曾多次批示生物安全实验室建设要统筹规划，加强管理和监控。根据国务院领导的指示精神，现将有关P3实验室建设和管理事宜通知如下：

1．统一规划管理。科技部会同卫生部、农业部、国家食品药品监管局和国家环保总局等部门，负责P3实验室建设的统一规划、立项审批、考核认证和监督管理等工作。制订全国P3实验室总体规划、管理办法及实施细则，确保P3实验室合理布局，安全运行，并实现资源共享。

2．严格立项审批。各省市、各部门的P3实验室改扩建和新建工作必须在国家的统一规划和布局下，合理安排。除国家已安排的P3实验室改扩建和新建项目外，其他改扩建和新建项目必须报送国家科技部批准后，方可开展相关工作。未经批准，不得擅自开展改扩建和新建工作。

3．强化责任意识。各有关单位、各P3实验室要严格按照四部局联合发布的《传染性非典型肺炎病毒研究实验室暂行管理办法》执行。各省、部级科技行政主管部门要切实加强对本地区、本部门P3实验室的监督、检查和管理，明确专人负责，建立责任追究制。对不按规定执行的单位和个人要严肃处理。

4．加强技术指导。科技部会同卫生部、农业部、国家食品药品监管局和国家环保总局等部门，成立国家生物安全实验室专家委员会，加强对生物安全实验室技术人员的技术指导和培训以及对生物安全实验室安全检查、质量评估等工作，确保生物安全实验工作得到有效的监督。

1. 国务院应对新型冠状病毒肺炎疫情联防联控机制综合组：关于印发《大规模新冠病毒核酸检测实验室管理办法（试行）》的通知

联防联控机制综发〔2021〕33号

第一条 为规范大规模新冠病毒核酸检测工作，保障检测效率和质量，有效控制疫情，根据《传染病防治法》《突发公共卫生事件应急条例》《医疗机构管理条例》《病原微生物实验室生物安全管理条例》《医疗机构临床实验室管理办法》等法律法规规定，制定本办法。

第二条 本办法所称大规模新冠病毒核酸检测，是指辖区内局部或全部人群需开展新冠病毒核酸检测，检测量超过辖区内单体医疗卫生机构日常最大检测能力，需调动辖区内更多或辖区外检测力量，共同完成的核酸检测工作。

第三条 开展大规模新冠病毒核酸检测的实验室（以下简称大规模检测实验室），包括具备新冠病毒核酸检测资质的医疗机构实验室（含医学检验实验室，下同）和疾控机构实验室。

第四条 根据交通条件、人口及医疗卫生机构资源分布等因素，对设区的地市级以上城市及县域内开展大规模检测的实验室进行分类管理。

第五条 大规模检测实验室应当同时符合以下条件：

（一）取得《医疗机构执业许可证》的医疗机构，或取得《事业单位法人证书》的疾控机构；

（二）医疗机构实验室应当符合《医疗机构临床基因扩增检验实验室管理办法》的要求；

（三）按照规定规范开展室内质控，并参加省级及以上卫生健康行政部门委托临床检验中心或其他机构组织的实验室室间质评，且最近两次质评结果合格；

（四）具备经过卫生健康行政部门审核备案的生物安全二级或以上实验室条件；

（五）近两年内未受行政处罚，信誉良好；

（六）省级卫生健康行政部门根据检测时效要求规定的其他条件。

第六条 承担设区的地市级以上城市大规模新冠病毒核酸检测的实验室，除具备第五条规定的条件外，原则上还应当具备每天检测至少5000管的能力。

承担县域大规模新冠病毒核酸检测的实验室，除具备第五条规定的条件外，原则上还应当具备每天检测至少1000管的能力。县域内的最大检测能力不足时，可委托其他大规模检测实验室开展检测。

地方卫生健康行政部门可根据检测工作需要，对不具备大规模核酸检测能力的实验室统筹分派检测任务。

第七条 按照《进一步推进新冠病毒核酸检测能力建设工作方案》建设的公共检测实验室和城市检测基地，应当承担大规模新冠病毒核酸检测任务。

第八条 接受卫生健康行政部门调度前往支援的大规模检测实验室应当符合本办法第五条的规定。受援地应当为实验室开展工作提供必要的交通、食宿、场地、医疗废物收集和处置等条件，保障检测及时顺利安全开展。

第九条 拟承担大规模新冠病毒核酸检测任务的实验室应当向省级卫生健康行政部门提出申请，按照规定的条件提供书面材料，并保证材料真实、准确、可靠。主要包括实验室场地、人员、仪器设备、检测能力、室内质控记录、室间质评结果等。

第十条 省级卫生健康行政部门收到材料后，应当组织相关专业技术人员对材料进行审核，必要时进行现场查验。对符合条件的，应当在临床基因扩增检验实验室技术审核合格的证明文件中，加注“大规模新冠病毒核酸检测”；或通过证明文件、文书等方式标明该实验室具备大规模新冠病毒核酸检测能力。同时，在“全国新冠病毒核酸检测信息平台”中予以标识，供辖区内开展大规模新冠病毒核酸检测时参考使用。

第十一条 开展大规模新冠病毒核酸检测时，可通过临时增加人员、设备等快速提高检测能力。拟承担检测任务的实验室，应当按照前款规定向省级卫生健康行政部门提出审核申请。审核通过的，在临床基因扩增检验实验室技术审核合格的证明文件中，加注“大规模新冠病毒核酸检测”；或通过证明文件、文书等方式标明该实验室具备大规模新冠病毒核酸检测能力，并标明有效期。

第十二条 大规模检测实验室应当建立实验室质量管理体系和应急管理体系，制订工作预案，加强物资储备，强化人员技术培训，提高应急反应能力，确保随时开展工作。

第十三条 大规模检测实验室应当根据自身检测能力接收新冠病毒核酸样本，避免样本数量明显超出检验能力导致的样本积压、样本失效、检测结果反馈迟缓等问题。

第十四条 大规模检测实验室从事检验工作的人员应当是按照规定接受技术培训并考核合格，持有《临床基因扩增检验技术人员上岗证》的卫生技术人员。签发核酸检测报告的人员还应当同时具备相应资质。

第十五条 大规模检测实验室应当在规定的时间内出具核酸检测结果，出具的核酸检测结果应当真实、准确、客观、公正。

第十六条 各地决定开展大规模新冠病毒核酸检测后，应当根据交通条件、检测能力等，从通过审核的大规模检测实验室中遴选相关机构，签订委托协议，明确检测量、完成时限、检测费用等，并约定其他有关事项。

第十七条 接到检测任务后，大规模检测实验室应当立即激活应急管理体系，确保在短时间内人员到位、设备到位、物资到位，样本送达后即刻开展检测。

第十八条 大规模检测实验室应当按照有关规定，每批次检测时，随机进行弱阳性和阴性室内质控，并定期参加国家或省级组织的室间质评。

第十九条 鼓励大规模检测实验室在每个分区的核心工作区域安装摄像设备，安装位置能清楚记录检测的关键流程、关键部位和操作，实时录制加样、抽取、扩增和报告等关键环节全过程。影像资料至少保存一个月。

第二十条 在开展大规模新冠病毒核酸检测期间，卫生健康行政部门应当向大规模检测实验室派驻质量监督员，对实验室室内质控等工作进行监督，并做好记录。出现问题时，派驻的质量监督员应当及时向卫生健康行政部门报告。

第二十一条 大规模检测实验室出现以下情形之一的，卫生健康行政部门不再允许其承担检测任务，废止加注“大规模新冠病毒核酸检测”临床基因扩增检验实验室技术审核合格证明文件：

（一）实验室条件发生明显变化，不符合大规模检测实验室规定条件时；

（二）在质量监督过程中发现检测不规范，可能影响检测质量的；

（三）擅自将样本转包给其他实验室的；

（四）样本积压超过样本保存有效期的；

（五）在检测过程中未开展室内质控，未参加室间质评的；

（六）未经卫生健康行政部门同意，擅自进行混采、混检的；

（七）未在规定时间内出具核酸检测结果的；

（八）未在约定时限内完成核酸检测任务的。

第二十二条 室间质量评价连续两次以上不合格，经整改后仍不合格的，由卫生健康行政部门暂停其开展新冠病毒核酸检测业务。

第二十三条 使用未经专业培训、无核酸检测培训证明的人员从事核酸检验工作的，按照《医疗机构管理条例》第四十八条处罚。

第二十四条 出具虚假检验报告的大规模检测实验室，按照《医疗机构管理条例》第四十九条处罚。对出具虚假检验报告的医师，按照《执业医师法》第三十七条处罚。

第二十五条 出现其他违反《医疗机构管理条例》《病原微生物实验室生物安全管理条例》及《医疗机构管理条例实施细则》的，由卫生健康行政部门依法依规从严从重处理。

第二十六条 卫生健康行政部门未按照规定从具备条件的大规模检测实验室中确定检测单位的，各地应当依法依规对相关责任人予以处分；造成严重后果的，依法依规追究相关责任。

第二十七条 本办法由国家卫生健康委负责解释。

第二十八条 本办法自发布之日起施行。

1. 国务院应对新型冠状病毒肺炎疫情联防联控机制医疗救治组：关于印发《医疗机构新型冠状病毒核酸检测工作手册（试行 第二版）》的通知

联防联控机制医疗发〔2020〕313号

（一）标本安全管理。标本转运箱封闭前，须使用75%酒精或0.2%含氯消毒剂喷洒消毒。标本包装应符合国际民航组织文件Doc9284《危险品航空安全运输技术细则》的PI602分类包装要求。根据当前版本的国际航空运输协会（IATA）《危险品规则》，SARS-CoV-2感染疑似和确诊患者标本属于UN3373B类生物物质，涉及外部标本运输的，应按照B类感染性物质进行三层包装。疑似或确诊患者标本应标示有特殊标识，并进行单独转运。检测完成后的剩余标本，可在结果报告发出到达其保存时限要求后，如为检测前非灭活标本，则装入专用密封废物转运袋中进行压力蒸汽灭菌处理，随后随其他医疗废物一起转运出实验室进行销毁处理；如为检测前已灭活标本，则无需高压灭活，直接按医疗废物一起转运出实验室进行销毁处理。

（二）实验室检测安全管理。

1.基本要求。核酸检测应当在生物安全二级实验室进行，并应在生物安全风险评估的基础上，采取适当的个体防护措施，包括手套、口罩和隔离衣等。开展新冠病毒核酸检测的实验室应当制定实验室生物安全相关程序文件及实验室生物安全操作失误或意外的处理操作程序，并有记录。

2.实验前安全要求。应使用0.2%含氯消毒剂或75%酒精进行桌面、台面及地面消毒。消毒液需每天新鲜配制，不超过24小时。转运至实验室的标本转运桶应在生物安全柜内开启。转运桶开启后，使用0.2%含氯消毒剂或75%酒精对转运桶内壁和标本采集密封袋进行喷洒消毒。取出标本采集管后，应首先检查标本管外壁是否有破损、管口是否泄露或是否有管壁残留物。确认无渗漏后，推荐用0.2%含氯消毒剂喷洒、擦拭消毒样品管外表面（此处不建议使用75%酒精，以免破坏标本标识）。如发现渗漏应立即用吸水纸覆盖，并喷洒有效氯含量为0.55%的含氯消毒剂进行消毒处理，不得对标本继续检测操作，做好标本不合格记录后需立即进行密封打包，压力蒸汽灭菌处理后销毁。

如为采样管为非灭活管，实验室操作人员在进行标本热灭活时，温浴前需旋紧标本采集管管盖，必要时可用封口膜密闭管盖；温浴过程中可每隔10分钟将标本轻柔摇匀1次，以保证标本均匀灭活；温浴后标本需静置至室温至少10分钟使气溶胶沉降，随后再开盖进行后续核酸提取。

3.核酸提取和检测安全要求。标本进行核酸提取和检测时应尽可能在生物安全柜内进行操作。如为打开标本管盖或其他有可能产生气溶胶的操作，则必须在生物安全柜内进行。

4.实验结束后清洁要求。需对实验室环境进行清洁，消除可能的核酸污染。

（1）实验室空气清洁。实验室每次检测完毕后，可采用房间固定和/或可移动紫外灯进行紫外照射2小时以上。必要时可采用核酸清除剂等试剂清除实验室残留核酸。

（2）工作台面清洁。每天实验后，使用0.2%含氯消毒剂或75%酒精进行台面、地面清洁。

（3）生物安全柜消毒。实验使用后的耗材废弃物放入医疗废物垃圾袋中，包扎后使用0.2%含有效氯消毒液或75%酒精喷洒消毒其外表面。手消毒后将垃圾袋带出生物安全柜放入实验室废弃物转运袋中。试管架、实验台面、移液器等使用75%酒精进行擦拭。随后关闭生物安全柜，紫外灯照射30分钟。

（4）转运容器消毒。转运及存放标本的容器使用前后需使用0.2%含氯消毒剂或75%酒精进行擦拭或喷洒消毒。

（5）塑料或有机玻璃材质物品清洁：使用0.2%含氯消毒剂或过氧乙酸或过氧化氢擦拭或喷洒。

（三）实验室医疗废物管理。

1.基本要求。开展新冠病毒核酸检测的实验室应当制定医疗废物处置程序及污物、污水处理操作程序。所有的危险性医疗废物必须按照统一规格化的容器和标示方式，完整且合规地标示废物内容。应当由经过适当培训的人员使用适当的个人防护装备和设备处理危险性医疗废物。实验室应建立医疗废物处理记录，定期对实验室排风HEPA过滤器进行更换，定期对处理后的污水进行监测，采用生物指示剂监测压力灭菌效果。

2.医疗废物的处理措施。医疗废物的处理是控制实验室安全的关键环节，必须充分掌握生物安全废弃物的分类，并严格执行相应的处理程序。

（1）废液的处理。实验室产生的废液可分为普通污水和感染性废液。普通污水产生于洗手池等设备，对此类污水应当排入实验室水处理系统，经统一处理达标后进行排放。感染性废液即在实验操作过程中产生的废液，需采用化学消毒（0.55%含氯消毒剂处理）或物理消毒（紫外照射30分钟以上）方式处理，确认彻底消毒灭活后方可排入实验室水处理系统，经统一处理达标后进行排放。污水消毒处理效果按GB18466《医疗机构水污染物排放标准》相关规定进行评价。

（2）固体废物的处理。实验室固体废物应当分类收集。固体废物的收集容器应当具有不易破裂、防渗漏、耐湿耐热、可密封等特性。实验室内的潜在感染性废物不允许堆积存放，应当及时进行压力蒸汽灭菌处理。废物处置之前，应当存放在实验室内指定的安全位置。小型固体废物如检测耗材、个人防护装备等均需使用双层防渗漏专用包装袋打包密封后经过压力蒸汽灭菌处理，再转运出实验室。

体积较大的固体废物如HEPA过滤器，应当由专业人士进行原位消毒后，装入安全容器内进行消毒灭菌。不能进行压力蒸汽灭菌的物品如电子设备可采用环氧乙烷熏蒸消毒处理。经消毒灭菌处理后移出实验室的固体废物需集中交由医疗废物处理单位进行处置。

（四）实验室污染的处理。

1.标本污染生物安全柜的操作台造成局限污染时：立即用吸水纸覆盖，并使用0.55%含氯消毒剂进行喷洒消毒。消毒液需要现用现配，24小时内使用。

2.标本倾覆造成实验室污染时：保持实验室空间密闭，避免污染物扩散。立即使用润湿有0.55%含氯消毒剂的毛巾覆盖污染区。必要时（如大量溢撒时）可用过氧乙酸加热熏蒸实验室，剂量为2g/m3，熏蒸过夜；或20g/L过氧乙酸消毒液用气溶胶喷雾器喷雾，用量8ml/m3，作用1-2小时；必要时或用高锰酸钾-甲醛熏蒸：高锰酸钾8g/m3，放入耐热耐腐蚀容器（陶罐或玻璃容器），后加入甲醛（40%）10ml/m3，熏蒸4小时以上。熏蒸时室内湿度60%-80%。

3.清理污染物时严格遵循活病毒生物安全操作要求，采用压力蒸汽灭菌处理，并进行实验室换气等，防止次生危害。

1. 国家卫生健康委员会、中央军委后勤保障部：关于印发《血站秋冬季新冠肺炎疫情防控工作指引》的通知

国卫办医函〔2020〕930号

四、实验室检测相关要求

（一）加强实验室规范化管理。实验室应当严格遵从《病原微生物实验室生物安全管理条例》、《实验室生物安全通用要求》（GB19489-2008）和《病原微生物实验室生物安全通用准则》（WS233-2017）的规定，建立并严格遵守生物安全管理制度与安全操作规程。加强血液检测人员基础知识、基本理论、检测技术和生物安全的培训，正确认识实验室范围内可能存在的风险。

1.实验室工作人员防护。参照血液检测实验室人员防护要求。

2.样本接收与处理。实验室工作人员在收送标本过程中，应当对转运箱、转运标本架及标本进行消毒，建议采用75%乙醇或其他有效的消毒剂擦拭消毒。

标本在运输、接收、离心或检测过程中出现标本管破损或渗漏时应当及时处理，建议采用有效氯浓度为5500mg/L的消毒液或其他有效的消毒剂喷洒，覆盖足够大的范围，包括喷溅的最远处。作用30分钟后再将标本管及吸水纸等放入医疗废物袋中，注意不要污染地面台面。

检测后样本宜加盖保存，阳性或疑似样本加化学消毒剂或其他有效的消毒剂消毒，放置在双层医疗废物袋内密封，用75%乙醇或其他有效的消毒剂擦拭消毒医疗废物袋表面后按医疗废物处理。注意样本储存冰箱应当有消毒措施。

3.环境清洁消毒。一般情况下使用有效氯浓度为500mg/L，严重污染时（样本有渗漏、溅出时）使用有效氯浓度为5500mg/L。实验室在工作状态时可采用动态空气消毒（如有），非工作状态时开启紫外照射（应当验证紫外照度）。必要时,采用过氧化氢消毒设备开展终末消毒。

（二）加强实验室能力建设。实验室的设备配备应当满足各地检测策略、检测方法、检测样本量的要求，做好设备的维护、校准和性能监测等工作，特别是应当做好备用设备与常规使用设备的性能比对，确保备用设备随时可以启用。有条件的地区可配备常规血液核酸筛查和新冠病毒血液核酸检测通用的设备作为技术储备，在出现新冠肺炎病例社区传播的流行地区，可对献血者进行新冠病毒核酸和/或IgM抗体检测。

1. 国家卫生健康委员会：卫生部关于切实加强传染性非典型肺炎病毒毒株、人体标本集中管理、确保病毒实验室及保管单位生物安全的紧急通知

一、各地要严格执行《卫生部办公厅关于落实传染性非典型肺炎相关人体样品和病毒毒株集中管理工作的通知》（卫办科教发[2003]141号）精神，切实加强非典病毒毒株、人体标本和实验室生物安全的管理工作。各地、各单位必须按照有关规定将非典病毒毒株和人体标本在规定时间内送交指定单位统一保管，并确保无一遗漏，对拒不执行者予以通告，对出现严重后果的将依法严肃追究其责任。

二、凡从事非典研究的单位必须按程序申报，并经有关部门考察批准后方可进行有关非典的研究。未经批准、不具备研究条件的单位，严禁擅自开展非典研究工作。经批准的研究单位要严格按照《卫生部关于进一步加强从事传染性非典型肺炎病毒研究实验室生物安全的紧急通知》等有关文件要求开展工作。

三、对非典病毒毒株、人体标本的定点保管单位和具备资质的实验室要切实加强生物安全防护的管理，完善生物安全防护的标准和规范，落实生物安全防护的科学措施，确保保管单位和实验室生物安全工作万无一失。

四、非典病毒毒株、人体标本的定点保管单位和具备资质的实验室要立即按本通知精神和有关规定开展自查自纠工作，排除生物安全工作中存在的任何隐患，确保研究及保管人员的安全，确保病毒毒株和样品无外泄，确保人民群众的身体健康和生命安全。

五、要进一步加强对从事非典研究、毒株和样品保管人员的系统规范培训，提高安全防护意识和生物安全操作技能，未接受培训者不得接触毒株和样本。

我部将组织专家进行专项督查。各地、各单位在执行过程中如发现问题，要及时报告上级单位，并尽快反馈我部。有关部门和单位、有关领导要本着对人民身体健康和生命安全高度负责的态度，切实加强非典毒株、样品的保管和实验室安全防护管理工作。

1. [国家卫生健康委员会](https://www.pkulaw.com/law/chl?Aggs.IssueDepartment=60321&way=textBasic) [国家发展和改革委员会](https://www.pkulaw.com/law/chl?Aggs.IssueDepartment=60301&way=textBasic)：关于印发《医疗卫生机构检验实验室建筑技术导则（试行）》的通知

国卫办规划函〔2020〕751号

一、总则

第一条为指导各地医疗卫生机构检验实验室建设，根据《综合医院建筑设计规范》GB51039、《传染病医院建筑设计规范》GB50849、《传染病医院建筑施工及验收规范》GB50686、《疾病预防控制中心建筑技术规范》GB50881等相关规范、标准的要求，制定本技术导则。

第二条本导则适用于医院检验科实验室的新建、改建、扩建工程项目，疾控中心、急救站等其他医疗卫生机构检验实验室项目可参照执行。

第三条医疗卫生机构检验实验室的建设，必须坚持科学、合理、实用、安全、环保等原则，应正确处理现状与发展、需求与可行性的关系。

第四条有生物安全要求的检验实验室，应符合现行《生物安全实验室建筑技术规范》GB50346、《实验室生物安全通用要求》GB19489、《移动式实验室生物安全要求》GB27421、《实验室设备生物安全性能评价技术规范》RB/T199、《病原微生物实验室生物安全通用准则》WS233的有关规定。

二、选址和建筑设计

第五条检验实验室应根据工作属性、内容、服务对象等，结合工作流程、人物流线、洁污流线、空间要求、物理条件等做好选址和布局。

第六条检验实验室内部空间布局应满足日常业务操作，兼顾大型设备的搬运、安装和检修等空间要求，并适当考虑未来发展需要。

第七条核酸检测实验室可分为试剂准备区、样本制备区、核酸扩增区和产物分析区。结合实际，采用集中布置或分散布置形式，并配套设置洗消设施。当采用实时荧光定量PCR仪时，核酸扩增区和产物分析区可合并为一区。当采用一体化自动化核酸分析设备时，样本制备区、核酸扩增区和产物分析区可合并为一区。

第八条检验实验室应结合工作流程和流线布局，做好导向、警示标识，确保出入流线清晰，安全警示到位。

第九条检验实验室入口处应设置标识，明确说明生物防护级别、操作的致病性生物因子、检验实验室负责人姓名、紧急联络方式和国际通用的生物危险符号；必要时，还应注明其他危险。

检验实验室所有房间的出口和紧急撤离路线应选用夜光标识。

第十条有静压差要求的检验实验室，应在合适位置设测压孔，并采用密封措施。在入口处宜安装空气压力显示装置，量程应与实验室静压差相匹配。需要时，可设置自动报警功能。

三、应急（临时）实验室围护结构

第十一条围护结构主体应防渗、防漏及密闭。采用轻质房屋时，荷载较大的设备应在首层布置。

第十二条应急需要的检测检验实验室，围护结构形式应因地制宜，选择方便快速加工、运输、安装，可考虑装配式轻型结构。轻质结构结合实际，考虑抗风措施，构件连接安全可靠。

四、通风与空气调节

第十三条检验实验室应根据房间功能、操作需求等合理确定新风量和换气次数，适用时可以利用自然通风。

检验实验室温度宜控制在18℃～26℃，相对湿度宜控制在30%～70%。对室内温湿度有特殊工艺要求的，室内温湿度参数应符合工艺要求。

第十四条空调冷热源的设置应确保全年正常运行。可采用集中或分散式空调冷热源，宜独立设置空调冷热源，当采用集中冷热源时宜设置备用冷热源。

第十五条采用机械通风系统时应避免交叉污染，排风应通过独立于建筑物其他公共通风系统的管道排出。

第十六条核酸检测实验室通风空调系统应保证各工作区的空气不产生交叉污染。

第十七条仪器设备相对集中、设备散热量较大的房间，应根据仪器设备运行功率及散热情况合理配置通风空调设备，考虑全年供冷的可能性。

第十八条凡涉及高危险性挥发物质或气体产生时，应在风险评估的基础上，配备适当的负压排风柜，排风机应设置在排风管路末端，室外排风应达到环保要求。核酸检测实验室的样本制备区宜设置ⅡA2型生物安全柜，当使用高危险有毒化学物质时应采用通风橱。

第十九条检验实验室新风应直接取自室外，新风口应设有粗效、中效二级过滤器，并应设置压差报警装置，提示清洗或更换过滤器，末端宜设置高效过滤送风口。新风口应远离排风口。

第二十条设置生物安全柜采用机械通风的检验实验室气流组织应符合定向气流原则，应有利于室内气流由被污染风险低的空间向被污染风险高的空间流动，最大限度减少室内回流与涡流。必要时，采用全新风直流式空调通风系统。

在生物安全柜操作面或其他有气溶胶操作地点的上方附近不应设送风口。

五、给水排水

第二十一条检验实验室应设置手卫生装置、洗眼装置，宜设置在靠近实验室出口处。手工检验使用的实验水池应根据专业要求合理设置，宜至少设置两个水池分别用于清洁、污洗，水池深度不宜小于200mm，以防止外溅。

第二十二条实验用水应满足以下要求：

1.水处理设备宜设置在单独房间内，供水管路材质应防腐、防锈，宜选用不锈钢材质。

2.水处理设备宜按照每小时最大用水量的1.5倍选型。

3.管路应设计为循环回路，尽可能减少拐弯，防止微生物滋生繁殖降低水质。

4.实验用水应符合相关工艺要求。

第二十三条检验实验室内部的给排水管道宜暗装敷设。给排水管道穿越墙壁、楼板时应加设套管，管道和套管之间应采取密封措施，无法设置套管时应采取有效的密封措施。

第二十四条检验实验室给水管不应与卫生器具、实验设备直接连接。应设置空气隔断或倒流防止器，并为后期检修、更换预留条件。

第二十五条当检验实验室内部设集中热水系统时，储热设备供热水温宜不宜低于60℃；循环系统供热水温不宜低于50℃。

第二十六条实验污水、生活污水系统应分别设置。实验污水做无害化处理后方可排入市政排水系统，并满足现行《医疗机构水污染物排放标准》GB18466的有关规定。

第二十七条当检验实验室内设置洁净室时，洁净区内不宜设置地漏；确需设置的，应采用专用密封地漏，且不应选用钟罩式和机械密封式。排水系统应采取防止水封破坏的措施。

六、电气及智能化

第二十八条检验实验室应保证用电的可靠性，用电负荷等级、自动恢复供电时间的确定应符合现行标准规定。当设置不间断电源（UPS）时，工作时间不宜小于30min。

第二十九条检验实验室应设置独立专用配电箱，除一级负荷及一级负荷中特别重负荷外，其余负荷配电回路应具备消防联动切断电源功能。低温冰箱、高温高压消毒锅、纯水机等有特殊用电要求的设备，宜单独设置配电箱。

第三十条检验实验室内应设置足够数量的固定电源插座。重要设备应采用单独回路配电，并设置漏电保护装置。

第三十一条检验实验室应有独立的有效接地系统。接地系统型式宜为TN-S或TN-C-S。有特殊要求时，应按实验仪器设备的具体要求确定。

第三十二条设置紫外线消毒灯具时，控制开关应设置在消毒区域之外，控制开关的面板形式或颜色宜区别于普通照明开关，安装高度宜距地1.8m以上，防止误操作。

第三十三条检验实验室室内环境控制系统的设置应根据区域需求确定。当有静压差要求时，应具有压力梯度、温湿度、连锁控制、报警等参数的历史数据存储显示功能，并预留接口。

第三十四条空调通风设备应能自动和手动控制，应急手动应有优先控制权，当实验室有静压差要求时，送排风系统应具备开关机连锁控制功能。

第三十五条检验实验室应配备适用的通讯设备。关键区域应设置监视器。条件允许的情况下，宜具备实时监视、录制功能。

七、运行维护

第三十六条有下列情况时，应对涉及生物安全的检验实验室设施设备进行综合性能检测，确保符合现行《实验室生物安全通用要求》GB19489、《生物安全实验室建筑技术规范》GB50346、《病原微生物实验室生物安全通用准则》WS233的有关规定。

1 .停止使用半年以上重新投入使用；

2 .空调机组进行大修或更换；

3.每年的定期维护检测；

4.高效过滤器更换后。

第三十七条检验实验室应定期消毒，并制定日常巡检制度，严格执行安全操作规程，并按时保质进行保养，确保隐患及时发现和排除。

1. 应对新型冠状病毒感染的肺炎疫情联防联控工作机制：国务院应对新型冠状病毒感染肺炎疫情联防联控机制关于印发《进一步推进新冠病毒核酸检测能力建设工作方案》的通知

国办发明电〔2020〕22号

做好有关实验室监督管理工作。各地要将医疗机构、疾控中心、第三方实验室等各类核酸检测机构纳入质控、质评体系统一管理，做好日常质控监管和不定期抽查等工作。要加强对各类检测实验室的统筹协调，做到接收样本数量与检测能力相匹配、检测流程规范、检测质量可靠、检测报告反馈及时。严格按照《病原微生物实验室生物安全管理条例》及相关技术规范要求开展实验活动，加强样本管理，防止实验室泄露或人员感染，保障实验室生物安全

1. 国务院应对新型冠状病毒肺炎疫情联防联控机制医疗救治组：关于印发《医学检验实验室管理暂行办法》的通知

联防联控机制医疗发〔2020〕279号

第一章 总则

第一条为加强对医学检验实验室的管理，提高医学检验水平，保证医疗质量和医疗安全，根据《基本医疗卫生与健康促进法》《执业医师法》《医疗机构管理条例》《病原微生物实验室生物安全管理条例》《医疗废物管理条例》《医疗器械监督管理条例》及《医疗机构临床实验室管理办法》等有关法律、法规，制定本办法。

第二条本办法所称医学检验实验室是指具有独立法人资质的医疗机构，以提供人类疾病诊断、管理、预防和治疗或健康评估的相关信息为目的，对来自人体的标本进行医学检验，包括临床血液与体液检验、临床化学检验、临床免疫检验、临床微生物检验、临床核酸和基因检验以及临床病理检查等，并出具检验结果。

第三条本办法适用于独立设置的对人类血液、体液、组织标本开展医学检验的医学检验实验室，不包括医疗机构内设的医学检验科。

第四条医学检验实验室应当履行医学检验工作的主体责任。医学检验应当遵循安全、准确、及时、有效、经济、便民和保护患者隐私的原则。

医学检验实验室应当制定完善的规章制度和流程规范，保证检验结果真实、准确、客观、公正，不受不当因素影响，不出具虚假或不符合规定的检验报告。

第二章 机构管理

第五条医学检验实验室应当制定并落实管理规章制度，执行国家制定颁布或者认可的技术规范和操作规程，明确工作人员岗位职责，落实实验室内感染预防、控制和改进的措施，保障医学检验工作安全、有效地开展。

第六条医学检验实验室应当设置独立实验室质量安全管理部门或配备专职人员，负责实验室质量管理与安全工作，履行以下职责：

（一）对规章制度、技术规范、操作规程的落实情况进行检查；

（二）对医疗质量、感染预防与控制、器械和设备管理、一次性医疗器械管理等方面进行检查；

（三）对重点环节，以及影响诊断质量和医疗安全的高危因素进行监测、分析和反馈，提出预防和控制措施；

（四）对工作人员的职业安全防护和健康管理提供指导；

（五）预防控制医学检验实验室的污染物外泄及感染；

（六）对医学检验实验室检测报告的书写、保存进行指导和检查，对病理检查病例的信息登记进行督查，并保障登记数据的真实性、及时性以及患者隐私。

（七）对试剂与耗材的存储部门、消毒供应等部门进行指导和监督检查，并提出质量改进意见和措施。

第七条医学检验实验室质量安全管理人员应当具有中级以上专业技术职务任职资格，具备相关专业知识和5 年以上工作经验。

第八条财务部门要对实验室业务费用和检验项目费用结算进行检查，并提出调控措施。

第九条后勤管理部门负责防火、治安等工作。

第三章 质量管理

第十条医学检验实验室应当遵循《医疗机构临床实验室管理办法》的要求，参考IS015189 《医学实验室质量和能力认可准则》，建立并运行医学检验质量管理体系，遵守相关技术规范和标准，落实分析前、分析中、分析后三个阶段的质量管理制度，包括医学检验项目的标准操作规程、检验仪器的标准操作与维护规程、性能验证或确认规程等，持续改进检验质量。

第十一条医学检验实验室可根据其他医疗卫生机构和执业医师提出的检验申请，接收其提供的标本或者直接采集受检者相关标本，并向申请者提供检验报告。受检者的经治医师负责对检验结果最终解释，必要时，医学检验实验室应当提供与检验结果相关的技术解释。

第十二条医学检验实验室接收或直接采集的标本数量应当与检验能力相匹配，建立检验需求超过自身服务能力的预案，避免标本数量明显超出检验能力导致的标本积压、标本失效、检测结果反馈迟缓等问题。

第十三条医学检验实验室应当具有分析前质量保证措施，制定患者准备、标本采集、标本储存、标本运送、标本接收等标准操作规程，应当定期评估标本质量，特别关注标本采集至送达实验室的时间是否符合要求。

第十四条医学检验实验室应当加强对分析中的管理， 规范医学检验活动，按有关规定开展室内质量控制，参加室间质量评价，保证检验结果公正性与准确性。

第十五条医学检验实验室应当开展分析后管理，采取有效措施保证检验活动的质量满足临床医疗的需求。应当对危急值、检验周转时间、检验结果准确性等质控指标进行监控。建立检测后标本、已发出报告标本的保留时限相关管理制度。制定报告召回的管理程序。

第十六条医学检验实验室应当建立医学检验报告发放制度，保证医学检验报告准确、及时和信息完整，并保护患者隐私。

第十七条医学检验报告应当使用中文或者国际通用的、规范的缩写。保存期限按照有关规定执行。

医学检验报告或诊断报告内容应当符合《病历书写基本规范》等规定，至少应当包括：

（一）检查单号、标本类型、临床诊断、检验方法、仪器型号、互认项目提示。

（二）患者姓名、性别、年龄、独立或其连锁经营医学检验实验室名称和地址、咨询电话。

（三）其他机构送检标本需注明送检机构名称、住院病历号或者门诊病历号。

（四）检验项目、检验结果和计量单位、参考区间（如适用）、危急值（如适用）、异常结果提示。

（五）检验者姓名、审核者姓名、标本采集时间、接收时间、报告时间。

（六）其他需要报告的内容和备注信息，并附检测局限性说明，必要时应报告与临床诊断相关重要信息。

第十八条医学检验实验室应当参加省级及以上医学检验室间质量评价活动。对于尚无室间质量评价的项目，应当建立与三级医疗机构医学检验科相同项目的比对方案，确定检验结果的可接受性，促进临床结果互认。

第十九条医学检验技术人员应当具有相关的专业学历，并取得相应专业技术职务任职资格和执业资格。

第二十条医学检验实验室应当对需要检定或校准的检验仪器设备，以及对医学检验结果有影响的辅助设备定期进行检定或校准。

第二十一条医学检验实验室应当建立满足服务质量要求的实验室信息系统，建立系统数据安全管理制度和应急措施。具备与所服务的机构信息系统联网的能力。

第二十二条医学检验实验室在与其他类别医疗机构等建立长期合作时，应当签订合同，明确双方在分析前、分析中和分析后以及检验结果所致医疗纠纷的责任、权利和义务。开展产前筛查与诊断的医学检验实验室只能与具有产前筛查与诊断资质的医疗机构开展合作。

第二十三条对于连锁经营的医学检验实验室，在保证生物安全和检验质量的前提下，可以在其符合相关资质的连锁经营的实验室之间进行标本的异地检测，并在检验报告中清晰标注实际检验实验室，便于出现差错时查找原因。

第四章 安全与感染防控

第二十四条医学检验实验室应当加强安全管理，强化感染预防与控制措施，建立并落实相关规章制度和工作规范，科学设置工作流程，降低发生感染的风险。保障检验服务的质量、安全，以及员工、患者和来访者的健康和安全。建立并严格遵守生物安全管理制度与安全操作规程。

第二十五条医学检验实验室应当设专人负责标本在实验室内部，以及其他机构与实验室之间传递过程的生物安全工作，包括生物安全培训以及相关设备耗材的管理等。

第二十六条医学检验实验室开展基因扩增、艾滋病检测、产前筛查与诊断、胚胎植入前遗传学筛查与诊断等特殊检验项目，应按照国家卫生健康委相关规定通过有关部门审核后，方可开展。

第二十七条医学检验实验室的建筑布局应当遵循环境卫生学和医疗机构感染防控的原则，符合功能流程合理和洁污区域分开的基本要求，做到布局合理、分区明确、标识清楚。

第二十八条医学检验实验室应当划分为医学检验功能区、辅助功能区和管理区。医学检验功能区包括接诊及标本接收区、标本采集区、标本准备区、标本检验区、试剂和耗材保存区、标本保存区、医疗废物处理区和医务人员办公区等基本功能区域；辅助功能区包括医疗费用结算区、供电区、纯水集中供应区和消毒供应室等；管理区包括病案、信息、实验室质量控制与安全管理部门等。

第二十九条标本采样区域应当达到《医院消毒卫生标准》中规定Ⅱ类环境标准。

第三十条医学检验实验室应当严格按照《病原微生物实验室生物安全管理条例》有关规定，加强对传染性疾病标本的采集、运输、储存、检验相关管理。医学检验功能区应达到生物安全II级标准。

第三十一条医学检验实验室应当按照《医疗废物管理条例》和《医疗卫生机构医疗废物管理办法》相关规定妥善处理医疗废物。

第三十二条医学检验实验室应当按照国家有关法规加强消防安全管理、信息安全管理。

第五章 人员培训与职业安全防护

第三十三条医学检验实验室应当制定并落实工作人员的岗前培训和轮岗培训计划，并进行考核，使工作人员具备与本职工作相关的专业知识，落实相关管理制度和工作规范。

第三十四条医学检验实验室应当对工作人员进行上岗前安全教育，每年进行生物安全防护知识培训。制定生物安全事故和危险品、危险设施等意外事故的预防措施和应急预案。

第三十五条医学检验实验室应当建立对技术人员的专业知识更新、专业技能维持与持续培养等管理的相关制度和记录。

第三十六条医学检验实验室应当按照生物防护级别配备必要的安全设备和个人防护用品，保证实验室工作人员能够正确使用。

第三十七条医学检验实验室应当加强实验室人员职业安全防护和健康管理工作，定期进行健康检查，必要时对有关人员进行免疫接种，保障医务人员的职业安全。

第三十八条医学检验实验室工作人员在工作中发生职业暴露事件时，应当采取相应的处理措施，并及时报告机构内的相关部门。

第三十九条医学检验实验室管理人员应当定期对实验室的危害因子和安全风险进行评估，确保实验室安全。定期举行实验室生物安全和消防安全演练并形成记录。

第六章 监督管理

第四十条县级以上卫生健康行政部门应当对辖区内医学检验实验室的管理、质量与安全等情况进行日常监督检查，发现存在质量问题或者安全隐患时，应当责令其立即整改。整改未达到要求的，在行业内进行通报批评。

第四十一条县级以上卫生健康行政部门接到对医学检验实验室的举报、投诉后，应当及时核查并依法处理。

第四十二条县级以上卫生健康行政部门履行监督检查职责时，有权采取下列措施：

（一）对开展的医学检验活动进行现场检查，了解情况，调查取证；

（二）查阅或者复制医学检验活动质量和安全管理的有关资料，采集、封存样品；

（三）责令违反本办法及有关规定的机构停止违法违规行为；

（四）对违反本办法及有关规定的行为进行查处。

第四十三条县级以上卫生健康行政部门应当加强对医学检验实验室医疗机构执业许可证的校验管理，将日常监督检查的结果与校验工作挂钩。对于有严重违规行为或多起违规行为的，医疗机构执业许可证不予校验。

第四十四条医学检验实验室未进行医学检验诊疗科目登记而开展医学检验服务的，按照《医疗机构管理条例 》第四十七条处罚。

第四十五条使用非卫生技术人员从事医学检验工作的，按照《医疗机构管理条例》第四十八条处罚。

第四十六条出具虚假检验报告的医学检验实验室，按照《医疗机构管理条例》第四十九条处罚。对出具虚假检验报告的医师，按照《执业医师法》第三十七条处罚。

第四十七条室间质量评价连续两次以上不合格，经整改后仍不合格的，由卫生健康行政部门进行公告。未开展室内质量控制或未参加室间质量评价的，医疗机构执业许可证不予校验。

第四十八条出现其他违反《医疗机构管理条例》及《医疗机构管理条例实施细则》的，由卫生健康行政部门依法依规从严从重处理。

1. 国务院应对新型冠状病毒肺炎疫情联防联控机制医疗救治组：关于印发《医疗机构新型冠状病毒核酸检测工作手册（试行）》的通知

联防联控机制医疗发〔2020〕271号

为落实国务院应对新型冠状病毒感染肺炎疫情联防联控机制《关于做好新冠肺炎疫情常态化防控工作的指导意见》（国发明电〔2020〕14号）要求，进一步规范新型冠状病毒（以下简称新冠病毒）核酸检测的技术人员、标本采集、标本管理、实验室检测、结果报告等工作，保证检测质量，提高检测效率，满足新冠病毒核酸检测需求，特制定本手册。本手册适用于所有开展新冠病毒核酸检测的医疗机构。

一、技术人员基本要求

（一）采样人员。从事新冠病毒核酸检测标本采集的技术人员应当经过生物安全培训（培训合格），熟悉标本种类和采集方法，熟练掌握标本采集操作流程及注意事项，做好标本信息的记录，确保标本质量符合要求、标本及相关信息可追溯。

（二）检测人员。实验室检测技术人员应当具备相关专业的大专以上学历或具有中级及以上专业技术职务任职资格，并有2年以上的实验室工作经历和基因检验相关培训合格证书。实验室配备的工作人员应当与所开展检测项目及标本量相适宜，以保证及时、熟练地进行实验和报告结果，保证结果的准确性。

二、标本采集基本要求

（一）基本原则。

1.各医疗机构的检测能力应当与门急诊就诊人次、住院人次等诊疗量相匹配，并与采集的标本量相适应，避免采集数量明显超出检测能力导致的标本积压、标本失效、检测结果反馈迟缓等问题。

2.各医疗机构在采集标本时，要根据不同采集对象设置不同的采样区域，将发热患者与其他患者、“愿检尽检”人群分区采样，避免交叉感染。

3.标本采集应当在满足本机构发热门诊、住院患者、陪护人员及院内职工的检测需求基础上，进一步保障其他重点人群“应检尽检”和一般人群“愿检尽检”的要求。

（二）采样点设置。医疗机构设置新冠病毒采样点应当遵循安全、科学、便民的原则。采样点应当为独立空间，具备通风条件，内部划分相应的清洁区和污染区，配备手卫生设施或装置。采样点需设立清晰的指引标识，并明确采样流程和注意事项。设立独立的等候区域，尽可能保证人员单向流动，落实“1米线”间隔要求，严控人员密度。

（三）人员配置及防护要求。每个采样点应当配备1-2名采样人员。合理安排采样人员轮替，原则上每2-4小时轮岗休息1次。采样人员防护装备要求：N95及以上防护口罩、护目镜、防护服、乳胶手套、防水靴套；如果接触患者血液、体液、分泌物或排泄物，戴双层乳胶手套；手套被污染时，及时更换外层乳胶手套。每采一个人应当进行严格手消毒或更换手套。

（四）采样流程。各医疗机构应当建立新冠病毒核酸检测采样操作流程制度，根据采样对象类别确定具体采样流程，包括预约、缴费、信息核对、采样、送检、报告发放等。应当利用条码扫描等信息化手段采集受检者信息。标本采集前，采样人员应当对受检者身份信息进行核对，并在公共区域以信息公告形式告知核酸检测报告发放时限和发放方式。每个标本应当至少记录以下信息：1.受检者（患者）姓名、身份证号、居住地址、联系方式；2.采样单位名称、标本编号，标本采集的日期、时间、采集部位、类型、数量等。

（五）采集方法。应当采集呼吸道标本，包括上呼吸道标本（口咽拭子、鼻咽拭子等）或下呼吸道标本（呼吸道吸取物、支气管灌洗液、肺泡灌洗液、深咳痰液等）。其中，重症病例优先采集下呼吸道标本；根据临床需要可留取便标本。

1.口咽拭子。被采集人员先用生理盐水漱口，采样人员将拭子放入无菌生理盐水中湿润(禁止将拭子放入病毒保存液中，避免抗生素引起过敏)，被采集人员头部微仰，嘴张大，并发“啊”音，露出两侧咽扁桃体，将拭子越过舌根，在被采集者两侧咽扁桃体稍微用力来回擦拭至少3次，然后再在咽后壁上下擦拭至少3次，将拭子头浸入含2～3ml病毒保存液（也可使用等渗盐溶液、组织培养液或磷酸盐缓冲液）的管中，尾部弃去，旋紧管盖。

2.鼻咽拭子。采样人员一手轻扶被采集人员的头部，一手执拭子贴鼻孔进入，沿下鼻道的底部向后缓缓深入，由于鼻道呈弧形，不可用力过猛，以免发生外伤出血。待拭子顶端到达鼻咽腔后壁时，轻轻旋转一周（如遇反射性咳嗽，应停留片刻），然后缓缓取出拭子，将拭子头浸入含2～3ml病毒保存液的管中。

3.深咳痰液。要求患者深咳后，将咳出的痰液收集于含3ml采样液的50ml螺口塑料管中。如果痰液未收集于采样液中，可在检测前，加入2～3ml采样液，或加入痰液等体积的痰消化液。可以采用痰液等体积的含1g/L蛋白酶K的磷酸盐缓冲液将痰液化。

4.鼻咽或呼吸道抽取物。用与负压泵相连的收集器从鼻咽部抽取粘液或从气管抽取呼吸道分泌物。将收集器头部插入鼻腔或气管，接通负压，旋转收集器头部并缓慢退出，收集抽取的粘液，并用3ml采样液冲洗收集器1次（亦可用小儿导尿管接在50ml注射器上来替代收集器）。

5.支气管灌洗液。将收集器头部从鼻孔或气管插口处插入气管（约30cm深处），注入5ml生理盐水，接通负压，旋转收集器头部并缓慢退出。收集抽取的粘液，并用采样液冲洗收集器1次（亦可用小儿导尿管接在50ml注射器上来替代收集）。

6.肺泡灌洗液。局部麻醉后将纤维支气管镜通过口或鼻经过咽部插入右肺中叶或左肺舌段的支管，将其顶端契入支气管分支开口，经气管活检孔缓缓加入灭菌生理盐水，每次30～50ml，总量100～250ml，不应超过300ml。

三、标本管理基本要求

（一）标本包装。所有标本应当放在大小适合的带螺旋盖内有垫圈、耐冷冻的标本采集管里，拧紧。容器外注明标本编号、种类、姓名及采样日期。将密闭后的标本放入大小合适的塑料袋内密封，每袋装一份标本。

（二）标本送检。标本采集后室温放置不超过4小时，应在2-4h内送到实验室。如果需要长途运输标本，应采用干冰等制冷方式进行保存，严格按照相关规定包装运输。

（三）标本接收。标本接收人员的个人防护按采样人员防护装备执行。标本运送人员和接收人员对标本进行双签收。

（四）标本保存。用于病毒分离和核酸检测的标本应当尽快进行检测，能在24小时内检测的标本可置于4℃保存；24小时内无法检测的标本则应置于-70℃或以下保存（如无-70℃保存条件，则于-20℃冰箱暂存）。应当设立专库或专柜单独保存标本。标本运送期间避免反复冻融。

（五）混检标本的采集和检测。

1.采样方法。按本手册“第二部分（五）采集方法”进行样本采集。

2.样本混合。将采集的数个样本（原则上不超过5个）各取200ul进行充分混合，形成混合待检样本。

3.混检样本的结果判断与重测。检测结果先按照各检测试剂盒使用说明书的要求，对被检测基因位点逐一判定，出现任一位点阳性或能测出Ct值的混合样本，均作为重测对象，按照常规方法进行单样本核酸检测。

四、实验室管理基本要求

（一）实验室资质要求。开展核酸检测的实验室，应当符合《病原微生物实验室生物安全管理条例》（国务院令第424号）和《医疗机构临床基因扩增检验实验室管理办法》（卫办医政发〔2010〕194号）有关规定，具备经过卫生健康行政部门审核备案的生物安全二级及以上实验室条件，以及临床基因扩增检验实验室条件。独立设置的医学检验实验室还应当符合《医学检验实验室基本标准（试行）》《医学检验实验室管理规范（试行）》等要求。

（二）实验室分区要求。原则上开展新冠病毒核酸检测的实验室应当设置以下区域：试剂储存和准备区、标本制备区、扩增和产物分析区。这3个区域在物理空间上应当是完全相互独立的，不能有空气的直接相通。各区的功能是：

1.试剂储存和准备区：贮存试剂的制备、试剂的分装和扩增反应混合液的制备，以及离心管、吸头等消耗品的贮存和准备。

2.标本制备区：转运桶的开启、标本的灭活，核酸提取及其加入至扩增反应管等。

3.扩增和产物分析区：核酸扩增和产物分析。

根据使用仪器的功能，区域可适当合并。如采用标本处理、核酸提取及扩增检测为一体的自动化分析仪，标本制备区、扩增和产物分析区可合并。

（三）主要仪器设备。实验室应当配备与开展检验项目相适宜的仪器设备，包括核酸提取仪、医用PCR扩增仪、生物安全柜、病毒灭活设备（如水浴锅等）、保存试剂和标本的冰箱和冰柜、离心机、不间断电源（UPS）或备用电源等。

（四）实验室检测。实验室接到标本后，应当在生物安全柜内对标本进行清点核对，并对标本进行灭活处理。按照标准操作程序进行试剂准备、标本前处理、核酸提取、核酸扩增、结果分析及报告。实验室应当建立可疑标本和阳性标本复检的流程。

1.试剂准备。应当选择国家药品监督管理部门批准的试剂，并在选择标本保存液和核酸提取试剂时，使用试剂盒说明书上建议的配套试剂。核酸提取方法与标本保存液和灭活方式相关，有些核酸提取试剂（如磁珠法或者一步法），容易受到胍盐或保存液中特殊成分的影响，特别是一步法提取多需要使用试剂厂家配套的标本保存液。

2.标本前处理。已经使用含胍盐的灭活型标本保存液的实验室，这一环节无需进行灭活处理，直接进行核酸提取，而使用非灭活型标本保存液的实验室，则有56℃孵育30分钟热灭活、化学灭活的处理方式。

3.核酸提取。将灭活后的标本取出，在生物安全柜内打开标本采集管加样。核酸提取完成后，立即将提取物进行封盖处理。在生物安全柜内将提取核酸加至PCR扩增反应体系中。

4.核酸扩增。将扩增体系放入扩增仪，核对扩增程序是否与试剂说明书相符，启动扩增程序。扩增后产物置于含1 mol/L的盐酸中，应将产物全部浸泡至液体中，浸泡后将扩增产物转移出实验室。

五、实验室质量控制与管理

各医疗机构应当加强核酸检测质量控制。实验室应对检测体系进行必要的性能验证，性能指标包括但不限于精密度和最低检测限。实验室要做好日常室内质控，按照《国家卫生健康委办公厅关于医疗机构开展新型冠状病毒核酸检测有关要求的通知》（国卫办医函〔2020〕53号）要求规范开展室内质控。应常态化接受国家级或省级检验质量控制。不按照本地要求参加室间质评的，或室间质评结果不合格的，或检测结果质量问题突出的，不得开展核酸检测。

质量管理包括人员的操作技能和生物安全培训，标本采集、运输、接收和保存，耗材的管理，试剂的选择和性能验证，质控品的合理设置及其有效判定，复检规则，结果的报告、解释与建议等。

六、核酸检测结果反馈基本要求

（一）报告时限。对于发热门诊、急诊患者，在6小时内报告核酸检测结果；对于普通门诊、住院患者及陪护人员等人群，原则上在12小时内报告结果；对于“愿检尽检”人群，一般在24小时内报告结果。医疗机构应当为受检者出具检测报告，并告知其查询方式，不得以任何理由不出具检测报告。

（二）检测报告。各医疗机构应当按照《新型冠状病毒核酸检测报告单》的参考样式出具检测报告（见附件），在卫生健康行政部门的规定下，互认检测结果。医疗机构可采用纸质、快递、网络或信息化系统等多种形式，发放核酸检测报告，并注意保护个人隐私。发现核酸检测阳性结果时应按相关要求在12小时内进行传染病上报及后续流行病学调查工作。

七、核酸检测安全管理

（一）标本安全管理。标本转运箱封闭前，须使用75%酒精或0.2%含氯消毒剂喷洒消毒。标本包装应符合国际民航组织文件Doc9284《危险品航空安全运输技术细则》的PI602分类包装要求。涉及外部标本运输的，应按照A类感染性物质进行三层包装。疑似或确诊患者标本应标示有特殊标识，并进行单独转运。检测完成后标本，若检测结果为阴性，剩余标本及核酸可在结果报告发出24小时后装入专用密封废物转运袋中进行压力蒸汽灭菌处理，随后随其他医疗废物一起转运出实验室进行销毁处理；若检测结果为阳性，剩余标本应进行复核检测。

（二）实验室检测安全管理。

1.基本要求。标本灭活及检测应当在生物安全二级实验室进行，同时采用生物安全三级实验室的个人防护。开展新冠病毒核酸检测的实验室应当制定实验室生物安全相关程序文件及实验室生物安全操作失误或意外的处理操作程序，并建立实验室环境消毒处理记录。

2.实验前安全要求。应使用0.2%含氯消毒剂或75%酒精进行桌面、台面及地面消毒。消毒液需每天新鲜配制，不超过24小时。转运至实验室的标本转运桶应在生物安全柜内开启。转运桶开启后，使用0.2%含氯消毒剂或75%酒精对转运桶内壁和标本采集密封袋进行喷洒消毒。取出标本采集管后应首先检查标本管外壁是否有破损、管口是否泄露或有管壁残留物。确认无渗漏后，推荐用0.2%含氯消毒剂喷洒、擦拭消毒样品管外表面（此处不建议使用75%酒精，以免破坏样品标识）。如发现渗漏应立即用吸水纸覆盖，并喷洒有效氯含量为0.55%的含氯消毒剂进行消毒处理，不得对标本继续检测操作，做好标本不合格记录后需立即进行密封打包，压力蒸汽灭菌处理后销毁。

实验室操作人员在进行标本热灭活时，温浴前需旋紧标本采集管管盖，必要时可用封口膜密闭管盖；温浴过程中可每隔10分钟将标本轻柔摇匀1次，以保证标本均匀灭活；温浴后标本需静置至室温或至少10min使气溶胶沉降，随后再开盖进行后续核酸提取。

3.核酸提取和检测安全要求。标本进行核酸提取和检测时应尽可能在生物安全柜内进行操作。如为打开标本管盖或其他有可能产生气溶胶的操作，则必须在生物安全柜内进行。

4.实验结束后安全要求。需对实验室环境进行清洁消毒。

（1）实验室空气消毒。实验室每次检测完毕后应进行房间紫外消毒30分钟或紫外消毒机照射消毒1小时。必要时可采用核酸清除剂等试剂清除实验室残留核酸。

（2）工作台面消毒。每天实验后，使用0.2%含氯消毒剂或75%酒精进行台面、地面消毒。

（3）生物安全柜消毒。实验使用后的耗材废弃物放入医疗废物垃圾袋中，包扎后使用0.2%含有效氯消毒液或75%酒精喷洒消毒其外表面。手消毒后将垃圾袋带出生物安全柜放入实验室废弃物转运袋中。试管架、实验台面、移液器等使用75%酒精进行擦拭。随后关闭生物安全柜，紫外灯消毒30分钟。

（4）转运容器消毒。转运及存放标本的容器使用前后需使用0.2%含氯消毒剂或75%酒精进行擦拭或喷洒消毒。

（5）塑料或有机玻璃材质物品消毒：使用0.2%含氯消毒剂或过氧乙酸或过氧化氢擦拭或喷洒消毒。

（三）实验室医疗废物管理。

1.基本要求。开展新冠病毒核酸检测的实验室应当制定医疗废物处置程序及污物、污水处理操作程序。所有的危险性医疗废物必须按照统一规格化的容器和标示方式，完整且合规地标示废物内容。应当由经过适当培训的人员使用适当的个人防护装备和设备处理危险性医疗废物。实验室应建立医疗废物处理记录，定期对实验室排风HEPA过滤器进行捡漏和更换，定期对处理后的污水进行监测，采用生物指示剂监测压力灭菌效果。

2.医疗废物的处理措施。医疗废物的处理是控制实验室安全的关键环节，必须充分掌握生物安全废弃物的分类，并严格执行相应的处理程序。

（1）废液的处理。实验室产生的废液可分为普通污水和感染性废液。普通污水产生于洗手池等设备，对此类污水应当排入实验室水处理系统，经统一处理达标后进行排放。感染性废液即在实验操作过程中产生的废液，需采用化学消毒（0.55%含氯消毒剂处理）或物理消毒（紫外照射30分钟以上）方式处理，确认彻底消毒灭活后方可排入实验室水处理系统，经统一处理达标后进行排放。污水消毒处理效果按GB18466《医疗机构水污染物排放标准》相关规定进行评价。

（2）固体废物的处理。实验室固体废物应当分类收集。固体废物的收集容器应当具有不易破裂、防渗漏、耐湿耐热、可密封等特性。实验室内的潜在感染性废物不允许堆积存放，应当及时进行压力蒸汽灭菌处理。废物处置之前，应当存放在实验室内指定的安全位置。小型固体废物如检测耗材、个人防护装备等均需使用双层防渗漏专用包装袋打包密封后经过压力蒸汽灭菌处理，再沿医疗废物通道转运出实验室。

体积较大的固体废物如HEPA过滤器，应当由专业人士进行原位消毒后，装入安全容器内进行消毒灭菌。不能进行压力蒸汽灭菌的物品如电子设备可采用环氧乙烷熏蒸消毒处理。经消毒灭菌处理后移出实验室的固体废物需集中交由医疗废物处理单位进行处置。

（四）实验室污染的处理。

1.标本污染生物安全柜的操作台造成局限污染时：立即用吸水纸覆盖，并使用0.55%含氯消毒剂进行喷洒消毒。消毒液需要现用现配，24小时内使用。

2.标本倾覆造成实验室污染时：保持实验室空间密闭，避免污染物扩散。立即使用润湿有0.55%含氯消毒剂的毛巾覆盖污染区。必要时（如大量溢撒时）可用过氧乙酸加热熏蒸实验室，剂量为2g/m3，熏蒸过夜；或20g/L过氧乙酸消毒液用气溶胶喷雾器喷雾，用量8ml/m3，作用1-2小时；必要时或用高锰酸钾-甲醛熏蒸：高锰酸钾8g/m3，放入耐热耐腐蚀容器（陶罐或玻璃容器），后加入甲醛（40%）10ml/m3，熏蒸4小时以上。熏蒸时室内湿度60%-80%。

3.清理污染物时严格遵循活病毒生物安全操作要求，采用压力蒸汽灭菌处理，并进行实验室换气等，防止次生危害。

八、核酸检测信息化管理

医疗机构应当在卫生健康行政部门统筹下，做好标本采集、核酸检测、检测报告的信息对接工作。建立统一的信息采集扫码程序，信息应至少包括姓名、性别、年龄、身份证号、联系电话，做到标本采集的个人信息与医疗机构信息系统顺利对接，各医疗机构间应做到信息互通、互采、互认。

九、其他要求

因疫情防控需要等因素，医疗机构采集的标本量明显超出自身检测能力范围的，可以建立医疗机构间新冠病毒核酸检测协作机制，分散检测压力，保证时效性和有效性。

1. 国家卫生健康委员会：国家卫生健康委办公厅关于在新冠肺炎疫情常态化防控中进一步加强实验室生物安全监督管理的通知

国卫办科教函〔2020〕534号

为落实《国务院应对新型冠状病毒感染肺炎疫情联防联控机制关于做好新冠肺炎疫情常态化防控工作的指导意见》（国发明电〔2020〕14号，以下简称《指导意见》）要求，确保疫情防控期间实验室生物安全，防范发生次生灾害，现就做好实验室生物安全监督管理工作提出如下要求：

一、严格执行新型冠状病毒实验活动管理要求

根据新型冠状病毒(以下简称新冠病毒)传播特性、致病性和临床资料等信息，该病毒按照第二类病原微生物进行管理。各地卫生健康行政部门应当要求生物安全实验室严格按照防护要求开展相关实验活动：新冠病毒培养、动物感染实验应当在生物安全三级及以上实验室开展；未经培养的感染性材料的操作应当在生物安全二级及以上实验室进行，同时采用不低于生物安全三级实验室的个人防护；灭活材料的操作应当在生物安全二级及以上实验室进行；不涉及感染性材料的操作，可以在生物安全一级实验室进行。

二、做好实验室生物安全服务保障和规范管理

各地卫生健康行政部门要做好检测实验室备案管理工作，压实实验室设立单位主体责任，督促相关单位加强实验操作技术、个人防护、检测样本处置等方面的培训与考核，规范实验人员新冠病毒样本检测操作流程，提升检测能力，保障检测人员和周围环境安全。

三、加强新冠病毒毒株及相关样本管理

各地卫生健康行政部门要依法依规严格管理新冠病毒毒株和相关样本，确保安全。

（一）新冠病毒毒株及样本运输。新冠病毒毒株及潜在感染性材料运输应当按照《可感染人类的高致病性病原微生物菌（毒）种或样本运输管理规定》管理。各地根据疫情防控形势需要，在运输环节中，对“应检尽检”人员检测样本严格按照高致病性病原微生物样本管理；对“愿检尽检”人员检测样本，经样本运出单位生物安全专家委员会进行风险评估后，可按照普通样本管理。“应检尽检”和“愿检尽检”人员范围按照《指导意见》执行。各省级卫生健康行政部门要加强对毒株及相关样本保存单位的监督管理，严格防范和杜绝未经审批擅自运输的情况发生。

（二）相关样本处置。各省级卫生健康行政部门要根据疫情防控需要和实验室生物安全有关要求，及时研判提出新冠病毒实验室检测生物样本处置意见。对确需保存的，应当尽快指定具备保存条件的机构按照相对集中原则进行保存，或送至国家级菌（毒）种保藏中心保藏；对无需保存的，由相关机构按照生物安全有关要求及时处理。

（三）毒株分离和保藏。请各省级卫生健康行政部门督促辖区内高等级生物安全实验室将新冠病毒毒株分离、分享等相关情况及时报送我委科教司，同时指导实验室在分离出新冠病毒毒株后90天内，向国家级菌（毒）种保藏中心申请保藏，完成相关实验活动后及时将新冠病毒毒株送交保藏机构保藏。请各省级卫生健康行政部门在办理有关实验室和各级菌（毒）种保藏机构向其他实验室或外单位提供新冠病毒毒株或以新冠病毒作为母本病毒的疫苗株的准运手续时，及时将准运证书复印件提供给我委科教司。

四、加强实验室生物安全监管

各省级卫生健康行政部门要切实加强组织领导，提升实验室生物安全监管能力，按照属地化、分级分类的原则开展实验室生物安全监管工作，强化新冠病毒实验活动监督检查，指导辖区内相关机构加强生物安全管理，严格按照《病原微生物实验室生物安全管理条例》及相关技术规范要求开展实验活动，防止实验室泄露或人员感染，确保实验室生物安全万无一失。

1. 国务院应对新型冠状病毒肺炎疫情联防联控机制医疗救治组：关于做好疫情常态化防控下新冠病毒核酸检测质量控制工作的通知

联防联控机制医疗发〔2020〕242号

二、加强实验室备案或准入管理

开展核酸检测的实验室，应当符合《病原微生物实验室生物安全管理条例》（国务院令第424号）和《医疗机构临床基因扩增检验实验室管理办法》（卫办医政发〔2010〕194号）有关规定，并在相应的卫生健康行政部门进行登记备案，具备生物安全二级及以上实验室条件以及PCR实验室条件。医学检验实验室（常称为第三方实验室）还应当符合《医学检验实验室基本标准（试行）》《医学检验实验室管理规范（试行）》等要求。

三、加强实验室检测质量控制

各地应当加强核酸检测质量控制，将开展核酸检测的医疗机构（含医学检验实验室，下同）和疾控机构实验室统一纳入质量控制体系。实验室要做好日常室内质控，并常态化接受国家级或省级检验质量控制。各省级卫生健康行政部门要加强对核酸检测实验室的日常质量控制工作，并组织实验室分批参加室间质评，保证短期内每个实验室至少参加1次室间质评并合格。检测结果质量问题突出的，或室间质量评价不合格的，不得开展核酸检测。

四、加强核酸检测人员培训

各地要按照《新型冠状病毒肺炎实验室检测技术指南》要求，加强医疗机构、疾控机构核酸检测相关人员的技术培训和指导，覆盖标本的采集、保存、运输、处理和检测等各个环节，最大限度减少产生假阴性的因素。对标本采集和处理等重点环节，加大人员培训力度，确保采样人员规范采集各类标本，实验室检测人员要熟练掌握标本处理和检测方法，开展检测时做好生物安全防护。

1. 应对新型冠状病毒感染的肺炎疫情联防联控工作机制：关于进一步做好疫情期间新冠病毒检测有关工作的通知

联防联控机制综发〔2020〕152号

五、加强实验室生物安全管理，做好剩余样本处理

各地开展新冠病毒检测应当符合《病原微生物实验室生物安全管理条例》（国务院令第424号）有关规定，并执行《新型冠状病毒实验室生物安全指南（第二版）》（国卫办科教函〔2020〕70号）要求，切实加强生物安全管理。各省级卫生健康行政部门要结合实际情况研究提出本区域实验室剩余生物样本处置意见，医疗机构和疾控机构要落实主体责任，做好样本的使用、保存与销毁等工作。

1. 财政部、国家卫生健康委员会、国家医疗保障局、国家中医药管理局：关于印发《关于进一步加强公立医院内部控制建设的指导意见》的通知

财会〔2023〕31号

加强生物安全管理，规范生物医学新技术临床研究管理，强化实验室生物安全风险管控，加强人类遗传资源采集、保藏、利用、对外提供等活动的管理和监督，健全生物安全相关管理制度，筑牢公立医院生物安全防线。

1. 农业农村部：农业农村部办公厅关于加强国家非洲猪瘟相关实验室管理的通知

农办牧〔2019〕41号

进一步加强相关实验室管理

各相关实验室及实验室所在单位要按照《病原微生物实验室生物安全管理条例》等有关法律法规和我部相关规定开展非洲猪瘟病毒实验工作。一经发现疑似或确诊非洲猪瘟疫情，应立即按照规定报告。各相关实验室在使用具有知识产权的菌（毒）种和样本时，应当经原提供者或持有人的同意。

各级畜牧兽医主管部门要切实履行好对相关实验室的监管职责，对相关实验室实验活动实行全程监管。一是加强非洲猪瘟病毒相关实验活动初审，把好审批关。对未经批准从事非洲猪瘟病毒相关实验活动的，要依法严肃查处，对由此产生的科研成果均不予认可。二是督促落实非洲猪瘟病毒相关实验活动承诺制度和报告制度。三是定期组织开展考核评估和监督检查，督促各相关实验室加强内部管理，制定并严格落实生物安全管理、安全防护、感染控制和生物安全应急预案等规章制度。

1. 国务院应对新型冠状病毒肺炎疫情联防联控机制综合组：关于印发医疗机构内新型冠状病毒感染预防与控制技术指南(第三版)的通知

联防联控机制综发〔2021〕96号

严格落实实验室生物安全制度，规范新冠病毒核酸检测相关操作，实验室工作人员做好个人防护，规范处置医疗废物。

1. 农业农村部：农业部办公厅关于印发《2018年兽医工作要点》的通知

农办医〔2018〕5号

强化实验室建设与管理。加强兽医实验室体系建设，完善兽医实验室动态管理制度，提高动物疫病检测诊断能力。推动建立各级各类兽医实验室合作共享和开放交流机制。开展兽医实验室生物安全专项检查。规范兽医实验室生物安全审批，做好事中事后监管工作

1. 国务院应对新型冠状病毒肺炎疫情联防联控机制综合组：关于印发《全员新型冠状病毒核酸检测组织实施指南（第二版）》的通知

联防联控机制综发〔2021〕97号

2.医疗废物的处理措施。医疗废物的处理是控制采集场所和检测实验室生物安全的关键环节，必须充分掌握涉及生物安全的相关分类，并严格执行相应的处理程序。

1. 国家中医药管理局：关于印发《中医医疗机构传染病防治和感染防控监督执法专项检查方案》的通知

国中医药办法监发〔2017〕30号

（一） 组织和管理

监督检查是否按照相关法律法规要求建立完善的组织管理体系、明确部门工作职责和人员配备、制定有关规章制度及预案以及开展培训等。

（二）疫情防控和血制品管理

监督检查传染病疫情登记、报告卡填写情况;隐瞒、谎报、缓报传染病疫情情况;对传染病病人和疑似传染病病人的消毒隔离落实情况;因应急用血而有临时采集血液行为，进行艾滋病检测情况；采集、使用人体组织、器官、细胞、骨髓进行艾滋病检测情况。

（三）消毒隔离制度落实

监督检查消毒管理制度落实、布局流程、医疗用品和器械的消毒与灭菌、一次性医疗器械用品的使用、隔离防护、消毒与灭菌效果检测以及消毒产品进货检查验收使用和管理等情况;中医诊疗有关技术、设备使用是否符合消毒隔离要求，重点检查针刺类、微创类、刮痧类、拔罐类、敷熨熏浴类、灌肠类、灸类等中医特色诊疗器械的消毒灭菌情况。

（四）医疗废物管理

监督检查医疗废物实行分类收集情况；使用专用包装物及容器情况，特别是针刺类器械、破损的拔罐类器械是否置于利器盒内，废弃的敷熨熏浴类物品是否按照感染性废物处置等；医疗废物暂时贮存设施建立情况；医疗废物交接、运送、暂存及处置情况；医院污水消毒处理情况，相关指标定期监测情况。

（五）病原微生物实验室安全管理

监督检查一、二级实验室备案情况，三、四级实验室取得高致病性病原微生物实验活动的资格证书情况；从事实验活动的人员资质；有关操作规程执行情况；实验档案建立和保存情况；实验结束将菌（毒）种或样本销毁或者送交保藏机构保藏情况。

1. 国家发展和改革委员会、科技部：关于印发《高级别生物安全实验室体系建设规划（2016—2025年）》的通知

发改高技〔2016〕2361号

高级别生物安全实验室是指生物安全防护级别为三级和四级的生物安全实验室。为进一步提高我国高级别生物安全实验室体系的建设水平，增强生物安全科技自主创新能力，根据《病原微生物实验室生物安全管理条例》、《国务院关于加强传染病防治人员安全防护的意见》有关要求制定本规划。

一、体系建设的必要性和重要性

高级别生物安全实验室是国家生物安全体系的基础支撑平台，是人口健康与动物卫生领域开展科研、生产和服务的重要保障条件。2004 年《国家高级别生物安全实验室建设规划》发布以来，我国已经形成了高级别生物安全实验室体系的基本框架，一批生物安全三级实验室（以下简称“三级实验室”）投入运行，建成了若干生物安全四级实验室（以下简称“四级实验室”），为我国的烈性与重大传染病防控、生物防范和产业发展做出了重要贡献。随着国际生物安全形势日趋复杂多变，我国战略性新兴产业蓬勃发展，实验室需求日益增加，原有的建设布局和管理能力已经不能完全适应新形势的需要。在整体布局方面，用于科研的实验室相对较多，产业和特殊领域的实验室数量不足，区域布局不均衡；在组织协调方面，全国性协调管理和资源共享机制有待完善，统筹管理、快速反应和临机决策能力亟需加强；在经费投入方面，缺乏长期稳定的建设、运行维护投入，不利于实验室体系的可持续发展。因此要充分把握新形势下经济社会发展的新要求，加快建设合理布局、功能完善、统筹管理、高效运行的国家高级别生物安全实验室网络体系。

二、指导思想和原则

（一）指导思想

贯彻落实总体国家安全观，面向医药人口健康、动物卫生、检验检疫、生态环境安全四大领域，针对微生物菌种保藏、科学研究、产业转化三大主体功能，围绕烈性、突发、外来、热带传染病病原体的监测预警、检测、消杀、防控、治疗五大环节的需求，按照“统筹布局，网络运行；应急优先，稳步推进；加强协调，科学管理” 的原则，统筹全国高级别生物安全实验室整体布局，为保障我国生物安全提供重要支撑。

（二）建设原则

——统筹布局，网络运行。统筹高级别生物安全实验室的整体布局，调控增量、激活存量，既要充分满足当前需求，又要为未来的发展留有余地；既要满足区域对实验室的建设需求，又要根据全国一盘棋的思路形成高效运行的实验室网络。

——应急优先，稳步推进。围绕国家战略需求和生物安全科技发展趋势，根据我国周边安全环境和我国区域特点、行业特点，优先建设实验室应急保障能力，逐步完善国家高级别生物安全实验室体系，满足国家生物安全科研和应用需要。

——加强协调，科学管理。完善国家高级别生物安全实验室管理协调机制，加强实验室管理的政策法规制度建设，进一步明确实验室的建设、运行、维护的相关标准和准入条件，强化事中和事后监管，保障实验室体系健康发展。

三、发展目标

到 2025 年，形成布局合理、网络运行的高级别生物安全实验室国家体系。

一是建成我国高级别生物安全实验室体系。按照区域分布、功能齐备、特色突出的原则，形成 5-7 个四级实验室建设布局。在充分利用现有三级实验室的基础上，新建一批三级实验室（含移动三级实验室），实现每个省份至少设有一家三级实验室的目标。以四级实验室和公益性三级实验室为主要组成部分，吸纳其他非公益三级实验室和生物安全防护设施，建成国家高级别生物安全实验室体系。

二是管理运维、技术发展、标准制定、评价认证以及应用指导能力显著提高。形成完善的国家实验室生物安全管理的法律法规体系、标准和评价认证体系；实验室技术水平持续提高，一大批实验室的技术指标居国际领先地位；实验室运行和使用效率整体进入世界前列；建立产学研用相结合的实验室生物安全设备技术创新体系，形成一批具有自主知识产权的设备产品；建立实验室生物安全培训体系，形成结构优化、布局合理、素质优良的人才队伍。探索建立国家生物安全实验室创新中心，为实验室的生物安全管理、人员培训提供支撑服务，成为国家生物安全实验室网络的资源和信息共享平台。

三是国际科技合作水平显著改善。在相关国际组织中发挥更加重要的作用，广泛参与国际实验室生物安全法律法规和标准规范的制订和修订，发起与参与国际科技合作计划，一批实验室成为相关国际组织的参考和参比实验室，在海外建设一批联合实验室。

四、重点任务

（一）实验室体系建设

1、四级实验室

加快现有四级实验室建设，在东北地区，依托中国农业科学院建设的高级别生物安全设施，建成以重要动物传染病与人兽共患病为特色的综合性研究平台；在西南地区，依托中国医学科学院和中国科学院共建的高级别生物安全设施，建成以灵长类动物实验为特色的综合性研究平台；在华中地区，依托中国科学院建设的高级别生物安全设施，建成高致病性病原微生物的综合研究中心和世界卫生组织参考实验室，成为重要的国际合作交流平台；在华北地区，依托中国疾病预防控制中心，推动建设高级别生物安全实验室，形成以传染病预防控制和研究为特色的综合性研究平台。在完善在建四级实验室功能，保障其安全高效运行的基础上，根据国家需求和实验室建设进度，在华南、华东和西北地区择机启动四级实验室的审批和建设。

2、三级实验室

三级实验室的建设实行分类建设、强化监管的原则，根据医药人口健康、动物卫生、检验检疫和生态环境安全等四大领域需求，建设具备病原体检测分析、疫苗检验、菌（毒）种保藏、病理解剖、科学研究和生产服务等功能的公益性三级实验室，同时鼓励企业根据自身需求建设或联合建设非公益性三级实验室。对于已经建成三级实验室的省份，应保障其高效运行，充分发挥其功能；对于正在建设三级实验室的省份，应抓紧建设；对部分尚未建设三级实验室的省份，可暂时通过购买其他省份实验室服务来满足需求，同时应抓紧建设本省的三级实验室。

3、其他生物安全防护设施

按照需求导向的原则，在具备条件的部门、地方和行业，建设用于科学研究和特殊领域（从事深海、太空、极地等特殊环境和未知生命研究等的相关工作）的生物安全实验室或者防护设施。

（二）完善实验室保障条件

1、健全实验室管理体系，加强对实验室生物安全防护的质量控制和全过程监管，完善实验室生物安全、菌（毒）种保藏、储存运输相关规范和操作流程，落实安保设置措施，制定实验室生物安全事故应对和处置预案。

2、突破实验室关键技术和设备研发的技术瓶颈；加强现有设备的验证与产业化开发和新设备研制，形成自主可控并具有世界先进水平的实验室关键技术和设备的开发与制造能力。

3、强化实验室生物安全理论和实践培训，建立高级别生物安全模拟实验室，建立高级别实验室生物安全专业技术人员培训和记录制度。

4、建设生物安全实验室创新中心，制定科学、合理、统一的技术标准和规范，对高级别生物安全实验室的有关资源和信息进行整理、汇交和建库，实现资源的信息化、网络化，为体系的资源和信息共享提供支撑。

五、保障机制

（一）健全规章制度

抓紧制定《高级别生物安全实验室活动规范》、《病原微生物实验室生物安保管理办法》等配套文件，积极推动《病原微生物实验室生物安全管理条例》、《人间传染的病原微生物名录》等相关规定及部门管理文件的修订，加快人员防护等领域有关法规的研究论证工作。完善高级别生物安全实验室评价标准体系和认证制度体系。

（二）完善协调管理机制

将高级别生物安全实验室建设规划纳入国家生物安全的相关战略和政策，完善高级别生物安全实验室体系建设的协调机制。国务院有关部门、各地方人民政府等按照国务院规定依法负责实验室及其实验活动的生物安全管理工作。相关行业主管部门结合本部门实际情况，在评估已建在建实验室布局的基础上，制定高级别生物安全实验室建设方案。

（三）保障经费多元投入

国家投资主管部门和科技主管部门根据行业部门的方案，统筹考虑实验室整体布局，继续安排国家资金用于高级别生物安全实验室建设。继续加强对高级别生物安全实验室科研活动的投入。研究推动在国家科技计划“基地和人才专项”中，增设高级别生物安全实验室科目。公益性实验室的建设单位在主管部门和属地政府协调下，将实验室运行经费纳入一般性财政预算中，非公益性实验室的运行经费由建设单位自行负责，确保实验室安全运行。加强高级别实验室生物安全关键技术、关键零部件、配套系统和装备研发的支持力度。

（四）强化人才队伍培养

围绕《国家中长期人才发展规划纲要》，推进实验室设计、建设、管理、科研和战略研究人才队伍建设。结合国家和地方人才计划工程，吸引和凝聚一大批高层次人才。完善满足高级别生物安全实验室特点的人才分类考核、评价和激励机制，为实验室建设和运行提供专业人员保障。加强相关人员的生物安全防护工作，维护人员健康权益，调动工作积极性，保护人员健康安全。

（五）加强资源信息共享

制定促进高级别生物安全实验室分类开放共享的管理制度和办法，并建立相应评价体系和激励引导机制，将开放共享程度作为四级和公益类三级实验室运行考核的指标，根据评价结果配置资源，鼓励非公益类的三级实验室参与资源共享。建立健全高级别生物安全实验室体系内部的信息搜集与上报机制，在符合保密管理规定的前提下建立信息发布机制，做好高级别生物安全实验室发展形势的信息搜集、监测、分析工作，定期向国家安全委员会和国务院提交发展状况年度报告或白皮书引导实验室建设健康有序发展。

六、规划的修订与调整

严格执行规划修改的法定程序和条件，规定当出现下列几种情况之一时，规划的制定机构可按照规定的权限和程序修改规划：国家更高层面规划发生变更，本规划需要进行调整以适应新要求的；产生新的重大需求并经规划的审批机构会同国家病原微生物实验室生物安全专家委员会评估确需修改的；规划的审批机构认为应当修改规划的其他情形；出现其他情况，由国家发展改革委会同科技部依照法定权限和程序进行修改。军队高级别生物安全实验室体系建设发展规划由中国人民解放军卫生主管部门参照本规划负责具体制定。

1. 国务院应对新型冠状病毒肺炎疫情联防联控机制综合组：关于印发《全员新型冠状病毒核酸检测组织实施指南》的通知

联防联控机制综发〔2021〕27号

医疗废物的处理措施。医疗废物的处理是控制采集场所和检测实验室生物安全的关键环节，必须充分掌握生物安全废弃物的分类，并严格执行相应的处理程序。

1. 国家卫生健康委员会：国家卫生计生委办公厅做好高致病性病原微生物科研项目生物安全监督工作的通知

国卫办科教函〔2016〕785号

为做好行政审批取消后与人体健康有关高致病性病原微生物科研项目的实验室生物安全监督工作，现将有关要求通知如下：

一、在高等级生物安全实验室开展与人体健康有关的高致病性病原微生物科研项目，应当符合现行法规、规章对于从事高致病性病原微生物实验活动的管理要求。

二、在四级生物安全实验室开展高致病性病原微生物科研项目，实验室应当将立项结果告知国家卫生计生委；在三级生物安全实验室开展高致病性病原微生物科研项目，实验室应当将立项结果告知省级人民政府卫生计生行政部门。各级卫生计生行政部门应当根据国家法律法规和属地化管理原则，认真做好事中和事后监督。

三、各相关部门和单位应当在职责范围内落实好高致病性病原微生物科研项目的生物安全管理职责，按照谁立项谁负责、谁主管谁负责的原则，进一步研究完善相关的监管措施。

四、各相关部门和单位要重点加强对人间传染的高致病性病原微生物和人畜共患疾病有关科研项目的全过程管理，特别是要加强科研伦理和实验室生物安全审核，督促有关科研人员严格遵守相关法律法规要求，不得开展对人群和生态环境造成潜在巨大风险的实验活动。

1. 农业农村部：农业部关于进一步加强国家兽医参考实验室管理的通知

农医发〔2016〕13号

各省级兽医主管部门要充分认识加强国家参考实验室管理的重要性，积极参与有关工作，依法加强实验室生物安全监管。

1. 国家卫生健康委员会：国家卫生健康委办公厅关于做好妇幼保健机构秋冬季新冠肺炎疫情防控工作的通知

国卫办妇幼函〔2020〕879号

要按照规定做好实验室备案，对实验室工作人员加强技术培训和指导，严格落实《医疗机构新型冠状病毒核酸检测工作手册（试行）》要求，提高技术操作的规范性。规范开展实验室室内质控和室间质评，做好核酸检测质控工作，加强实验室生物安全管理，做好剩余样本处理，保证检测质量和生物安全。

1. 国务院应对新型冠状病毒肺炎疫情联防联控机制综合组：关于鼓励疾控机构开展新冠病毒核酸检测服务的通知

联防联控机制综发〔2020〕230号

要加强实验室生物安全管理，严格操作流程，妥善处置剩余生物样本和实验室废弃物。

1. 国家卫生健康委员会：国家卫生计生委关于印发《传染病防治卫生监督工作规范》的通知

国卫监督发〔2014〕44号

第六节 病原微生物实验室生物安全管理的卫生监督

第二十五条病原微生物实验室生物安全管理的卫生监督内容：

（一）一、二级病原微生物实验室的备案情况；三、四级病原微生物实验室开展高致病性病原微生物实验活动的资格；

（二）从事实验活动的人员培训、考核及上岗持证情况；

（三）管理制度、应急预案的制定和落实情况；

（四）开展实验活动情况；

（五）实验档案建立和保存情况；

（六）菌（毒）种和样本的采集、运输和储存情况。

第二十六条监督检查病原微生物实验室菌（毒）种和样本采集、运输及实验活动等管理情况时，主要采取以下方法：

（一）查阅一级、二级实验室的备案证明和三级、四级实验室《高致病性病原微生物实验室资格证书》；

（二）查阅实验室工作人员的培训、考核资料和上岗证；

（三）核查实验室将病原微生物菌（毒）种和样本就地销毁或者送交保藏机构保管的记录；

（四）检查二级及以上实验室相应设备配置情况；

（五）查阅实验档案；核查高致病性病原微生物相关实验活动实验档案的保存年限；

（六）查阅从事某种高致病性病原微生物或者疑似高致病性病原微生物实验活动的批准文件；查阅实验室经论证可使用新技术、新方法从事高致病性病原微生物相关实验活动的证明文件；查阅从事在我国尚未发现或者已经宣布消灭的病原微生物相关实验活动的资质证明文件，以及相关实验活动的记录；

（七）查阅高致病性病原微生物实验室安全保卫制度；检查三、四级实验室在明显位置标示的生物危险标识和生物安全实验室级别标志，以及进入实验室人员的防护用品配备情况；

（八）查阅高致病性病原微生物或者疑似高致病性病原微生物相关实验活动的登记及结果报告记录；检查是否在同一个实验室的同一个独立安全区域内同时从事两种或者两种以上高致病性病原微生物的相关实验活动；

（九）查阅高致病性病原微生物实验室感染应急处置预案及向所在地省级卫生计生行政部门备案的资料；

（十）查阅实验室工作人员出现高致病性病原微生物感染、实验室发生高致病性病原微生物泄漏的报告、处置记录；

（十一）查阅高致病性病原微生物样本来源、采集过程和方法的记录；

（十二）查阅运输高致病性病原微生物菌（毒）种或样本的批准文件；查阅高致病性病原微生物菌(毒)种和样本运输过程中发生被盗、被抢、丢失、泄漏后的报告记录。

第二十七条监督检查保藏机构菌（毒）种和样本储存管理时，主要采取以下方法：

（一）查阅保藏机构的资格证书；

（二）查阅安全保管制度、病原微生物菌（毒）种和样本进出与储存的记录，接受实验室提交的病原微生物菌（毒）种和样本的登记和开具接收证明情况；

（三）查阅向实验室提供高致病性病原微生物菌（毒）种和样本的登记，核查实验室提交的从事高致病性病原微生物相关实验活动的批准文件；检查高致病性病原微生物菌（毒）种和样本设专库或者专柜单独储存的情况；

（四）查阅高致病性病原微生物菌(毒)种和样本储存过程中发生被盗、被抢、丢失、泄漏后的报告记录。

1. 国家中医药管理局：国家中医药管理局办公室关于做好新冠肺炎疫情常态化防控中医药各项工作的通知

国中医药办医政函〔2020〕116号

各中医医疗机构要按照《关于进一步做好疫情期间新冠病毒检测有关工作的通知》(联防联控机制综发〔2020〕152号)和《新型冠状病毒肺炎实验室检测技术指南》(国卫办疾控函〔2020〕156号)要求，加大相关医务人员培训力度、规范技术操作，加强实验室检测质量控制，提高检测质量，加强实验室生物安全管理、做好剩余样本处理。

1. 国家食品药品监督管理总局：关于开展食品药品检验检测机构实验室生物安全情况调查工作的通知

食药监科便函〔2014〕60号

主要包括从事病原微生物菌（毒）种、样本有关实验活动的食品药品检验检测机构的生物安全组织管理基本信息，以及机构内各生物安全实验室的病原微生物使用情况、认证认可及备案情况、人员情况、建筑情况、生物安全仪器设备配备情况等详细信息。

1. 国家卫生健康委：国家卫生健康委办公厅关于加强新型冠状病毒感染的肺炎重症病例医疗救治工作的通知

国卫办医函〔2020〕64号

医疗机构要加强临床实验室生物安全管理，尽最大努力避免医院感染发生。

1. 农业农村部：对十三届全国人大二次会议第7088号建议的答复

在防控工作中，我部高度重视实验室检测及相关技术研发工作，规范实验室生物安全管理，为防控决策提供了有力支持。

1. 农业农村部：农业部办公厅关于加强“猪流感”A/H1N1病毒实验活动监管工作的通知

农明字[2009]第63号

现就加强“猪流感”A/H1N1病毒实验监管工作提出如下要求。

一、严格“猪流感”A/H1N1病毒实验活动审批

根据监测和流行病学调查结果，我国以往未在猪和人体中检测到引起此次疫情的“猪流感”A/H1N1病毒。按照《病原微生物实验室生物安全管理条例》第二十八条的规定，对我国尚未发现或者已经宣布消灭的病原微生物，任何单位和个人未经批准不得从事相关实验活动。为了预防、控制人感染猪流感疫情，需要从事“猪流感”A/H1N1病毒相关实验活动的，应当经农业部批准，并在指定的专业实验室中进行。为此，各级各类实验室和教学科研单位从动物体上检测到“猪流感”A/H1N1病毒阳性的，必须按照规定向农业部兽医局报告，并采集病料分别送国家禽流感参考室和国家外来动物疫病诊断中心进行病毒分离鉴定和抗原分析。除国家禽流感参考实验室和国家外来动物疫病诊断中心外，任何实验室不得从事“猪流感”A/H1N1病毒的分离鉴定和抗原分析实验。同时，未经农业部批准，任何单位和个人不得从国外引进“猪流感”A/H1N1病毒。

二、做好“猪流感”A/H1N1病毒样本运送管理

（一）严格执行样本运送审批制度。各级动物防疫机构为检测、诊断染“猪流感”A/H1N1疫情，需要运送“猪流感”A/H1N1病毒样本的，应当按照《病原微生物实验室生物安全管理条例》第十一条规定的分工，报省级以上兽医主管部门批准；出入境检验检疫机构在检验检疫过程中需要运输“猪流感”A/H1N1病毒样本的，应当报国家质检总局批准，并同时向农业部通报。

（二）加强样本运送生物安全管理。“猪流感”A/H1N1病毒样本运送过程中，要按照《高致病性动物病原微生物菌（毒）种或者样本运输包装规范》的规定，切实做好运送过程中病原微生物生物安全管理，严防病毒样本被盗、被抢、丢失或者泄漏。通过民用航空运送病毒样本的，还要符合《中国民用航空危险品运输管理规定》（CCAR-276部）和国际民航组织《危险物品安全航空运输技术细则》（ICAO Doc 9284 AN/905）的要求，并遵守《关于运输动物菌毒种样本 病料等有关事宜的通知》（局发明电[2008]4487号）的规定。

三、加强“猪流感”A/H1N1病毒实验活动监督管理

各级兽医主管部门一定要全面贯彻落实《病原微生物实验室生物安全管理条例》的各项规定，采取切实有效措施，加强“猪流感”A/H1N1病毒实验活动监管，确保广大人民群众身体健康和生命安全。

（一）切实落实实验室生物安全责任制。落实实验室设立单位是生物安全第一责任人制度，明确实验室生物安全管理责任，按照属地管理原则，签署和完善兽医实验室生物安全责任书，做到一级抓一级，层层抓落实。加强实验室内部管理，健全生物安全管理制度，确保各项制度落到实处。

（二）严肃查处违法从事实验活动的行为。各级兽医主管部门要严格执行“猪流感”A/H1N1病毒实验活动事前审批制度，对未经批准从事“猪流感”A/H1N1病毒实验活动的，要依法严肃查处；构成犯罪的，要依法追究刑事责任。

（三）严格执行实验活动报告制度。经批准从事“猪流感”A/H1N1病毒实验的国家禽流感参考实验室和国家外来动物疫病诊断中心，应当及时向农业部报告实验活动情况；未及时报告的，将依法追究单位负责人责任。

1. 农业农村部：农业部办公厅关于印发《农业部2009年兽医工作要点》的通知

农办医〔2009〕3号

规范高致病性动物病原微生物实验室资格审查和实验活动审批，严格动物病原微生物菌（毒）种管理，强化兽医实验室生物安全培训，组织开展对兽医实验室生物安全状况抽查，加强兽医实验室生物安全监督检查。出台实施兽医实验室考核管理办法，明确兽医系统实验室建设标准和工作要求，组织有关单位开展分级考核，提高实验室建设水平和检测诊断能力。

1. 中国民用航空局：中国民用航空局关于运输动物菌毒种、样本、病料等有关事宜的通知

局发明电〔2008〕4487号

为做好重大动物疫病的检测诊断和菌（毒）种保藏工作，各地兽医部门需要通过航空运输方式将动物病原微生物菌（毒）种或者样本以及动物病料（以下简称“菌毒种和样本及动物病料”）送至有关实验室检测。为做好菌毒种和样本及动物病料的运输工作，确保航空运输安全，按照《动物防疫法》和《病原微生物实验室生物安全管理条例 》的规定，经研究，制定以下运输方案：

一、菌毒种和样本及动物病料必须作为货物进行航空运输，禁止随身携带或作为托运行李或邮件进行运输。菌毒种和样本及动物病料的航空运输需符合《中国民用航空危险品运输管理规定

》（CCAR-276 部，以下简称“CCAR-276 部”）和国际民航组织《危险品安全航空运输技术细则》（ICAO Doc 9284 AN/905，以下简称《技术细则》）的要求。

二、菌毒种和样本及动物病料的托运人或其代理人必须接受符合CCAR-276 部和《技术细则》要求的危险品航空运输训练，并持有有效证书。目前，农业部及各省兽医部门已派员完成危险品航空运输训练，具体人员名单及联系电话见附件3。

三、菌毒种和样本及动物病料的托运手续必须符合国务院《病原微生物实验室生物安全管理条例 》、（国务院第424 号令）农业部《高致病性动物病原微生物实验室生物安全管理审批办法

》（农业部第52 号令）以及《动物病原微生物分类名录》（农业部第53 号令）的规定。跨省、自治区、直辖市或向境外运输动物病原微生物菌（毒）种或者样本时，托运人需持有农业部颁发的《动物病原微生物菌（毒）种或样本及动物病料准运证书》（样本见附件1）。运输动物病料或在省、自治区、直辖市人民政府行政区域内运输动物病原微生物菌（毒）种或者样本时，托运人需持有出发地省、自治区、直辖市人民政府兽医行政管理部门（名单见附件4）颁发的《动物病原微生物菌（毒）种或样本及动物病料准运证书》。对于出入境菌毒种和样本及动物病料的运输，需由出入境检验检疫机构进行检疫。

四、菌毒种和样本及动物病料必须由已获得局方颁发的《危险品航空运输许可》的航空公司进行运输。对于运输航空公司尚未获得危险品运输许可的航点，运输航空公司可向地区管理局申请《危险品航空运输临时许可》，通过特殊安排或派有资质的人员赴始发站办理收运等方法，在托运方满足上述第二、第三条的基础上进行航空运输，其间产生的费用由货物托运方承担。

五、菌毒种和样本及动物病料的包装需符合国际民航组织《技术细则》以及农业部《高致病性病原微生物菌（毒）种或者样本运输包装规范》（农业部公告第503号）的要求，同时必须符合国家质量监督检验检疫部门的要求或附有进口包装材料符合国际标准的有关证明文件。

六、民航各单位应制定航空运输感染性物质的应急处置程序。菌毒种和样本及动物病料如在运输过程中出现紧急情况，应及时与运输申请单位及机场所在地的省、自治区、直辖市人民政府兽医行政管理部门联系，机场应急部门、航空公司危险品运输管理部门和民航各地区管理局（含各监管办）危险品空运主管部门应积极提供相关协助。

1. 农业农村部：农业部办公厅关于做好2018年春节期间兽医领域有关工作的通知

农办医〔2018〕3号

扎实做好兽医实验室生物安全管理。加强兽医实验室生物安全监管，督促指导各有关实验室所属法人单位严格落实生物安全管理规定，规范高致病性动物病原微生物实验活动，切实落实动物病原微生物菌（毒）种保藏场所安保措施，严防病原微生物失窃、泄露等事件发生。在春节前，组织开展对辖区内兽医实验室生物安全管理情况集中检查，及时发现和消除隐患。

1. 国家卫生健康委：卫生部关于印发《医院管理评价指南(2008版)》的通知

卫医发〔2008〕27号

（1）贯彻落实《病原微生物实验室生物安全管理条例》、《医疗机构临床实验室管理办法》等有关规定。临床实验室集中设置，统一管理，资源共享。实验室管理统一标准，统一质控，保证质量。

（2）临床实验室布局与流程安全、合理，符合医院感染控制和生物安全要求。

（3）开展检验项目符合卫生行政部门公布的目录，不开展淘汰和未经批准的项目。特殊实验室取得审批许可。

1. 国家卫生计生委办公厅关于做好人感染H7N9禽流感医疗救治工作的通知

国卫发明电〔2017〕3号

加强临床实验室生物安全管理，严格实验室质量控制。

1. 农业农村部：农业部关于切实加强农业安全生产工作的通知

农人发〔2008〕9号

兽医部门要加强实验室监督管理，健全生物安全管理制度，狠抓实验室生物安全责任落实。要完善兽医实验室管理制度，规范动物病原微生物菌（毒）种保藏、病料采集运输、病原分离、实验室安全操作等工作。要继续做好《病原微生物实验室生物安全管理条例》的学习宣传工作，进一步提高相关人员的生物安全意识。

1. 国家卫生健康委员会：国家卫生计生委关于印发2017年卫生计生工作要点的通知

国卫办函﹝2017﹞11号

强化实验室生物安全监管。

1. 农业农村部：农业部办公厅关于做好兽医领域安全风险防范工作的通知

农办医〔2016〕61号

进一步抓好兽医实验室生物安全管理工作

要进一步强化兽医实验室生物安全监管，督促指导各有关实验室所属法人单位严格落实生物安全管理规定，规范高致病性动物病原微生物实验活动,切实落实动物病原微生物菌（毒）种保藏场所安保措施，严防病原微生物失窃、泄露等事件发生。要及时组织对本地区、本单位的兽医实验室生物安全情况及相关安全生产工作进行一次全面检查，及时排除安全隐患。

1. 农业农村部：农业部关于实施发展现代农业重点行动的意见

农发〔2007〕2号

严格病原微生物实验室生物安全监管

1. 农业农村部：农业部办公厅关于印发《2016年兽医工作要点》的通知

农办医〔2016〕7号

（十二）强化兽医实验室监督管理。严格动物病原微生物高级别生物安全实验室资格和实验活动审批，规范动物病原微生物菌（毒）种保存使用监管。组织做好兽医系统实验室考核、检测能力比对工作。开展辖区内兽医实验室基本情况及生物安全管理情况摸底调查。严厉打击违反实验室生物安全管理法律法规的行为。

1. 国家卫生计生委关于印发《2016年卫生计生工作要点》的通知

国卫办发〔2016〕6号

强化实验室生物安全监管。

1. 中国合格评定国家认可委员会：CNAS－CL05实验室生物安全认可准则

第一部分：实验室生物安全通用要求

1

范围

本标准规定了实验室生物安全管理和实验室的建设原则，同时，还规定了生物安全分级、实验室设施设备的配置、个人防护和实验室安全行为的要求。本标准为最低要求， 此类实验室还应同时符合国家其他相关规定的要求。

2

术语和定义下列术语和定义适用于本标准：

2.1 生物因子biological agents

一切微生物和生物活性物质。

2.2 病原体pathogens

可使人、动物或植物致病的生物因子。

2.3 危险废弃物hazardous waste

有潜在生物危险、可燃、易燃、腐蚀、有毒、放射和起破坏作用的对人、环境有害的一切废弃物。

2.4 危害risk

伤害发生的概率及其严重性的综合。

2.5 气溶胶aerosols

悬浮于气体介质中的粒径一般为 0.001-100μm 的固态或液态微小粒子形成的相对稳定的分散体系。

2.6 生物安全biosafety

避免危险生物因子造成实验室人员暴露、向实验室外扩散并导致危害的综合措施。

2.7 高效空气过滤器high efficiency particulate air filter （HEPA）

通常以滤除≥0.3μm 微粒为目的，滤除效率符合相关要求的过滤器。

2.8 安全罩safety hood

置于实验室工作台或仪器设备上的负压排风罩，以减少实验室工作者的暴露危险。

2.9 生物安全柜biological safety cabinet（BSC）

负压过滤排风柜。防止操作者和环境暴露于实验过程中产生的生物气溶胶。

2.10 个人防护装备personal protective equipment（PPE）

用于防止人员受到化学和生物等有害因子伤害的器材和用品。

2.11 实验室分区laboratory area

按照生物因子污染概率的大小，实验室可进行合理的分区。

2.12 缓冲间 buffer room

设置在清洁区、半污染区和污染区相临两区之间的缓冲密闭室，具有通风系统，其两个门具有互锁功能，且不能同时处于开启状态。

2.13 气锁 air lock

气压可调节的气密室，用于连接气压不同的两个相邻区域，其两个门具有互锁功能，不能同时处于开启状态。在实验室中用作特殊通道。

2.14 定向气流directional airflow

在气压低于外环境大气压的实验室中，从污染概率小且相对压力高处向污染概率高且相对压力低处受控制流动的气流。

2.15 材料安全数据单material safety data sheet（MSDS）

提供详细的危险和注意事项信息的技术通报。

3

危害程度分级

根据生物因子对个体和群体的危害程度将其分为4 级。

3.1危害等级I （低个体危害，低群体危害）

3.2危害等级Ⅱ（中等个体危害，有限群体危害）

不会导致健康工作者和动物致病的细菌、真菌、病毒和寄生虫等生物因子。

能引起人或动物发病，但一般情况下对健康工作者、群体、家畜或环境不会引起严重危害的病原体。实验室感染不导致严重疾病，具备有效治疗和预防措施，并且传播风险有限。

3.3 危害等级 Ⅲ （高个体危害，低群体危害）

能引起人类或动物严重疾病，或造成严重经济损失，但通常不能因偶然接触而在个体间传播，或能使用抗生素、抗寄生虫药治疗的病原体。

3.4 危害等级Ⅳ（高个体危害，高群体危害）

能引起人类或动物非常严重的疾病，一般不能治愈，容易直接或间接或因偶然接触在人与人，或动物与人，或人与动物，或动物与动物间传播的病原体。

4

生物危害评估

当实验室活动涉及传染或潜在传染性生物因子时，应进行危害程度评估。危害程度评估应至少包括下列内容：生物因子的种类（已知的、未知的、基因修饰的或未知传染性的生物材料）、来源、传染性、致病性、传播途径、在环境中的稳定性、感染剂量、浓度、动物实验数据、预防和治疗。

危害程度评估应由适当的有经验的专业人员进行。

5

防护屏障和生物安全水平分级

5.1 防护屏障

5.1.1一级防护屏障

5.1.2二级防护屏障

实验室的生物安全柜和个人防护装备等构成的防护屏障。

实验室的设施结构和通风系统等构成的防护屏障。

5.2 生物安全水平分级

根据所操作的生物因子的危害程度和采取的防护措施，将生物安全防护水平（biosafety level，BSL）分为4 级，I 级防护水平最低，IV 级防护水平最高。以BSL-1、BSL-2、BSL-3、BSL-4 表示实验室的相应生物安全防护水平；以ABSL-1、ABSL-2、ABSL-3、ABSL-4 表示动物实验室的相应生物安全防护水平。

6

设施和设备要求

实验室所用设施、设备和材料（含防护屏障）均应符合国家相关的标准和要求。

6.1 BSL－1 实验室

1） 无需特殊选址，普通建筑物即可，但应有防止节肢动物和啮齿动物进入的设计。

2） 每个实验室应设洗手池，宜设置在靠近出口处。

3） 在实验室门口处应设挂衣装置，个人便装与实验室工作服分开放置。

4） 实验室的墙壁、天花板和地面应平整、易清洁、不渗水、耐化学品和消毒剂的腐蚀。地面应防滑，不得铺设地毯。

5） 实验台面应防水，耐腐蚀、耐热。

6） 实验室中的橱柜和实验台应牢固。橱柜、实验台彼此之间应保持一定距离，以便于清洁。

7） 实验室如有可开启的窗户，应设置纱窗。

8） 实验室内应保证工作照明，避免不必要的反光和强光。

9） 应有适当的消毒设备。

6.2 BSL-2 实验室

1） 满足6.1 的要求。

2） 实验室门应带锁并可自动关闭。实验室的门应有可视窗。

3） 应有足够的存储空间摆放物品以方便使用。在实验室工作区域外还应当有供长期使用的存储空间。

4） 在实验室内应使用专门的工作服；应戴乳胶手套。

5） 在实验室的工作区域外应有存放个人衣物的条件。

6） 在实验室所在的建筑内应配备高压蒸汽灭菌器，并按期检查和验证，以保证符合要求。

7）应在实验室内配备生物安全柜。

8） 应设洗眼设施，必要时应有应急喷淋装置。

9） 应通风，如使用窗户自然通风，应有防虫纱窗。

10） 有可靠的电力供应和应急照明。必要时，重要设备如培养箱、生物安全柜、冰箱等应设备用电源。

11） 实验室出口应有在黑暗中可明确辨认的标识。

6.3 BSL－3 实验室

应在建筑物中自成隔离区（有出入控制）或为独立建筑物。

6.3.1 布局

1） 由清洁区、半污染区和污染区组成。污染区和半污染区之间应设缓冲间。必要时，半污染区和清洁区之间应设缓冲间。

2） 在半污染区应设供紧急撤离使用的安全门。

3） 污染区与半污染区之间、半污染区和清洁区之间应设置传递窗，传递窗双门不能同时处于开启状态，传递窗内应设物理消毒装置。

6.3.2 围护结构

1） 实验室围护结构内表面应光滑、耐腐蚀、防水，以易于消毒清洁；所有缝隙应可靠密封，防震、防火。

2） 围护结构外围墙体应有适当的抗震和防火能力。

3） 天花板、地板、墙间的交角均为圆弧形且可靠密封。

4） 地面应防渗漏、无接缝、光洁、防滑。

5） 实验室内所有的门应可自动关闭；实验室出口应有在黑暗中可明确辨认的标识。

6） 外围结构不应有窗户；内设窗户应防破碎、防漏气及安全。

7） 所有出入口处应采用防止节肢动物和啮齿动物进入的设计。

6.3.3 送排风系统

1） 应安装独立的送排风系统以控制实验室气流方向和压力梯度。应确保在使用实验室时气流由清洁区流向污染区，同时确保实验室空气只能通过高效过滤后经专用排风管道排出。

2） 送风口和排风口的布置应该是对面分布，上送下排，应使污染区和半污染区内的气流死角和涡流降至最小程度。

3） 送排风系统应为直排式，不得采用回风系统。

4） 由生物安全柜排出的经内部高效过滤的空气可通过系统的排风管直接排出。应确保生物安全柜与排风系统的压力平衡。

5） 实验室的送风应经初、中、高三级过滤，保证污染区的静态洁净度达到7 级到8 级。

6） 实验室的排风应经高效过滤后向空中排放。外部排风口应远离送风口并设置在主导风的下风向，应至少高出所在建筑2m，应有防雨、防鼠、防虫设计，但不应影响气体直接向上空排放。

7） 高效空气过滤器应安装在送风管道的末端和排风管道的前端。

8） 通风系统、高效空气过滤器的安装应牢固，符合气密性要求。高效过滤器在更换前应消毒，或采用可在气密袋中进行更换的过滤器，更换后应立即进行消毒或焚烧。每台高效过滤器安装、更换、维护后都应按照经确认的方法进行检测，运行后每年至少进行一次检测以确保其性能。

9） 在送风和排风总管处应安装气密型密闭阀，必要时可完全关闭以进行室内化学熏蒸消毒。

10） 应安装风机和生物安全柜启动自动联锁装置，确保实验室内不出现正压和确保生物安全柜内气流不倒流。排风机一备一用。

11）在污染区和半污染区内不应另外安装分体空调、暖气和电风扇等。

6.3.4 环境参数

1） 相对室外大气压，污染区为-40Pa （名义值），并与生物安全柜等装置内气压保持安全合理压差。保持定向气流并保持各区之间气压差均匀。

2） 实验室内的温度、湿度符合工作要求且适合于人员工作。

3） 实验室的人工照明应符合工作要求。

4） 实验室内噪声水平应符合国家相关标准。

6.3.5 特殊设备装置

1） 应有符合安全和工作要求的II 级或Ⅲ 级生物安全柜，其安装位置应离开污染区入口和频繁走动区域。

2） 低温高速离心机或其他可能产生气溶胶的设备应置于负压罩或其他排风装置（通风橱、排气罩等）之中，应将其可能产生的气溶胶经高效过滤后排出。

3） 污染区内应设置不排蒸汽的高压蒸汽灭菌器或其他消毒装置。

4） 应在实验室入口处的显著位置设置带报警功能的室内压力显示装置，显示污染区、半污染区的负压状况。当负压值偏离控制区间时应通过声、光等手段向实验室内外的人员发出警报。还应设置高效过滤器气流阻力的显示。

5） 应有备用电源以确保实验室工作期间有不间断的电力供应。

6） 应在污染区和半污染区出口处设洗手装置。洗手装置的供水应为非手动开关。供水管应安装防回流装置。不得在实验室内安设地漏。下水道应与建筑物的下水管线完全隔离，且有明显标识。下水应直接通往独立的液体消毒系统集中收集，经有效消毒后处置。

6.3.6 其他

1）实验台表面应防水，耐腐蚀、耐热。

2）实验室中的家具应牢固。为便于清洁，实验室设备彼此之间应保持一定距离。

3）实验室所需压力设备（如泵，压缩气体等）不应影响室内负压的有效梯度。

4）实验室应设置通讯系统。

5）实验记录等资料应通过传真机、计算机等手段发送至实验室外。

6）清洁区设置淋浴装置。必要时，在半污染区，设置紧急消毒淋浴装置。

6.4 BSL－4 实验室

BSL－4 实验室根据使用的生物安全柜的类型和穿着防护服的不同，可以分为安全柜型、正压服型和混合型实验室。

6.4.1安全柜型BSL－4 实验室

6.4.1.1 选址实验室应建造在独立的建筑物内或建筑物中独立的完全隔离区域内，该建筑物应远离城区。

6.4.1.2 布局

1） 由清洁区、半污染区和安放有Ⅲ 级生物安全柜的污染区组成。清洁区包括外更衣室、淋浴室和内更衣室。相邻区由缓冲间连接。

2） 应在半污染区和清洁区墙上、半污染区和污染区墙上设置不排蒸汽的双扉高压灭菌器和浸泡消毒渡槽或熏蒸消毒室或带有消毒装置的通风互锁传递窗，以便传递或消毒不能从更衣室携带进出的材料、物品和器材。

3）污染区和半污染区墙上设置不排蒸汽的双扉高压灭菌器应与Ⅲ 级生物安全柜直接相连。

4） 半污染区应设紧急出口，紧急出口通道应设置缓冲间和紧急消毒处理室。

6.4.1.3围护结构按6.3.2 的规定。

6.4.1.4送排风系统排风应连续经过两个高效过滤器处理。其他要求按6.3.3 的规定。

6.4.1.5环境参数按6.3.4 的规定。

6.4.1.6安全装置及特殊设备1） 应有符合安全和工作要求的Ⅲ 级生物安全柜。其他要求按6.3.5 的规定。

6.4.1.7其他按6.3.6 的规定。

6.4.2正压服型BSL－4 实验室

由BSL－4 级实验设施、II 级生物安全柜和具有生命支持供气系统的正压防护服组成。

6.4.2.1选址按6.4.1.1 的规定。

6.4.2.2布局

1） 由清洁区、半污染区和安放有II 级生物安全柜的污染区组成，相邻区由缓冲间连接。清洁区包括外更衣室、淋浴室、内更衣室（可兼缓冲间）， 污染区、半污染区之间的缓冲间应设化学淋浴装置，工作人员离开实验室时，经化学淋浴对正压防护服表面进行消毒。

2） 其他要求按6.4.1.2 的2）和4）的规定。

6.4.2.3围护结构按6.3.2 的规定。

6.4.2.4送排风系统按6.4.1.4 的规定。

6.4.2.5环境参数按6.3.4 的规定。

6.4.2.6安全装置及特殊设备

1） 应使用II 级外排风型生物安全柜。

2） 进入污染区的工作人员应穿着正压防护服。生命支持系统包括提供超量清洁呼吸气体的正压供气装置，报警器和紧急支援气罐。工作服内气压相对周围环境应为持续正压，并符合要求。生命支持系统应有自动启动的紧急电源供应。

3） 其他要求按6.3.5 的规定。

6.4.2.7其他按6.3.6 的规定。

6.4.3混合型BSL-4 实验室

在本级实验设施基础上，同时使用Ⅲ 级生物安全柜和具有生命支持供气系统（正压防护服）。应同时符合本标准6.4.1 和6.4.2 的全部要求。

7

动物实验室的生物安全

动物实验室的生物安全防护设施应参照BSL1-4 实验室的要求（见6），还应考虑对动物呼吸、排泄、毛发、抓咬、挣扎、逃逸、动物实验（如染毒、医学检查、取样、解剖、检验等）、动物饲养、动物尸体及排泄物的处置等过程产生的潜在生物危害的防护。应特别注意对动物源性气溶胶的防护，例如对感染动物的剖检应在负压剖检台上进行。

应根据动物的种类、身体大小、生活习性、实验目的等选择具适当防护水平的、专用于动物的、符合国家相关标准的生物安全柜、动物饲养设施、动物实验设施、消毒设施和清洗设施等。

实验室建筑应确保实验动物不能逃逸，非实验室动物（如野鼠、昆虫等）不能进入。实验室设计（如空间、进出通道等）应符合所用动物的需要。动物实验室空气不应循环。动物源气溶胶应经适当的高效过滤/消毒后排出，不能进入室内循环。如动物需要饮用无菌水，供水系统应可安全消毒。动物实验室内的温度、湿度、照度、噪声、洁净度等饲养环境应符合国家相关标准的要求。

7.1 ABSL－1 实验室

除满足6.1 的要求外，还应满足以下要求：

1） 建筑物内动物设施应与开放的人员活动区分开。

2） 应安装自动闭门器，当有实验动物时应保持锁闭状态。

3） 如果有地漏，应始终用水或消毒剂液封。

4） 动物笼具的洗涤应满足清洁要求。

7.2 ABSL-2 实验室

除满足6.2 和7.1 的要求外，还应满足以下要求：

1） 出入口应设缓冲间。

2） 动物实验室的门应当具有可视窗，可以自动关闭，并有适当的火灾报警器。

3） 为保证动物实验室运转和控制污染的要求，用于处理固体废弃物的高压灭菌器应经过特殊设计，合理摆放，加强保养；焚烧炉应经过特殊设计，同时配备补燃和消烟设备；污染的废水必须经过消毒处理。

7.3 ABSL－3 实验室

除满足6.3 和7.2 的要求外，还应满足以下要求：

1） 建筑物应有符合要求的抗震能力，防鼠、防虫、防盗。

2） 由清洁区、半污染区和污染区（动物饲养间）组成。污染区和半污染区之间应设缓冲间。必要时，半污染区和清洁区之间应设缓冲间。

3） 相对室外大气压，污染区为-60Pa （名义值），并与生物安全柜等装置内气压保持安全合理压差。保持定向气流并保持各区之间气压差均匀。

4） 室内应配备人工或自动消毒器具（如消毒喷雾器、臭氧消毒器）并备有足够的消毒剂。5） 当房间内有感染动物时，应戴防护面具。

7.4 ABSL－4 实验室

应满足6.4 和7.3 的要求，并符合以下要求：

1）应增加动物进入的通道。

2） 感染动物应饲养在具有Ⅲ 级生物安全柜性能的隔离器内。

3） 动物饲养方法要保证动物气溶胶经高效过滤后排放，不能进入室内。

4） 一般情况，操作感染动物，包括接种、取血、解剖、更换垫料、传递等，都要在物理防护条件下进行。能在生物安全柜内进行的必须在其内进行；特殊情况下，不能在生物安全柜内饲养的大动物或动物数量较多时，要根据情况特殊设计，例如设置较大的生物安全柜和可操作的物理防护设备，尽可能在其内进行高浓度污染的操作。

8

个人防护装备

实验室所用任何个人防护装备应符合国家有关标准的要求。在危害评估的基础上，按不同级别的防护要求选择适当的个人防护装备。实验室对个人防护装备的选择、使用、维护应有明确的书面规定、程序和使用指导。

8.1 实验室防护服

实验室应确保具备足够的有适当防护水平的清洁防护服可供使用。不用时，只应将清洁的防护服置于专用存放处。污染的防护服应于适当标记的防漏袋中放置并搬运。

每隔适当的时间应更换防护服以确保清洁，当知道防护服已被危险材料污染应立即更换。离开实验室区域之前应脱去防护服。

当具潜在危险的物质极有可能溅到工作人员时，应使用塑料围裙或防液体的长罩服。在这种工作环境中，如必要，还应穿戴其它的个人防护装备，如手套、防护镜、面具、头部面部保护罩等。

8.2 面部及身体保护

处理样本的过程中，如可产生含生物因子的气溶胶，应在适当的生物安全柜中操作。

在处理危险材料时应有许可使用的安全眼镜、面部防护罩或其他的眼部面部保护装置可供使用。

8.3 手套

手套应在实验室工作时可供使用，以防生物危险、化学品、辐射污染，冷和热，产品污染，刺伤、擦伤和动物抓咬伤等。

手套应按所从事操作的性质符合舒服、合适、灵活、握牢、耐磨、耐扎和耐撕的要求，并应对所涉及的危险提供足够的防护。应对实验室工作人员进行选择手套，使用前及使用后的配戴及摘除等培训。

应保证：

1）所戴手套无漏损；

2）戴好手套后可完全遮住手及腕部，如必要，可覆盖实验室长罩服或外衣的袖子；

3）在撕破、损坏或怀疑内部受污染时更换手套；

4）手套为实验室工作专用。在工作完成或中止后应消毒、摘掉并安全处置。

8.4 鞋

鞋应舒适，鞋底防滑。推荐使用皮制或合成材料的不渗液体的鞋类。在从事可能出现漏出的工作时可穿一次性防水鞋套。在实验室的特殊区域（例如有防静电要求的区域）或BSL-3 和BSL-4 实验室要求使用专用鞋（例如一次性或橡胶靴子）。

8.5 呼吸防护

当要求使用呼吸防护装备（如面具、个人呼吸器、正压服等）时，其使用和维护的作业指导书应包括在相应活动的安全操作程序手册中。呼吸器应只能按照作业指导书及培训的要求使用。

应安排工作场所监控、医学评估和对呼吸器使用者的监督，以确保其始终正确使用该类装备。应对呼吸器作个体适合性测试。

进行容易产生高危害气溶胶的操作时，要求同时使用适当的个人防护装备、生物安全柜和/或其它物理防护设备。

9

管理要求

9.1 管理责任

实验室管理层对所有员工和实验室来访者的安全负责。最终责任由实验室负责人或指定的与其地位相当者承担。应任命一名有适当资质和经验的实验室安全负责人协助管理层负责安全事宜。安全负责人应制定、维护和监督有效的实验室安全计划。一个有效的实验室安全计划应包括教育、定位及培训、审核及评估、促进实验室安全行为的程序。

实验室安全负责人应有权阻止不安全的活动。如设有安全委员会，实验室安全负责人如果不是该委员会的主任，至少应是该委员会有职权的成员。

实验室负责人应制定规定和程序确保实验室设施、设备、个人防护设备、材料等符合国家有关安全要求，定期检查、维护、更新，确保不降低其设计性能。

9.2 员工健康管理

所有人员应有文件证明其对工作及实验室全部设施中潜在的风险受过培训。

应要求所有人员根据可能接触的生物接受免疫以预防感染。应保存免疫记录。

9.3 安全设计

在考虑建筑新的实验室或计划对已建好的实验室进行结构改造时，应遵守相应的国家、地方建筑法规和对实验室的专用建筑安全标准。未得到实验室负责人或其指定代表的许可禁止进行建筑或工程作业。

实验室的设计应保证对技术区域中生物、化学、辐射和物理危害的防护水平控制在经过评估的相应风险程度，为关联的办公区和临近的公共空间提供安全的工作环境，及防止风险进入周围社区。通向出口的走廊和通道应无障碍。

应对空气的流动速度进行常规监测以保证足够的通风和防止潜在传染因子和有害气体的扩散。

实验室的每个出口和入口应可分辨，入口处应有标记，标记应包括国际通用的危险标志（如：生物危险标志、火险标志和放射性标志）以及其它有关的规定的标记。应设紧急出口并有标记以和普通出口区别。紧急撤离路线应有在黑暗中也可明确辨认的标识。

实验室入口应有可锁闭的门。门锁应不妨碍紧急疏散。实验室的进入应仅限于经授权的人员。房间内的门按需要安装门锁；正当操作高危险样本时应有进入限制。存放高危险样本、培养物、化学试剂或供应品，还需采取其他的保安措施，如可锁闭的门、可锁闭的冷冻箱、特殊人员的进入限制等。应评估生物材料、样本、药品、化学品和机密资料被偷盗和被不正当使用的危险，并采取相应措施防范其发生。

应有专门设计以确保存储、转运、收集、处理和处置危险物料的安全。

实验室内温度、湿度、照度、噪声和洁净度等内环境符合工作要求和有关要求。

9.4 程序

应根据实验对象、生物危害程度评估、研究内容、设施特点、设备具体制定相应的标准操作程序。实验室的标准操作程序应包括对涉及的任何危险以及如何在风险最小的情况下开展工作之详细的作业指导书。负责工作区活动的管理责任人每年应对这些程序至少评审和更新一次。应制定书面计划，至少包括以下内容：

1） 员工的健康监护；

2） 实施危害评估，记录结果及采取措施的安排；

3） 化学品和其它危险物品的确认（包括适当的标识要求）、安全存放与处置及监控程序；

4） 操作有害材料的安全行为的程序；

5） 防止高风险和污染材料失窃的程序；

6） 确认培训需求和教材的方法；

7） 获得、维持和分发实验室所有使用材料之安全数据单（MSDS）的程序；

8）实验室设备安全去污染和维护的程序；

9） 紧急程序，包括漏出处理程序；

10） 事件记录、报告及调查；

11） 废弃物处理和处置。

9.5安全计划的审核及检查

9.5.1安全计划的审核

每年应（由受过适当培训的人员）对安全计划至少审核和检查一次，包括但不限于下列要素：

1） 安全和健康规定；

2） 书面的工作程序包括安全工作行为；

3） 教育及培训；

4） 对工作人员的监督；

5） 常规检查；

6） 危险材料和物质；

7） 健康监护；

8） 急救服务及设备；

9） 事故及病情调查；

10） 健康和安全委员会评审；

11） 记录及统计；

12） 确保落实审核中提出需要采取的全部措施的计划。

为每个领域特制的检查表可有效地协助审核工作。

9.5.2 安全检查

实验室管理层有责任确保安全检查的执行。每年应对工作场所至少检查一次，以保证：

1） 应急装备、警报体系和撤离程序功能及状态正常；

2） 用于危险物质漏出控制的程序和物品状态，包括紧急淋浴；

3） 对可燃易燃性、可传染性、放射性和有毒物质的存放进行适当的防护和控制；

4） 去污染和废弃物处理程序的状态；

5） 实验室设施、设备、人员的状态。

9.5.3 安全手册

要求所有员工阅读的安全手册应在工作区随时可用。手册应针对实验室的需要，主要包括但不限于以下几方面：

1） 生物危险；

2） 消防；

3） 电气安全；

4） 化学品安全；

5） 辐射；

6） 危险废弃物处理和处置。

安全手册应对从工作区撤离和事件处理规程有详细说明。实验室管理层应至少每年对安全手册评审和更新。

实验室中其他有用的信息来源还包括（但不限于）实验室涉及的所有材料的安全数据单，教科书和权威性杂志文章等参考资料。

9.6 记录

9.6.1 职业性疾病，伤害和不利事件记录

应有机制记录并报告职业性疾病、伤害、不利事件或事故以及所采取的相应行动，同时应尊重个人机密。

应保持人员培训记录。应包括对每一员工的安全指导和安全预备状态的年度更新资料。

9.6.2 危害评估记录

应有正式的危害评估体系。可利用安全检查表对危害评估过程记录及文件化。安全审核记录和事件趋势分析记录有助于制定和采取补救措施。

9.6.3 危险废弃物记录

危险废弃物处理和处置记录应是安全计划的一个组成部分。危险废弃物处理和处置、危害评估、安全调查记录和所采取的相应行动记录应按有关规定的期限保存并可查阅。

9.6.4 危险标识

应系统而清晰地标识出危险区，且适用于相关的危险。在某些情况下，宜同时使用标记和物质屏障标识出危险区。应清楚地标识在实验室或实验室设备上使用的具体危险材料。通向工作区的所有进出口都应标明存在其中的危险。尤其应注意火险以及易燃、有毒、放射性、有害和生物危险材料。实验室管理层应负责定期评审和更新危险标识系统以确保其适用现有的危险。该活动每年应至少进行一次。应使涉及的非实验室员工（如维护人员、合同方、分包方）知道其可能遇到的任何危险。员工应受培训，熟悉并有关于紧急程序的专用书面指导。应标识和评审对孕妇健康和易感人员的潜在危险。应进行危害评估并记录。

9.6.5 事件、伤害、事故和职业性疾病的报告

实验室应有程序报告实验室事件、伤害、事故、职业性疾病以及潜在危险。所有事件（包括伤害）报告应形成文件，应包括事件的详细描述、原因评估、预防类似事件发生的建议以及为实施建议所采取的措施。事件报告（包括补救措施）应经高层管理者、安全委员会或实验室安全负责人评审。

9.7 培训

实验室负责人应保证对实验室所有相关人员包括运输和清洁员工等工作人员安全培训计划的实施。培训应强调安全工作行为。

一项全面的培训计划始于书面的规划，应包括对新员工的指导以及对有经验员工的周期性再培训。应要求员工在某一领域工作前阅读适用的安全手册。员工应书面确认其已接受适当的培训，阅读并理解了安全手册，包括其执行日期。

一项安全培训计划至少要有消防和预备状态、化学和放射安全、生物危险和传染预防。课程应按照员工的岗位制定，应适当考虑怀孕、免疫缺陷和身体残障情况。应有一套系统评估每个员工对提供给其信息的理解力。

实验室应保证全体人员受过急救培训。应提供物品和程序以减少涉及潜在传染性材料、化学品或有害物质的不利作用和事件的发生。

应有救治指南，必要时，还应有与实验室内可能遇到的危险相适应的紧急医学处理措施。所有员工应熟悉被刺伤后所执行的程序。

9.8 个人责任

9.8.1 食品、饮料及类似物品

食品、饮料及类似物品只应在指定的区域中准备和食用。食品和饮料只应存放于非实验室区域内指定的专用处。冰箱应适当标记以明确其规定用途。实验室内禁止吸烟。

9.8.2 化妆品、发、珠宝

禁止在工作区内使用化妆品和处理隐形眼镜。

长发应束在脑后。在工作区内不应配带戒指、耳环、腕表、手镯、项链和其他珠宝。

9.8.3 免疫状态

所有实验室工作人员应接受免疫以预防其可能被所接触的生物因子感染。应按有关规定保存免疫记录。

对一特定实验室的免疫计划应根据文件化的实验室传染危害评估和地方公共卫生部门的建议制定。

9.8.4 个人物品

个人物品、服装和化妆品不应放在有规定禁放的和可能发生污染的区域。

10

良好内务行为

应指定专人监督保持良好内务的行为。工作区应时刻保持整洁有序。禁止在工作场所存放可能导致阻碍和绊倒危险的大量一次性材料。

所有用于处理污染性材料的设备和工作表面在每班工作结束、有任何漏出或发生了其他污染时应使用适当的试剂清洁和消毒。

对漏出的样本、化学品、放射性核素或培养物应在风险评估后清除并对涉及区域去污染。清除时应使用经核准的安全预防措施、安全方法和个人防护装备。

内务行为改变时应报告实验室负责人以确保避免发生无意识的风险或危险。实验室行为、工作习惯或材料改变可能对内务和/或维护人员有潜在危险时，应报告实验室负责人，并书面告知内务和维护人员的管理者。应制定在发生事故或漏出导致生物、化学或放射性污染时，设备保养或修理之前对每件设备去污染、净化和消毒的专用规程。

11

安全工作行为

11.1 洗手

实验室工作人员在实际或可能接触了血液、体液或其他污染材料后，即使戴有手套也应立即洗手。摘除手套后、使用卫生间前后、离开实验室前、进食或吸烟前、接触每一患者前后应例行洗手。实验室应为过敏或对某些消毒防腐剂中的特殊化合物有其他反应的工作人员提供洗手用的替代品。洗手池不得用于其他目的。在限制使用洗手池的地点，使用基于乙醇的 “无水”手部清洁产品是可接受的替代方式。

11.2 接触生物源性材料的安全工作行为

处理、检验和处置生物源性材料的规定和程序应利用良好微生物行为标准。工作行为应可降低污染的风险。执行污染区内的工作行为应可预防个人暴露。

如果样本在收到时有损坏或泄漏，应由穿着个人防护装备之受过培训的人员开启样本以防止漏出或产生气溶胶。应在生物安全柜内开启此类容器。如果污染过量或认为样本有不可接受的损坏，则应将样本安全地废弃而勿开启。

禁止口吸移液。应培训实验室工作人员安全操作尖利器具及装置。禁止用手对任何利器剪、弯、折断、重新戴套或从注射器上移去针头。安全工作行为应尽可能减少使用利器和尽量使用替代品。

包括针头、玻璃、一次性手术刀在内的利器应在使用后立即放在耐扎容器中。尖利物容器应在内容物达到三分之二前置换。所有样本、培养物和废弃物应被假定含有传染性生物因子，应以安全方式处理和处置。所有有潜在传染性或毒性的质量控制和参考物质在存放、处理和使用时应按未知风险的样本对待。操作样本、血清或培养物的全过程应穿戴适当的且符合风险级别的个人防护装备。操作实验动物应穿戴耐抓咬、防水个人防护服和手套；应戴适当的面部、眼部防护装置，必要时，增加呼吸防护；应在生物安全柜内操作。摘除手套后一定要彻底洗手。应最好采用电子灼烧灭菌装置对微生物接种环灭菌。

11.3 气溶胶

实验室工作行为的设计和执行应能减少人员接触化学或生物源性有害气溶胶。样本只应在有盖安全罩内离心。所有进行涡流搅拌的样本应置于有盖容器内。在能产生气溶胶的大型分析设备上应使用局部通风防护，在操作小型仪器时使用定制的排气罩。在可能出现有害气体和生物源性气溶胶的地方应采取局部排风措施。饲养、操作动物应在适当的动物源性气溶胶防护设备中进行，工作人员应同时使用适当的个人防护设备。有害气溶胶不得直接排放。

11.4 生物安全柜、安全罩

在实验室员工接触危害等级Ⅰ和Ⅱ的场所，生物安全柜内的空气在排放前只要通过高效过滤器可以再循环；在实验室员工接触可能有危害等级Ⅲ或以上的生物因子的场所，禁止将空气再循环。动物实验室禁止将空气再循环。

对于新安装的生物安全柜和安全罩及其高效过滤器的安装与更换，应由有资格的人员进行，安装或更换后应按照经确认的方法进行现场生物和物理的检测，并每年进行验证。实验室应时常监测生物安全柜以确保其设计性能能够符合相关要求。应保存检查记录和任何功能性测试结果。在安全柜上应有作为检查证明的标记。所用生物安全柜的放置、设计和类型应符合安全工作所要求的风险防护级别。所有生物安全柜之使用方式应避免降低其功能。生物安全柜、化学安全罩的通风应符合微生物和/或化学的风险级别及符合安全要求。

12

化学品安全

在实验室中，对化学品的存放、处理、使用及处置的规定和程序均应符合良好化学实验室行为标准。应按照相关标准在每个储存容器上标明每个产品的危害性质和风险性，还应在“使用中”材料的容器上清楚标明。对化学、物理及火灾危害应有足够可行的控制措施。应定期对这些措施进行监督以确保其有效可用。应保存监督结果记录。应要求所有人员按安全操作规程工作，包括使用被认为适用于所从事工作的安全装备或装置。

对实验室内所用的每种化学制品的废弃和安全处置应有明确的书面程序。其应包括对相关法规的充分及详细说明，以保证完全符合其要求，使这些物质安全及合法地脱离实验室控制。

13

放射安全

在批准使用放射性核素之前，实验室负责人应对使用的理由、限度和地点进行评估。实验室应保存充分的放射性核素的获取、使用和处置记录。所有放射性化学品的存放应安全及保险。所有操作或接触放射性核素的实验室人员应接受放射性基础知识、相关技术和放射性防护的指导和培训，应符合放射性安全规定和程序。

实验室应有适当的、满足工作需要的书面标准操作程序和相关的法规。程序应包括清楚的作业指导书，在使用放射性核素的地方应重点显示的作业指导书之摘要，在出现放射性事故和漏出时应采取行动的详细说明。程序应详细说明安全处置不用的放射性材料、与放射性材料相混合的或受其污染的材料之方法。应公示经过批准的警告和禁止标志。从事放射性工作的实验室应向相关主管部门征询有关放射性防护行为和法律要求的建议，包括对实验室设计和设备标准的所有要求，并制定适当的措施确保遵守。

实验室应任命一名放射防护安全员， 其负有设计、执行及维护可操作性放射防护计划的专门责任。实验室应任命若干放射防护监督员监督日常与离子辐射相关的工作，以保证执行良好放射性行为。

应制定系统性监督计划以保证对工作场所进行全面及经常性监督。应保存监督记录。应制定并实施常规清洁和去污染规程。应定期评审放射性核素的使用情况，经常监督工作行为并及时更新。补救措施或程序性变化应记录并按相关要求规定的期限保存。

放射活性废物应有标志并存放在专用于此目的之安全且防辐射的储存库。在每个需弃置的包装上应清楚地说明风险的性质和程度。储存及处置应遵守相关规定。

14

紫外线和激光光源（包括高强度光源的光线）

在使用紫外线和激光光源的场所，应提供适用且充分的个人防护装备，应有适当的标识公示。应为安全使用设备提供培训。这些光源只能用于其设计目的。

15

电气设备

电气设备的设计及制造应符合相关安全标准的要求。为确保安全，某些设备应连接备用电源。新的、改装过的或修理过的电气设备在未经合格的人员（如有资质的电工或生物医学工程师）完成电气安全测试和设备符合安全使用要求之前，不允许使用。

电气设备使用人员应接受正确操作的培训，操作方式应不降低电气安全性。电气设备使用人员应定期检查设备的可能引起电气故障之破损。只有合格的人员许可从事电气设备和电路工作。禁止未经授权的工作。

应采取措施对设备去污染以减少维护人员受化学或生物性污染的风险。

16

防火

建筑防火规格应以实验室所含危险的类型而定。应指定主出口路线。应备有辅助出口确保人员可从实验室安全撤离。指定的消防出口应通向防火区。

应在使用或存放可燃气体或液体的所有实验室区内备有自动烟雾和热量探测及报警系统。应定期检测报警系统以确保其功能正常并使所有人员熟知其运行。

16.1 消防安全培训计划

应对实验室工作人员及建筑物内所有人员进行消防指导和培训。内容包括：

1） 火险的识别及评估；

2） 制定减少火险的计划；

3） 失火时应采取的全部行动。

现场应配备符合相关要求的适当设备用于扑灭可控制的火灾及帮助人员撤离火场。实验室人员的责任是确保人员安全有序地撤离而不是试图去灭火。应寻求消防部门援助。

17

水灾和其他自然灾害

应制定灾害应急预案。如可能，救援人员应事先了解危险物的性质、数量和存放位置，应熟悉实验室的布局和设备。

当遇水灾、地震或其他自然灾害时，视建筑物或实验室遭破坏程度，应采取隔离污染区域和污染源、有效消毒、疏散人员等紧急措施。应对危害进行评估，并采取进一步措施。应有灾害报告制度。

18

紧急撤离

应制定紧急撤离的行动计划。该计划应考虑到生物性、化学性、失火和其他紧急情况。应包括所采取的使留下的建筑物处于尽可能安全状态的措施。所有人员都应了解行动计划、撤离路线和紧急撤离的集合地点。所有人员每年应至少参加一次演习。实验室负责人应确保有用于急救和紧急程序的设备在实验室内可供使用。

19

样本的运送

实验室负责人应负责为所有向实验室提交样本的地点准备适当的指南和指示。所有样本应以防止污染工作人员、患者或环境的方式运送到实验室。样本应置于被承认的、本质安全、防漏的容器中运输。样本在机构所属建筑物内运送应遵守该机构的安全运输规定。样本运送到机构外部应遵守现行的有关运输可传染性和其它生物源性材料的法规。

样本、培养物和其他生物材料在实验室间或其他机构间的运送方式应符合相应的安全规定。应遵守国际和国家关于道路、铁路和水路运输危险材料的有关要求。

按国家或国际标准认为是危险货物的材料拟通过国内或国际空运时，应包装、标记和提供资料，并符合现行国家或国际相关的要求。

20

废弃物处置

实验室废弃物处置的管理应符合国家、地区或地方的相关要求。实验室废弃物管理的目的如下：

1） 将操作、收集、运输、处理及处置废弃物的危险减至最小；

2） 将其对环境的有害作用减至最小。所有不再需要的样本、培养物和其他生物性材料应弃置于专门设计的、专用的和有标记的用于处置危险废弃物的容器内。生物废弃物容器的充满量不能超过其设计容量。

利器（包括针头、小刀、金属和玻璃等）应直接弃置于耐扎容器内。实验室管理层应确保由经过适当培训的人员使用适当的个人防护装备和设备处理危险废弃物。不允许积存垃圾和实验室废弃物。已装满的容器应定期运走。在去污染或最终处置之前，应存放在指定的安全地方，通常在实验室区内。所有弃置的实验室生物样本、培养物和被污染的废弃物在从实验室中取走之前，应使其达到生物学安全。生物学安全可通过高压消毒处理或其他被承认的技术达到。实验室废弃物应置于适当的密封且防漏容器中安全运出实验室。有害气体、气溶胶、污水、废液应经适当的无害化处理后排放，应符合国家相关的要求。动物尸体和组织的处置和焚化应符合国家相关的要求。

第二部分：病原微生物实验室生物安全管理条例的相关要求

本部分实验室生物安全认可要求直接引用国务院《病原微生物实验室生物安全管理条例》的相关条款，条款号与《病原微生物实验室生物安全管理条例》的相同。

第九条 采集病原微生物样本应当具备下列条件：

（一）具有与采集病原微生物样本所需要的生物安全防护水平相适应的设备；

（二）具有掌握相关专业知识和操作技能的工作人员；

（三）具有有效的防止病原微生物扩散和感染的措施；

（四）具有保证病原微生物样本质量的技术方法和手段。

采集高致病性病原微生物样本的工作人员在采集过程中应当防止病原微生物扩散和感染，并对样本的来源、采集过程和方法等作详细记录。第十条运输高致病性病原微生物菌（毒）种或者样本，应当通过陆路运输；没有陆路通道，必须经水路运输的，可以通过水路运输；紧急情况下或者需要将高致病性病原微生物菌（毒）种或者样本运往国外的，可以通过民用航空运输。第十一条运输高致病性病原微生物菌（毒）种或者样本，应当具备下列条件：

（一）运输目的、高致病性病原微生物的用途和接收单位符合国务院卫生主管部门或者兽医主管部门的规定；

（二）高致病性病原微生物菌（毒）种或者样本的容器应当密封，容器或者包装材料还应当符合防水、防破损、防外泄、耐高（低）温、耐高压的要求；

（三）容器或者包装材料上应当印有国务院卫生主管部门或者兽医主管部门规定的生物危险标识、警告用语和提示用语。

运输高致病性病原微生物菌（毒）种或者样本，应当经省级以上人民政府卫生主管部门或者兽医主管部门批准。在省、自治区、直辖市行政区域内运输的，由省、自治区、直辖市人民政府卫生主管部门或者兽医主管部门批准；需要跨省、自治区、直辖市运输或者运往国外的，由出发地的省、自治区、直辖市人民政府卫生主管部门或者兽医主管部门进行初审后，分别报国务院卫生主管部门或者兽医主管部门批准。

出入境检验检疫机构在检验检疫过程中需要运输病原微生物样本的，由国务院出入境检验检疫部门批准，并同时向国务院卫生主管部门或者兽医主管部门通报。

通过民用航空运输高致病性病原微生物菌（毒）种或者样本的，除依照本条第二款、第三款规定取得批准外，还应当经国务院民用航空主管部门批准。第十二条运输高致病性病原微生物菌（毒）种或者样本，应当由不少于2 人的专人护送，并采取相应的防护措施。

有关单位或者个人不得通过公共电（汽）车和城市铁路运输病原微生物菌（毒）种或者样本。第十三条需要通过铁路、公路、民用航空等公共交通工具运输高致病性病原微生物菌（毒）种或者样本的，承运单位应当凭本条例第十一条规定的批准文件予以运输。

承运单位应当与护送人共同采取措施，确保所运输的高致病性病原微生物菌（毒）种或者样本的安全，严防发生被盗、被抢、丢失、泄漏事件。第十六条实验室在相关实验活动结束后，应当依照国务院卫生主管部门或者兽医主管部门的规定，及时将病原微生物菌（毒）种和样本就地销毁或者送交保藏机构保管。第十七条高致病性病原微生物菌（毒）种或者样本在运输、储存中被盗、被抢、丢失、泄漏的，承运单位、护送人、保藏机构应当采取必要的控制措施，并在2 小时内分别向承运单位的主管部门、护送人所在单位和保藏机构的主管部门报告，同时向所在地的县级人民政府卫生主管部门或者兽医主管部门报告，发生被盗、被抢、丢失的，还应当向公安机关报告。

任何单位和个人发现高致病性病原微生物菌（毒）种或者样本的容器或者包装材料，应当及时向附近的卫生主管部门或者兽医主管部门报告。第十九条新建、改建、扩建三级、四级实验室或者生产、进口移动式三级、四级实验室应当遵守下列规定：

（一）符合国家生物安全实验室体系规划并依法履行有关审批手续；

（二）经国务院科技主管部门审查同意；

（三）符合国家生物安全实验室建筑技术规范；

（四）依照《中华人民共和国环境影响评价法》的规定进行环境影响评价并经环境保护主管部门审查批准；

（五）生物安全防护级别与其拟从事的实验活动相适应。第二十条三级、四级实验室应当通过实验室国家认可。第二十一条一级、二级实验室不得从事高致病性病原微生物实验活动。三级、四级实验室从事高致病性病原微生物实验活动，应当具备下列条件：

（一）实验目的和拟从事的实验活动符合国务院卫生主管部门或者兽医主管部门的规定；

（二）通过实验室国家认可；

（三）具有与拟从事的实验活动相适应的工作人员；

（四）工程质量经建筑主管部门依法检测验收合格。第二十二条取得从事高致病性病原微生物实验活动资格证书的实验室，需要从事某种高致病性病原微生物或者疑似高致病性病原微生物实验活动的，应当依照国务院卫生主管部门或者兽医主管部门的规定报省级以上人民政府卫生主管部门或者兽医主管部门批准。实验活动结果以及工作情况应当向原批准部门报告。

实验室申报或者接受与高致病性病原微生物有关的科研项目，应当符合科研需要和生物安全要求，具有相应的生物安全防护水平，并经国务院卫生主管部门或者兽医主管部门同意。第二十三条出入境检验检疫机构、医疗卫生机构、动物防疫机构在实验室开展检测、诊断工作时，发现高致病性病原微生物或者疑似高致病性病原微生物，需要进一步从事这类高致病性病原微生物相关实验活动的，应当依照本条例的规定经批准同意，并在取得相应资格证书的实验室中进行。

专门从事检测、诊断的实验室应当严格依照国务院卫生主管部门或者兽医主管部门的规定，建立健全规章制度，保证实验室生物安全。第二十五条新建、改建或者扩建一级、二级实验室，应当向设区的市级人民政府卫生主管部门或者兽医主管部门备案。第二十七条已经建成并通过实验室国家认可的三级、四级实验室应当向所在地的县级人民政府环境保护主管部门备案。第二十八条对我国尚未发现或者已经宣布消灭的病原微生物，任何单位和个人未经批准不得从事相关实验活动。

为了预防、控制传染病，需要从事前款所指病原微生物相关实验活动的，应当经国务院卫生主管部门或者兽医主管部门批准，并在批准部门指定的专业实验室中进行。第二十九条实验室使用新技术、新方法从事高致病性病原微生物相关实验活动的，应当符合防止高致病性病原微生物扩散、保证生物安全和操作者人身安全的要求，并经国家病原微生物实验室生物安全专家委员会论证；经论证可行的，方可使用。第三十条需要在动物体上从事高致病性病原微生物相关实验活动的，应当在符合动物实验室生物安全国家标准的三级以上实验室进行。第三十一条实验室的设立单位负责实验室的生物安全管理。实验室的设立单位应当依照本条例的规定制定科学、严格的管理制度，并定期对有关生物安全规定的落实情况进行检查，定期对实验室设施、设备、材料等进行检查、维护和更新，以确保其符合国家标准。实验室的设立单位及其主管部门应当加强对实验室日常活动的管理。第三十二条实验室负责人为实验室生物安全的第一责任人。实验室从事实验活动应当严格遵守有关国家标准和实验室技术规范、操作规程。实验室负责人应当指定专人监督检查实验室技术规范和操作规程的落实情况。第三十三条从事高致病性病原微生物相关实验活动的实验室的设立单位，应当建立健全安全保卫制度，采取安全保卫措施，严防高致病性病原微生物被盗、被抢、丢失、泄漏，保障实验室及其病原微生物的安全。实验室发生高致病性病原微生物被盗、被抢、丢失、泄漏的，实验室的设立单位应当依照本条例第十七条的规定进行报告。

从事高致病性病原微生物相关实验活动的实验室应当向当地公安机关备案，并接受公安机关有关实验室安全保卫工作的监督指导。第三十四条实验室或者实验室的设立单位应当每年定期对工作人员进行培训，保证其掌握实验室技术规范、操作规程、生物安全防护知识和实际操作技能，并进行考核。工作人员经考核合格的，方可上岗。

从事高致病性病原微生物相关实验活动的实验室，应当每半年将培训、考核其工作人员的情况和实验室运行情况向省、自治区、直辖市人民政府卫生主管部门或者兽医主管部门报告。第三十五条从事高致病性病原微生物相关实验活动应当有2 名以上的工作人员共同进行。

进入从事高致病性病原微生物相关实验活动的实验室的工作人员或者其他有关人员，应当经实验室负责人批准。实验室应当为其提供符合防护要求的防护用品并采取其他职业防护措施。从事高致病性病原微生物相关实验活动的实验室，还应当对实验室工作人员进行健康监测，每年组织对其进行体检，并建立健康档案；必要时，应当对实验室工作人员进行预防接种。第三十六条在同一个实验室的同一个独立安全区域内，只能同时从事一种高致病性病原微生物的相关实验活动。第三十七条实验室应当建立实验档案，记录实验室使用情况和安全监督情况。实验室从事高致病性病原微生物相关实验活动的实验档案保存期，不得少于20 年。第三十八条实验室应当依照环境保护的有关法律、行政法规和国务院有关部门的规定，对废水、废气以及其他废物进行处置，并制定相应的环境保护措施，防止环境污染。第三十九条三级、四级实验室应当在明显位置标示国务院卫生主管部门和兽医主管部门规定的生物危险标识和生物安全实验室级别标志。 第四十条 从事高致病性病原微生物相关实验活动的实验室应当制定实验室感染应急处置预案，并向该实验室所在地的省、自治区、直辖市人民政府卫生主管部门或者兽医主管部门备案。第四十二条实验室的设立单位应当指定专门的机构或者人员承担实验室感染控制工作，定期检查实验室的生物安全防护、病原微生物菌（毒）种和样本保存与使用、安全操作、实验室排放的废水和废气以及其他废物处置等规章制度的实施情况。

负责实验室感染控制工作的机构或者人员应当具有与该实验室中的病原微生物有关的传染病防治知识，并定期调查、了解实验室工作人员的健康状况。第四十三条实验室工作人员出现与本实验室从事的高致病性病原微生物相关实验活动有关的感染临床症状或者体征时，实验室负责人应当向负责实验室感染控制工作的机构或者人员报告，同时派专人陪同及时就诊；实验室工作人员应当将近期所接触的病原微生物的种类和危险程度如实告知诊治医疗机构。第四十四条实验室发生高致病性病原微生物泄漏时，实验室工作人员应当立即采取控制措施，防止高致病性病原微生物扩散，并同时向负责实验室感染控制工作的机构或者人员报告。第四十五条负责实验室感染控制工作的机构或者人员接到本条例第四十三条、第四十四条规定的报告后，应当立即启动实验室感染应急处置预案，并组织人员对该实验室生物安全状况等情况进行调查；确认发生实验室感染或者高致病性病原微生物泄漏的，应当依照本条例第十七条的规定进行报告，并同时采取控制措施，对有关人员进行医学观察或者隔离治疗，封闭实验室，防止扩散。

1. 农业农村部：农业部关于印发《全国小反刍兽疫消灭计划（2016—2020年）》的通知

农医发〔2015〕34号

各地要加强兽医体系能力建设，特别是各级兽医实验室能力建设。中国动物卫生与流行病学中心（国家外来动物疫病研究中心）和农业部指定实验室应做好病毒跟踪监测与风险评估工作，加强新型疫苗研究和快速诊断技术研究，做好技术储备，及时提出措施建议，做好技术支持。加强实验室生物安全管理，未经农业部批准，任何单位和个人不得从事小反刍兽疫病原分离、鉴定、纯化和保存工作。

1. 国家卫生健康委员会：卫生部办公厅关于做好基层卫生机构实验室生物安全能力建设工作的通知

卫办科教发〔2006〕17号

根据国务院《病原微生物实验室生物安全管理条例》和卫生部《人间传染的病原微生物名录》的有关要求，生物安全实验室的建设和人员培训是开展病原微生物实验活动的基本条件。为保障防病治病工作的正常开展，各医疗卫生机构应当配置相应的生物安全实验室，其中二级生物安全实验室是基本配置。各级卫生主管部门应当加大对于生物安全实验室建设和人员培训工作的投入，争取用几年的时间使本地区的医疗卫生机构病原微生物实验室达到生物安全实验室的标准。

为加强农村和中西部地区基层疾病预防控制机构病原微生物检测实验室生物安全能力建设，提高微生物检测实验室工作人员生物安全意识和能力，中央财政在2005年度补助地方公共卫生专项资金中安排了加强中西部地区基层卫生机构实验室生物安全能力建设项目。总投资额为3000万元。主要内容是为中西部地区和辽宁省368个县级疾病预防控制中心、新疆生产建设兵团7个师级疾病预防控制中心各1个二级生物安全实验室提供基本装备，同时在全国范围内开展生物安全知识培训。项目具体内容见《财政部、卫生部关于补助公共卫生专项资金的通知》(财社[2005] 169号)。为做好这项工作，提出以下要求：

一、各项目省卫生主管部门要高度重视这项工作，加强项目的组织领导与管理，积极组织协调并与其他有关部门密切配合，根据中央财政补助地方卫生专项资金管理的要求，结合当地的实际情况，认真抓好项目实施的具体工作，确保项目实施的进度和质量。

要合理配置生物安全实验室资源，避免重复设置。应根据当地防病工作的需求，优先选择没有二级生物安全实验室的县进行配置。

二、严格资金的使用管理，提高资金的使用效益。各项目单位要严格按照《财政部、卫生部关于印发〈中央补助地方卫生事业专项资金管理暂行办法〉的通知》(财社[2004]24号)的要求，切实加强专项资金管理。合理安排使用资金，保证重点工作，专款专用，不得挪用。严格执行政府集中招标采购制度，提高设备采购透明度，选用质量可靠，价格合理，售后服务好的有信誉的企业的产品。

三、要认真做好人员培训工作。各项目省卫生行政部门要制订好培训工作计划，加强对人员培训工作的监督管理。培训结束后应通过考试或考核的方式对培训效果进行评估。

四、加强监督检查，建立绩效评价机制，制定项目考核指标和评估标准。各项目省卫生行政部门负责项目实施和实施中的监督管理和效果评价工作。加强对项目方案制定、执行情况和有关财务制度执行情况的监督检查工作。各项目省卫生行政部门要按照要求对于二级生物安全实验室进行验收。对不符合要求的要令其整改。

五、中西部地区要在认真实施本项目的基础上，加大投入力度。东部地区也要多方筹集资金，加强基层卫生机构实验室生物安全能力建设。请各项目省将本项目二级实验室配置及培训计划和本地区生物安全实验室建设规划分别于2006年2月28日和3月31日前报我部科教司。卫生部将加强对各地区实验室生物安全建设进程的督导检查。

1. 国家卫生健康委员会：国家卫生计生委关于印发《2015年卫生计生工作要点》的通知

国卫办发〔2015〕3号

加强实验室生物安全监管。

1. 国家卫生健康委员会：卫生部关于印发《卫生部农业部关于人畜共患传染病防治合作机制》的通知

卫疾控发〔2005〕383号

双方共同遵守《病原微生物实验室生物安全条例》，对病料保存和毒株进行严格管理，防止泄漏和扩散。

1. 农业部、科学技术部、教育部：农业部、科学技术部、教育部等关于加强高致病性病原微生物研究管理工作的紧急通知

农医发〔2005〕14号

加强青海湖高致病性禽流感防控，规范高致病性禽流感疫情监测、疫病诊断和病毒分离等科研工作。针对目前一些科研、教学单位未经批准，在不具备相应实验室生物安全和自我防护条件下，到疫区采集病料、分离病毒等情况，依据《动物防疫法 》和《病原微生物实验室生物安全管理条例》等有关法律法规规定，现将有关问题紧急通知如下：

一、提高认识、高度重视高致病性病原微生物管理

高致病性病原微生物管理关系到动物疫病的防控、实验室工作人员和公众健康。国家高度重视病原微生物的生物安全管理。2004年11月12日，国务院颁布的《病原微生物实验室生物安全管理条例》，对高致病性病原微生物的管理作出了明确规定。各地、各部门要从防控重大动物疫病、保护人民群众身体健康和维护社会安全与稳定高度，充分认识做好高致病性原微生物管理的重要性，加强领导，明确责任，防止未经批准擅自从事高致病性病原微生物的病料采集、诊断以及病原分离、鉴定等活动。

二、加强对高致病性病原微生物病料采集管理

发生高致病性禽流感等重大动物疫情或疑似重大动物疫情，需进行实验室检测和确诊的，必须由动物防疫监督机构或在动物防疫监督机构指导下，采集和运输病料、血清等样品。任何单位和个人未经省级以上兽医行政主管部门批准，不得到疫区解剖病死畜禽和野生动物，采集、运输病料；已经采集的病料须做无害化处理；分离到的病原微生物须送国家兽医参考实验室或中国兽医药品监察所（国家兽医微生物保藏中心）保存。未经农业部批准，任何单位和个人采集的病料、病原微生物不得带出境外。

三、加强实验室生物安全管理

根据《病原微生物实验室生物安全管理条例》，凡从事高致病性病原微生物研究、教学、诊断、检测等活动的单位，必须经过农业部、卫生部批准，符合实验室生物安全管理有关规定。国家相关参考实验室也要严格执行有关规定，严格病料保存和毒株管理等措施，防止病毒泄漏和扩散。

四、加强疫病诊断、监测及疫情信息发布管理

根据《动物防疫法 》规定，由农业部统一发布动物疫情信息，任何单位、个人不得擅自对外发布重大动物疫病诊断、监测结果和疫情信息。根据《传染病防治法》的规定，未经卫生部批准，任何单位、个人不得发布相关的人间疫情信息。对违反国家规定，造成病原微生物扩散或擅自发布疫情信息，造成重大经济损失和社会影响的，要依法追究有关当事人和所在单位负责人的责任。

1. 农业农村部：农业部公告第503号—高致病性动物病原微生物菌种或者样本运输包装规范

农业部公告第503号

运输高致病性动物病原微生物菌（毒）种或者样本的，其包装应当符合以下要求：

一、内包装

（一）必须是不透水、防泄漏的主容器，保证完全密封；

（二）必须是结实、不透水和防泄漏的辅助包装；

（三）必须在主容器和辅助包装之间填充吸附材料。吸附材料必须充足，能够吸收所有的内装物。多个主容器装入一个辅助包装时，必须将它们分别包装。

（四）主容器的表面贴上标签，表明菌（毒）种或样本类别、编号、名称、数量等信息。

（五）相关文件，例如菌（毒）种或样本数量表格、危险性声明、信件、菌（毒）种或样本鉴定资料、发送者和接收者的信息等应当放入一个防水的袋中，并贴在辅助包装的外面。

二、外包装

（一）外包装的强度应当充分满足对于其容器、重量及预期使用方式的要求；

（二）外包装应当印上生物危险标识并标注“高致病性动物病原微生物，非专业人员严禁拆开！”的警告语。

注：生物危险标识如下图：（略）

三、包装要求

（一）冻干样本

主容器必须是火焰封口的玻璃安瓿或者是用金属封口的胶塞玻璃瓶。

（二）液体或者固体样本

1.在环境温度或者较高温度下运输的样本：只能用玻璃、金属或者塑料容器作为主容器，向容器中罐装液体时须保留足够的剩余空间，同时采用可靠的防漏封口，如热封、带缘的塞子或者金属卷边封口。如果使用旋盖，必须用胶带加固。

2.在制冷或者冷冻条件下运输的样本：冰、干冰或者其他冷冻剂必须放在辅助包装周围，或者按照规定放在由一个或者多个完整包装件组成的合成包装件中。内部要有支撑物，当冰或者干冰消耗掉以后，仍可以把辅助包装固定在原位置上。如果使用冰，包装必须不透水；如果使用干冰，外包装必须能排出二氧化碳气体；如果使用冷冻剂，主容器和辅助包装必须保持良好的性能，在冷冻剂消耗完以后，应仍能承受运输中的温度和压力。

四、民用航空运输特殊要求

通过民用航空运输的，应当符合《中国民用航空危险品运输管理规定》（CCAR276）和国际民航组织文件Doc9284《危险物品航空安全运输技术细则》中的有关包装要求。

1. 国家卫生健康委员会：卫生部关于印发《医院管理评价指南(试行)》的通知

卫医发〔2005〕104号

（1）贯彻落实《病原微生物实验室生物安全管理条例》等有关规定。

（2）临床检验实验室集中设置，统一管理，资源共享。实验室管理统一标准，统一质控，保证质量。

（3）临床检验实验室布局与流程应当安全、合理，并符合医院感染控制和生物安全要求。

1. 农业农村部：农业部办公厅关于开展2014年兽医系统实验室检测能力比对工作的通知

农办医〔2014〕13号

1. 加强实验室生物安全管理。各省兽医部门要合理设置地（市）、县级兽医实验室比对项目和试验方法。各实验室要严格按照国家有关标准、技术规范、操作规程以及本单位制定的实验活动安全作业指导书，做好实验材料运输、使用、保管，确保实验室生物安全。
2. 国家卫生健康委员会：卫生部关于印发《2005年卫生工作要点》的通知

全面贯彻实施《病原微生物实验室生物安全管理条例》，抓紧制定相关配套文件，不断完善实验室生物安全体系和监督管理体系。开展生物安全监督检查和培训工作。

1. 国家卫生健康委员会：关于进一步做好人感染H7N9禽流感医疗救治工作的通知

落实医院感染防控措施。医疗机构要严格执行人感染H7N9禽流感医院感染预防与控制的工作要求，完善管理制度，落实岗位责任，严格执行消毒隔离，科学实施个人防护措施。要严格执行传染病预检分诊管理的工作要求，医疗机构要设立相对独立的发热门诊和隔离观察室，设置明显标识引导。发热门诊要保证充足的候诊、就诊空间，改善通风条件，及时分流患者。要加强临床实验室生物安全管理，严格实验室质量控制。

1. 国家卫生健康委员会：关于加强人感染H7N9禽流感疫情防控工作的通知

三、加强监测和实验室检测等防控工作

各级各类医疗卫生机构要严格按照有关规定，做好疫情监测、排查和报告工作。各地要加强不明原因肺炎病例监测，对不明原因肺炎病例要增加H7N9禽流感病毒感染的排查，尤其是已报告确诊病例的地区。一旦发现病例，要及时报告，并加强密切接触者追踪管理、疫情溯源、流行病学调查和实验室检测等工作。

各地要加强疫情形势跟踪和研判，指定专人每日审核和分析医疗机构疫情报告情况。要强化专业人员培训和实验室生物安全监管，提高疫情防控能力，并加强对医疗卫生机构传染病疫情报告、预检分诊和消毒隔离制度落实情况、医疗废物处置情况及疾病预防控制机构菌（毒）种管理制度执行情况的监督检查。同时，做好相关预案、工作方案的准备，以及检测试剂、耗材、消杀器械和防护用品的保障。

1. 农业农村部：农业部办公厅关于开展2013年兽医系统实验室检测能力比对工作的通知

农办医〔2013〕14号

1. 切实做好实验室生物安全管理工作。各省级兽医主管部门要按照实验室生物安全管理要求，合理设置地（市）、县级兽医实验室比对项目和试验方法。各实验室要严格按照国家有关标准、技术规范、操作规程以及本单位制定的实验活动安全作业指导书，做好实验材料运输、使用、保管，确保实验室生物安全。
2. 国家卫生健康委员会：卫生部关于印发《医疗机构临床实验室管理办法》的通知

卫医发〔2006〕73号

第四章 医疗机构临床实验室安全管理

第三十三条医疗机构应当加强临床实验室生物安全管理。

医疗机构临床实验室生物安全管理要严格执行《病原微生物实验室生物安全管理条例》等有关规定。

第三十四条医疗机构临床实验室应当建立并严格遵守生物安全管理制度与安全操作规程。

第三十五条医疗机构应当对临床实验室工作人员进行上岗前安全教育，并每年进行生物安全防护知识培训。

第三十六条医疗机构临床实验室应当按照有关规定，根据生物危害风险，保证生物安全防护水平达到相应的生物安全防护级别。

第三十七条医疗机构临床实验室的建筑设计应当符合有关标准，并与其生物安全防护级别相适应。

第三十八条医疗机构临床实验室应当按照生物防护级别配备必要的安全设备和个人防护用品，保证实验室工作人员能够正确使用。

第三十九条医疗机构病原微生物样本的采集、运输、储存严格按照《病原微生物实验室生物安全管理条例》等有关规定执行。

第四十条医疗机构临床实验室应当严格管理实验标本及实验所需的菌（毒）种，对于高致病性病原微生物，应当按照《病原微生物实验室生物安全管理条例》规定，送至相应级别的生物安全实验室进行检验。

第四十一条医疗机构临床实验室应当按照卫生部有关规定加强医院感染预防与控制工作。

第四十二条医疗机构临床实验室应当按照《医疗废物管理条例》和《医疗卫生机构医疗废物管理办法》相关规定妥善处理医疗废物。

第四十三条医疗机构临床实验室应当制定生物安全事故和危险品、危险设施等意外事故的预防措施和应急预案。

1. 国家卫生健康委员会：卫生部关于印发2012年卫生工作要点的通知

卫办发〔2012〕8号

重点抓好重大传染病防治工作和国家重大活动中的实验室生物安全监管工作。

1. 农业农村部：农业部办公厅关于印发《农业部2010年兽医工作要点》的通知

农办医〔2010〕5号

加强兽医实验室和动物诊疗机构管理。加强兽医实验室生物安全监管，组织开展兽医实验室生物安全检查，进一步规范高致病性动物病原微生物实验活动，严厉查处违法违规行为。创新兽医实验室管理机制，组织开展兽医实验室考核认证工作，提高兽医系统实验室建设水平和检测诊断能力。组织开展动物诊疗市场调研，提出动物诊疗市场规范化管理意见。加强动物诊疗机构能力建设，研究起草动物诊疗机构分级管理办法，提高动物诊疗水平。

1. 国家卫生健康委员会：卫生部关于印发2010年卫生工作要点的通知

卫办发〔2010〕1号

落实《国家职业病防治规划（2009－2015年）》，继续深入开展以职业卫生、放射卫生、环境卫生、医疗执法、学校卫生和传染病防治监督及实验室生物安全监管为重点的卫生监督工作。

1. 国家中医药管理局：国家中医药管理局关于做好应对甲型H1N1流感大流行中医药防治准备工作的通知

国中医药发〔2009〕25号

要加强实验室生物安全管理，严格实验室质量控制。

1. 国家卫生健康委员会：卫生部办公厅关于做好应对甲型H1N1流感大流行医疗救治准备工作的通知

要加强实验室生物安全管理，严格实验室质量控制。

1. 国家卫生健康委员会：卫生部关于印发2009年卫生工作要点的通知

卫办发〔2009〕15号

创新实验室生物安全监管模式，确保实验室生物安全。

1. 国家卫生健康委员会：卫生部办公厅关于印发《汶川地震灾区疾病预防控制对口支援工作意见》的通知

按照国家有关规定和要求，恢复和建立疾病预防控制中心公共卫生实验室，加强实验室生物安全管理，基本满足恢复重建时期实验室检测工作任务。

1. 农业农村部：农业部关于进一步做好奥运期间农产品有效供应和质量安全工作的通知

农市发〔2008〕14号

加强兽医实验室生物安全管理，加强人畜共患病菌毒种管理。

1. 国家卫生健康委员会：卫生部办公厅关于印发埃博拉出血热等6种传染病预防控制指南和临床诊疗方案的通知

卫办应急发〔2008〕140号

加强实验室生物安全。所有涉及马尔堡病毒活病毒的操作必须在BSL-4级实验室中进行。实验室检验应在生物安全柜内进行，如果没有生物安全三级以上的试验条件，则尽可能减少检验次数，操作时做好个人防护。

1. 国家卫生健康委员会：卫生部关于印发2008年卫生工作要点的通知

卫办发〔2008〕8号

加强实验室生物安全体系建设，认真做好有关细菌病毒毒株的管理。

1. 国家卫生健康委员会：卫生部关于印发《突发急性传染病预防控制战略》的通知

卫应急发〔2007〕203号

制订实验室标本采集、运输、和实验室生物安全规范。建立规范的生物样本库，为突发急性传染病的甄别与比对提供资源。

1. 科学技术部：科学技术部关于印发《社会发展科技工作要点(2006－2010年)》的通知

国科发社字〔2007〕232号

研制一批实验室生物安全防护装备，建立不同级别的实验室生物安全评价标准体系，逐步建立我国实验室生物安全监控和标准体系，保障实验室安全；

1. 农业农村部：农业部关于贯彻落实《中共中央、国务院关于推进社会主义新农村建设的若干意见》的意见

农发〔2006〕1号

强化动物卫生执法监督，开展动物防疫标识溯源信息系统建设试点，加强兽医实验室生物安全监督。

1. 国家卫生健康委员会：卫生部关于印发《2006年卫生工作要点》的通知

卫政法发〔2005〕533号

继续加强实验室生物安全管理，发布并实施有关技术规范，推进卫生行业认证认可工作，加强重点实验室建设与管理。

1. 国家卫生健康委员会：卫生部关于下发《2004－2005年冬春季全国传染性非典型肺炎及流感防治工作方案》的通知

卫发电〔2004〕60号

加强实验室生物安全管理。

1、各级疾病预防控制机构及科研机构要完善有关生物安全规章制度和相关机构的建设，配备必要的人员，健全实验室安全管理制度，使生物安全管理做到经常化、制度化。

2、开展非典、禽流感病毒检测工作的实验室必须符合中华人民共和国卫生行业标准（WS233-2002）《微生物生物医学实验室生物安全通用准则》中三级实验室（P3）的要求，并经过国家有关部门的批准。凡未经批准的单位和个人不得从事非典、禽流感样本的采集、保藏、携带、运输和使用等相关活动。

3、各医疗机构要对医务人员进行有关生物安全知识的培训，提高医务人员生物安全防护意识；要加强对内设检验科和有关实验室生物安全管理，对于医疗机构中病原微生物比较集中的区域和密切接触病原微生物的部门，要配备必要的防护设备。

1. 国家卫生健康委员会：卫生部关于印发传染性非典型肺炎人体样品资源管理规范的通知

一、为加强对传染性非典型肺炎（以下简称SARS）的科学研究工作，合理采集、运输、保藏、规范使用并保护SARS人体样品资源，实现资源共享，规范管理，防止流失，特制定本规范。

二、SARS人体样品资源是指SARS病人和疑似病人及其密切接触者的血液、血清、鼻咽拭子、口咽拭子、痰液、气管吸取液或支气管灌洗液、尿液、粪便，以及SARS死亡患者尸体的组织、器官等样品。

三、本规范适用于各级疾病预防控制、医疗等采集、使用SARS人体样品的机构，以及其他所有可能利用SARS人体样品开展工作的机构。

四、SARS人体样品属于国家特殊生物资源，各级卫生行政主管部门和采集、使用单位负有保护SARS人体样品资源的责任和义务。SARS人体样品用于国家开展与SARS有关的科学研究工作，禁止以任何形式买卖。

第一章 采集

五、采集对象为SARS确诊病人、疑似病人及其密切接触者。采集人员为具有执业资格的医务人员。定点医院的医务人员负责采集住院病人(确诊和疑似病人)的样品，各级疾病预防控制机构的技术人员负责采集非住院对象（出院病人和密切接触者）的样品。未经批准的任何单位和个人不得擅自采集SARS人体样品。

六、SARS人体样品的采集必须严格遵循知情同意的原则。

七、采集要求：

1、第一次采集住院病人的样品须在入院后24小时内进行；采集前应向病人说明采集样品的使用目的；

2、采集单位应指定专人负责样品登记、收集、管理，并认真填写《传染性非典型肺炎病人或密切接触者人体样品采集登记表》（附后）；

3、装有样品的螺口塑料管均用胶布或封口膜密封，用清洁塑料袋包裹严实，外包装按照卫生部《传染性非典型肺炎实验室生物安全操作规范》有关规定执行。

4、每份样品必须标明编号及采集日期；

5、样品采集后应立即送当地疾病预防控制机构，如不能立即送交，可置于冰箱在-20℃以下短暂（24小时之内）保存；尿液样品应暂存于4℃冰箱。

八、样品采集种类和方法：

1、呼吸道样品：包括鼻咽拭子、口咽拭子、痰液及下呼吸道样品。

鼻咽拭子：将棉签平行于上颚插入鼻孔，保持几秒钟，吸收分泌物，拭抹双侧鼻孔。

口咽拭子：适度用力拭抹咽后壁和扁桃体部位，应避免触及舌部。迅速将棉签放入无菌、内装3-5ml样品运送液（其液体配制见第三章第十五条）、带垫圈的螺口塑料管中。在靠近顶端处折断棉签杆，旋紧管盖并密封。

痰液：让病人将痰液咳入无菌、内装4～5ml样品运送液、带垫圈的50ml螺口塑料管中。旋紧管盖并密封。

下呼吸道样品（适用于气管插管病人）：收集气管吸取液或支气管灌洗液5-10ml放入无菌、带垫圈的50ml螺口塑料管中，立即密封。

2、血液样品：包括血清、血凝块。

血清（包括病人急性期和恢复期血清）：采集5-10ml全血于带垫圈的螺口管中，不加抗凝剂。待血液凝固后，分离血清，放置到 -20℃以下冰箱中冷冻保存。采集第一份血清应在入院后24小时内采集；第二份血清应在发病后10-14天采集；第三份血清应在发病后22-28天或出院当日（出院当日未采集的，应在出院后）采集。

3、粪便样品：采集5-10g粪便放入无菌塑料瓶内。在4℃条件下保存并及时送至有关机构。

4、尿液样品：应在患者的急性期收集。收集中段尿10-20ml放入无菌、带垫圈的50ml 塑料管中。在4℃条件下保存并及时送至有关机构。

5、尸检组织样品：病人死亡后应尽早进行尸体解剖，采集肺、气管、心脏、脾、肝、脑、肾和淋巴结等重要组织和器官样品。每采集一部位应更换、消毒采集器械。每种组织和器官应多部位采集，每份样品应采集20-50g，淋巴结2个，分别置于50ml无菌螺口塑料管中，立即置于-70℃以下保存或冷藏送至有关机构。

九、样品采集器械要求。

1、注射器：医用（5ml和10ml）一次性注射器。

2、棉拭子：塑料杆棉签。

3、全血收集管：10ml螺口塑料管(外螺旋、带密封垫圈)。

4、血清保存管：2ml螺口塑料管(外螺旋、带密封垫圈)，可耐深低温（液氮保存）。

5、粪便保存管：50ml螺口塑料管(外螺旋、带密封垫圈)，可耐深低温（液氮保存）。

6、鼻咽拭子保存管：10ml螺口塑料管(外螺旋、带密封垫圈)，可耐深低温（液氮保存）。

7、痰液保存管：50ml螺口塑料管(外螺旋、带密封垫圈)，可耐深低温（液氮保存）。

8、尿液保存管：50ml螺口塑料管（外螺旋、带密封垫圈）。

9、尸检组织冻存管：50ml螺口塑料管(外螺旋、带密封垫圈)。

10、油性，防水的记号笔。

十、样品采集的防护措施。

1、采集样品时，采集人必须穿戴连体式隔离衣、防护鞋套、防护面罩或眼罩、“N95”级防护口罩和乳胶手套（2层）。

2、样品采集完毕，首先消毒并脱掉外层手套，然后戴内层手套依次脱掉帽子、眼罩、口罩、衣裤和鞋套，最后脱掉内层手套，再用消毒巾擦拭面部和双手。

3、防护材料要求：应选用防水面料的防护服、具备宽阔视野，高透光度和防溅性的防护眼镜、外科手术用乳胶手套、“N95”级防护口罩、密封性能好，呼吸阻力较小的全面呼吸防护器。

第二章 保藏

十一、各级疾病预防控制机构在同级卫生行政部门的统一管理下负责SARS人体样品的保藏。地市级疾病预防控制机构负责样品收集和短期保存；省级疾病预防控制机构负责样品的长期安全保藏和管理下级单位及本单位采集的样品；国家疾病预防控制中心负责收集各省保藏的样品，建立专门的国家资源库，长期保藏和管理。

十二、SARS人体样品应使用专用的设备保藏。专用设备应符合以下要求：4℃专用保藏容器，用于呼吸道灌洗液、尿液等样品的短期保存； -20℃以下冰箱，用于保藏血清样品；-70℃或以下深低温冰箱，用于保藏鼻咽拭子、咽拭子、痰液样品及尸检样品。保藏样品的单位应有样品数据的储存和管理设备。

第三章 运输

十三、所有需要运输的SARS人体样品外包装上必须印有生物危险标志并标明“传染性物质，SARS病毒”。短途运输需加冰保冷，使用专车，由2人以上专人运送；长途运送需加干冰保冷；空运应按照民航检疫部门有关传染性物品运输的规定办理。

十四、运输含有活病毒的SARS人体样品或SARS实验样品（如病毒株等）时，必须对样品进行严格的三层包装，避免在运输过程中发生污染。样品的容器要使用能够承受不少于95Kpa的压力的高质量的防水包装材料并且密封，以防止运输过程中发生内容物的外泄；第二层和第三层包装中应使用吸水性好的柔软的物质充填；几个易碎的容器在同一个包裹中运输时，应分别独立包装并相互隔离，以免互相碰撞。

十五、样品运输时所需材料的要求。样品运送液（MEM）：牛血清5%，青霉素2000U/ml，链霉素200ug/ml，制霉菌素25U/ml，用2%NaHCO3调PH值到7.4（由省或市疾病预防控制机构配制提供）；如无样品运送液，也可使用等渗盐溶液或磷酸盐缓冲液；样品密封袋；专用样品运送箱；专用不干胶标签。

第四章 使用与管理

十六、卫生部主管全国SARS人体样品的使用管理工作，负责样品资源的统一调配和使用；地市级以上地方人民政府卫生行政部门负责本行政区域内SARS人体样品的日常监督和管理。

十七、含有人类遗传资源的SARS人体样品（如人体细胞、组织、器官、全血）确需出境的，必须严格按照《人类遗传资源管理暂行办法》规定的程序，报人类遗传资源管理办公室批准；此类样品的入境需报卫生部批准。

十八、不含有人类遗传资源的SARS人体样品（如血清、尿液、粪便、痰液等）和科研样品（如病毒株）需出境和入境的，必须按有关规定报卫生部审批，履行有关手续。

十九、各类企业和个人不得擅自采集、收集和保藏SARS人体样品和科研样品（如病毒株），确需使用SARS人体样品时，须报卫生部批准。

二十、样品的使用单位。地市级以上临床医院可使用本院采集的样品；地市级及以上疾病预防控制机构可使用本机构采集的和下级医疗预防机构采集的样品;有关科研机构和高等学校与临床医院、疾病预防控制机构合作开展相关研究工作时，需报卫生部备案后，方可取得并使用样品。

二十一、SARS人体样品的提供和使用。

1、定点医疗机构负责采集并向所在地市级疾病预防控制机构提供SARS病人、疑似病人样品。有条件的医疗机构可使用本院采集的样品开展血清学检测抗体和PCR法检测病毒RNA工作。

2、地市级疾病预防控制机构负责收集医疗机构和下级疾病预防控制机构采集的SARS人体样品。有条件的疾病预防控制机构可使用本单位采集和收集的样品，开展血清学检测抗体和PCR法检测病毒RNA工作，但不可进行病原分离工作。按规定时间和要求向省级疾病预防控制机构提供所有样品，并附有《传染性非典型肺炎病人或密切接触者人体样品采集登记表》。

3、省级疾病预防控制机构负责接受并保藏地市级疾病预防控制机构提供的所有SARS人体样品及完整的《传染性非典型肺炎病人或密切接触者人体样品采集登记表》。可利用样品开展血清学检测抗体、PCR法检测RNA、SARS病原体分离和鉴定工作。按规定（另发）数量和要求及时向国家疾病预防控制中心提供样品。

4、国家疾病预防控制中心负责接收、统一保藏和管理各省、区、市提供的SARS人体样品和《传染性非典型肺炎病人或密切接触者人体样品采集登记表》，建立专门的SARS人体样品国家资源库，按照卫生部要求向有关机构和单位提供样品。按照卫生部统一部署，开展血清学检测抗体、抗原、PCR法检测RNA工作、病原体分离和鉴定、电镜形态学研究等相关科学研究工作。按疫情的轻重缓急对鼻咽拭子、口咽拭子、痰液、粪便及血清样品进行复核或鉴定工作。

二十二、SARS人体样品接受和检测单位必须及时将检测结果反馈送检单位。

二十三、所有负责收集和保藏SARS人体样品的机构必须指定专人负责样品的使用和管理，对相关工作人员进行培训，严格操作规程，提高自我防护意识。建立监测和登记制度、事故报告制度和应急预案。严格按照国家特殊生物资源保护的有关规定进行管理和保护，不得以任何形式擅自向其他机构和个人提供SARS人体样品。

凡违反有关规定，造成样品丢失、病毒扩散、人员感染及资源流失等严重后果的机构和人员，卫生部将根据国家有关规定和法律法规追究相关单位和个人的行政或法律责任。

1. 生态环境部：国家环境保护总局关于加强非典防治建设项目环境保护管理工作的通知

环发〔2003〕87号

2、涉及二级以上生物安全防护实验室的建设项目，必须严格按照《微生物和生物医学实验室生物安全通用准则》（WS233－2003）等有关规范设计。实验室应采取负压操作，禁止未经消毒、灭活的有害物质流出实验室。实验室废水须经单独消毒处理后再进入污水处理系统。对生物性气态污染物应采取消毒或高温、高压灭活处理，对化学性气态污染物应采取专用通风柜捕集后无害化处理，动物实验室的排风须经活性炭过滤吸收器等设施处理后达标排放。应按国家有关危险废物法规的规定对含有害微生物固体废物、动物残体和二级以上生物安全防护实验室产生的废液等进行无害化处理。

3、新建、扩建和改建专门诊疗“非典”人员的医院及有关卫生防疫项目，必须落实国家有关废水和固体废物的环境保护措施。对于“非典” 病人产生的粪便、尿、呕吐物等排泄物必须配备专用的容器收集，进行单独的消毒处理，不得进入污水处理系统。污水经消毒处理后再进入污水处理系统。在不具备医疗废物集中处理条件的地区，新建、扩建和改建“非典”诊疗医院，应同时建设焚烧装置，就地处理可能被病毒污染的废物，具体要求按我局《“SARS”病毒污染废弃物应急处理处置技术方案》规定执行。

4、按国家有关规定规范污染物排放口、贮存（处置）场，制定并落实有关污染物的监测制度和报告制度。

1. 科技技术部、国家卫生健康委员会、国家药品监督管理总局、生态环境部：《传染性非典型肺炎病毒的毒种保存、使用和感染动物模型的暂行管理办法》

第一条 传染性非典型肺炎属于法定管理传染病，其病原体为传染性非典型肺炎病毒。按《中华人民共和国传染病防治法》的有关规定，特制定本办法。

第二条 国家对传染性非典型肺炎病毒毒种的保存、使用以及感染动物模型建立实行申请、审定制度。未经国家许可,任何单位和个人不得用传染性非典型肺炎病毒和动物模型从事研究活动。

第三条 分离出的传染性非典型肺炎病毒和建立的动物模型要按规定要求登记、上报和核准。

第四条 保存和使用传染性非典型肺炎病毒的单位，必须具备三级生物安全实验室（P3）条件，并须在二级以上生物安全柜中操作。动物模型须在P3和P3级以上实验室中进行。

第五条 病毒毒种保藏：必须具有该病毒毒种的详细历史及有关资料。应在带锁的-80℃超低温冰箱或液氮罐中，用双层套管保存，外层套管须作消毒处理。保存传染性非典型肺炎病毒的冰箱或液氮罐，必须有明确的警示标签。采用双锁双人管理。

第六条 传染性非典型肺炎病毒毒种应有专人负责管理，并建立严格的使用登记制度。

第七条 病毒毒种运输：经申请并由国家主管部门批准后，使用单位应持批准件和本单位证件，派专人（两人或两人以上）领取和携带，不得邮寄。样品的容器要使用能够承受不少于95Kpa压力的高质量的防水包装材料并且密封，以防止运输过程中发生内容物的外泄；第二层和第三层包装中应使用吸水性好的柔软的物质充填；样品的容器须印有生物危险标志。

第八条 研究单位或实验室应具有做疾病或感染动物模型的工作基础与经验，如要进行灵长类动物感染模型研究，必须有做过传染性微生物感染灵长类动物模型的经验。

第九条 建立动物疾病模型必须有医学、兽医学、实验动物学以及具有从事经验的专业人员参加。在实验过程中，必须保证实验动物可随时进行微生物、病理检测，以掌握动物健康状况。

第十条 研究单位或实验室应有动物质量、健康、疾病和感染模型的监控和评价技术。

第十一条 感染用实验动物必须符合国家对科研用动物的相关要求，并附有"实验动物许可证"和"实验动物等级许可证"等背景资料。使用灵长类动物的，必须持有林业部门颁发的"灵长类动物驯养繁殖许可证"。

第十二条 感染非标准化实验动物，包括野生动物，来源必须清楚，须经过检疫后方可使用，在实验过程中需制定其饲养和检测标准。

第十三条 参加实验人员应取得其健康资料，并采取严格的防护措施。

第十四条 使用单位要建立监测制度、事故报告制度和应急措施办法。

第十五条 除国家指定的传染性非典型肺炎病毒保存单位外，经批准使用传染性非典型肺炎病毒研究的单位，在实验过程中应严格安全保存病毒，任务完成后，应在国家派出人员的监督下将传染性非典型肺炎病毒销毁。

1. 科技技术部、国家卫生健康委员会、国家药品监督管理总局、生态环境部：《传染性非典型肺炎病毒研究实验室暂行管理办法》

传染性非典型肺炎是一种严重的传染性疾病。为确保生物安全，防止实验人员感染和污染环境，特制定本办法。

本办法对从事传染性非典型肺炎病毒研究的实验室实行分级管理。实验室分为：传染性非典型肺炎病毒实验室、传染性非典型肺炎病毒感染小动物实验室、传染性非典型肺炎病毒感染大动物实验室。

1. 传染性非典型肺炎病毒实验室

传染性非典型肺炎病毒实验室必须符合中华人民共和国卫生行业标准（WS233-2002）《微生物生物医学实验室生物安全通用准则》中三级实验室（P3）的要求。在使用前要自查，并通过专家考核认证合格后方可使用。

1.1 实验范围

1.1.1 用细胞培养方法分离、培养病毒；

1.1.2 供实验室研究用的病毒收集和浓缩；

1.1.3 除感染动物外的其它传染性非典型肺炎病毒试验。

1.2 实验室条件

1.2.1 实验室应划分为清洁区、半污染区和污染区，各区之间的过渡必须有缓冲区，并有明显的区域标志和负压梯度显示。

1.2.2 实验室各区之间保持气流从清洁区到半污染区再到污染区的单向流动，经过两个串连高效过滤器（HEPA）过滤后排放至大气中，排放口应置在通风良好的环境中，过滤器应安装在靠近实验室的排风口处，所有HEPA必须通过检漏试验合格，并定期消毒、更换。

1.2.3 半污染区和污染区只设上水，不设下水管道。上水管道应设有防止水流回流装置。所有半污染区和污染区的废物（含废弃防护用品）、废水和需取出的物品必须在原地高压蒸汽灭菌，不能高压蒸汽灭菌的必须使用可靠的消毒方法消毒。灭菌、消毒后的废物作为危险废物集中处理。高压蒸汽灭菌器必须具有蒸汽冷凝水自动回收再高压装置。

1.2.4 实验室的生物安全柜应为外排放型二级和三级安全柜，使用时应按WS233-2002附录B检验合格。

1.2.5 所有带病毒的操作或容易产生气溶胶的操作必须在生物安全柜内或负压罩里进行。

1.2.6 实验室排出的气体、消毒后废弃物应进行病毒分离、监测。

2. 传染性非典型肺炎病毒感染小动物实验室

实验室除了达到传染性非典型肺炎病毒实验室要求外，还必须达到如下要求：

2.1 实验必需在三级生物安全柜或负压隔离器内进行，排出气体经高效过滤后，应通过实验室的系统排风管道排出，确保生物安全柜与排风系统的压力平衡。

2.2 操作前，需在乳胶手套外戴布手套，防止乳胶手套破损。

2.3 隔离器内所有物品传出前，应进行化学消毒并装入密封袋，放入带气锁的传递仓，经消毒后，再用密封袋包装，废物放入高压灭菌器灭菌。

2.4 生物安全柜的污物用化学消毒后，装入密封袋放入高压灭菌器灭菌。

2.5 小动物解剖和取样必须在隔离器或生物安全柜内进行。在生物安全柜内进行解剖的小动物，必须先在隔离器内处死并密闭包装后转移至生物安全柜，实验后的废物和动物尸体按2.3和2.4程序处理。

2.6 实验人员须穿正压防护服或穿三套隔离服，可在进入的更衣室一次穿好。戴特制防护帽、护目镜、口罩、防护靴或鞋，戴两副乳胶手套。

2.7 实验结束后，应严格按更衣程序脱去正压防护服和隔离服，并淋浴后，出更衣室。

3. 传染性非典型肺炎病毒感染大动物实验室

实验室除了达到传染性非典型肺炎病毒实验室和传染性非典型肺炎病毒感染小动物实验室要求外，还必须达到如下要求：

3.1 实验用灵长类动物在进入实验前应在清洁区进行清洗，所用废水可排入公共下水道。更衣室的外侧淋浴间下水也排入公共下水道。

3.2 在负压隔离器内饲养动物。应严防动物咬伤、抓伤工作人员，一旦发生，必须立即报告，并隔离观察；防止袖套老化和被动物破坏。

3.3 隔离器内保持足量的过滤通风和负压状态。

3.4 动物取材和处死按2.3、2.4程序进行。

3.5 实验人员在操作时必须穿内防护服和正压防护服，出实验室按2.7程序进行。

4. 组织管理

4.1 管理责任制

4.1.1 单位法人责任制：由单位法人负责组织专门的领导小组和生物安全委员会负责生物安全，制定管理制度，监督有关法规和操作规程的执行。

4.1.2 实验室主任和项目负责人责任制：有权决定本部门进入实验室的工作人员。孕妇、不健康者和无关人员不得进入实验室。

4.1.3 参加实验动物的人员，必须持有《实验动物从业人员岗位证书》。

4.1.4 所有参加实验的人员必须进行一周传染性非典型肺炎病毒实验室操作培训，考核合格后方可从事此项工作。

4.1.5 在实验室的入口处贴上生物安全实验室标志和负责人的姓名和电话号码。

4.2 规章制度

4.2.1 通风空调净化系统、灭菌系统设施的验证与验收规程。

4.2.2 实验室准入制度。

4.2.3 实验室高压、消毒规程。

4.2.4 实验室突发事故处理规程。

4.2.5 实验室设施、设备的监测、检测和维护制度。

4.2.6 制定严格的更衣程序。

4.3 健康医疗监督

4.3.1 所有参加实验的人员必须身体健康，实验前进行体检，采集并保留血清。

4.3.2 在参加动物实验过程中，人员每天检测体温2次，每月进行血常规检查。发现异常时，应立刻到医院就诊。

1. 全国人民代表大会常务委员会：中华人民共和国传染病防治法

疾病预防控制机构、医疗机构的实验室和从事病原微生物实验的单位，应当符合国家规定的条件和技术标准，建立严格的监督管理制度，对传染病病原体样本按照规定的措施实行严格监督管理，严防传染病病原体的实验室感染和病原微生物的扩散。

对传染病菌种、毒种和传染病检测样本的采集、保藏、携带、运输和使用实行分类管理，建立健全严格的管理制度。

1. 全国人民代表大会常务委员会：中华人民共和国固体废物污染环境防治法

各级各类实验室及其设立单位应当加强对实验室产生的固体废物的管理，依法收集、贮存、运输、利用、处置实验室固体废物。实验室固体废物属于危险废物的，应当按照危险废物管理。

1. 国务院：实验动物管理条例

为了加强实验动物的管理工作，保证实验动物质量，适应科学研究、经济建设和社会发展的需要，制定本条例。　本条例所称实验动物，是指经人工饲育，对其携带的微生物实行控制，遗传背景明确或者来源清楚的，用于科学研究、教学、生产、检定以及其他科学实验的动物。

本条例适用于从事实验动物的研究、保种、饲育、供应、应用、管理和监督的单位和个人。

实验动物的管理，应当遵循统一规划、合理分工，有利于促进实验动物科学研究和应用的原则。

国家科学技术委员会主管全国实验动物工作。省、自治区、直辖市科学技术委员会主管本地区的实验动物工作。国务院各有关部门负责管理本部门的实验动物工作。

国家实行实验动物的质量监督和质量合格认证制度。具体办法由国家科学技术委员会另行制定。

实验动物遗传学、微生物学、营养学和饲育环境等方面的国家标准由国家技术监督局制定。

实验动物的饲育管理

从事实验动物饲育工作的单位，必须根据遗传学、微生物学、营养学和饲育环境方面的标准，定期对实验动物进行质量监测。各项作业过程和监测数据应有完整、准确的记录，并建立统计报告制度。

实验动物的饲育室、实验室应设在不同区域，并进行严格隔离。实验动物饲育室、实验室要有科学的管理制度和操作规程。

实验动物的保种、饲育应采用国内或国外认可的品种、品系，并持有效的合格证书。

实验动物必须按照不同来源，不同品种、品系和不同的实验目的，分开饲养。

实验动物分为四级：一级，普通动物；二级，清洁动物；三级，无特定病原体动物；四级，无菌动物。对不同等级的实验动物，应当按照相应的微生物控制标准进行管理。

实验动物必须饲喂质量合格的全价饲料。霉烂、变质、虫蛀、污染的饲料，不得用于饲喂实验动物。直接用作饲料的蔬菜、水果等，要经过清洗消毒，并保持新鲜。

一级实验动物的饮水，应当符合城市生活饮水的卫生标准。二、三、四级实验动物的饮水，应当符合城市生活饮水的卫生标准并经灭菌处理。

验动物的垫料应当按照不同等级实验动物的需要，进行相应处理，达到清洁、干燥、吸水、无毒、无虫、无感染源、无污染。

实验动物的检疫和传染病控制

对引入的实验动物，必须进行隔离检疫。为补充种源或开发新品种而捕捉的野生动物，必须在当地进行隔离检疫，并取得动物检疫部门出具的证明。野生动物运抵实验动物处所，需经再次检疫，方可进入实验动物饲育室。

对必须进行预防接种的实验动物，应当根据实验要求或者按照《中华人民共和国动物防疫法》的有关规定，进行预防接种，但用作生物制品原料的实验动物除外。

实验动物患病死亡的，应当及时查明原因，妥善处理，并记录在案。实验动物患有传染性疾病的，必须立即视情况分别予以销毁或者隔离治疗。对可能被传染的实验动物，进行紧急预防接种，对饲育室内外可能被污染的区域采取严格消毒措施，并报告上级实验动物管理部门和当地动物检疫、卫生防疫单位，采取紧急预防措施，防止疫病蔓延。

实验动物的应用

应用实验动物应当根据不同的实验目的，选用相应的合格实验动物。申报科研课题和鉴定科研成果，应当把应用合格实验动物作为基本条件。应用不合格实验动物取得的检定或者安全评价结果无效，所生产的制品不得使用。

供应用的实验动物应当具备下列完整的资料：品种、品系及亚系的确切名称；遗传背景或其来源；微生物检测状况；合格证书；饲育单位负责人签名。无上述资料的实验动物不得应用。

实验动物的运输工作应当有专人负责。实验动物的装运工具应当安全、可靠。不得将不同品种、品系或者不同等级的实验动物混合装运。

实验动物的进口与出口管理

从国外进口作为原种的实验动物，应附有饲育单位负责人签发的品系和亚系名称以及遗传和微生物状况等资料。无上述资料的实验动物不得进口和应用。

出口应用国家重点保护的野生动物物种开发的实验动物，必须按照国家的有关规定，取得出口许可证后，方可办理出口手续。

进口、出口实验动物的检疫工作，按照《中华人民共和国进出境动植物检疫法》的规定办理。

从事实验动物工作的人员

实验动物工作单位应当根据需要，配备科技人员和经过专业培训的饲育人员。各类人员都要遵守实验动物饲育管理的各项制度，熟悉、掌握操作规程。

实验动物工作单位对直接接触实验动物的工作人员，必须定期组织体格检查。对患有传染性疾病，不宜承担所做工作的人员，应当及时调换工作。

从事实验动物工作的人员对实验动物必须爱护，不得戏弄或虐待。

奖励与处罚

对长期从事实验动物饲育管理，取得显著成绩的单位或者个人，由管理实验动物工作的部门给予表彰或奖励。

对违反本条例规定的单位，由管理实验动物工作的部门视情节轻重，分别给予警告、限期改进、责令关闭的行政处罚。

对违反本条例规定的有关工作人员，由其所在单位视情节轻重，根据国家有关规定，给予行政处分。

1. 国务院：医疗废物管理条例

为了加强医疗废物的安全管理，防止疾病传播，保护环境，保障人体健康，根据《中华人民共和国传染病防治法》和《中华人民共和国固体废物污染环境防治法》，制定本条例。

本条例所称医疗废物，是指医疗卫生机构在医疗、预防、保健以及其他相关活动中产生的具有直接或者间接感染性、毒性以及其他危害性的废物。

医疗废物分类目录，由国务院卫生行政主管部门和环境保护行政主管部门共同制定、公布。

本条例适用于医疗废物的收集、运送、贮存、处置以及监督管理等活动。

医疗卫生机构收治的传染病病人或者疑似传染病病人产生的生活垃圾，按照医疗废物进行管理和处置。

医疗卫生机构废弃的麻醉、精神、放射性、毒性等药品及其相关的废物的管理，依照有关法律、行政法规和国家有关规定、标准执行。

国家推行医疗废物集中无害化处置，鼓励有关医疗废物安全处置技术的研究与开发。

县级以上地方人民政府负责组织建设医疗废物集中处置设施。

国家对边远贫困地区建设医疗废物集中处置设施给予适当的支持。

县级以上各级人民政府卫生行政主管部门，对医疗废物收集、运送、贮存、处置活动中的疾病防治工作实施统一监督管理；环境保护行政主管部门，对医疗废物收集、运送、贮存、处置活动中的环境污染防治工作实施统一监督管理。

县级以上各级人民政府其他有关部门在各自的职责范围内负责与医疗废物处置有关的监督管理工作。

任何单位和个人有权对医疗卫生机构、医疗废物集中处置单位和监督管理部门及其工作人员的违法行为进行举报、投诉、检举和控告。

医疗废物管理的一般规定

医疗卫生机构和医疗废物集中处置单位，应当建立、健全医疗废物管理责任制，其法定代表人为第一责任人，切实履行职责，防止因医疗废物导致传染病传播和环境污染事故。

医疗卫生机构和医疗废物集中处置单位，应当制定与医疗废物安全处置有关的规章制度和在发生意外事故时的应急方案；设置监控部门或者专（兼）职人员，负责检查、督促、落实本单位医疗废物的管理工作，防止违反本条例的行为发生。

医疗卫生机构和医疗废物集中处置单位，应当对本单位从事医疗废物收集、运送、贮存、处置等工作的人员和管理人员，进行相关法律和专业技术、安全防护以及紧急处理等知识的培训。

医疗卫生机构和医疗废物集中处置单位，应当采取有效的职业卫生防护措施，为从事医疗废物收集、运送、贮存、处置等工作的人员和管理人员，配备必要的防护用品，定期进行健康检查；必要时，对有关人员进行免疫接种，防止其受到健康损害。

医疗卫生机构和医疗废物集中处置单位，应当依照《中华人民共和国固体废物污染环境防治法》的规定，执行危险废物转移联单管理制度。

医疗卫生机构和医疗废物集中处置单位，应当对医疗废物进行登记，登记内容应当包括医疗废物的来源、种类、重量或者数量、交接时间、处置方法、最终去向以及经办人签名等项目。登记资料至少保存３年。

医疗卫生机构和医疗废物集中处置单位，应当采取有效措施，防止医疗废物流失、泄漏、扩散。

发生医疗废物流失、泄漏、扩散时，医疗卫生机构和医疗废物集中处置单位应当采取减少危害的紧急处理措施，对致病人员提供医疗救护和现场救援；同时向所在地的县级人民政府卫生行政主管部门、环境保护行政主管部门报告，并向可能受到危害的单位和居民通报。

禁止任何单位和个人转让、买卖医疗废物。

禁止在运送过程中丢弃医疗废物；禁止在非贮存地点倾倒、堆放医疗废物或者将医疗废物混入其他废物和生活垃圾。

禁止邮寄医疗废物。

禁止通过铁路、航空运输医疗废物。

有陆路通道的，禁止通过水路运输医疗废物；没有陆路通道必需经水路运输医疗废物的，应当经设区的市级以上人民政府环境保护行政主管部门批准，并采取严格的环境保护措施后，方可通过水路运输。

禁止将医疗废物与旅客在同一运输工具上载运。

禁止在饮用水源保护区的水体上运输医疗废物。

第三章　医疗卫生机构对医疗废物的管理

医疗卫生机构应当及时收集本单位产生的医疗废物，并按照类别分置于防渗漏、防锐器穿透的专用包装物或者密闭的容器内。

医疗废物专用包装物、容器，应当有明显的警示标识和警示说明。

医疗废物专用包装物、容器的标准和警示标识的规定，由国务院卫生行政主管部门和环境保护行政主管部门共同制定。

医疗卫生机构应当建立医疗废物的暂时贮存设施、设备，不得露天存放医疗废物；医疗废物暂时贮存的时间不得超过２天。

医疗废物的暂时贮存设施、设备，应当远离医疗区、食品加工区和人员活动区以及生活垃圾存放场所，并设置明显的警示标识和防渗漏、防鼠、防蚊蝇、防蟑螂、防盗以及预防儿童接触等安全措施。

医疗废物的暂时贮存设施、设备应当定期消毒和清洁。

医疗卫生机构应当使用防渗漏、防遗撒的专用运送工具，按照本单位确定的内部医疗废物运送时间、路线，将医疗废物收集、运送至暂时贮存地点。

运送工具使用后应当在医疗卫生机构内指定的地点及时消毒和清洁。

医疗卫生机构应当根据就近集中处置的原则，及时将医疗废物交由医疗废物集中处置单位处置。

医疗废物中病原体的培养基、标本和菌种、毒种保存液等高危险废物，在交医疗废物集中处置单位处置前应当就地消毒。

医疗卫生机构产生的污水、传染病病人或者疑似传染病病人的排泄物，应当按照国家规定严格消毒；达到国家规定的排放标准后，方可排入污水处理系统。

不具备集中处置医疗废物条件的农村，医疗卫生机构应当按照县级人民政府卫生行政主管部门、环境保护行政主管部门的要求，自行就地处置其产生的医疗废物。自行处置医疗废物的，应当符合下列基本要求：

（一）使用后的一次性医疗器具和容易致人损伤的医疗废物，应当消毒并作毁形处理；

（二）能够焚烧的，应当及时焚烧；

（三）不能焚烧的，消毒后集中填埋。

第四章　医疗废物的集中处置

从事医疗废物集中处置活动的单位，应当向县级以上人民政府环境保护行政主管部门申请领取经营许可证；未取得经营许可证的单位，不得从事有关医疗废物集中处置的活动。

医疗废物集中处置单位，应当符合下列条件：

（一）具有符合环境保护和卫生要求的医疗废物贮存、处置设施或者设备；

（二）具有经过培训的技术人员以及相应的技术工人；

（三）具有负责医疗废物处置效果检测、评价工作的机构和人员；

（四）具有保证医疗废物安全处置的规章制度。

医疗废物集中处置单位的贮存、处置设施，应当远离居（村）民居住区、水源保护区和交通干道，与工厂、企业等工作场所有适当的安全防护距离，并符合国务院环境保护行政主管部门的规定。

医疗废物集中处置单位应当至少每２天到医疗卫生机构收集、运送一次医疗废物，并负责医疗废物的贮存、处置。

医疗废物集中处置单位运送医疗废物，应当遵守国家有关危险货物运输管理的规定，使用有明显医疗废物标识的专用车辆。医疗废物专用车辆应当达到防渗漏、防遗撒以及其他环境保护和卫生要求。

运送医疗废物的专用车辆使用后，应当在医疗废物集中处置场所内及时进行消毒和清洁。

运送医疗废物的专用车辆不得运送其他物品。

医疗废物集中处置单位在运送医疗废物过程中应当确保安全，不得丢弃、遗撒医疗废物。

医疗废物集中处置单位应当安装污染物排放在线监控装置，并确保监控装置经常处于正常运行状态。

医疗废物集中处置单位处置医疗废物，应当符合国家规定的环境保护、卫生标准、规范。

医疗废物集中处置单位应当按照环境保护行政主管部门和卫生行政主管部门的规定，定期对医疗废物处置设施的环境污染防治和卫生学效果进行检测、评价。检测、评价结果存入医疗废物集中处置单位档案，每半年向所在地环境保护行政主管部门和卫生行政主管部门报告一次。

医疗废物集中处置单位处置医疗废物，按照国家有关规定向医疗卫生机构收取医疗废物处置费用。

医疗卫生机构按照规定支付的医疗废物处置费用，可以纳入医疗成本。

各地区应当利用和改造现有固体废物处置设施和其他设施，对医疗废物集中处置，并达到基本的环境保护和卫生要求。

尚无集中处置设施或者处置能力不足的城市，自本条例施行之日起，设区的市级以上城市应当在１年内建成医疗废物集中处置设施；县级市应当在２年内建成医疗废物集中处置设施。县（旗）医疗废物集中处置设施的建设，由省、自治区、直辖市人民政府规定。

在尚未建成医疗废物集中处置设施期间，有关地方人民政府应当组织制定符合环境保护和卫生要求的医疗废物过渡性处置方案，确定医疗废物收集、运送、处置方式和处置单位。

第五章　监督管理

县级以上地方人民政府卫生行政主管部门、环境保护行政主管部门，应当依照本条例的规定，按照职责分工，对医疗卫生机构和医疗废物集中处置单位进行监督检查。

县级以上地方人民政府卫生行政主管部门，应当对医疗卫生机构和医疗废物集中处置单位从事医疗废物的收集、运送、贮存、处置中的疾病防治工作，以及工作人员的卫生防护等情况进行定期监督检查或者不定期的抽查。

县级以上地方人民政府环境保护行政主管部门，应当对医疗卫生机构和医疗废物集中处置单位从事医疗废物收集、运送、贮存、处置中的环境污染防治工作进行定期监督检查或者不定期的抽查。

卫生行政主管部门、环境保护行政主管部门应当定期交换监督检查和抽查结果。在监督检查或者抽查中发现医疗卫生机构和医疗废物集中处置单位存在隐患时，应当责令立即消除隐患。

卫生行政主管部门、环境保护行政主管部门接到对医疗卫生机构、医疗废物集中处置单位和监督管理部门及其工作人员违反本条例行为的举报、投诉、检举和控告后，应当及时核实，依法作出处理，并将处理结果予以公布。

卫生行政主管部门、环境保护行政主管部门履行监督检查职责时，有权采取下列措施：

（一）对有关单位进行实地检查，了解情况，现场监测，调查取证；

（二）查阅或者复制医疗废物管理的有关资料，采集样品；

（三）责令违反本条例规定的单位和个人停止违法行为；

（四）查封或者暂扣涉嫌违反本条例规定的场所、设备、运输工具和物品；

（五）对违反本条例规定的行为进行查处。

发生因医疗废物管理不当导致传染病传播或者环境污染事故，或者有证据证明传染病传播或者环境污染的事故有可能发生时，卫生行政主管部门、环境保护行政主管部门应当采取临时控制措施，疏散人员，控制现场，并根据需要责令暂停导致或者可能导致传染病传播或者环境污染事故的作业。

医疗卫生机构和医疗废物集中处置单位，对有关部门的检查、监测、调查取证，应当予以配合，不得拒绝和阻碍，不得提供虚假材料。

第六章　法律责任

县级以上地方人民政府未依照本条例的规定，组织建设医疗废物集中处置设施或者组织制定医疗废物过渡性处置方案的，由上级人民政府通报批评，责令限期建成医疗废物集中处置设施或者组织制定医疗废物过渡性处置方案；并可以对政府主要领导人、负有责任的主管人员，依法给予行政处分。

县级以上各级人民政府卫生行政主管部门、环境保护行政主管部门或者其他有关部门，未按照本条例的规定履行监督检查职责，发现医疗卫生机构和医疗废物集中处置单位的违法行为不及时处理，发生或者可能发生传染病传播或者环境污染事故时未及时采取减少危害措施，以及有其他玩忽职守、失职、渎职行为的，由本级人民政府或者上级人民政府有关部门责令改正，通报批评；造成传染病传播或者环境污染事故的，对主要负责人、负有责任的主管人员和其他直接责任人员依法给予降级、撤职、开除的行政处分；构成犯罪的，依法追究刑事责任。

县级以上人民政府环境保护行政主管部门，违反本条例的规定发给医疗废物集中处置单位经营许可证的，由本级人民政府或者上级人民政府环境保护行政主管部门通报批评，责令收回违法发给的证书；并可以对主要负责人、负有责任的主管人员和其他直接责任人员依法给予行政处分。

医疗卫生机构、医疗废物集中处置单位违反本条例规定，有下列情形之一的，由县级以上地方人民政府卫生行政主管部门或者环境保护行政主管部门按照各自的职责责令限期改正，给予警告；逾期不改正的，处２０００元以上５０００元以下的罚款：

（一）未建立、健全医疗废物管理制度，或者未设置监控部门或者专（兼）职人员的；

（二）未对有关人员进行相关法律和专业技术、安全防护以及紧急处理等知识的培训的；

（三）未对从事医疗废物收集、运送、贮存、处置等工作的人员和管理人员采取职业卫生防护措施的；

（四）未对医疗废物进行登记或者未保存登记资料的；

（五）对使用后的医疗废物运送工具或者运送车辆未在指定地点及时进行消毒和清洁的；

（六）未及时收集、运送医疗废物的；

（七）未定期对医疗废物处置设施的环境污染防治和卫生学效果进行检测、评价，或者未将检测、评价效果存档、报告的。

医疗卫生机构、医疗废物集中处置单位违反本条例规定，有下列情形之一的，由县级以上地方人民政府卫生行政主管部门或者环境保护行政主管部门按照各自的职责责令限期改正，给予警告，可以并处５０００元以下的罚款；逾期不改正的，处５０００元以上３万元以下的罚款：

（一）贮存设施或者设备不符合环境保护、卫生要求的；

（二）未将医疗废物按照类别分置于专用包装物或者容器的；

（三）未使用符合标准的专用车辆运送医疗废物或者使用运送医疗废物的车辆运送其他物品的；

（四）未安装污染物排放在线监控装置或者监控装置未经常处于正常运行状态的。

医疗卫生机构、医疗废物集中处置单位有下列情形之一的，由县级以上地方人民政府卫生行政主管部门或者环境保护行政主管部门按照各自的职责责令限期改正，给予警告，并处５０００元以上１万元以下的罚款；逾期不改正的，处１万元以上３万元以下的罚款；造成传染病传播或者环境污染事故的，由原发证部门暂扣或者吊销执业许可证件或者经营许可证件；构成犯罪的，依法追究刑事责任：

（一）在运送过程中丢弃医疗废物，在非贮存地点倾倒、堆放医疗废物或者将医疗废物混入其他废物和生活垃圾的；

（二）未执行危险废物转移联单管理制度的；

（三）将医疗废物交给未取得经营许可证的单位或者个人收集、运送、贮存、处置的；

（四）对医疗废物的处置不符合国家规定的环境保护、卫生标准、规范的；

（五）未按照本条例的规定对污水、传染病病人或者疑似传染病病人的排泄物，进行严格消毒，或者未达到国家规定的排放标准，排入污水处理系统的；

（六）对收治的传染病病人或者疑似传染病病人产生的生活垃圾，未按照医疗废物进行管理和处置的。

医疗卫生机构违反本条例规定，将未达到国家规定标准的污水、传染病病人或者疑似传染病病人的排泄物排入城市排水管网的，由县级以上地方人民政府建设行政主管部门责令限期改正，给予警告，并处５０００元以上１万元以下的罚款；逾期不改正的，处１万元以上３万元以下的罚款；造成传染病传播或者环境污染事故的，由原发证部门暂扣或者吊销执业许可证件；构成犯罪的，依法追究刑事责任。

医疗卫生机构、医疗废物集中处置单位发生医疗废物流失、泄漏、扩散时，未采取紧急处理措施，或者未及时向卫生行政主管部门和环境保护行政主管部门报告的，由县级以上地方人民政府卫生行政主管部门或者环境保护行政主管部门按照各自的职责责令改正，给予警告，并处１万元以上３万元以下的罚款；造成传染病传播或者环境污染事故的，由原发证部门暂扣或者吊销执业许可证件或者经营许可证件；构成犯罪的，依法追究刑事责任。

医疗卫生机构、医疗废物集中处置单位，无正当理由，阻碍卫生行政主管部门或者环境保护行政主管部门执法人员执行职务，拒绝执法人员进入现场，或者不配合执法部门的检查、监测、调查取证的，由县级以上地方人民政府卫生行政主管部门或者环境保护行政主管部门按照各自的职责责令改正，给予警告；拒不改正的，由原发证部门暂扣或者吊销执业许可证件或者经营许可证件；触犯《中华人民共和国治安管理处罚法》，构成违反治安管理行为的，由公安机关依法予以处罚；构成犯罪的，依法追究刑事责任。

不具备集中处置医疗废物条件的农村，医疗卫生机构未按照本条例的要求处置医疗废物的，由县级人民政府卫生行政主管部门或者环境保护行政主管部门按照各自的职责责令限期改正，给予警告；逾期不改正的，处１０００元以上５０００元以下的罚款；造成传染病传播或者环境污染事故的，由原发证部门暂扣或者吊销执业许可证件；构成犯罪的，依法追究刑事责任。

未取得经营许可证从事医疗废物的收集、运送、贮存、处置等活动的，由县级以上地方人民政府环境保护行政主管部门责令立即停止违法行为，没收违法所得，可以并处违法所得１倍以下的罚款。

转让、买卖医疗废物，邮寄或者通过铁路、航空运输医疗废物，或者违反本条例规定通过水路运输医疗废物的，由县级以上地方人民政府环境保护行政主管部门责令转让、买卖双方、邮寄人、托运人立即停止违法行为，给予警告，没收违法所得；违法所得５０００元以上的，并处违法所得２倍以上５倍以下的罚款；没有违法所得或者违法所得不足５０００元的，并处５０００元以上２万元以下的罚款。

承运人明知托运人违反本条例的规定运输医疗废物，仍予以运输的，或者承运人将医疗废物与旅客在同一工具上载运的，按照前款的规定予以处罚。

医疗卫生机构、医疗废物集中处置单位违反本条例规定，导致传染病传播或者发生环境污染事故，给他人造成损害的，依法承担民事赔偿责任。

1. 国务院办公厅：国务院办公厅转发卫生计生委等部门关于切实履行《国际卫生条例2005》加快推进公共卫生应急核心能力建设指导意见的通知

国办发〔2013〕84号

"加强实验室检测能力建设和生物安全管理：各级人民政府要建立各类突发急性传染病检测试剂的有效供给保障机制，提高疾病预防控制机构及有条件的医疗机构的实验室检测、疾病诊断和确诊能力。卫生计生部门要加强高等级生物实验室检测能力建设，完善公共卫生应急联合检测工作机制，建立健全突发公共卫生事件实验室应急检测网络。质检部门加强对通过质量认证实验室的调查工作，建立健全实验室目录数据库。

各级卫生计生、农业、质检、食品药品监管、林业等部门要加强病原微生物实验室和实验活动、菌毒种保藏与提供的监管，特别是要给予基层实验室生物安全监管和培训支持，建立健全实验室质量管理体系和卫生应急检测的标准方法。各级卫生计生、农业、质检、民航等部门要完善危险品样本特别是感染性样本的采集、包装和运输工具的保障机制，加强实验室检验人员、感染性样本运输技术人员和管理人员的培养与教育，强化机场运输感染性样本的能力建设，确保机场和相关人员资质、感染性样本包装容器符合航空运输要求。"

1. 国家卫生健康委员会：关于进一步做好新冠病毒实验室生物安全管理工作的通知

国卫办科教函〔2023〕13号

随着新冠病毒感染疫情防控政策的优化调整，病原微生物实验室生物安全面临新的形势和任务。为保障实验室生物安全，防范生物安全风险，依据《中华人民共和国生物安全法》及《病原微生物实验室生物安全管理条例》等有关规定，现就进一步做好新冠病毒实验室生物安全管理工作通知如下。

    一 、关于新冠病毒实验活动管理

    新冠病毒继续按照第二类病原微生物进行管理。新冠病毒培养、动物感染实验应当在生物安全三级及以上实验室开展；未经培养的感染性材料的操作、灭活材料的操作应当在生物安全二级及以上实验室进行，其中未经可靠灭活或固定的人和动物组织标本的操作应当在三级及以上实验室开展；无感染性材料的操作可以在生物安全一级实验室进行。在三级及以上实验室开展的实验活动，开展前应当依法经过批准。实验室应当根据风险评估结果，选择适宜的个人防护用品。

    二、关于新冠病毒运输管理

    新冠病毒毒株或未经培养的潜在感染性生物材料样本属于A类感染性物质，对应的联合国编号为UN2814,包装应当符合世界卫生组织《感染性物质运输规章指导》的PI620分类包装要求，运输应当经省级以上卫生健康行政部门批准。环境样本按照B类感染性物质管理，对应的联合国编号为UN3373,包装应当符合世界卫生组织《感染性物质运输规章指导》的PI650分类包装要求。感染性物质运输应当遵守相应交通运输主管部门关于危险货物运输的规定。灭活样本按照非感染性材料管理。

    三、关于新冠病毒毒株和样本管理

    新冠病毒毒株和感染性样本应当由专人集中管理，准确记录毒株和感染性样本的来源、种类、数量，建立台账进行登记编号，采取有效措施确保安全。各实验室及其设立单位要特别加强新冠病毒既往流行毒株和感染性样本及其实验活动的管理。实验活动结束后，实验室应当在6个月内将毒株送交保藏机构保藏或者就地销毁；感染性样本原则上就地销毁或灭活处理，对确需保存的非灭活样本，应当及时送交具备保存条件的机构保存，或者送交保藏机构保藏。实验室分离到新出现的变异毒株，应在90天内送交保藏机构保藏。

    四、加强实验室生物安全监管

   实验室设立单位要切实落实主体责任，加强实验室生物安全管理，建立健全管理制度，强化人员培训，严格按照法律法规及相关标准规范操作，定期开展自查，及时排查消除风险隐患。各级卫生健康行政部门要依法履行监管职责，提升实验室生物安全监管能力，按照属地化、分级分类的原则，认真做好实验室相关许可和备案工作，强化实验室监督检查，督促实验室及其设立单位规范管理，发现违法违规行为要坚决依法严肃查处。

1. 国家卫生健康委员会：人间传染的病原微生物菌（毒）种保藏机构指定工作细则

卫科教发〔2011〕43号

第一章 总 则

第一条 为贯彻落实《病原微生物实验室生物安全管理条例》和《人间传染的病原微生物菌（毒）种保藏机构管理办法》（以下简称《办法》），做好人间传染的病原微生物菌（毒）种保藏机构指定工作，依照科学、规范、公开的原则，制定本细则。

第二条 符合《办法》和卫生部有关规定的单位方可申请。

第二章 申 请

第三条 申请人间传染的病原微生物菌（毒）种保藏机构的单位应当具备《办法》第十三条规定的条件。

第四条 申请人间传染的病原微生物菌（毒）种保藏机构资格应当填写《人间传染的病原微生物菌（毒）种保藏机构申请表》（以下简称《申请表》），并按照《办法》第十四条的规定，提交完整的资料。

第五条 所有申请资料应当一式2份（申请资料应当使用A4规格纸张打印，中文使用宋体小4号字，英文使用12号字）。申报的各项内容应当完整、清楚。申请资料的复印件应当足够清楚并与原件一致。所有申请资料应当加盖申请单位公章。

第六条 申请单位将符合要求的申请资料报送省级卫生行政部门。省级卫生行政部门收到材料后，在15个工作日内对申请材料进行审核，审核同意的报送卫生部。

省级卫生行政部门应当直接向卫生部报送资料，不得委托申请单位向卫生部报送资料。

第七条 卫生部在收到省级卫生行政部门报送材料之日起的5个工作日内，对申报材料进行形式审查，对于符合要求的，在60个工作日内组织专家进行评估和论证。对于不符合形式审查要求的，卫生部应当及时通知省级卫生行政部门，由省级卫生行政部门告知申请单位补正。

第三章 现场评估论证

第八条 卫生部组织专家组进行现场评估论证，应当将有关要求提前5个工作日书面告知申请单位。

第九条 专家组由5～7名相关专业的专家组成，主要专家由卫生部从病原微生物实验室生物安全评审专家委员会中聘请，并可根据评审专业的需要，聘请1—2名非评委会专家。专家组组长由卫生部指定，为现场评估论证工作技术总负责人。

卫生部可指派1~2名管理或专业人员以观察员的身份参加现场评估论证工作。

第十条 专家组成员如与申请单位有利害关系，应主动提出回避。申请单位也可要求其回避。

有利害关系是指三年内曾在申请单位任职（包括一般工作）或担任顾问，配偶或直系亲属在申请单位中任职或担任顾问，与申请单位发生过法律纠纷，以及其他可能影响公正评审的情况。

第十一条 现场评估论证时间一般为2~3天，有特殊情况需要延长的，经专家组半数以上成员同意，可适当延长。在现场评估论证前，专家组组长应当提前制定现场技术考核初步计划。

第十二条 现场评估程序包括：专家组预备会议、首次会议、资料审查、保藏机构考察、现场模拟操作考核、理论知识测试、专家组内部会议、末次会议等。

专家组依据计划进行现场评估论证，申请单位应积极配合，并提供相应协助。

第十三条 专家组在现场评估论证工作开始前召开全体专家组成员参加的预备会议，会议内容包括：

（一）专家组长重申评估论证工作的公正、客观、保密要求，专家组全体人员签署公正性声明和保密协议；

（二）明确评估范围、内容、依据和要求；

（三）明确工作日程和专家组成员分工，确定现场技术考核计划，准备现场评估和论证所需考核试题等有关资料和表格。

第十四条 召开首次会议。参加会议人员包括专家组成员、申请单位负责人及相关人员。会议由专家组组长主持，会议程序及内容如下：

（一）介绍专家组成员和分工；

（二）宣布现场评估论证工作安排、要求和时间表；

（三）明确评估的方法、程序和评定原则；

（四）向申请单位做公正和保密的承诺；

（五）申请单位负责人报告工作情况；

（六）与申请单位确认现场评估所需现场操作和面试考核项目以及被考核人员名单。

第十五条 资料审查。专家组审查实验室生物安全手册、程序文件、危害评估报告、标准操作程序、相关记录表格以及《人间传染的病原微生物菌（毒）种保藏机构现场检查表》涉及的其他资料，并对审查情况进行记录。

第十六条 保藏机构考察。由专家组根据《人间传染的病原微生物菌（毒）种保藏机构现场检查表》的内容对保藏机构进行实地考察，并对考察情况进行记录。

第十七条 现场模拟操作考核。由专家组从实验室操作人员名单中抽取30%的人员进行现场操作考核，并对考核情况进行记录。

现场模拟操作考核题目由专家组制订，现场操作应涉及申请范围的主要项目，应当覆盖主要仪器设备、主要人员和主要操作技术。

由每名参试人员抽取1个题目进行现场操作，由2位专家组成员进行评判。评判标准依据实验室的标准操作程序。

第十八条 理论知识测试。采取面试形式，除参加现场模拟操作考核的人员以外的其他实验室人员均应参加。由2位专家组成员组成考核组，对每名被考核人员进行单独面试并进行评判。

面试考核内容及评判标准依据为《中华人民共和国传染病防治法》、《病原微生物实验室生物安全管理条例》、《人间传染的高致病性病原微生物实验室和实验活动生物安全审批管理办法》（卫生部令第50号）、《人间传染的病原微生物名录》、《可感染人类的高致病性病原微生物菌（毒）种或样本运输管理规定》（卫生部令第45号）、《办法》、《实验室生物安全通用要求》（GB 19489）、《人间传染的病原微生物菌（毒）种保藏机构设置技术规范》（WS315-2010）、本实验室生物安全手册、程序文件、标准操作程序（SOP）以及世界卫生组织生物安全手册第三版等相关内容。

接受现场操作及面试考核的人员中，未合格者应当重新培训，经考核合格后方能上岗。

第十九条 专家组内部会议。由专家组组长主持，全体专家组成员参加，会议程序及内容：

（一）专家组成员分别报告资料审查、现场模拟操作考核、理论和知识测试、保藏机构检查等结果，讨论并提出评估论证意见；

（二）编写并通过《人间传染的病原微生物菌（毒）种保藏机构现场评估论证报告》。

第二十条 末次会议。会议由专家组组长主持，参加人员包括专家组成员、申请单位负责人及相关人员。会议程序及内容：

（一）专家组组长宣读评估论证报告及审查结论；

（二）专家组指出存在的问题，提出整改建议；

（三）专家组与申请单位人员沟通交流意见。

第二十一条 专家组长应在现场评估论证结束之日起5个工作日内将评估论证报告、原始记录及有关资料移交卫生部。评估论证报告应当由专家组全体成员签字。

申请单位应按照专家组提出的整改意见，在三个月内完成整改工作，并向卫生部提交整改报告。卫生部在收到整改报告之日起的20个工作日内完成整改复核工作。整改复核工作由原现场评估论证专家组成员完成。

第四章 指 定

第二十二条 卫生部在收到专家组评估论证报告或者整改复核意见之日起30个工作日内进行审核，做出是否同意指定的决定。对同意指定的，由卫生部颁发《人间传染的病原微生物菌（毒）种保藏机构证书》（以下简称《证书》），并通知保藏机构所在地的省级卫生行政部门。对不同意指定的，由卫生部书面通知申请单位，并说明理由。申请单位对卫生部的指定结论有异议的，可以自收到通知书之日起20个工作日内书面向卫生部提出复核申请，逾期不予受理。

未被指定的单位，自收到书面通知之日起6个月后可重新申请。

第二十三条《证书》有效期5年。保藏机构需要继续从事保藏工作的，应当在有效期届满前6个月按照本办法的规定重新申请《证书》。

第二十四条 取得《证书》的保藏机构发生《办法》第十五条规定的变化时，应当及时向省级卫生行政部门报告，省级卫生行政部门经核查后报卫生部。

第五章 评估论证工作纪律

第二十五条 专家组成员要严格按照规定的时间到达和离开现场评估目的地。评审期间，专家组成员不得私下与申请单位联系和接触、传递评审相关信息。

第二十六条 专家组成员应严格遵守廉洁自律各项规定，不得接受申请单位接待，不得接受财物或其他不正当利益，不得提出任何与评审工作无关的要求。

第二十七条 现场评估过程中，申请单位不得弄虚作假或者通过任何形式对评审人员施加压力，不得以各种理由不予配合或拒绝检查。如果出现上述问题，专家组有权终止评审。

第六章 附 则

第二十八条 本细则自颁布之日起施行。

1. 国家卫生健康委员会：医疗卫生机构医疗废物管理办法

中华人民共和国卫生部令第36号

第一章　总　　则

第一条　为规范医疗卫生机构对医疗废物的管理，有效预防和控制医疗废物对人体健康和环境产生危害，根据《医疗废物管理条例》，制定本办法。

第二条　各级各类医疗卫生机构应当按照《医疗废物管理条例》和本办法的规定对医疗废物进行管理。

第三条　卫生部对全国医疗卫生机构的医疗废物管理工作实施监督。

县级以上地方人民政府卫生行政主管部门对本行政区域医疗卫生机构的医疗废物管理工作实施监督。

第二章　医疗卫生机构对医疗废物的管理职责

第四条　医疗卫生机构应当建立、健全医疗废物管理责任制，其法定代表人或者主要负责人为第一责任人，切实履行职责，确保医疗废物的安全管理。

第五条　医疗卫生机构应当依据国家有关法律、行政法规、部门规章和规范性文件的规定，制定并落实医疗废物管理的规章制度、工作流程和要求、有关人员的工作职责及发生医疗卫生机构内医疗废物流失、泄漏、扩散和意外事故的应急方案。内容包括：

(一)医疗卫生机构内医疗废物各产生地点对医疗废物分类收集方法和工作要求；

(二)医疗卫生机构内医疗废物的产生地点、暂时贮存地点的工作制度及从产生地点运送至暂时贮存地点的工作要求；

(三)医疗废物在医疗卫生机构内部运送及将医疗废物交由医疗废物处置单位的有关交接、登记的规定；

(四)医疗废物管理过程中的特殊操作程序及发生医疗废物流失、泄漏、扩散和意外事故的紧急处理措施；

(五)医疗废物分类收集、运送、暂时贮存过程中有关工作人员的职业卫生安全防护。

第六条　医疗卫生机构应当设置负责医疗废物管理的监控部门或者专(兼)职人员，履行以下职责：

(一)负责指导、检查医疗废物分类收集、运送、暂时贮存及机构内处置过程中各项工作的落实情况；

(二)负责指导、检查医疗废物分类收集、运送、暂时贮存及机构内处置过程中的职业卫生安全防护工作；

(三)负责组织医疗废物流失、泄漏、扩散和意外事故发生时的紧急处理工作；

(四)负责组织有关医疗废物管理的培训工作；

(五)负责有关医疗废物登记和档案资料的管理；

(六)负责及时分析和处理医疗废物管理中的其他问题。

第七条　医疗卫生机构发生医疗废物流失、泄漏、扩散和意外事故时，应当按照《医疗废物管理条例》和本办法的规定采取相应紧急处理措施，并在48小时内向所在地的县级人民政府卫生行政主管部门、环境保护行政主管部门报告。调查处理工作结束后，医疗卫生机构应当将调查处理结果向所在地的县级人民政府卫生行政主管部门、环境保护行政主管部门报告。

县级人民政府卫生行政主管部门每月汇总逐级上报至当地省级人民政府卫生行政主管部门。

省级人民政府卫生行政主管部门每半年汇总后报卫生部。

第八条　医疗卫生机构发生因医疗废物管理不当导致1人以上死亡或者3人以上健康损害，需要对致病人员提供医疗救护和现场救援的重大事故时，应当在12小时内向所在地的县级人民政府卫生行政主管部门报告，并按照《医疗废物管理条例》和本办法的规定，采取相应紧急处理措施。

县级人民政府卫生行政主管部门接到报告后，应当在12小时内逐级向省级人民政府卫生行政主管部门报告。

医疗卫生机构发生因医疗废物管理不当导致3人以上死亡或者10人以上健康损害，需要对致病人员提供医疗救护和现场救援的重大事故时，应当在2小时内向所在地的县级人民政府卫生行政主管部门报告，并按照《医疗废物管理条例》和本办法的规定，采取相应紧急处理措施。

县级人民政府卫生行政主管部门接到报告后，应当在6小时内逐级向省级人民政府卫生行政主管部门报告。

省级人民政府卫生行政主管部门接到报告后，应当在6小时内向卫生部报告。

发生医疗废物管理不当导致传染病传播事故，或者有证据证明传染病传播的事故有可能发生时，应当按照《传染病防治法》及有关规定报告，并采取相应措施。

第九条　医疗卫生机构应当根据医疗废物分类收集、运送、暂时贮存及机构内处置过程中所需要的专业技术、职业卫生安全防护和紧急处理知识等，制订相关工作人员的培训计划并组织实施。

第三章　分类收集、运送与暂时贮存

第十条　医疗卫生机构应当根据《医疗废物分类目录》，对医疗废物实施分类管理。

第十一条　医疗卫生机构应当按照以下要求，及时分类收集医疗废物：

(一)根据医疗废物的类别，将医疗废物分置于符合《医疗废物专用包装物、容器的标准和警示标识的规定》的包装物或者容器内；

(二)在盛装医疗废物前，应当对医疗废物包装物或者容器进行认真检查，确保无破损、渗漏和其他缺陷；

(三)感染性废物、病理性废物、损伤性废物、药物性废物及化学性废物不能混合收集。少量的药物性废物可以混入感染性废物，但应当在标签上注明；

(四)废弃的麻醉、精神、放射性、毒性等药品及其相关的废物的管理，依照有关法律、行政法规和国家有关规定、标准执行；

(五)化学性废物中批量的废化学试剂、废消毒剂应当交由专门机构处置；

(六)批量的含有汞的体温计、血压计等医疗器具报废时，应当交由专门机构处置；

(七)医疗废物中病原体的培养基、标本和菌种、毒种保存液等高危险废物，应当首先在产生地点进行压力蒸汽灭菌或者化学消毒处理，然后按感染性废物收集处理；

(八)隔离的传染病病人或者疑似传染病病人产生的具有传染性的排泄物，应当按照国家规定严格消毒，达到国家规定的排放标准后方可排入污水处理系统；

(九)隔离的传染病病人或者疑似传染病病人产生的医疗废物应当使用双层包装物，并及时密封；

(十)放入包装物或者容器内的感染性废物、病理性废物、损伤性废物不得取出。

第十二条　医疗卫生机构内医疗废物产生地点应当有医疗废物分类收集方法的示意图或者文字说明。

第十三条　盛装的医疗废物达到包装物或者容器的3/4时，应当使用有效的封口方式，使包装物或者容器的封口紧实、严密。

第十四条　包装物或者容器的外表面被感染性废物污染时，应当对被污染处进行消毒处理或者增加一层包装。

第十五条　盛装医疗废物的每个包装物、容器外表面应当有警示标识，在每个包装物、容器上应当系中文标签，中文标签的内容应当包括：医疗废物产生单位、产生日期、类别及需要的特别说明等。

第十六条　运送人员每天从医疗废物产生地点将分类包装的医疗废物按照规定的时间和路线运送至内部指定的暂时贮存地点。

第十七条　运送人员在运送医疗废物前，应当检查包装物或者容器的标识、标签及封口是否符合要求，不得将不符合要求的医疗废物运送至暂时贮存地点。

第十八条　运送人员在运送医疗废物时，应当防止造成包装物或容器破损和医疗废物的流失、泄漏和扩散，并防止医疗废物直接接触身体。

第十九条　运送医疗废物应当使用防渗漏、防遗撒、无锐利边角、易于装卸和清洁的专用运送工具。

每天运送工作结束后，应当对运送工具及时进行清洁和消毒。

第二十条　医疗卫生机构应当建立医疗废物暂时贮存设施、设备，不得露天存放医疗废物；医疗废物暂时贮存的时间不得超过2天。

第二十一条　医疗卫生机构建立的医疗废物暂时贮存设施、设备应当达到以下要求：

(一)远离医疗区、食品加工区、人员活动区和生活垃圾存放场所，方便医疗废物运送人员及运送工具、车辆的出入；

(二)有严密的封闭措施，设专(兼)职人员管理，防止非工作人员接触医疗废物；

(三)有防鼠、防蚊蝇、防蟑螂的安全措施；

(四)防止渗漏和雨水冲刷；

(五)易于清洁和消毒；

(六)避免阳光直射；

(七)设有明显的医疗废物警示标识和“禁止吸烟、饮食”的警示标识。

第二十二条　暂时贮存病理性废物，应当具备低温贮存或者防腐条件。

第二十三条　医疗卫生机构应当将医疗废物交由取得县级以上人民政府环境保护行政主管部门许可的医疗废物集中处置单位处置，依照危险废物转移联单制度填写和保存转移联单。

第二十四条　医疗卫生机构应当对医疗废物进行登记，登记内容应当包括医疗废物的来源、种类、重量或者数量、交接时间、最终去向以及经办人签名等项目。登记资料至少保存3年。

第二十五条　医疗废物转交出去后，应当对暂时贮存地点、设施及时进行清洁和消毒处理。

第二十六条　禁止医疗卫生机构及其工作人员转让、买卖医疗废物。

禁止在非收集、非暂时贮存地点倾倒、堆放医疗废物，禁止将医疗废物混入其它废物和生活垃圾。

第二十七条　不具备集中处置医疗废物条件的农村地区，医疗卫生机构应当按照当地卫生行政主管部门和环境保护行政主管部门的要求，自行就地处置其产生的医疗废物。自行处置医疗废物的，应当符合以下基本要求：

(一)使用后的一次性医疗器具和容易致人损伤的医疗废物应当消毒并作毁形处理；

(二)能够焚烧的，应当及时焚烧；

(三)不能焚烧的，应当消毒后集中填埋。

第二十八条　医疗卫生机构发生医疗废物流失、泄漏、扩散和意外事故时，应当按照以下要求及时采取紧急处理措施：

(一)确定流失、泄漏、扩散的医疗废物的类别、数量、发生时间、影响范围及严重程度；

(二)组织有关人员尽快按照应急方案，对发生医疗废物泄漏、扩散的现场进行处理；

(三)对被医疗废物污染的区域进行处理时，应当尽可能减少对病人、医务人员、其它现场人员及环境的影响；

(四)采取适当的安全处置措施，对泄漏物及受污染的区域、物品进行消毒或者其他无害化处置，必要时封锁污染区域，以防扩大污染；

(五)对感染性废物污染区域进行消毒时，消毒工作从污染最轻区域向污染最严重区域进行，对可能被污染的所有使用过的工具也应当进行消毒；

(六)工作人员应当做好卫生安全防护后进行工作。

处理工作结束后，医疗卫生机构应当对事件的起因进行调查，并采取有效的防范措施预防类似事件的发生。

第四章人员培训和职业安全防护

第二十九条　医疗卫生机构应当对本机构工作人员进行培训，提高全体工作人员对医疗废物管理工作的认识。对从事医疗废物分类收集、运送、暂时贮存、处置等工作的人员和管理人员，进行相关法律和专业技术、安全防护以及紧急处理等知识的培训。

第三十条　医疗废物相关工作人员和管理人员应当达到以下要求：

(一)掌握国家相关法律、法规、规章和有关规范性文件的规定，熟悉本机构制定的医疗废物管理的规章制度、工作流程和各项工作要求；

(二)掌握医疗废物分类收集、运送、暂时贮存的正确方法和操作程序；

(三)掌握医疗废物分类中的安全知识、专业技术、职业卫生安全防护等知识；

(四)掌握在医疗废物分类收集、运送、暂时贮存及处置过程中预防被医疗废物刺伤、擦伤等伤害的措施及发生后的处理措施；

(五)掌握发生医疗废物流失、泄漏、扩散和意外事故情况时的紧急处理措施。

第三十一条　医疗卫生机构应当根据接触医疗废物种类及风险大小的不同，采取适宜、有效的职业卫生防护措施，为机构内从事医疗废物分类收集、运送、暂时贮存和处置等工作的人员和管理人员配备必要的防护用品，定期进行健康检查，必要时，对有关人员进行免疫接种，防止其受到健康损害。

第三十二条医疗卫生机构的工作人员在工作中发生被医疗废物刺伤、擦伤等伤害时，应当采取相应的处理措施，并及时报告机构内的相关部门。

第五章　监督管理

第三十三条　县级以上地方人民政府卫生行政主管部门应当依照《医疗废物管理条例》和本办法的规定，对所辖区域的医疗卫生机构进行定期监督检查和不定期抽查。

第三十四条　对医疗卫生机构监督检查和抽查的主要内容是：

(一)医疗废物管理的规章制度及落实情况；

(二)医疗废物分类收集、运送、暂时贮存及机构内处置的工作状况；

(三)有关医疗废物管理的登记资料和记录；

(四)医疗废物管理工作中，相关人员的安全防护工作；

(五)发生医疗废物流失、泄漏、扩散和意外事故的上报及调查处理情况；

(六)进行现场卫生学监测。

第三十五条　卫生行政主管部门在监督检查或者抽查中发现医疗卫生机构存在隐患时，应当责令立即消除隐患。

第三十六条　县级以上卫生行政主管部门应当对医疗卫生机构发生违反《医疗废物管理条例》和本办法规定的行为依法进行查处。

第三十七条　发生因医疗废物管理不当导致传染病传播事故，或者有证据证明传染病传播的事故有可能发生时，卫生行政主管部门应当按照《医疗废物管理条例》第四十条的规定及时采取相应措施。

第三十八条　医疗卫生机构对卫生行政主管部门的检查、监测、调查取证等工作，应当予以配合，不得拒绝和阻碍，不得提供虚假材料。

第六章　罚　　则

第三十九条　医疗卫生机构违反《医疗废物管理条例》及本办法规定，有下列情形之一的，由县级以上地方人民政府卫生行政主管部门责令限期改正、给予警告；逾期不改正的，处以2000元以上5000元以下的罚款：

(一)未建立、健全医疗废物管理制度，或者未设置监控部门或者专(兼)职人员的；

(二)未对有关人员进行相关法律和专业技术、安全防护以及紧急处理等知识的培训的；

(三)未对医疗废物进行登记或者未保存登记资料的；

(四)未对机构内从事医疗废物分类收集、运送、暂时贮存、处置等工作的人员和管理人员采取职业卫生防护措施的；

(五)未对使用后的医疗废物运送工具及时进行清洁和消毒的；

(六)自行建有医疗废物处置设施的医疗卫生机构，未定期对医疗废物处置设施的卫生学效果进行检测、评价，或者未将检测、评价效果存档、报告的。

第四十条　医疗卫生机构违反《医疗废物管理条例》及本办法规定，有下列情形之一的，由县级以上地方人民政府卫生行政主管部门责令限期改正、给予警告，可以并处5000元以下的罚款；逾期不改正的，处5000元以上3万元以下的罚款：

(一)医疗废物暂时贮存地点、设施或者设备不符合卫生要求的；

(二)未将医疗废物按类别分置于专用包装物或者容器的；

(三)使用的医疗废物运送工具不符合要求的。

第四十一条　医疗卫生机构违反《医疗废物管理条例》及本办法规定，有下列情形之一的，由县级以上地方人民政府卫生行政主管部门责令限期改正，给予警告，并处5000元以上1万以下的罚款；逾期不改正的，处1万元以上3万元以下的罚款；造成传染病传播的，由原发证部门暂扣或者吊销医疗卫生机构执业许可证件；构成犯罪的，依法追究刑事责任：

(一)在医疗卫生机构内丢弃医疗废物和在非贮存地点倾倒、堆放医疗废物或者将医疗废物混入其他废物和生活垃圾的；

(二)将医疗废物交给未取得经营许可证的单位或者个人的；

(三)未按照条例及本办法的规定对污水、传染病病人和疑似传染病病人的排泄物进行严格消毒，或者未达到国家规定的排放标准，排入污水处理系统的；

(四)对收治的传染病病人或者疑似传染病病人产生的生活垃圾，未按照医疗废物进行管理和处置的。

第四十二条　医疗卫生机构转让、买卖医疗废物的，依照《医疗废物管理条例》第五十三条处罚。

第四十三条　医疗卫生机构发生医疗废物流失、泄漏、扩散时，未采取紧急处理措施，或者未及时向卫生行政主管部门报告的，由县级以上地方人民政府卫生行政主管部门责令改正，给予警告，并处1万元以上3万元以下的罚款；造成传染病传播的，由原发证部门暂扣或者吊销医疗卫生机构执业许可证件；构成犯罪的，依法追究刑事责任。

第四十四条　医疗卫生机构无正当理由，阻碍卫生行政主管部门执法人员执行职务，拒绝执法人员进入现场，或者不配合执法部门的检查、监测、调查取证的，由县级以上地方人民政府卫生行政主管部门责令改正，给予警告；拒不改正的，由原发证部门暂扣或者吊销医疗卫生机构执业许可证件；触犯《中华人民共和国治安管理处罚条例》，构成违反治安管理行为的，由公安机关依法予以处罚；构成犯罪的，依法追究刑事责任。

第四十五条　不具备集中处置医疗废物条件的农村，医疗卫生机构未按照《医疗废物管理条例》和本办法的要求处置医疗废物的，由县级以上地方人民政府卫生行政主管部门责令限期改正，给予警告；逾期不改的，处1000元以上5000元以下的罚款；造成传染病传播的，由原发证部门暂扣或者吊销医疗卫生机构执业许可证件；构成犯罪的，依法追究刑事责任。

第四十六条　医疗卫生机构违反《医疗废物管理条例》及本办法规定，导致传染病传播，给他人造成损害的，依法承担民事赔偿责任。

1. 中国合格评定国家认可委员会：关于发布《实验室生物安全认可规则》(CNAS―RL05：2016)等认可规范文件的通知

1 范围

本规则适用于CNAS 进行实验室生物安全认可体系运作的程序规则。

2 引用文件

下列文件中的条款通过引用而成为本文件的条款。

2.1《病原微生物实验室生物安全管理条例》

2.2《中华人民共和国认证认可条例》

2.3《中国合格评定国家认可委员会章程》

2.4 GB19489《实验室 生物安全通用要求》

2.5 CNAS-RL01《实验室认可规则》

2.6 CNAS-R01《认可标识使用和认可状态声明规则》

2.7 CNAS-R02《公正性和保密规则》

2.8 CNAS-R03《申诉、投诉和争议处理规则》

3 术语和定义

本规则引用上述文件（见2）中的有关术语并采用下列定义：

3.1 病原微生物：指能够使人或者动物致病的微生物。

3.2 实验室：涉及生物因子操作的实验室。

3.3 实验活动：指实验室从事与病原微生物菌（毒）种、样本有关的研究、教学、检测、诊断等活动。

3.4 申请人：正在寻求认可的机构。

4 申请认可条件

4.1 实验室具有明确的法律地位，具备承担相应法律责任的能力。

4.2 实验室的设立符合《病原微生物实验室生物安全管理条例》的有关规定。

4.3 通过自我评价，符合CNAS实验室生物安全认可准则的要求。

4.4 遵守认可规则、认可政策的有关规定，同意履行相关义务。

5 认可流程

5.1 初次认可

5.1.1 意向申请

申请人可以用任何方式向CNAS秘书处表示认可意向，如来访、电话、传真以及其他电子通讯方式。CNAS秘书处应向申请人提供最新版本的认可规则和有关文件。

5.1.2 正式申请

5.1.2.1 申请人应按CNAS秘书处的要求提供申请资料，并交纳申请费用。

5.1.2.2 申请资料提交CNAS-AL05《实验室生物安全认可申请书》，并根据其要求提交相关附件。

5.1.2.3 申请资料提交的文件份数按照CNAS-AL05《实验室生物安全认可申请书》规定，同时提供电子版本。

5.1.2.4 申请人应对申请材料的真实有效性负责。

5.1.3 受理

5.1.3.1 CNAS秘书处审查申请人提交的申请资料，做出是否受理的决定并通知申请人。

5.1.3.2 CNAS秘书处应在收到申请资料后审查资料的完整性。若申请人提交的资料齐全、符合要求，秘书处予以正式受理；若申请人提交的资料不全，秘书处应以邮件、电话等方式通知申请人补充相应文件，符合要求后，秘书处予以正式受理。

5.1.4 评审准备

5.1.4.1 CNAS秘书处应在受理后组成评审组，评审组由具备资格的评审员和相应的技术专家组成，组长负责制定评审计划。

5.1.4.2 CNAS秘书处指定评审组并征得申请人同意，如申请人基于公正性理由对评审组的任何成员有异议时，秘书处经核实后可给予调整。

5.1.4.3 评审组应对申请资料和实验室安全管理体系文件进行审查，当发现文件不符合认可准则的要求时，评审组长应以书面方式通知申请人采取纠正措施。

5.1.4.4 应申请人的申请或根据评审组长对申请资料审查的情况，经评审组长建议并征得申请人的同意，CNAS 秘书处可安排有关人员对申请人进行预访，进一步了解申请人的情况、解释认可要求，以便制定详细的评审计划。有关人员进行预访时，不得做咨询。

5.1.4.5 文件通过审查后，评审组长与申请人商定现场评审的具体时间安排和评审计划，报CNAS秘书处批准后实施。

5.1.4.6 需要时，CNAS可在评审组中委派观察员。

5.1.5 现场评审

5.1.5.1 现场评审包括文件审核和现场审核。

5.1.5.2 文件审核是指由评审组对申请人提供的申请资料和实验室安全管理体系文件与认可准则的符合性进行的进一步的审查与核实过程, 必要时,提出不符合项和观察项。

5.1.5.3 现场审核指由评审组对申请人实验室的布局、结构、设施设备等硬件系统以及实验室安全管理体系文件的运行情况,依据认可准则的要求进行符合性审核与验证,并进行必要的现场测试（例如，压力场、定向流，报警系统、应急系统等）的过程，必要时,提出不符合项和观察项。

5.1.5.4 综合文件审核和现场审核结果，现场评审结论包括“符合”、“基本符合”或“不符合”三种，由评审组在现场评审结束时给出；适用时，应同时提供不符合项或观察项报告。

5.1.5.5 评审组长应在现场评审末次会议上，将现场评审报告复印件提交给实验室。

5.1.5.6 对于评审中发现的不符合，实验室应及时采取纠正措施，纠正措施通常应在3个月内完成。评审组应对纠正措施的有效性进行验证。如需进行现场验证时，被评审实验室应予配合，支付评审费，并承担其他相关费用。

5.1.5.7 纠正措施验证完毕后，评审组长将最终评审报告和推荐意见报CNAS秘书处。

5.1.6 评定

5.1.6.1 CNAS 秘书处负责将评审报告及其推荐意见提交给评定委员会，评定委员会对申请人与认可要求的符合性进行评价并作出决定。评定结果可以是以下三种类型之一：

a)同意认可；

b)补充资料，再行评定；

c)不同意认可。

5.1.6.2 经评定后，由秘书处办理相关手续。

5.1.7 批准发证

5.1.7.1 CNAS秘书处向获准认可实验室颁发认可证书以及认可决定书，认可证书有效期为5 年。

5.1.7.2 CNAS秘书处负责公布获得认可实验室的有关认可信息。

5.2 监督评审

监督评审的目的是为了证实获得认可的实验室在证书有效期内持续符合要求，并保证在相关规则和要求修订后，及时将有关要求纳入其安全管理体系。监督评审包括定期和不定期的监督评审。所有获得认可的实验室均须接受监督评审。

5.2.1 定期监督评审

5.2.1.1获准认可的二级、三级生物安全实验室应在认可批准后的第12个月前、第30个月前、第48个月前接受定期监督评审。四级实验室监督评审应每12个月一次。三级、四级实验室监督评审应在实验室终末消毒后进行。

5.2.1.2 定期监督评审不需要申请。监督评审（包括监督+变更评审）中发现不符合时，实验室应及时采取纠正措施，纠正措施完成期限一般为2个月。

5.2.1.3 评审的方式包括现场审核、查阅与实验活动相关的档案和记录等，评审要求同初次认可评审。

5.2.2 不定期监督评审

5.2.2.1 在发生（但不限于）以下情况时，CNAS可视需要随时安排对实验室的不定期监督评审：

a) 获准认可实验室发生本规则6章所述变化；

b) CNAS秘书处认为需要对投诉或其他情况反映进行调查；

c) 获准认可实验室因违反认可要求曾被暂停认可资格；

d) CNAS秘书处认为有必要进行的专项检查。

5.2.2.2 不定期监督评审方式可以是现场评审，也可以是其他评审方式，如文件评审等。5.2.2.3当不定期监督评审中发现不符合时，被评审实验室在明确整改要求后应拟订并实施纠正措施，纠正措施完成期限与定期监督评审要求一致。。

5.3 复评审

5.3.1 实验室应在认可证书有效期满前6个月提出复评审申请，并按初次认可要求提交申请资料。 此外，还应提交前一认可有效期内的实验室使用报告、实验室变化状况报告（如平面布局变化、结构变化、设施设备变化、关键岗位人员变化、所操作的生物因子变化、重要操作程序变化等）和实验室安全事故报告等。

5.3.2 复评审程序同初次认可程序。复评审中发现不符合时，实验室应及时采取纠正措施，纠正措施完成期限一般为2个月。

6 变更

6.1 获得认可的实验室的变更

6.1.1 变更通知

在证书有效期内，发生下述任何变化时，应在20个工作日内以书面形式通知CNAS秘书处：

a) 实验室的名称、地址、法律地位发生变化；

b) 实验室的关键管理和技术人员、安全管理人员发生变化；

c) 实验室在同一危害程度分类（根据国家卫生和兽医主管部门发布的病原微生物名录）中的生物因子或实验活动发生变化；

d) 实验室的设施设备发生变化且可能影响生物安全防护能力时；

e）其他可能影响实验室活动和运行安全的变化。

6.1.2 变更的处理

CNAS 秘书处在得到变更通知并核实情况后，视变更性质可采取以下措施：

a) 对 6.1.1 中的 a)、b)、c)变更情况，实验室应向 CNAS 提交书面报告；

b) 对 6.1.1 中的 d)、e)变更情况，实验室应向 CNAS 提交变更申请，CNAS 应进行评审。

6.1.3 当实验室发生6.1.1 的变更，且未按要求通知CNAS，CNAS将视情况予以暂停或撤销认可。

6.1.4 当实验室的变更（如平面布局和位置变化等）导致实验活动不能正常开展，实验室除按6.1.1条规定通报CNAS秘书处外，还应立即停止实验活动，保留相关记录，待CNAS通过现场评审方式确认后，方可继续（恢复）在相应防护级别内的实验活动。

6.2 认可要求的变更

6.2.1 当相关认可要求发生变更时，CNAS 秘书处应及时通知可能受影响的实验室和有关申请人，详细说明所发生的变化。

6.2.2 CNAS 秘书处应公布转换的办法和期限。

6.2.3 获得认可的实验室在完成转换后，应及时通知CNAS秘书处。CNAS秘书处通过监督评审或复评审的方式对实验室与新要求的符合性进行确认，在确认符合要求后，继续维持认可；如实验室在规定的期限不能完成转换，CNAS可暂停或撤销认可。

7 暂停、恢复、撤销和注销认可

7.1 暂停认可

获准认可实验室不能持续地符合CNAS的认可条件和要求，CNAS应暂停认可，例如：

a）无故不接受定期监督；

b）不按时缴纳费用；

c）在监督评审和复评审过程中发现已获认可的实验室防护级别达不到要求或不能在规定的期限内完成纠正措施；

d）实验室的人员、设施、设备等发生重大变化，未按6.1.1条规定通报CNAS秘书处；

e）当认可规则、认可要求和认可准则发生变化，获准认可实验室不能按时完成转换；

f）实验室发生工作人员感染事故或者病原微生物泄漏事件的报告，或者发现实验室从事病原微生物相关实验活动造成实验室感染事故；

g）获准认可实验室存在其他违反认可规定的情况。暂停期不少于60天，但不超过1年。

7.2 恢复认可

被暂停认可的实验室，在规定的暂停期限内实施纠正措施并经CNAS确认符合认可要求后，可恢复认可。

7.3 撤销认可

在下列情况下，CNAS可以撤销认可：

a) 被暂停认可的已认可实验室超过暂停期仍不能恢复认可；

b) 由于认可规则或认可准则变更，已认可实验室不能或不愿继续满足认可要求；

c) 已认可实验室不能履行CNAS本规则规定的义务。

7.4 注销认可

在下列情况下，CNAS应予注销认可：

a) 已认可实验室终止从事实验室活动；

b) 已认可实验室自愿申请撤销认可或有效期满未申请继续认可。

8 权利和义务

8.1 CNAS 的权利和义务

8.1.1 CNAS 有权对实验室开展的活动和认可证书及认可标识的使用情况进行不定期监督。

8.1.2 CNAS 有权根据相关方的投诉对实验室进行现场调查和跟踪调查，并据以提出整改要求。

8.1.3 CNAS 有权针对实验室不符合 CNAS 规定的情况，作出暂停、恢复、撤销认可资格的决定。

8.1.4 CNAS 有义务利用网站公开获准认可实验室的认可状态信息(有保密要求的除外)并及时更新，信息包括:

a) 已认可实验室的名称和地址；

b) 认可的批准日期和终止日期。

8.1.5 CNAS 有义务在认可要求发生变化时及时通知已获准认可实验室，在对更改内容和生效日期作出决定之前，听取各有关方面的意见，以便获准认可的实验室在合理的期限内作出调整。

8.1.6 CNAS 有义务及时向申请/已获认可实验室提供最新版本的认可规则、准则和其它有关文件，有计划地对实验室进行有关的认可知识的宣贯和培训，并以积极态度，主动征询实验室的意见，注意随时收集认可工作中实验室的相关信息反馈，促进CNAS 认可体系的持续改进。

8.1.7 为了解实验室和潜在客户的需求，CNAS 有义务及时答复有关认可问询，建立行之有效的信息发布和客户反馈系统，通过组织宣传、培训活动，满足实验室需求。

8.1.8 除需要公开的信息外，CNAS 有义务对在实验室认可活动中获得或产生的其他信息，如商业、技术等信息保密。

8.2 实验室的权利和义务

8.2.1 申请认可实验室的权利和义务

8.2.1.1 实验室有权获得 CNAS 的相关公开文件。

8.2.1.2 实验室有权获得本实验室认可评审安排进度、评审组成员及所服务的单位等信息。

8.2.1.3 实验室有权对与认可有关的决定提出申诉，有权对 CNAS 工作人员及评审组成员的工作提出投诉。

8.2.1.4 在基于公正性原因时，实验室有权对评审组的组成提出异议。

8.2.1.5 实验室有义务了解 CNAS 的有关认可要求和规定。

8.2.1.6 实验室有义务按照 CNAS 的要求提供申请文件和相关信息，并保证内容真实、准确。

8.2.1.7 实验室有义务服从 CNAS 秘书处的各项评审安排，为评审活动提供必要的支持，并为有关人员进入被评审的区域、查阅记录、见证现场活动和接触工作人员等方面提供方便，不得拒绝 CNAS 秘书处派出的见证评审活动的人员（包括国际同行评审的见证人员）。

8.2.2 获准认可实验室的权利和义务

8.2.2.1 实验室有权在规定的范围内宣传其从事活动的生物安全防护级别已被认可。

8.2.2.2 实验室有权在其获认可范围内出具的证书或报告以及拟用的广告、专用信笺、宣传刊物上使用认可标识/联合标识。

8.2.2.3 实验室有权对 CNAS 工作人员、评审人员的工作提出投诉，并有权对 CNAS针对其作出的与认可有关的决定提出申诉。

8.2.2.4 实验室有权自愿终止认可资格。

8.2.2.5 实验室有义务确保其运作和提供的服务持续符合本规则第 4 条中规定的认可条件。

8.2.2.6 实验室有义务自觉遵守相关法律法规。

8.2.2.7 实验室有义务为 CNAS 秘书处安排评审活动提供必要的支持，并为有关人员进入被评审的区域、查阅记录、见证现场活动和接触工作人员等方面提供方便，并不得拒绝 CNAS 秘书处派出的见证评审活动的人员（包括国际同行评审的见证人员）。

8.2.2.8 实验室有义务建立客户投诉处理程序，如在收到投诉后 2 个月内未能使相关方满意，应将投诉的概要和处理经过等情况通知 CNAS 秘书处。

8.2.2.9 实验室在发生本规则 6.1.1 条所述变化时，有义务及时书面通知 CNAS 秘书处；有义务在认可要求发生变化时按照 CNAS 要求进行调整，并在调整完成后通知CNAS 秘书处。

8.2.2.10 实验室有义务做到公正诚实，不弄虚作假，不从事任何有损 CNAS 声誉的活动。

8.2.2.11 实验室有义务在其证书、报告或宣传媒介，如广告、宣传资料或其他场合中表明其认可状态时，符合 CNAS 的有关规定。

8.2.2.12 实验室有义务在被 CNAS 撤销认可或自愿注销认可资格时，或在认可证书（或认可决定书）明示认可的期限逾期时，立即交回认可证书，停止在证书、报告或宣传材料上使用认可标识，并不得采用任何方式表示其认可资格仍然有效。

8.2.2.13 实验室有义务经常浏览 CNAS 网站，及时获得认可状态、认可要求等相关信息。

8.2.2.14 实验室有义务按有关规定缴纳费用。

8.2.2.15 实验室有义务及时将认可资格的暂停、缩小、撤销及相关后果告知其受影响的客户，不得有不当延误。

1. 中华人民共和国公共安部：中华人民共和国公共安全行业标准 第一部分：高等级病原微生物实验室

本文件规定了高等级病原微生物实验室反恐怖防范的重点目标和重点部位、总体防范要求、常态防范要求、非常态防范要求和安全防范系统技术要求。

本文件适用于高等级病原微生物实验室的反恐怖防范工作与管理

2 规范性引用文件

下列文件中的内容通过文中的规范性引用而构成本文件必不可少的条款。其中,注日期的引用文件,仅该日期对应的版本适用于本文件;不注日期的引用文件,其最新版本(包括所有的修改单)适用于本文件。

GB12899手持式金属探测器通用技术规范

GB15208.1 微剂量X射线安全检查设备第1部分:通用技术要求

GB17565-2022防盗安全门通用技术条件

GB/T22239信息安全技术网络安全等级保护基本要求

GB/T 28181公共安全视频监控联网系统信息传输、交换、控制技术要求

GB/T32581-2016侵和紧急报警系统技术要求

GB35114公共安全视频监控联网信息安全技术要求

GB/T37078-2018出人口控制系统技术要求

公共安全重点区域视频图像信息采集规范GB 37300

GB50348安全防范工程技术标准

GB55029安全防范工程通用规范

GA69防爆毯

GA/T644电子巡查系统技术要求

GA844防砸透明材料

WS233病原微生物实验室生物安全通用准则

3 术语和定义

GB50348和WS233界定的以及下列术语和定义适用于本文件。

3.1

病原微生物实验室pathogenic microorganismlaboratory

从事能够使人或者动物致病的微生物菌(毒)种、样本有关的研究、教学、检测、诊断等活动的实验室。

3.2

高等级病原微生物实验室high-levelpathogenic microorganismlaboratory

生物安全防护水平为三级和四级的病原微生物实验室。

3.3

核心工作间coreroom病原微生物实验室中开展实验活动的主要区域,通常是指进行病原微生物操作、感染动物饲养或实验活动的房间。

[来源:WS 233-2017,2.11,有修改]

3.4设备机房equipmentroom

维持高等级病原微生物实验室正常功能配套的通风空调、生命支持、活毒废水处理、电力供应、动力等系统的核心设备所在的区域或空间。

3.5安全防范security

综合运用人力防范、实体防范、电子防范等多种手段,预防、延迟、阻止治安和暴恐事件(包括入侵盗窃、抢劫、破坏、爆炸、暴力袭击等)发生的活动。

来源:GB50348-2018,2.0.1,有修改7

3.6

人力防范personnel protection

具有相应素质的人员有组织的防范、处置等安全管理行为。

[来源:GB50348-2018,2.0.2,有修改]

3.7

实体防范physicalprotection

利用建(构)筑物、屏障、器具、设备或其组合,延迟或阻止风险事件发生的实体防护手段。[来源:GB50348-2018,2.0.3,有修改]

3.8

电子防范electronic security

利用传感、通信、计算机、信息处理及其控制、生物特征识别等技术,提高探测、延迟、反应能力的防护手段。

[来源:GB 50348-2018,2.0.4,有修改]

3.9

安全防范系统securitysystem

以安全为目的,综合运用实体防护、电子防护等技术构成的防范系统。

[来源:GB 50348-2018,2.0.57

3.10

常态防范regular protection

运用人力防范、实体防范、电子防范等多种手段和措施,常规性预防、延迟、阻止发生恐怖案事件的管理行为。

3.11

非常态防范unusualprotection

在重要会议、重大活动等重要时段以及获得涉恐怖袭击等预警信息或发生上述案事件时,相关单位临时性加强防范手段和措施,提升反忍怖防范能力的管理行为。

4 重点目标和重点部位

4.1 重点目标

高等级病原微生物实验室(以下简称实验室)为反恐怖防范的重点目标。

4.2 重点部位

下列部位为实验室反恐怖防范的重点部位:

a)实验室设立单位和(或)实验室所在建筑物的周界;

b)实验室设立单位和(或)实验室所在建筑物的周界出人口;

c)实验室所在建筑物出入口;

d)门卫值班室;

e) 通往实验室的电梯、电梯厅、通道(楼道、楼梯);

f)实验室设立单位的安防监控中心(室);

g)实验室出人口;

h) 核心工作间;

i)保存菌(毒)种及样本的房间;

i)中控室;

k)设备机房;

1)其他经风险评估应防范的部位。

5 总体防范要求

5.1 实验室设立单位新建、改建、扩建实验室的安全防范系统应与主体工程同步规划、同步设计、同步建设、同步验收、同步运行。已建、在建的实验室应按本文件要求补充完善安全防范系统。5.2 实验室设立单位应针对重点部位定期开展风险评估工作,综合运用人力防范、实体防范、电子防范等手段,按常态防范与非常态防范的不同要求,落实各项安全防范措施。5.3 实验室设立单位应建立健全反恐怖防范管理档案和台账,包括重点目标的名称、地址或位置、实验室平面布局图、设立单位及实验室负责人、保卫部门负责人,及现有的人力防范、实体防范、电子防范措施等。

5.4 实验室设立单位应根据公安机关和有关部门的要求报告防范措施落实情况,并提供实验室的相关信息和重要动态,接受安全保卫工作监督指导。

5.5 实验室设立单位应对实验室工作人员及其安全保卫人员进行安全背景审查。进入实验室的人员应当经实验室负责人批准。

5.6 实验室设立单位应设立反恐怖防范专项资金,保障反恐怖防范工作机制运转正常。5.7 实验室设立单位应建立安全防范系统运行与维护的保障体系和长效机制,定期对系统进行维护，及时排除故障,保持系统处于良好的运行状态。

5.8实验室设立单位应制定反恐怖突发事件应急预案和现场处置方案,并组织开展相关培训和定期

演练。

5.9实验室设立单位应与属地公安机关等政府有关部门建立联防、联动、联治工作机制5.10 实验室设立单位应建立反恐怖与实验室运行管理等有关信息的共享和联动机制。

5.11实验室的网络与信息系统应明确安全保护等级,采取GB/T22239中相应的安全保护等级的防护措施。

5.12 实验室安全防范系统中涉及公民个人信息的,应依法依规进行处理,包括收集、存储、使用、加工、传输、提供、公开、删除等。

5.13非常态防范要求应在常态防范要求的基础上执行。

5.14 实验室常态防范设施配置应符合附录A的要求。

6 常态防范要求

6.1 人力防范要求

6.1.1 实验室设立单位应设置与安全保卫任务相适应的反恐怖工作保卫部门,配备专职保卫管理人员,建立健全值守、巡逻、培训、检查、考核、安全防范系统运行与维护等制度。

6.1.2 实验室设立单位应配备专职保卫执勤人员,并对承担实验室安全保卫工作的机构及人员资质进行审查。

6.1.3 实验室设立单位应对外来人员及车辆进行登记备案。

6.1.4应在实验室所在院落或所在建筑物出入口设置门卫值班室,实行24h值班制,每班人数应不少于2人。

6.1.5 安防监控中心(室)值班人员应24h值守,每班人数应不少于2人。6.1.6 保卫执勤人员应对重点部位[除4.2中g)、h)、i)、j)、k)外]进行日常巡逻,巡逻周期间隔应不大于4 h。

6.1.7 保卫执勤人员应配备棍棒、钢叉、盾牌、头盔、防刺背心等防卫防护装备器材,及对讲机等必要的通信工具。

6.1.8 门卫值班室应配置符合GA69要求的防爆等处置设备:

6.1.9 实验室设立单位应每半年至少组织一次反恐怖教育培训.

6.1.10实验室设立单位应每半年至少组织一次反恐怖应急预案演练。6.1.11 在实验室开展工作时,应有2名及以上的工作人员共同进行,实验室中控室应设置能熟练操作安全防范相关设备和软件的工作人员。

6.2 实体防范要求

6.2.1 实验室设立单位和(或)实验室所在建筑物具有独立院落的,应在实验室所在院蒸周界设置实体围墙或栅栏等实体屏障,实体屏障外侧整体高度(含防攀爬设施)应不小于2.5m,且周界出人口应设置车辆阻挡装置，采用电动操作的车辆阻挡装置,应具有手动应急操作功能。6.2.2生物安全四级实验室建筑距相邻建筑物或构筑物的距离应不小于相邻建筑物或构筑物相对高度的1.5倍,距最近的非本单位建筑物或构筑物距离应不小于80m。6.2.3 核心工作间与外界相通的窗户应有防外部窥视的措施,窗户应为密闭窗,玻璃应采用符合GA844要求的防砸透明材料。

6.2.4 实验室设立单位独立设置的安防监控中心(室)中控室应设置防盗安全门,其防盗安全级别应不低于GB17565-2022规定的3级。

6.3 电子防范要求

6.3.1 实验室设立单位和(或)实验室所在建筑物具有独立院落的,应在实验室所在院落周界设置视频图像采集装置,视频监视和回放图像应能清晰显示周界区域人员活动情况;其周界出入口应设置视频图像采集装置和出入口控制装置,视频监视和回放图像应能清晰显示进出人员的体貌特征和进出车辆的号牌,出人口控制装置应对进出人员及车辆进行权限识别和出人控制。

6.3.2 实验室设立单位和(或)实验室所在建筑物没有独立院落的,实验室所在建筑物周界应设置视频图像采集装置,视频监视和回放图像应能清晰显示周界区域人员活动情况。

6.3.3 生物安全四级实验室所在院落周界应设置人侵探测装置,探测范围应能对周界实现全覆盖。

6.3.4 实验室所在建筑物出入口应设置视频图像采集装置和出入口控制装置,视频监视和回放图像应能清晰显示进出人员的体貌特征,出入口控制装置应对进出人员进行权限识别和出人控制。

6.3.5 生物安全四级实验室所在建筑物出入口应配备符合GB12899要求的手持式金属探测器和符合GB15208.1要求的微剂量X射线安全检查设备等安全检查设备,对进出人员及携带物品进行安全检查。

6.3.6 通往实验室的电梯、电梯厅、通道(楼道、楼梯)应设置视频图像采集装置,视频监视和回放图像应能清晰显示电梯内、电梯厅及通道区域的人员活动情况。

6.3.7 实验室出入口应设置视频图像采集装置和出入口控制装置,视频监视和回放图像应能清晰显示进出人员的体貌特征,出人口控制装置应对进出人员进行权限识别和出入控制。

6.3.8核心工作间的出入口及内部应设置视频图像采集装置,视频监视和回放图像应能清晰显示人员出人及活动情况。

6.3.9 保存菌(毒)种及样本的房间出人口及内部应设置视频图像采集装置,视频监视和回放图像应能清晰显示人员出入及活动情况。保存菌(毒)种及样本的房间如在防护区内,可与实验室共用出入口控制装置。

6.3.10中控室的出人口应设置视频图像采集装置和出人口控制装置,视频监视和回放图像应能清晰显示进出人员的体貌特征,出入口控制装置应对进出人员进行权限识别和出入控制;其内部应设置通信装置和视频图像采集装置,视频监视和回放图像应能清晰显示中控室内的人员活动情况。中控室的通信装置应能保证与实验室设立单位的安防监控中心(室)间信息畅通。

6.3.11 设备机房的出入口应设置视频图像采集装置和出入口控制装置,视频监视和回放图像应能清晰显示进出人员的体貌特征,出人口控制装置应对进出人员进行权限识别和出入控制;其内部应设置视频图像采集装置,视频监视和回放图像应能清晰显示设备机房内的人员活动情况。

6.3.12安防监控中心(室)的出人口应设置视频图像采集装置和出入口控制装置,视频监视和回放图像应能清晰显示进出人员的体貌特征,出人口控制装置应对进出人员进行权限识别和出入控制;其内部应设置视频图像采集装置,视频监视和回放图像应能清晰显示室内人员活动情况。

6.3.13 安防监控中心(室)应设置紧急报警装置。

6.3.14重点部位[除4.2中g)h)i)j)k)外]应设置电子巡查装置。

7 非常态防范要求

7.1 人力防范要求

7.1.1 实验室设立单位应启动应急响应机制,组织开展反恐怖动员;实验室设立单位负责人或其授权人员应24h带班组织防范工作,在常态防范基础上加强保卫力量。

7.1.2 保卫执勤人员对重点部位[除4.2中g)、h)、i)、j)、k)外]的巡逻周期间隔应不大于2h。

7.1.3 实验室设立单位和(或)实验室所在院落周界出入口应设置警戒区域,对人员、车辆实行进入许可管控。

7.1.4 应加强对出人实验室的人员及所携带物品的安全检查,对外来人员携带物品进行开包检查。

7.2 实体防范要求

7.2.1 应加强防护器具、救援器材、应急物资以及重点部位的门、窗、锁、车辆阻挡装置等设施的有效性检查。

7.2.2 应减少周界出人口的开放数量。

7.2.3 周界出入口的车辆阻挡装置应设置为阻截状态。

7.3 电子防范要求

7.3.1 应加强电子防范设施、通信设备的检查和维护,确保安全防范系统正常运行及通信设备的正常使用。

7.3.2应提高出入口控制系统对各受控区的出人识别权限与核验规则配置,强化出入口控制系统对各区域的人员管理。

8 安全防范系统技术要求

8.1 一般要求

8.1.1 安全防范系统的设备和材料应符合相关标准并检验合格。

8.1.2安全防范管理平台应对系统内具有计时功能的设备进行校时,设备的时钟与北京时间误差应不

大于5 s。

8.1.3安全防范系统和设备登录密码不应为弱口令,不应存在网络安全漏洞和隐患。当基于不同传输网络的系统和设备联网时,应采取相应的网络边界安全管理措施。8.1.4 安全防范系统的各子系统应符合GB55029的相关规定。

8.2 入侵和紧急报警系统

8.2.1 系统应能探测防范区域内的人侵行为。系统报警后,安防监控中心(室)应能有声、光指示,并能准确指示发出报警的位置。

8.2.2 系统应具备防拆、开路、短路报警功能。

8.2.3系统应具备故障报警和断电报警功能。

8.2.4 系统应与视频监控系统联动。

8.2.5系统布防、撤防、故障和报警信息存储时间应不少于180d。

8.2.6 系统应有备用电源,应能保证系统正常工作时间不小于8h。

8.2.7 系统的安全等级应不低于GB/T32581-2016规定的2级要求。

8.3 视频监控系统

8.3.1 系统监视和回放图像的水平像素数应不小于1920,垂直像素数应不小于1080,图像帧率应不小于 25 fps。

8.3.2 系统应与人侵和紧急报警系统联动。

8.3.3 视频图像信息应实时记录,存储时间应不少于90d。

8.3.4 涉及公共区域的视频图像信息的采集要求应符合GB37300的相关规定8.3.5 系统应有备用电源,应能保证系统关键设备的应急供电时间不小于1h。

8.4 出入口控制系统

8.4.1 系统应能对强行破坏,非法进人的行为发出报警信号,报警信号应与相关出人口的视频图像联动。

8.4.2 系统应满足紧急逃生时人员疏散的相关要求。

8.4.3系统信息存储时间应不少于180d。

8.4.4 断电开启的出人口控制点应配置备用电源,应能保证执行装置正常工作时间不小于 48 h。

8.4.5系统的安全等级应不低于GB/T37078-2018规定的2级要求。

8.5 电子巡查系统

8.5.1 巡查路线、巡查时间应能根据安全管理需要进行设定和修改。

8.5.2 巡查记录保存时间应不少于180 d。

8.6 集成联网

8.6.1 实验室设立单位安防监控中心(室)的安全防范管理平台应实现对除实验室出入口、核心工作间、保存菌(毒)种及样本的房间、中控室以外区域的入侵和紧急报警、视频监控、出入口控制、电子巡查等各安全防范子系统的集成与管理。

8.6.2 安全防范管理平台应具有系统集成、联动控制、权限管理、存储管理、检索与回放、设备管理、统计分析、系统校时、指挥调度等功能。

8.6.3 安全防范管理平台的故障不应影响各子系统的正常运行,某一子系统的故障不应影响其他子系

统的正常运行。

8.6.4 视频监控系统应留有与公共安全视频图像信息共享交换平台联网的接口,联网信息传输、交换、控制协议应符合GB/T28181的相关规定,联网信息安全应符合GB35114的相关规定。

1. 中华人民共和国国家质量监督检验检疫总局、中国国家标准化管理委员会：移动式实验室生物安全要求

国家标准GB 27421-2015

1 范围

本标准规定了对一级、二级和三级生物安全防护水平移动式实验室的设施、设备和安全管理的基本要求,不包括对移动式生物安全四级实验室和开放或半开放饲养动物的生物安全三级实验室的要求。

第6章以及7.1和7.2是对移动式实验室生物安全防护设施和设备的基础要求,需要时,适用于更高防护水平的移动式实验室。

针对与感染动物饲养相关的实验室活动,本标准规定了对移动式实验室内动物饲养设施和环境的基本要求。需要时,7.3适用于相应防护水平的动物生物安全移动式实验室。

本标准适用于涉及生物因子操作的移动式实验室。

2 规范性引用文件

下列文件对于本文件的应用是必不可少的。凡是注日期的引用文件,仅注日期的版本适用于本文件。凡是不注日期的引用文件,其最新版本(包括所有的修改单)适用于本文件。

GB14925—2010 实验动物 环境及设施

GB19489—2008 实验室 生物安全通用要求

3 术语和定义

下列术语和定义适用于本文件。

3.1

移动式实验室 mobilelaboratory

可变换地点使用的实验室。

3.2

定向气流 directionalairflow

流向受控制的气流。

3.3

一级防护屏障 primarybarrier

操作者和被操作对象之间的物理屏障或隔离。

注:个体防护装备也视为一级屏障。在本标准中未说明时,一级屏障指生物安全柜或隔离器等防护设备。

3.4

二级防护屏障 secondarybarrier

公共环境和被操作对象之间的物理屏障或隔离。

注:实验室的围护结构属二级防护屏障。

4 移动式实验室风险评估及风险控制

4.1 应满足GB19489—2008第3章适用的要求。

4.2 应评估实验室移动方式和移动过程中的风险,并采取适当的控制措施。

4.3 应评估环境对移动式实验室的风险(不限于生物风险),并采取适当的控制措施。

4.4 应评估移动式实验室和实验活动对环境的风险(不限于生物风险),并采取适当的控制措施。

4.5 在维护、维修、改造实验室前后或其退役前,应进行风险评估,并采取适当的控制措施。

4.6 应依据国家相关主管部门发布的病原微生物分类名录,在风险评估的基础上,确定实验室从事的活动。

5 移动式实验室的基本技术形式和安全防护水平分级

5.1 根据移动式实验室的移动模式分为自行式和运载式实验室。自行式实验室应具备机动行驶功能;运载式实验室应可借助运载工具实现移动功能。

5.2 根据移动式实验室的一级防护屏障模式分为开放式、二级生物安全柜式和三级生物安全柜式实验室。开放式实验室不使用生物安全柜或等效装置;二级生物安全柜式实验室应配备等效于二级生物安全柜的一级防护屏障;三级生物安全柜式应配备等效于三级生物安全柜的一级防护屏障。

5.3 根据移动式实验室的二级防护屏障模式分为自然通风式和负压通风式实验室。自然通风式实验室可设置通风窗或换气扇,不控制室内气压。负压通风式实验室应采用机械通风,应将室内气压控制为负压(相对于室外气压)。

5.4 GB19489—2008第4章对实验室生物安全防护水平的分级原则适用于对移动式实验室生物安全防护水平的分级:以BSL-1、BSL-2或BSL-3(bio-safetylevel,BSL)表示仅从事体外操作的实验室的相应生物安全防护水平(一级、二级或三级);以 ABSL-1、ABSL-2或 ABSL-3(animalbio-safetylevel,AB-SL)表示包括从事动物活体操作的实验室的相应生物安全防护水平(一级、二级或三级)。

6 移动式实验室设计原则及基本要求

6.1 设计原则

6.1.1 设计宗旨应以变换地点使用为目的,符合移动性的技术和材料要求,具备实验室的基本功能。

6.1.2 应适用于快速反应行动,可按要求自行或被运达指定地点,并开展符合相应生物安全防护级别要求的实验活动。

6.1.3 应安全、可靠、耐用、易用,符合生物安全防护要求,满足职业卫生要求、环境保护要求和节能要求。

6.1.4 如果适用,应满足GB19489—2008的相应要求。

6.1.5 涉及实验动物时,如果适用,应满足GB14925—2010的相应要求。

6.1.6 如果无特殊需求,应符合国家相关规定或标准对机动行驶装置或被运输装置的设计要求和制造要求。

6.1.7 按照客户特定要求设计制造的移动式实验室,如果技术指标与国家相关规定或标准的要求冲突,应事先征询相关主管部门的建议。

6.1.8 应安装行车定位系统和行车记录系统。

6.2 基本要求

6.2.1 应易于自行到达或被运达指定地点。

6.2.2 需运载部分应具有适宜的装卸、搬运和固定装置,满足搬运和运输工具的要求。宜设升降装置及支撑轮,以便实现短距离移动。

6.2.3 实验室和可拆卸部分的最大外廓尺寸宜参照国家对可移动设施的相关标准设计和制造。

6.2.4 应按模块化、集成化和标准化的原则和要求进行设计和选型,以保证通用性和易维护性。

6.2.5 如果适用,应选用免维护器材。

6.2.6 设施设备的布局、作业空间、设备操作方式等应合理,以保证工作流程顺畅并符合人机工效学的原则和要求。

6.2.7 应保证所有维护工作的可实施性,作业工位空间应适合人体量度、姿势及使用工具等的需求。如果选装机电设备,应不影响维修工作。

6.2.8 宜考虑实验室的扩展性能,以易于和其他独立的设施组合连接,提高应用性。

6.2.9 水、电、气、暖、行驶等各系统应满足实验室运行的要求和相关的安全性要求,同时考虑移动式实验室的特殊要求。水、电、气等也可由外部来源输入。

6.2.10 应有保证实验室内设施设备可靠固定的设计和措施。

6.2.11 应保证所用设备进出顺利。如果有安全(逃生)门(窗),适用时,可兼作设备门。

6.2.12 应保证消防、防电击、防雷击、抗振动与冲击、电磁兼容等的设计符合相关要求。

6.2.13 实验室布局应方便人员紧急出入,出入路径复杂的实验间应设置独立的安全(逃生)门(窗)。

6.2.14 实验室的可靠性应适应移动需求和环境变化。

6.2.15 应根据实验室拟工作地区,设定其对道路和自然条件等适应性的要求且不应低于国家相关标准的规定,包括(不限于)以下因素:

a) 道路和地面;

b) 温度;

c) 湿度;

d) 气压;

e) 风力;

f) 日晒;

g) 雷电;

h) 冰雪;

i) 雨雹;

j) 沙尘;

k) 烟雾(包括盐雾);

l) 有害生物(如:真菌、节肢动物、啮齿动物等)。

6.2.16 应配备满足现场使用、维护及维修需要的原理图、操作说明、维修手册和安全手册等文件。

6.2.17 自行式实验室应配备机动行驶部分的相关文件,符合6.2.16的要求。

6.2.18 适用时,应配备移动式实验室良好操作规范、现场应急处置预案等文件。

7 移动式实验室设施和设备要求

7.1 BSL-1实验室

7.1.1 实验室可由单个实验间组成。一级防护屏障模式可以为开放式,但应以风险评估为依据,包括对实验质量控制要求的内容。

7.1.2 实验室固定设备、台柜、壁柜应坚固并与舱体可靠连接,连接处应圆滑,便于清洁。

7.1.3 在实验室移动时,应有可靠机制和措施固定仪器设备、实验器材和座椅等物品。

7.1.4 实验室的高度应满足设备安装要求,应有维护和清洁空间。

7.1.5 实验室应通风。如采用自然通风,可设置可开启的窗户和/或换气扇,可开启的窗户和/或换气扇的进风口应安装可防蚊虫的纱窗。

7.1.6 如果采用机械通风,可采用带循环风的空调系统。应根据实验室使用地域及气候条件,合理设计实验室空调系统。

7.1.7 实验室宜预留市政供水接口,可设置下水收集装置。如下水外排,应以风险评估为依据。

7.1.8 若操作刺激或腐蚀性物质,应在实验室内设洗眼装置或配备洗眼瓶。若大量使用刺激或腐蚀性物质,应设置紧急喷淋装置。

7.1.9 实验室内应安装紫外线消毒灯并配备便携的消毒灭菌装置(如:消毒喷雾器等)。

7.1.10 需要时,应配备高压蒸汽灭菌器或其他适当消毒灭菌设备。

7.1.11 实验室工作区域的平均照度应不低于300lx。

7.1.12 应有机制保持通讯联络畅通。

7.2 BSL-2实验室

7.2.1 适用时,应符合7.1的要求。

7.2.2 核心实验间入口宜设置缓冲间,缓冲间可兼作防护服更换间。

7.2.3 缓冲间的门宜能互锁。如果使用互锁门,应在互锁门的附近设置紧急手动解除互锁开关,需要时,应可立即解除实验室门的互锁。

7.2.4 实验期间,核心实验间入口处的显著位置应有国际通用的生物危害警告标识和相关信息。

7.2.5 实验室可采用自然通风或负压通风。如果采用负压通风式空调系统,应符合定向气流原则。

7.2.6 采用负压通风式空调系统的新风口和排风口应有防风、防雨、防鼠、防虫设计,应根据风险评估的结果确定空气过滤器的规格。新风口应高于室外地面2.5m(可采用可拆卸结构),新风口设置尽量远离排风口。

7.2.7 核心实验间内应配备生物安全柜或其他生物安全隔离装置。

7.2.8 如果生物安全柜或其他生物安全隔离装置的排风在室内循环,实验室应具备通风换气条件。

7.2.9 实验室应配备适宜的消毒灭菌装置,需要时,应配备高压蒸汽灭菌器。

7.2.10 在负压通风式实验室核心实验间入口的显著位置,应安装显示房间负压状况的压力显示装置。

7.2.11 负压通风式实验室应有机制保持压力及压力梯度的稳定性,并可对异常情况报警。

7.2.12 负压通风式实验室的排风应与送风连锁,排风先于送风开启,后于送风关闭。

7.2.13 负压通风式实验室应有机制防止产生对人员有害的异常压力,围护结构应能承受送风机或排风机异常时导致的空气压力载荷。

7.3 BSL-3实验室

7.3.1 实验室应明确区分辅助工作区和防护区。

7.3.2 实验室主入口应有出入控制。实验室主入口处的生物危害警告标识和相关信息可采用可移动标牌,如磁性贴牌等。

7.3.3 辅助工作区应具备监控、技术保障(水、电、气、通风等)、清洁衣物更换、淋浴等功能,空间可共用。

7.3.4 缓冲间可不设置机械送排风系统。

7.3.5 实验室防护区内所有的门应可自动关闭,门应设密闭式观察窗,玻璃应耐撞击、防破碎。

7.3.6 有负压控制的区域相邻门应互锁,应在互锁门的附近设置紧急手动解除互锁开关,中控系统应具有解除所有门或指定门的互锁的功能。

7.3.7 淋浴间应有淋浴水收集装置,设防回流的装置,所收集污水应在风险评估的基础上有效处理。

7.3.8 应在实验室核心工作间内靠近出口处设置非手动洗手装置或自动手消毒装置。

7.3.9 实验室核心工作间宜设置活毒废水收集与灭活装置。

7.3.10 二级生物安全柜式实验室核心工作间的排风高效空气过滤器(或称 HEPA过滤器)应具备在原位进行消毒和检漏的条件。

7.3.11 空调系统的设计应考虑使用地域自然环境条件的适应性和各种设备的热湿负荷,送风和排风系统的设计应考虑所用生物安全柜、生物隔离器等通风设备的送排风量。

7.3.12 风口、门、设备应合理布局,以避免干扰和减少房间内的涡流和气流死角。

7.3.13 实验室的送风应经过 HEPA过滤器过滤,应同时安装初效和中效过滤器。

7.3.14 实验室应设置市政供水接口和储水箱,实验室给水与储水箱之间应设防回流装置。

7.3.15 如果有供气瓶或储水罐等,应放在实验室防护区外易更换和维护的位置,安装牢固。

7.3.16 如果实验操作需要真空装置,真空装置应安装在核心工作间内,真空装置排气应安装高效过滤装置。

7.3.17 应具备对实验室防护区及与其直接相通的通风管道、实验室设备和安全隔离装置(包括与其直接相通的管道)进行消毒灭菌的条件。

7.3.18 实验室应配备发电机自主供电,保证可靠、足够的电力供应,功率和燃料容量设计应有冗余,并设有外接电源输入接口。

7.3.19 应在辅助工作区设置专用配电箱和接地保护,实验室内应设置足够数量的固定电源插座,重要电源插座回路应单独回路配电,且应设置漏电检测报警装置。

7.3.20 灯具、开关、插座等所有在壁板、顶板需要安装的电气元件,其结构及安装应符合所在区域的密闭性要求,电气设备和接线应安装牢固。

7.3.21 应在实验室的关键部位(含室外)设置视频信号采集器,需要时,应实时监视并录制实验室活动情况和实验室周围情况。视频信号采集器应有足够的分辨率,影像存储介质应有足够的数据存储容量。 7.3.22 生物安全柜或其他生物安全隔离装置、送风机和排风机、照明、自控系统、监视和报警系统等应优先配备不间断备用电源,电力供应至少维持15min。如果不具备条件,应依据风险评估采取其他适宜的防护措施。

7.3.23 二级生物安全柜式实验室的核心工作间气压(负压)与室外大气压的压差值应不小于45Pa,与相邻区域的压差(负压)应不小于15Pa。

7.3.24 三级生物安全柜式实验室的核心工作间气压(负压)与室外大气压的压差值应不小于30Pa,与相邻区域的压差(负压)应不小于15Pa。

7.3.25 实验室防护区核心工作间的最小换气次数应不小于12次/h。

7.4 ABSL-1实验室

7.4.1 适用时,应符合7.1的要求。

7.4.2 应通过缓冲间或双门进入动物饲养间。

7.4.3 应设置实验动物饲养笼具,除考虑安全要求外还应考虑对动物质量和福利的要求。

7.4.4 动物饲养笼具排出的空气应通过管道排出室外。

7.4.5 适用时,动物饲养间的环境和设施条件应满足GB14925—2010的相关要求。

7.5 ABSL-2实验室

7.5.1 适用时,应符合7.2和7.4的要求。

7.5.2 应在安全隔离装置内饲养动物和从事可能产生有害气溶胶的活动;安全隔离装置的排气应经HEPA过滤器的过滤后排出。

7.6 ABSL-3实验室

7.6.1 适用时,应符合7.5的要求。

7.6.2 应使用Ⅲ级隔离器或等效设备饲养动物、转移和操作动物。可以使用Ⅱ级生物安全柜操作死亡动物。

7.6.3 应根据对实验活动风险评估的结果,确定淋浴间设置在防护区或辅助区。

7.6.4 缓冲间可不设置机械送排风系统。

7.6.5 动物饲养间气压(负压)与室外大气压的压差值应不小于45Pa,与相邻区域的压差(负压)应不小于15Pa。

7.7 对从事无脊椎动物操作实验室设施的要求

7.7.1 应满足GB19489—2008中6.5.5的适用要求。

7.7.2 应根据风险评估的结果确定是否需要其他措施。

8 管理要求

8.1 GB19489—2008第7章的相关要求适用于本标准。

8.2 移动式实验室不同于固定实验室。如果将移动式实验室作为固定实验室使用,应符合国家相关法规和标准对该类固定设施的要求。

8.3 每次移动实验室时,应有计划。需要时,应向相关管理部门备案或申请批准。应详细记录行车路线、驻留地点及时间,并建立工作日志。生物安全三级实验室的行动计划与现场工作方案参见附录B。 8.4 应指定现场工作负责人、安全负责人、技术负责人和工作团队,团队的规模和能力满足任务要求,应至少包括一名维护工程师。所有人员应经过相应级别实验室使用、维护和管理的相关培训,个人素质和能力胜任现场工作要求。

8.5 现场工作负责人应负责制定并向实验室或更高管理层提交活动计划、风险评估报告、安全及应急措施、人员培训及健康监督计划、技术支援方案、安全保障及资源要求、移动申请等。

8.6 应制定并维护包括移动过程的现场工作规程、安全手册和安保规定。

8.7 需要时,应在移动式实验室工作现场设立隔离带。

8.8 现场工作负责人应负责完成每次移动任务的总结报告,提交实验室或更高管理层,并归档保存。

8.9 实验室入口处的标识可以采用非固定的方式设置,如挂牌等。

8.10 应在移动实验室前、开始工作前进行安全检查(部分安全检查指南参见附录C),以保证:

a) 设施设备的功能和状态正常;

b) 警报系统的功能和状态正常;

c) 应急装备的功能及状态正常;

d) 消防装备的功能及状态正常;

e) 危险物品存放安全;

f) 废物处理装置数量和状态正常;

g) 所需备件(参见附录 D)和保障条件满足要求;

h) 人员能力及健康状态符合工作要求;

i) 不符合规定的工作已经得到纠正;

j) 所需资源满足工作要求。

8.11 在执行重大任务前,宜对关键要素和关键环节实施内部审核。

8.12 在现场执行任务周期超过180d时,宜在工作期间对关键要素和关键环节实施内部审核。

8.13 应提交现场工作总结报告作为管理评审材料。

8.14 应制定应急措施的政策和程序,包括生物性、化学性、物理性、放射性等紧急情况和火灾、水灾、风灾、冰冻、地震、人为破坏、倾覆等任何意外紧急情况,还应包括使留下的空实验室和辅助设施等处于尽可能安全状态的措施,应征询相关主管部门的意见和建议。应急预案编制大纲的编写指南参见附录E。 8.15 生物安全三级实验室应对高致病性病原微生物污染的废水废物消毒灭菌后移动。

8.16 如果实验室移动时需要携带可传染性物质(如样本等)、毒性物质等危险材料,应符合国家运输危险材料的相关规定。

8.17 在移动和工作期间发生的任何事件和事故应按国家规定及时上报。

8.18 应保证执行完任务的移动式实验室的内外部等所有部分符合卫生和生物安全要求,无不可接受的风险。

1. 中华人民共和国国家质量监督检验检疫总局、中国国家标准化管理委员会：实验室生物安全通用要求

国家标准GB 19489-2008

1． 范围

本标准规定了对不同生物安全防护级别实验室的设施、设备和安全管理的基本要求。

第5章以及6.1和6.2是对生物安全实验室的基础要求，需要时，适用于更高防护水平的生物安全实验室以及动物生物安全实验室。

针对与感染动物饲养相关的实验室活动，本标准规定了对实验室内动物饲养设施和环境的基本要求。需要时，6.3和6.4适用于相应防护水平的动物生物安全实验室。

本标准适用于涉及生物因子操作的实验室。

2． 术语和定义

下列术语和定义适用于本标准：

2.1 气溶胶 aerosols

悬浮于气体介质中的粒径一般为0.001 um--100um的固态或液态微小粒子形成的相对稳定的分散体系。

2.2 事故 accident

造成死亡、疾病、伤害、损坏以及其他损失的意外情况。

2.3 气锁 air lock

具备机械送排风系统、整体消毒灭菌条件、化学喷淋（适用时）和压力可监控的气密室，其门具有互锁功能，不能同时处于开启状态。

2.4 生物因子 biological agents

微生物和生物活性物质。

2.5 生物安全柜 biological safety cabinet，BSC

具备气流控制及高效空气过滤装置的操作柜．可有效降低实验过程中产生的有害气溶胶对操作者和环境的危害。

2.6 缓冲间 buffer room

设置在被污染概率不同的实验室区域间的密闭室，需要时，设置机械通风系统，其门具有互锁功能，不能同时处于开启状态。

2.7 定向气流 directional airflow

特指从污染概率小区域流向污染概率大区域的受控制的气流。

2.8 危险 hazard

可能导致死亡、伤害或疾病、财产损失、工作环境破坏或这些情况组合的根源或状态。

2.9 危险识别 hazard identification

识别存在的危险并确定其特性的过程。

2.10 高效空气过滤器（HEPA过滤器）high efficiency particulate air filter

通常以0.3um微粒为测试物，在规定的条件下滤除效率高于99.97%的空气过滤器。

2.11 事件 incident

导致或可能导致事故的情况。

2.12 实验室 laboratory

涉及生物因子操作的实验室。

2.13 实验室生物安全 laboratory biosafety

实验室的生物安全条件和状态不低于容许水平，可避免实验室人员、来访人员、社区及环境受到不可接受的损害，符合相关法规、标准等对实验室生物安全责任的要求。

2.14 实验室防护区 laboratory containment area

实验室的物理分区，该区域内生物风险相对较大，需对实验室的平面设计、围护结构的密闭性、气流，以及人员进入，个体防护等进行控制的区域。

2.15 材料安全数据单 material safety data sheet，MSDS

详细提供某材料的危险性和使用注意事项等信息的技术通报。

2.16 个体防护装备 personal protective equipment，PPE

防止人员个体受到生物性、化学性或物理性等危险因子伤害的器材和用品。

2.17 风险 risk

危险发生的概率及其后果严重性的综合。

2.18 风险评估 risk assessment

评估风险大小以及确定是否可接受的全过程。

2.19 风险控制 risk control

为降低风险而采取的综合措施。

3. 风险评估及风险控制

3.1 实验室应建立并维持风险评估和风险控制程序，以持续进行危险识别、风险评估和实施必要的控制措施。实验室需要考虑的内容包括：

3.1.1 当实验室活动涉及致病性生物因子时，实验室应进行生物风险评估。风险评估应考虑（但不限于）下列内容：

a) 生物因子已知或未知的特性，如生物因子的种类、来源、传染性、传播途径、易感性、潜伏期、剂量-效应（反应）关系、致病性（包括急性与远期效应）、变异性、在环境中的稳定性、与其他生物和环境的交互作用、相关实验数据、流行病学资料、预防和治疗方案等；

b) 适用时，实验室本身或相关实验室已发生的事故分析；

c) 实验室常规活动和非常规活动过程中的风险（不限于生物因素），包括所有进入工作场所的人员和可能涉及的人员（如：合同方人员）的活动；

d) 设施、设备等相关的风险；

e) 适用时，实验动物相关的风险；

f) 人员相关的风险，如身体状况、能力、可能影响工作的压为等；

g) 意外事件、事故带来的风险；

h) 被误用和恶意使用的风险；

i) 风险的范围、性质和时限性；

j) 危险发生的概率评估；

k) 可能产生的危害及后果分析；

1) 确定可接受的风险；

m) 适用时，消除、减少或控制风险的管理措施和技术措施，及采取措施后残余风险或新带来风险的评估；

n) 适用时，运行经验和所采取的风险控制措施的适应程度评估；

o) 适用时，应急措施及预期效果评估；

p) 适用时，为确定设施设备要求、识别培训需求、开展运行控制提供的输入信息；

q) 适用时，降低风险和控制危害所需资料、资源（包括外部资源）的评估；

r) 对风险、需求、资源、可行性、适用性等的综合评估

3.1.2 应事先对所有拟从事活动的风险进行评估，包括对化学、物理、辐射、电气、水灾、火灾、自然灾害等的风险进行评估。

3.1.3 风险评估应由具有经验的专业人员（不限于本机构内部的人员）进行。

3.1.4 应记录风险评估过程，风险评估报告应注明评估时间、编审人员和所依据的法规、标准、研究报告、权威资料、数据等。

3.1.5 应定期进行风险评估或对风险评估报告复审，评估的周期应根据实验室活动和风险特征而确定。

3.1.6 开展新的实验室活动或欲改变经评估过的实验室活动（包括相关的设施、设备、人员、活动范围、管理等），应事先或重新进行风险评估。

3.1.7 操作超常规量或从事特殊活动时，实验室应进行风险评估，以确定其生物安全防护要求，适用时，应经过相关主管部门的批准。

3.1.8 当发生事件、事故等时应重新进行风险评估。

3.1.9 当相关政策、法规、标准等发生改变时应重新进行风险评估。

3.1.10 采取风险控制措施时宜首先考虑消除危险源（如果可行），然后再考虑降低风险（降低潜在伤害友生的可能性或严重程度），最后考虑采用个体防护装备。

3.1.11 危险识别、风险评估和风险控制的过程不仅适用于实验室、设施设备的常规运行，而且适用于对实验室、设施设备进行清洁、维护或关停期间。

3.1.12 除考虑实验室自身活动的风险外，还应考虑外部人员活动、使用外部提供的物品或服务所带来的风险。

3.1.13 实验室应有机制监控其所要求的活动，以确保相关要求及时并有效地得以实施。

3.2 实验室风险评估和风险控制活动的复杂程度决定于实验室所存在危险的特性，适用时，实验室不一定需要复杂的风险评估和风险控制活动。

3.3 风险评估报告应是实验室采取风险控制措施、建立安全管理体系和制定安全操作规程的依据。

3.4 风险评估所依据的数据及拟采取的风险控制措施、安全操作规程等应以国家主管部门和世界卫生组织、世界动物卫生组织、国际标准化组织等机构或行业权威机构发布的指南、标准等为依据；任何新技术在使用前应经过充分验证，适用时，应得到相关主管部门的批准。

3.5 风险评估报告应得到实验室所在机构生物安全主管部门的批准；对未列入国家相关主管部门发布的病原微生物名录的生物因子的风险评估报告，适用时，应得到相关主管部门的批准。

4. 实验室生物安全防护水平分级

4.1 根据对所操作生物因子采取的防扩措施，将实验室生物安全防护水平分为一级、二级、三级和四级，一级防护水平最低，四级防护水平最高。依据国家相关规定：

a) 生物安全防护水平为一级的实验室适用于操作在通常情况下不会引起人类或者动物疾病的微生物；

b) 生物安全防护水平为二级的实验室适用于操作能够引起人类或者动物疾病，但一般情况下对人、动物或者环境不构成严重危害，传播风险有限，实验室感染后很少引起严重疾病，并且具备有效治疗和预防措施的微生物；

c) 生物安全防护水平为三级的实验室适用于操作能够引起人类或者动物严重疾病，比较容易直接或者间接在人与人、动物与人、动物与动物间传播的微生物；

d) 生物安全防护水平为四级的实验室适用于操作能够引起人类或者动物非常严重疾病的微生物，以及我国尚未发现或者已经宣布消灭的微生物。

4.2 以BSL-1、BSL-2、BSL-3、BSL-4 (bio-safety level，BSL)表示仅从事体外操作的实验室的相应生物安全防护水平。

4.3 以ABSL-1、ABSL-2、ABSL-3、ABSL-4 (animal bio-safety level，ABSL)表示包括从事动物活体操作的实验室的相应生物安全防护水平。

4. 4 根据实验活动的差异、采用的个体防护装备和基础隔离设施的不同，实验室分以下情况：

4.4.1 操作通常认为非经空气传播致病性生物因子的实验室。

4.4.2 可有效利用安仝隔离装置（如：生物安全柜）操作常规量经空气传播致病性生物因子的实验室。

4.4.3 不能有效利用安全隔离装置操作常规量经空气传播致病性生物因子的实验室。

4.4.4 利用具有生命支持系统的正压服操作常规量经空气传播致病性生物因子的实验室。

4.5 应依据国家相关主管部门发布的病原微生物分类名录，在风险评估的基础上，确定实验室的生物安全防护水平。

5. 实验室设计原则及基本要求

5.1 实验室选址、设计和建造应符合国家和地方环境保护和建设主管部门等的规定和要求。

5.2 实验室的防火和安全通道设置应符合国家的消防规定和要求，同时应考虑生物安全的特殊要求；必要时，应事先征询消防主管部门的建议。

5.3 实验室的安全保卫应符合国家相关部门对该类设施的安全管理规定和要求。

5.4 实验室的建筑材料和设备等应符合国家相关部门对该类产品生产、销售和使用的规定和要求。

5.5 实验室的设计应保证对生物、化学、辐射和物理等危险源的防护水平控制在经过评估的可接受程度，为关联的办公区和邻近的公共空间提供安全的工作环境，及防止危害环境。

5.6 实验室的走廊和通道应不妨碍人员和物品通过。

5.7 应设计紧急撤离路线，紧急出口应有明显的标识。

5.8 房间的门根据需要安装门锁，门锁虚便于内部快速打开。

5.9 需要时（如：正当操作危险材料时），房间的入口处应有警示和进入限制。

5.10 应评估生物材料、样本、药品、化学品和机密资料等被误用、被偷盗和被不正当使用的风险，并采取相应的物理防范措施。

5.11 应有专门设计以确保存储、转运、收集、处理和处置危险物料的安全。

5.12 实验室内温度、湿度、照度、噪声和洁净度等室内环境参数应符合工作要求和卫生等相关要求。

5.13 实验室设计还应考虑节能、环保及舒适性要求，应符合职业卫生要求和人机工效学要求。

5.14 实验室应有防止节肢动物和啮齿动物进入的措施。

5.15 动物实验室的生物安全防护设施还应考虑对动物呼吸、排泄、毛发、抓咬、挣扎、逃逸、动物实验（如：染毒、医学检查、取样、解剖、检验等）、动物饲养、动物尸体及排泄物的处置等过程产生的潜在生物危险的防护。

5.16 应根据动物的种类、身体大小、生活习性、实验目的等选择具有适当防护水平的、适用于动物的饲养设施、实验设施、消毒灭菌设施和清洗设施等。

5.17 不得循环使用动物实验室排出的空气。

5.18 动物实验室的设计，如：空间、进出通道、解剖室、笼具等应考虑动物实验及动物福利的要求。

5.19 适用时，动物实验室还应符合国家实验动物饲养设施标准的要求。

6. 实验室设施和设备要求

6.1 BSL-1实验室

6.1.1 实验室的门应有可视窗并可锁闭，门锁及门的开启方向应不妨碍室内人员逃生。

6.1.2 应设洗手池，宜设置在靠近实验室的出口处。

6.1.3 在实验室门口处应设存农或挂衣装置，可将个人服装与实验室工作服分开放置。

6.1.4 实验室的墙壁、天花板和地面应易清洁、不渗水、耐化学品和消毒灭菌剂的腐蚀。地面应平整、防滑，不应铺设地毯。

6.1.5 实验室台柜和座椅等应稳固，边角应圆滑。

6.1.6 实验室台柜等和其摆放应便于清洁，实验台面应防水、耐腐蚀、耐热和坚固。

6.1.7 实验室应有足够的空间和台柜等摆放实验室设备和物品。

6.1.8 应根据工作性质和流程合理摆放实验室设备、台柜、物品等，避免相互干扰、交叉污染，并应不妨碍逃生和急救。

6.1.9 实验室可以利用自然通风。如果采用机械通风，应避免交叉污染。

6.1.10 如果有可开启的窗户，应安装可防蚊虫的纱窗。

6.1.11 实验室内应避免不必要的反光和强光。

6.1.12 若操作刺激或腐蚀性物质，应在30 m内设洗眼装置，必要时应设紧急喷淋装置。

6.1.13 若操作有毒、刺激性、放射性挥发物质，应在风险评估的基础上，配备适当的负压排风柜。

6.1.14 若使用高毒性、放射性等物质，应配备相应的安全设施、设备和个体防护装备，应符合国家、地方的相关规定和要求。

6.1.15 若使用高压气体和可燃气体，应有安全措施，应符合国家、地方的相关规定和要求。

6.1.16 应设应急照明装置。

6.1.17 应有足够的电力供应。

6.1.18 应有足够的固定电源插座，避免多台设备使用共同的电源插座。应有可靠的接地系统，应在关键节点安装漏电保护装置或监测报警装置。

6.1.19 供水和排水管道系统应不渗漏，下水应有防回流设计。

6.1.20 应配备适用的应急器材，如消防器材、意外事故处理器材、急救器材等。

6.1.21 应配备适用的通讯设备。

6.1.22 必要时，应配备适当的消毒灭菌设备。

6.2 BSL-2实验室

6.2.1 适用时，应符合6.1的要求。

6.2.2 实验室主入口的门、放置生物安全柜实验间的门应可自动关闭；实验室主入口的门应有进入控制措施。

6.2.3 实验室工作区域外应有存放备用物品的条件。

6.2.4 应在实验室工作区配备洗眼装置。

6.2.5 应在实验室或其所在的建筑内配备高压蒸汽灭菌器或其他适当的消毒灭菌设备，所配备的消毒灭菌设备应以风险评估为依据。

6.2.6 应在操作病原微生物样本的实验间内配备生物安全柜。

6.2.7 应按产品的设计要求安装和使用生物安全柜。如果生物安全柜的排风在室内循环，室内应具备通风换气的条件；如果使用需要管道排风的生物安全柜，应通过独立于建筑物其他公英通风系统的管道排出。

6.2.8 应有可靠的电力供应。必要时，重要设备（如：培养箱、生物安全柜、冰箱等）应配置备用电源。

6.3 BSL-3实验室

6.3.1 平面布局

6.3.1.1 实验室应明确区分辅助工作区和防护区，应在建筑物中自成隔离区或为独立建筑物，应有出入控制。

6.3.1.2 防护区中直接从事高风险操作的工作间为核心工作间，人员应通过缓冲间进入核心工作间。

6.3.1.3 适用于4.4.1的实验室辅助工作区应至少包括监控室和清洁衣物更换间；防护区应至少包括缓冲间（可兼作脱防护服间）及核心工作间。

6.3.1.4 适用于4.4.2的实验室辅助工作区应至少包括监控室、清洁衣物更换间和淋浴间；防护区应至少包括防护服更换间、缓冲间及核心工作间。

6.3.1.5 适用于4.4.2的实验室核心工作间不宜直接与其他公共区域相邻。

6.3.1.6 如果安装传递窗，其结构承压力及密闭性应符合所在区域的要求，并具备对传递窗内物品进行消毒灭菌的条件。必要时，应设置具备送排风或自净化功能的传递窗，排风应经HEPA二过滤器过滤后排出。

6.3.2 围护结构

6.3.2.1 围护结构（包括墙体）应符合国家对该类建筑的抗震要求和防火要求。

6.3.2.2 天花板、地板、墙间的交角应易清洁和消毒灭菌。

6.3.2.3 实验室防护区内围护结构的所有缝隙和贯穿处的接缝都应可靠密封。

6.3.2.4 实验室防护区内围护结构的内表面应光滑、耐腐蚀、防水，以易于清洁和消毒灭菌。

6.3.2.5 实验室防护区内的地面应防渗漏、完整、光洁、防滑、耐腐蚀、不起尘。

6.3.2.6 实验室内所有的门应可自动关闭，需要时，应设观察窗；门的开启方向不应妨碍逃生。

6.3.2.7 实验室内所有窗户应为密闭窗，玻璃应耐撞击、防破碎。

6.3.2.8 实验室及设备间的高度应满足设备的安装要求，应有维修和清洁空间。

6.3.2.9 在通风空调系统正常运行状态下，采用烟雾测试等目视方法检查实验室防护区内围护结构的严密性时，所有缝隙应无可见泄漏（参见附录A）。

6.3.3 通风空调系统

6.3.3.1 应安装独立的实验室送排风系统，应确保在实验室运行时气流由低风险区向高风险区流动，同时确保实验室空气只能通过HEPA过滤器过滤后经专用的排风管道排出。

6.3.3.2 实验室防护区房间内送风口和排风口的布置应符合定向气流的原则，利于减少房间内的涡流和气流死角；送排风应不影响其他设备（如：II级生物安全柜）的正常功能。

6.3.3.3 不得循环使用实验室防护区排出的空气。

6.3.3.4 应按产品的设计要求安装生物安全柜和其排风管道，可以将生物安全柜排出的空气排入实验室的排风管道系统。

6.3.3.5 实验室的送风应经过HEPA过滤器过滤，宜同时安装初效和中效过滤器。

6.3.3.6 实验室的外部排风口应设置在主导风的下风向（相对于送风口），与送风口的直线距离应大于12m，应至少高出本实验室所在建筑的顶部2m，应有防风、防雨、防鼠、防虫设计，但不应影响气体向上空排放。

6.3.3.7 HEPA过滤器的安装位置应尽可能靠近送风管道在实验室内的送风口端和排风管道在实验室内的排风口端。

6.3.3.8 应可以在原位对排风HEPA过滤器进行消毒灭菌和检漏（参见附录A）。

6.3.3.9 如在实验室防护区外使用高效过滤器单元，其结构应牢固，应能承受2500 Pa的压力；高效过滤器单元的整体密封性应达到在关闭所有通路并维持腔室内的温度在设计范围上限的条件下，若使空气压力维持在1000 Pa时，腔室内每分钟泄漏的空气量应不超过腔室净容积的0.1%。

6.3.3.10 应在实验室防护区送风和排风管道的关键节点安装生物型密闭阀，必要时，可完全关闭。应在实验室送风和排风总管道的关键节点安装生物型密闭阀，必要时，可完全关闭。

6.3.3.11 生物型密闭阀与实验室防护区相通的送风管道和排风管道应牢固、易消毒灭菌、耐腐蚀、抗老化，宜使用不锈钢管道；管道的密封性应达到在关闭所有通路并维持管道内的温度在设计范围上限的条件下，若使空气压力维持在500 Pa时，管道内每分钟泄漏的空气量应不超过管道内净容积的0.2%。

6.3.3.12 应有备用排风机。应尽可能减少排风机后排风管道正压段的长度，该段管道不应穿过其他房间。

6.3.3.13 不应在实验室防护区内安装分体空调。

6.3.4 供水与供气系统

6.3.4.1 应在实验室防护区内的实验间的靠近出口处设置非手动洗手设施；如果实验室不具备供水条件，则应设非手动手消毒灭菌装置。

6.3.4.2 应在实验室的给水与市政给水系统之间设防回流装置。

6.3.4.3 进出实验室的液体和气体管道系统应牢固、不渗漏、防锈、耐压、耐温（冷或热）、耐腐蚀。应有足够的空间清洁、维护和维修实验室内暴露的管道，应在关键节点安装截止阀、防回流装置或HEPA过滤器等。

6.3.4.4 如果有供气（液）罐等，应放在实验室防护区外易更换和维护的位置，安装牢固，不应将不相容的气体或液体放在一起。

6.3.4.5 如果有真空装置，应有防止真空装置的内部被污染的措施；不应将真空装置安装在实验场所之外。

6.3.5 污物处理及消毒灭菌系统

6.3.5.1 应在实验室防护区内设置生物安全型高压蒸汽灭菌器。宜安装专用的双扉高压灭菌器，其主体应安装在易维护的位置，与围护结构的连接之处应可靠密封。

6.3.5.2 对实验室防护区内不能高压灭菌的物品应由其他消毒灭菌措施。

6.3.5.3 高压蒸汽灭菌器的安装位置不应影响生物安全柜等安全隔离装置的气流。

6.3.5.4 如果设置传递物品的渡槽，应使用强度符合要求的耐腐蚀性材料，并方便更换消毒灭菌液。

6.3.5.5 淋浴间或缓冲间的地面液体收集系统应有防液体回流的装置。

6.3.5.6 实验室防护区内如果有下水系统，应与建筑物的下水系统完全隔离；下水应直接通向本实验室专用的消毒灭菌系统。

6.3.5.7 所有下水管道应有足够的倾斜度和排量，确保管道内不存水；管道的关键节点应按需要安装防回流装置、存水弯（深度应适用于空气压差的变化）或密闭阀门等；下水系统应符合相应的耐压、耐热、耐化学腐蚀的要求，安装牢固，无泄漏，便于维护、清洁和检查。

6.3.5.8 应使用可靠的方式处理处置污水（包括污物），并应对消毒灭菌效果进行监测，以确保达到排放要求。

6.3.5.9 应在风险评估的基础上，适当处理实验室辅助区的污水，并应监测，以确保排放到市政管网之前达到排放要求。

6.3.5.10 可以在实验室内安装紫外线消毒灯或其他适用的消毒灭菌装置。

6.3.5.11 应具备对实验室防护区及与其直接相通的管道进行消毒灭菌的条件。

6.3.5.12 应具备对实验室设备和安全隔离装置（包括与其直接相通的管道）进行消毒灭菌的条件。

6.3.5.13 应在实验室防护区内的关键部位配备便携的局部消毒灭菌装置（如：消毒喷雾器等），并备有足够的适用消毒灭菌剂。

6.3.6 电力供应系统

6.3.6.1 电力供应应满足实验室的所有用电要求，并应有冗余。

6.3.6.2 生物安全柜、送风机和排风机、照明、自控系统、监视和报警系统等应配备不间断备用电源，电力供应应至少维持30 min。

6.3.6.3 应在安全的位置设置专用配电箱。

6.3.7 照明系统

6.3.7.1 实验室核心工作间的照度应不低于350 lx，其他区域的照度应不低于200 lx，宜采用吸顶式防水洁净照明灯。

6.3.7.2 应避免过强的光线和光反射。

6.3.7.3 应设不少于30 min的应急照明系统。

6.3.8 自控、监视与报警系统

6.3.8.1 进入实验室的门应有门禁系统，应保证只有获得授权的人员才能进入实验室。

6.3.8.2 需要时，应可立即解除实验室门的互锁；应在互锁门的附近设置紧急手动解除互锁开关。

6.3.8.3 核心工作间的缓冲间的入口处应有指示核心工作间工作状态的装置（如：文字显示或指示灯），必要时，应同时设置限制进入核心工作间的连锁机制。

6.3.8.4 启动实验室通风系统时，应先启动实验室排风，后启动实验室送风；关停时，应先关闭生物安全柜等安全隔离装置和排风支管密闭阀，再关实验室送风及密闭阀，后关实验室排风及密闭阀。

6.3.8.5 当排风系统出现故障时，应有机制避免实验室出现正压和影响定向气流。

6.3.8.6 当送风系统出现故障时，应有机制避免实验室内的负压影响实验室人员的安全、影响生物安全柜等安全隔离装置的正常功能和围护结构的完整性。

6.3.8.7 应通过对可能造成实验室压力波动的设备和装置实行连锁控制等措施，确保生物安全柜、负压排风柜（罩）等局部排风设备与实验室送排风系统之间的压力关系和必要的稳定性，并应在启动、运行和关停过程中保持有序的压力梯度。

6.3.8.8 应设装置连续监测送排风系统HEPA过滤器的阻力，需要时，及时更换HEPA过滤器。

6.3.8.9 应在有负压控制要求的房间入口的显著位置，安装显示房间负压状况的压力显示装置和控制区间提示。

6.3.8.10 中央控制系统应可以实时监控、记录和存储实验室防护区内有控制要求的参数、关键设施设备的运行状态；应能监控、记录和存储故障的现象、发生时间和持续时间；应可以随时查看历史记录。

6.3.8.11 中央控制系统的信号采集间隔时间应不超过1 min，各参数应易于区分和识别。

6.3.8.12 中央控制系统应能对所有故障和控制指标进行报警，报警应区分一般报警和紧急报警。

6.3.8.13 紧急报警应为声光同时报警，应可以向实验室内外人员同时发出紧急警报；应在实验室核心工作间内设置紧急报警按钮。

6.3.8.14 应在实验室的关键部位设置监视器，需要时，可实时监视并录制实验室活动情况和实验室周围情况。监视设备应有足够的分辨率，影像存储介质应有足够的数据存储容量。

6.3.9 实验室通讯系统

6.3.9.1 实验室防护区内应设置向外部传输资料和数据的传真机或其他电子设备。

6.3.9.2 监控室和实验室内应安装语音通讯系统。如果安装对讲系统，宜采用向内通话受控、向外通话非受控的选择性通话方式。

6.3.9.3 通讯系统的复杂性应与实验室的规模和复杂程度相适应。

6.3.10 参数要求

6.3.10.1 实验室的围护结构应能承受送风机或排风机异常时导致的空气压力载荷。

6.3.10.2 适用于4.4.1的实验室核心工作间的气压（负压）与室外大气压的压差值应不小于30 Pa，与相邻区域的压差（负压）应不小于10 Pa；适用于4.4.2的实验室的核心工作间的气压（负压）与室外大气压的压差值应不小于40 Pa，与相邻区域的压差（负压）应不小于15 Pa。

6.3.10.3 实验室防护区各房间的最小换气次数应不小于12次/h

6.3.10.4 实验室的温度宜控制在18℃～26℃范围内。

6.3.10.5 正常情况下，实验室的相对湿度宜控制在30%～70%范围内；消毒状态下，实验室的相对湿度应能满足消毒灭菌的技术要求。

6.3.10.6 在安全柜开启情况下，核心工作间的噪声应不大于68 dB(A)。

6.3.10.7 实验室防护区的静态洁净度应不低于8级水平。

6.4 BSL-4实验室

6.4.1 适用时，应符合6.3的要求。

6.4.2 实验室应建造在独立的建筑物内或建筑物中独立的隔离区域内。应有严格限制进入实验室的门禁措施，应记录进入人员的个人资料、进出时间、授权活动区域等信息；对与实验室运行相关的关键区域也应有严格和可靠的安保措施，避免非授权进入。

6.4.3 实验室的辅助工作区应至少包括监控室和清洁衣物更换间。适用于4.4.2的实验室防护区应至少包括防护走廊、内防护服更换间、淋浴间、外防护服更换间和核心工作间，外防护服更换间应为气锁。

6.4.4 适用于4.4.4的实验室的防护区应包括防护走廊、内防护服更换间、淋浴间、外防护服更换间、化学淋浴间和核心工作间。化学淋浴间应为气锁，具备对专用防护服或传递物品的表面进行清洁和消毒灭菌的条件，具备使用生命支持供气系统的条件。

6.4.5 实验室防护区的围护结构应尽量远离建筑外墙；实验室的核心工作间应尽可能设置在防护区的中部。

6.4.6 应在实验室的核心工作间内配备生物安全型高压灭菌器；如果配备双扉高压灭菌器，其主体所在房间的室内气压应为负压，并应设在实验室防护区内易更换和维护的位置。

6.4.7 如果安装传递窗，其结构承压力及密闭性应符合所在区域的要求；需要时，应配备符合气锁要求的并具备消毒灭菌条件的传递窗。

6.4.8 实验室防护区围护结构的气密性应达到在关闭受测房间所有通路并维持房间内的温度在设计范围上限的条件下，当房间内的空气压力上升到500 Pa后，20 min内自然衰减的气压小于250 Pa。

6.4.9 符合4.4.4要求的实验室应同时配备紧急支援气罐，紧急支援气罐的供气时间应不少于60min／人。

6.4.10 生命支持供气系统应有自动启动的不间断备用电源供应，供电时间应不步于60 min。

6.4.11 供呼吸使用的气体的压力、流量、含氧量、温度、湿度、有害物质的含量等应符合职业安全的要求。

6.4.12 生命支持系统应具备必要的报警装置。

6.4.13 实验室防护区内所有区域的室内气压应为负压，实验室核心工作间的气压（负压）与室外大气压的压差值应不小于60 Pa，与相邻区域的压差（负压）应不小于25 Pa。

6.4.14 适用于4.4.2的实验室，应在III级生物安全柜或相当的安全隔离装置内操作致病性生物因子；同时应具备与安全隔离装置配套的物品传递设备以及生物安全型高压蒸汽灭菌器。

6.4.15 实验室的排风应经过两级HEPA过滤器处理后排放。

6.4.16 应可以在原位对送风HEPA过滤器进行消毒灭菌和检漏。

6.4.17 实验室防护区内所有需要运出实验室的物品或其包装的表面应经过可靠消毒灭菌。

6.4.18 化学淋浴消毒灭菌装置应在无电力供应的情况下仍可以使用，消毒灭菌剂储存器的容量应满足所有情况下对消毒灭菌剂使用量的需求。

6.5 动物生物安全实验室

6.5.1 ABSL-1实验室

6.5.1.1 动物饲养间应与建筑物内的其他区域隔离。

6.5.1.2 动物饲养间的门应有可视窗，向里开；打开的门应能够自动关闭，需要时，可以锁上。

6.5.1.3 动物饲养间的工作表面应防水和易于消毒灭菌。

6.5.1.4 不宜安装窗户。如果安装窗户，所有窗户应密闭；需要时，窗户外部应装防扩网。

6.5.1.5 围护结构的强度应与所饲养的动物种类相适应。

6.5.1.6 如果有地面液体收集系统，应设防液体回流装置，存水弯应有足够的深度。

6.5.1.7 不得循环使用动物实验室排出的空气。

6.5.1.8 应设置洗手池或手部清洁装置，宜设置在出口处。

6.5.1.9 宜将动物饲养间的室内气压控制为负压。

6.5.1.10 应可以对动物笼具清洗和消毒灭菌。

6.5.1.11 应设置实验动物饲养笼具或护栏，除考虑安全要求外还应考虑对动物福利的要求。

6.5.1.12 动物尸体及相关废物的处置设施和设备应符合国家相关规定的要求。

6.5.2 ABSL-2实验室

6.5.2.1 适用时，应符合6.5.1的要求。

6.5.2.2 动物饲养间应在出入口处设置缓冲间。

6.5.2.3 应设置非手动洗手池或手部清洁装置，宜设置在出口处。

6.5.2.4 应在邻近区域配备高压蒸汽灭菌器。

6.5.2.5 适用时，应在安全隔离装置内从事可能产生有害气溶胶的活动；排气应经HEPA过滤器的过滤后排出。

6.5.2.6 应将动物饲养间的室内气压控制为负压，气体应直接排放到其所在的建筑物外。

6.5.2.7 应根据风险评估的结果，确定是否需要使用HEPA过滤器过滤动物饲养间排出的气体。

6.5.2.8 当不能满足6.5.2.5时，应使用HEPA过滤器过滤动物饲养问排出的气体。

6.5.2.9 实验室的外部排风口应至少高出本实验室所在建筑的顶部2m，应有防风、防雨、防鼠、防虫设计，但不应影响气体向上空排放。

6.5.2.10 污水（包括污物）应消毒灭菌处理，并应对消毒灭菌效果进行监测，以确保达到排放要求。

6.5.3 ABSL-3实验室

6.5.3.1 适用时，应符合6.5.2的要求。

6.5.3.2 应在实验室防护区内设淋浴间，需要时，应设置强制淋浴装置。

6.5.3.3 动物饲养间属于核心工作间，如果有入口和出口，均应设置缓冲间。

6.5.3.4 动物饲养间应尽可能设在整个实验室的中心部位，不应直接与其他公共区域相邻。

6.5.3.5 适用于4.4.1实验室的防护区应至少包括淋浴间、防护服更换间、缓冲间及核心工作间。当不能有效利用安全隔离装置饲养动物时，应根据进一步的风险评估确定实验室的生物安全防护要求。

6.5.3.6 适用于4.4.3的动物饲养间的缓冲间应为气锁，并具备对动物饲养间的防护服或传递物品的表面进行消毒灭菌的条件。

6.5.3.7 适用于4.4.3的动物饲养间，应有严格限制进入动物饲养间的门禁措施（如：个人密码和生物学识别技术等）。

6.5.3.8 动物饲养间内应安装监视设备和通讯设备。

6.5.3.9 动物饲养间内应配备便携式局部消毒灭菌装置（如：消毒喷雾器等），并应备有足够的适用消毒灭菌剂。

6.5.3.10 应有装置和技术对动物尸体和废物进行可靠消毒天菌。

6.5.3.11 应有装置和技术对动物笼具进行清洁和可靠消毒灭菌。

6.5.3.12 需要时，应有装置和技术对所有物品或其包装的表面在运出动物饲养间前进行清洁和可靠消毒灭菌。

6.5.3.13 应在风险评估的基础上，适当处理防护区内淋浴间的污水，并应对灭菌效果进行监测，以确保达到排放要求。

6.5.3.14 适用于4.4.3的动物饲养间，应根据风险评估的结果，确定其排出的气体是否需要经过两级HEPA过滤器的过滤后排出。

6.5.3.15 适用于4.4.3的动物饲养间，应可以在原位对送风HEPA过滤器进行消毒灭菌和检漏。

6.5.3.16 适用于4.4.1和4.4.2的动物饲养间的气压（负压）与室外大气压的压差值应不小于60Pa，与相邻区域的压差（负压）应不小于15 Pa。

6.5.3.17 适用于4.4.3的动物饲养间的气压（负压）与室外大气压的压差值应不小于80 Pa，与相邻区域的压差（负压）应不小于25 Pa。

6.5.3.18 适用于4.4.3的动物饲养间及其缓冲间的气密性应达到在关闭受测房间所有通路并维持房间内的温度在设计范围上限的条件下，若使空气压力维持在250 Pa时，房间内每小时泄漏的空气量应不超过受测房间净容积的10%。

6.5.3.19 在适用于4.4.3的动物饲养间从事可传染人的病原微生物活动时，应根据进一步的风险评估确定实验室的生物安全防护要求；适用时，应经过相关主管部门的批准。

6.5.4 ABSL-4实验室

6.5.4.1 适用时，应符合6.5.3的要求。

6.5.4.2 淋浴间应设置强制淋浴装置。

6.5.4.3 动物饲养间的缓冲间应为气锁。

6.5.4.4 应有严格限制进入动物饲养间的门禁措施。

6.5.4.5 动物饲养间的气压（负压）与室外大气压的压差值应不小于100 Pa;与相邻区域的压差（负压）应不小于25 Pa。

6.5.4.6 动物饲养间及其缓冲间的气密性应达到在关闭受测房间所有通路并维持房间内的温度在设计范围上限的条件下，当房间内的空气压力上升到500 Pa后，20 min内自然衰减的气压小于250 Pa。

6.5.4.7 应有装置和技术对所有物品或其包装的表面在运出动物饲养间前进行清洁和可靠消毒灭菌。

6.5.5 对从事无脊椎动物操作实验室设施的要求

6.5.5.1 该类动物设施的生物安全防护水平应根据国家相关主管部门的规定和风险评估的结果确定。

6.5.5.2 如果从事某些节肢动物（特别是呵飞行、快爬或跳跃的昆虫）的实验活动，应采取以下适用的措施（但不限于）：

a) 应通过缓冲间进入动物饲养间，缓冲间内应安装适用的捕虫器，并应在门上安装防

节肢动物逃逸的纱网；

b) 应在所有关键的可开启的门窗上安装防节肢动物逃逸的纱网；

c) 应在所有通风管道的关键节点安装防节肢动物逃逸的纱网；应具备分房间饲养己感

染和未感染节肢动物的条件；

d) 应具备密闭和进行整体消毒灭菌的条件；

e) 应设喷雾式杀虫装置；

f) 应设制冷装置，需要时，可以及时降低动物的活动能力；

g) 应有机制确保水槽和存水弯管内的液体或消毒灭菌液不干涸；

h) 只要可行，应对所有废物高压灭菌；

i) 应有机制监测和记录会飞、爬、跳跃的节肢动物幼虫和成虫的数量；

j) 应配备适用于放置装蜱螨容器的油碟；

k) 应具备带双层网的笼具以饲养或观察已感染或潜在感染的逃逸能力强的节肢动物；

1) 应具备适用的生物安全柜或相当的安全隔离装置以操作已感染或潜在感染的节肢动物；

m) 应具备操作已感染或潜在感染的节肢动物的低温盘；

n) 需要时，应设置监视器和通讯设备。

6.5.5.3 是否需要其他措施，应根据风险评估的结果确定。

7． 管理要求

7.1 组织和管理

7.1.1 实验室或其母体组织应有明确的法律地位和从事相关活动的资格。

7.1.2 实验室所在的机构应设立生物安全委员会，负责咨询、指导、评估、监督实验室的生物安全相关事宜。实验室负责人应至少是所在机构生物安全委员会有职权的成员。

7.1.3 实验室管理层应负责安全管理体系的设计、实施、维持和改进，应负责：

a) 为实验室所有人员提供履行其职责所需的适当权力和资源；

b) 建立机制以避免管理层和实验室人员受任何不利于其工作质量的压力或影响（如：

财务、人事或其他方面的），或卷入任何可能降低其公正性、判断力和能力的活动；

c) 制定保护机密信息的政策和程序；

d) 明确实验室的组织和管理结构，包括与其他相关机构的关系；

e) 规定所有人员的职责、权力和相互关系；

f) 安排有能力的人员，依据实验室人员的经验和职责对其进行必要的培训和监督；

g) 指定一名安全负责人，赋予其监督所有活动的职责和权力，包括制定、维持、监督

实验室安全计划的责任，阻止不安全行为或活动的权力，直接向决定实验室政策和

资源的管理层报告的权力；

h) 指定负责技术运作的技术管理层，并提供可以确保满足实验室规定的安全要求和技

术要求的资源；

i) 指定每项活动的项目负责人，其负责制定并向实验室管理层提交活动计划、风险评

估报告、安全及应急措施、项目组人员培训及健康监督计划、安全保障及资源要求；

j) 指定所有关键职位的代理人。

7.1.4 实验室安全管理体系应与实验室规模、实验室活动的复杂程度和风险相适应。

7.1.5 政策、过程、计划、程序和指导书等应文件化并传达至所有相关人员。实验室管理层应保证这些文件易于理解并可以实施。

7.1.6 安全管理体系文件通常包括管理手册、程序文件、说明及操作规程、记录等文件，应有供现场工作人员快速使用的安全手册。

7.1.7 应指导所有人员使用和应用与其相关的安全管理体系文件及其实施要求，并评估其理解和运用的能力。

7.2 管理责任

7.2.1 实验室管理层应对所有员工、来访者、合同方、社区和环境的安全负责。

7.2.2 应制定明确的准入政策并主动告知所有员工、来访者、合同方可能面临的风险。

7.2.3 应尊重员工的个人权利和隐私。

7.2.4 应为员工提供持续培训及继续教育的机会，保证员工可以胜任所分配的工作。

7.2.5 应为员工提供必要的免疫计划、定期的健康检查和医疗保障。

7.2.6 应保证实验室设施、设备、个体防护装备、材料等符合国家有关的安全要求，并定期检查、维护、更新，确保不降低其设计性能。

7.2.7 应为员工提供符合要求的适用防护用品和器材。

7.2.8 应为员工提供符合要求的适用实验物品和器材。

7.2.9 应保证员工不疲劳工作和不从事风险不可控制的或国家禁止的工作。

7.3 个人责任

7.3.1 应充分认识和理解所从事工作的风险。

7.3.2 应自觉遵守实验室的管理规定和要求。

7.3.3 在身体状态许可的情况下，应接受实验室的免疫计划和其他的健康管理规定。

7.3.4 应按规定正确使用设施、设备和个体防护装备。

7.3.5 应主动报告可能不适于从事特定任务的个人状态。

7.3.6 不应因人事、经济等任何压力而违反管理规定。

7.3.7 有责任和义务避免因个人原因造成生物安全事件或事故。

7.3.8 如果怀疑个人受到感染，应立即报告。

7.3.9 应主动识别任何危险和不符合规定的工作，并立即报告。

7.4 安全管理体系文件

7.4.1 实验室安全管理的方针和目标

7.4.1.1 在安全管理手册中应明确实验室安全管理的方针和目标。安全管理的方针应简明扼要，至少包括以下内容：

a) 实验室遵守国家以及地方相关法规和标准的承诺；

b) 实验室遵守良好职业规范、安全管理体系的承诺；

c) 实验室安全管理的宗旨。

7.4.1.2 实验室安全管理的目标应包括实验室的工作范围、对管理活动和技术活动制定的安全指标，应明确、可考核。

7.4.1.3 应在风险评估的基础上确定安全管理目标，并根据实验室活动的复杂性和风险程度定期评审安全管理目标和制定监督检查计划。

7.4.2 安全管理手册

7.4.2.1 应对组织结构、人员岗位及职责、安全及安保要求、安全管理体系、体系文件架构等进行规定和描述。安全要求不能低于国家和地方的相关规定及标准的要求。

7.4.2.2 应明确规定管理人员的权限和责任，包括保证其所管人员遵守安全管理体系要求的责任。

7.4.2.3 应规定涉及的安全要求和操作规程应以国家主管部门和世界卫生组织、世界动物卫生组织、国际标准化组织等机构或行业权威机构发布的指南或标准等为依据，并符合国家相关法规和标准的要求；任何新技术在使用前应经过充分验证，适用时，应得到国家相关主管部门的批准。

7.4.3 程序文件

7.4.3.1 应明确规定实施具体安全要求昀责任部门、责任范围、工作流程及责任人、任务安排及对操作人员能力的要求、与其他责任部门的关系、应使用的工作文件等。

7.4.3.2 应满足实验室实施所有的安全要求和管理要求的需要，工作流程清晰，各项职责得到落实。

7.4.4 说明及操作规程

7.4.4.1 应详细说明使用者的权限及资格要求、潜在危险、设施设备的功能、活动目的和具体操作步骤、防护和安全操作方法、应急措施、文件制定的依据等。

7.4.4.2 实验室应维持并合理使用实验室涉及的所有材料的最新安全数据单。

7.4.5 安全手册

7.4.5.1 应以安全管理体系文件为依据，制定实验室安全手册（快速阅读文件）；应要求所有员工阅读安全手册并在工作区随时可供使用；安全手册宜包括（但不限于）以下内容：

a) 紧急电话、联系人；

b) 实验室平面图、紧急出口、撤离路线；

c) 实验室标识系统；

d) 生物危险；

e) 化学品安全；

f) 辐射；

g) 机械安全；

h) 电气安全；

i) 低温、高热；

j) 消防；

k) 个体防护；

1) 险废物的处理和处置；

m) 事件、事故处理的规定和程序；

n) 从工作区撤离的规定和程序。

7.4.5.2 安全手册应简明、易懂、易读，实验室管理层应至少每年对安全手册评审和更新。

7.4.6 记录

7.4.6.1 应明确规定对实验室活动进行记录的要求，至少应包括：记录的内容、记录的要求、记录的档案管理、记录使用的权限、记录的安全、记录的保存期限等。保存期限应符合国家和地方法规或标准的要求。

7.4.6.2 实验室应建立对实验室活动记录进行识别、收集、索引、访问、存放、维护及安全处置的程序。

7.4.6.3 原始记录应真实并可以提供足够的信息，保证可追溯性。

7.4.6.4 对原始记录的任何更改均不应影响识别被修改的内容，修改人应签字和注明日期。

7.4.6.5 所有记录应易于阅读，便于检索。

7.4.6.6 记录可存储于任何适当的媒介，应符合国家和地方的法规或标准的要求。

7.4.6.7 应具备适宜的记录存放条仵，以防损坏、变质、丢失或未经授权的进入。

7.4.7 标识系统

7.4.7.1 实验室用于标示危险区、警示、指示、证明等的图文标识是管理体系文件的一部分，包括用于特殊情况下的临时标识，如“污染”、“消毒中”、“设备检修”等。

7.4.7.2 标识应明确、醒目和易区分。只要可行，应使用国际、国家规定的通用标识。

7.4.7.3 应系统而清晰地标示出危险区，且应适用于相关的危险。在某些情况下，宜同时使用标识和物理屏障标示出危险区。

7.4.7.4 应清楚地标示出具体的危险材料、危险，包括：生物危险、有毒有害、腐蚀性、辐射、刺伤、电击、易燃、易爆、高温、低温、强光、振动、噪声、动物咬伤、砸伤等；需要时，应同时提示必要的防护措施。

7.4.7.5 应在须验证或校准的实验室设备的明显位置注明设备的可用状态、验证周期、下次验证或校准的时间等信息。

7.4.7.6 实验室入口处应有标识，明确说明生物防护级别、操作的致病性生物因子、实验室负责人姓名、紧急联络方式和国际通用的生物危险符号；适用时，应同时注明其他危险。

7.4.7.7 实验室所有房间的出口和紧急撤离路线应有在无照明的情况下也可清楚识别的标识。

7.4.7.8 实验室的所有管道和线路应有明确、醒目和易区分的标识。

7.4.7.9 所有操作开关应有明确的功能指示标识，必要时，还应采取防止误操作或恶意操作的措施。

7.4.7.10实验室管理层应负责定期（至少每12个月一次）评审实验室标识系统，需要时及时更新，以确保其适用现有的危险。

7.5 文件控制

7.5.1 实验室应对所有管理体系文件进行控制，制定和维持文件控制程序，确保实验室人员使用现行有效的文件。

7.5.2 应将受控文件备份存档，并规定其保存期限。文件可以用任何适当的媒介保存，不限定为纸张。

7.5.3 应有相应的程序以保证：

a) 管理体系所有的文件应在发布前经过授权人员的审核与批准；

b) 动态维持文件清单控制记录，并可以识别现行有效的文件版本及发放情况；

c) 在相关场所只有现行有效的文件可供使用；

d) 定期评审文件，需要修订的文件经授权人员审核与批准后及时发布；

e) 及时撤掉无效或已废止的文件，或可以确保不误用；

f) 适当标注存留或归档的己废止文件，以防误用。

7.5.4 如果实验室的文件控制制度允许在换版之前对文件手写修改，应规定修改程序和权限。修改之处应有清晰的标注、签署并注明日期。被修改的文件应按程序及时发布。

7.5.5 应制定程序规定如何更改和控制保存在计算机系统中的文件。

7.5.6 安全管理体系文件应具备唯一识别性，文件中应包括以下信息：

a) 标题；

b) 文件编号、版本号、修订号；

c) 页数；

d) 生效日期；

e) 编制人、审核人、批准人；

f) 参考文献或编制依据。

7.6 安全计划

7.6.1 实验室安全负责人应负责制定年度安全计划，安全计划应经过管理层的审核与批准。需要时，实验室安全计划应包括（不限于）：

a) 实验室年度工作安排的说明和介绍；

b) 安全和健康管理目标；

c) 风险评估计划；

d) 程序文件与标准操作规程的制定与定期评审计划；

e) 人员教育、培训及能力评估计划；

f) 实验室活动计划；

g) 设施设备校准、验证和维护计划；

h) 危险物品使用计划；

i) 消毒灭菌计划；

j) 废物处置计划；

k) 设备淘汰、购置、更新计划；

1) 演习计划（包括泄漏处理、人员意外伤害、设施设备失效、消防、应急预案等）；

m) 监督及安全检查计划（包括核查表）；

n) 人员健康监督及免疫计划；

o) 审核与评审计划；

p) 持续改进计划；

q) 外部供应与服务计划；

r) 行业最新进展跟踪计划；

s) 与生物安全委员会相关的活动计划。

7.7 安全检查

7.7.1 实验室管理层应负责实施安全检查，每年应至少根据管理体系的要求系统性地检查一次，对关键控制点可根据风险评估报告适当增加检查频率，以保证：

a) 设施设备的功能和状态正常；

b) 警报系统的功能和状态正常；

c) 应急装备的功能及状态正常；

d) 消防装备的功能及状态正常；

e) 危险物品的使用及存放安全；

f) 废物处理及处置的安全；

g) 人员能力及健康状态符合工作要求；

h) 安全计划实施正常；

i) 实验室活动的运行状态正常；

j) 不符合规定的工作及时得到纠正；

k) 所需资源满足工作要求。

7.7.2 力保证检查工作的质量，应依据事先制定的适用于不同工作领域的核查表实施检查。

7.7.3 当发现不符合规定的工作、发生事件或事故时，应立即查找原因并评估后果；必要时，停止工作。

7.7.4 生物安全委员会应参与安全检查。

7.7.5 外部的评审活动不能代替实验室的自我安全检查。

7.8 不符合项的识别和控制

7.8.1 当发现有任何不符合实验室所制定的安全管理体系的要求时，实验室管理层应按需要采取以下措施（不限于）：

a) 将解决问题的责任落实到个人；

b) 明确规定应采取的措施；

c) 只要发现很有可能造成感染事件或其他损害，立即终止实验室活动并报告；

d) 立即评估危害并采取应急措施；

e) 分析产生不符合项的原因和影响范围，只要适用，应及时采取补救措施；

f) 进行新的风险评估；

g) 采取纠正措施并验证有效；

h) 明确规定恢复工作的授权人及责任；

i) 记录每一不符合项及其处理的过程并形成文件；

7.8.2 实验室管理层应按规定的周期评审不符合项报告，以发现趋势并采取预防措施。

7.9 纠正措施

7.9.1 纠正措施程序中应包括识别问题发生的根本原因的调查程序。纠正措施应与问题的严重性及风险的程度相适应。只要适用，应及时采取预防措施。

7.9.2 实验室管理层应将因纠正措施所致的管理体系的任何改变文件化并实施。

7.9.3 实验室管理层应负责监督和检查所采取纠正措施的效果，以确保这些措施已有效解决了识别出的问题。

7.10 预防措施

7.10.1 虚识别无论是技术还是管理体系方面的不符合项来源和所需的改进，定期进行趋势分析和风险分析，包括对外部评价的分析。如果需要采取预防措施，应制定行动计划、监督和检查实效果，以减少类似不符合项发生的可能性并借机改进。

7.10.2 预防措施程序应包括对预防措施的评价，以确保其有效性。

7.11 持续改进

7.11.1 实验室管理层应定期系统地评审管理体系，以识别所有潜在的不符合项来源、识别对管理体系或技术的改进机会。适用时，应及时改进识别出的需改进之处，应制定改进方案，文件化、实施并监督。

7.11.2 实验室管理层应设置可以系统地监测、评价实验室活动风险的客观指标。

7.11.3 如果采取措施，实验室管理层还应通过重点评审或审核相关范围的方式评价其效果。

7.11.4 需要时，实验室管理层应及时将因改进措施所致的管理体系的任何改变文件化并实施。

7.11.5 实验室管理层应有机制保证所有员工积极参加改进活动，并提供相关的教育和培训机会。

7.12 内部审核

7. 12.1 应根据安全管理体系的规定对所有管理要素和技术要素定期进行内部审核，以证实管理体系的运作持续符合要求。

7.12.2 应由安全负责人负责策划、组织并实施审核。

7.12.3 应明确内部审核程序并文件化，应包括审核范围、频次、方法及所需的文件。如果发现不足或改进机会，应采取适当的措施，并在约定的时间内完成。

7. 12.4 正常情况下，应按不大于12个月的周期对管理体系的每个要素进行内部审核。

7.12.5 员工不应审核自己的工作。

7.12.6 应将内部审核的结果提交实验室管理层评审。

7.13 管理评审

7.13.1 实验室管理层应对实验室安全管理体系及其全部活动进行评审，包括设施设备的状态、人员状态、实验室相关的活动、变更、事件、事故等。

7.13.2 需要时，管理评审应考虑以下内容（不限于）：

a) 前次管理评审输出的落实情况；

b) 所采取纠正措施的状态和所需的预防措施；

c) 管理或监督人员的报告；

d) 近期内部审核的结果；

e) 安全检查报告；

f) 适用时，外部机构的评价报告；

g) 任何变化、变更情况的报告；

h) 设施设备的状态报告；

i) 管理职责的落实情况；

j) 人员状态、培训、能为评估报告；

k) 员工健康状况报告；

1) 不符合项、事件、事故及其调查报告；

m) 实验室工作报告；

n) 风险评估报告；

o) 持续改进情况报告；

p) 对服务供应商的评价报告；

q) 国际、国家和地方相关规定和技术标准的更新与维持情况；

r) 安全管理方针及目标；

s) 管理体系的更新与维持；

t) 安全计划的落实情况、年度安全计划及所需资源。

7.13.3 只要可行，应以客观方式监测和评价实验室安全管理体系的适用性和有效性。

7.13.4 应记录管理评审的发现及提出的措施，应将评审发现和作为评审输出的决定列入含目的、目标和措施的工作计划中，并告知实验室人员。实验室管理层应确保所提出的措施在规定的时间内完成。

7.13.5 正常情况下，应按不大于12个月的周期进行管理评审。

7.14 实验室人员管理

7. 14.1 必要时，实验室负责人应指定若干适当的人员承担实验室安全相关的管理职责。实验室安全管理人员应：

a) 具备专业教育背景；

b) 熟悉国家相关政策、法规、标准；

c) 熟悉所负责的工作，有相关的工作经历或专业培训；

d) 熟悉实验室安全管理工作；

e) 定期参加相关的培训或继续教育。

7.14.2 实验室或其所在机构应有明确的人事政策和安排，并可供所有员工查阅。

7.14.3 应对所有岗位提供职责说明，包括人员的责任和任务，教育、培训和专业资格要求，应提供给相应岗位的每位员工。

7.14.4 应有足够的人力资源承担实验室所提供服务范围内的工作以及承担管理体系涉及的工作。

7.14.5 如果实验室聘用临时工作人员，应确保其有能力胜任所承担的工作，了解并遵守实验室管理体系的要求。

7.14.6 员工的工作量和工作时间安排不应影响实验室酒动的质量和员工的健康，符合国家法规要求。

7.14.7 在有规定的领域，实验室人员在从事相关的实验室活动时，应有相应的资格。

7.14.8 应培训员工独立工作的能力。

7.14.9 应定期评价员工可以胜任其工作任务的能力。

7.14.10应按工作的复杂程度定期评价所有员工的表现，应至少每12个月评价一次。

7.14.11人员培训计划应包括（不限于）：

a) 上岗培训，包括对较长期离岗或下岗人员的再上岗培训；

b) 实验室管理体系培训；

c) 安全知识及技能培训；

d) 实验室设施设备（包括个体防护装备）的安全使用；

e) 应急措施与现场救治；

f) 定期培训与继续教育；

g) 人员能力的考核与评估。

7.14.12 实验室或其所在机构应维持每个员工的人事资料，可靠保存并保护隐私权。人事档案应包括（不限于）：

a) 员工的岗位职责说明；

b) 岗位风险说明及员工的知情同意证明；

c) 教育背景和专业资格证明；

d) 培训记录，应有员工与培训者的签字及日期；

e) 员工的免疫、健康检查、职业禁忌症等资料；

f) 内部和外部的继续教育记录及成绩；

g) 与工作安全相关的意外事件、事故报告；

h) 有关确认员工能力的证据，应有能力评价的日期和承认该员工能力的日期或期限；

i) 员工表现评价。

7.15 实验室材料管理

7.15.1 实验室应有选择、购买、采集、接收、查验、使用、处置和存储实验室材料（包括外部服务）的政策和程序，以保证安全。

7.15.2 应确保所有与安全相关的实验室材料只有在经检查或证实其符合有关规定的要求之后投入使用，应保存相关活动的记录。

7.15.3 应评价重要消耗品、供应品和服务的供应商，保存评价记录和允许使用的供应商名单。

7.15.4 应对所有危险材料建立清单，包括来源、接收、使用、处置、存放、转移、使用权限、时间和数量等内容，相关记录安全保存，保存期限不少于20年。

7.15.5 应有可靠的物理措施和管理程序确保实验室危险材料的安全和安保。

7.15.6 应按国家相关规定的要求使用和管理实验室危险材料。

7.16 实验室活动管理

7. 16.1 实验室应有计划、申请、批准、实施、监督和评估实验室活动的政策和程序。

7.16.2 实验室负责人应指定每项实验室活动的项目负责人，同时见7.1.3 i）。

7.16.3 在开展活动前，应了解实验室活动涉及的任何危险，掌握良好工作行为（参见附录B）；为实验人员提供如何在风险最小情况下进行工作的详细指导，包括正确选择和使用个体防护装备。

7.16.4 涉及微生物的实验室活动操作规程应利用良好微生物标准操作要求和（或）特殊操作要求。

7.16.5 实验室应有针对未知风险材料操作的政策和程序。

7.17 实验室内务管理

7.17.1 实验室应有对内务管理的政策和程序，包括内务工作所用清洁剂和消毒灭菌剂的选择、配制、效期、使用方法、有效成分检测及消毒灭菌效果监测等政策和程序，应评估和避免消毒灭菌剂本身的风险。

7.17.2 不应在工作面放置过多的实验室耗材。

7.17.3 应时刻保持工作区整洁有序。

7.17.4 应指定专人使用经核准的方法和个体防护装备进行内务工作。

7.17.5 不应混用不同风险区的内务程序和装备。

7.17.6 应在安全处置后对被污染的区域和可能被污染的区域进行内务工作。

7.17.7 应制定日常清洁（包括消毒灭菌）计划和清场消毒灭菌计划，包括对实验室设备和工作表面的消毒灭菌和清洁。

7.17.8 应指定专人监督内务工作，应定期评价内务工作的质量。

7.17.9 实验室的内务规程和所用材料发生改变时应通知实验室负责人。

7.17.10实验室规程、工作习惯或材料的改变可能对内务人员有潜在危险时，应通知实验室负责人并书面告知内务管理负责人。

7.17.11发生危险材料溢洒时，应启用应急处理程序。

7.18 实验室设施设备管理

7.18.1 实验室应有对设施设备（包括个体防护装备）管理的政策和程序，包括设施设备的完好性监控指标、巡检计划、使用前核查、安全操作、使用限制、授权操作、消毒灭菌、禁止事项、定期校准或检定，定期维护、安全处置、运输、存放等。

7.18.2 应制定在发生事故或溢洒（包括生物、化学或放射性危险材料）时，对设施设备去污染、清洁和消毒灭菌的专用方案（参见附录C）。

7.18.3 设施设备维护、修理、报废或被移出实验室前应先去污染、浦洁和消毒灭菌；但应意识到，可能仍然需要要求维护人员穿戴适当的个体防护装备。

7.18.4 应明确标示出设施设备中存在危险的部位。

7.18.5 在投入使用前应核查并确认设施设备的性能可满足实验室的安全要求和相关标准。

7.18.6 每次使用前或使用中应根据监控指标确认设施设备的性能处于正常工作状态，并记录。

7.18.7 如果使用个体呼吸保护装置，应做个体适配性测试，每次使用前核查并确认符合佩戴要求。

7.18.8 设施设备应由经过授权的人员操作和维护，现行有效的使用和维护说明书应便于有关人员使用。

7.18.9 应依据制造商的建议使用和维护实验室设施设备。

7.18.10应在设施设备的显著部位标示出其唯一编号、校准或验证日期、下次校准或验证日期、准用或停用状态。

7.18.11应停止使用并安全处置性能己显示出缺陷或超出规定限度的设施设备。

7.18.12无论什么原因，如果设备脱离了实验室的直接控制，待该设备返回后，应在使用前对其性能进行确认并记录。

7.18.13应维持设施设备的档案，适用时，内容应至少包括（不限于）：

a) 制造商名称、型式标识、系列号或其他唯一性标识；

b) 验收标准及验收记录；

c) 接收日期和启用日期；

d) 接收时的状态（新品、使用过、修复过）；

e) 当前位置；

f) 制造商的使用说明或其存放处；

g) 维护记录和年度维护计划；

h) 校准（验证）记录和校准（验证）计划；

i) 任何损坏、故障、改装或修理记录；

j) 服务合同；

k) 预计更换日期或使用寿命；

1) 安全检查记录。

7.19 废物处置

7.19.1 实验室危险废物处理和处置的管理应符合国家或地方法规和标准的要求，应征询相关主管部门的意见和建议。

7.19.2 应遵循以下原则处理和处置危险废物：

a) 将操作、收集、运输、处理及处置废物的危险减至最小；

b) 将其对环境的有害作用减至最小；

c) 只可使用被承认的技术和方法处理和处置危险废物；

d) 排放符合国家或地方规定和标准的要求。

7.19.3 应有措施和能力安全处理和处置实验室危险废物。

7.19.4 应有对危险废物处理和处置的政策和程序，包括对排放标准及监测的规定。

7.19.5 应评估和避免危险废物处理和处置方法本身的风险。

7.19.6 应根据危险废物的性质和危险性按相关标准分类处理和处置废物。

7.19.7 危险废物应弃置于专门设计的、专月的和有标识的用于处置危险废物的容器内，装量不能超过建议的装载容量。

7.19.8 锐器（包括针头、小刀、金属和玻璃等）应直接弃置于耐扎的容器内。

7.19.9 应由经过培训的人员处理危险废物，并应穿戴适当的个体防护装备。

7.19.10 不应积存垃圾和实验室废物。在消毒灭菌或最终处置之前，应存放在指定的安全地方。

7.19.11 不应从实验室取走或排放不符合相关运输或排放要求的实验室废物。

7.19.12 应在实验室内消毒灭菌含活性高致病性生物因子的废物。

7.19.13 如果法规许可，只要包装和运输方式符合危险废物的运输要求，可以运送未处理的危险废物到指定机构处理。

7.20 危险材料运输

7.20.1 应制定对危险材料运输的政策和程序，包括危险材料在实验室内、实验室所在机构内及机构外部的运输，应符合国家和国际规定的要求。

7.20.2 应建立并维持危险材料接收和运出清单，至少包括危险材料的性质、数量、交接时包装的状态、交接人、收发时间和地点等，确保危险材料出入的可追溯性。

7.20.3 实验室负责人或其授权人员应负责向为实验室送交危险材料的所有部门提供适当的运输指南和说明。

7.20.4 应以防止污染人员或环境的方式运输危险材料，并有可靠的安保措施。

7.20.5 危险材料应置于被批准的本质安全的防漏容器中运输。

7.20.6 国际和国家关于道路、铁路、水路和航空运输危险材料的公约、法规和标准适用，应按国家或国际现行的规定和标准，包装、标示所运输的物品并提供文件资料。

7.21 应急措施

7.21.1 应制定应急措施的政策和程序，包括生物性、化学性、物理性、放射性等紧急情况和火灾、水灾、冰冻、地震、人为破坏等任何意外紧急情况，还应包括使留下的空建筑物处于尽可能安全状态的措施，应征询相关主管部门的意见和建议。

7.21.2 应急程序应至少包括负责人、组织、应急通讯、报告内容、个体防护和应对程序、应急设备、撤离计划和路线、污染源隔离和消毒灭菌、人员隔离和救治、现场隔离和控制、风险沟通等内容。

7.21.3 实验室应负责使所有人员（包括来访者）熟悉应急行动计划、撤离路线和紧急撤离的集合地点。

7.21.4 每年应至少组织所有实验室人员进行一次演习。

7.22 消防安全

7.22.1 虚有消防相关的政策和程序，并使所有人员理解，以确保人员安全和防止实验室内的危险扩散。

7.22.2 应制定年度消防计划，内容至少包括（不限于）：

a) 对实验室人员的消防指导和培训，内容至少包括火险的识别和判断、减少火险的良好操作规程、失火时应采取的全部行动；

b) 实验室消防设施设备和报警系统状态的检查；

c) 消防安全定期检查计划；

d) 消防演习（每年至少一次）。

7.22.3 在实验室内应尽量减少可燃气体和液体的存放量。

7.22.4 应在适用的排风罩或排风柜中操作可燃气体或液体。

7.22.5 应将可燃气体或液体放置在远离热源或打火源之处，避免阳光直射。

7.22.6 输送可燃气体或液体的管道应安装紧急关闭阀。

7.22.7 应配备控制可燃物少量泄漏的工具包。如果发生明显泄漏，应立即寻求消防部门的援助。

7.22.8 可燃气体或液体应存放在经批准的贮藏柜或库中。贮存量应符合国家相关的规定和标准。

7.22.9 需要冷藏的可燃液体应存放在防爆（无火花）的冰箱中。

7.22.10 需要时，实验室应使用防爆电器。

7.22.11 应配备适当的设备，需要时用于扑灭可控制的火情及帮助人员从火场撤离。

7.22.12 应依据实验室可能失火的类型配置适当的灭火器材并定期维护，应符合消防主管部门的要求。

7.22.13 如果发生火警，应立即寻求消防部门的援助，并告知实验室内存在的危险。

7.23 事故报告

7.23.1 实验室应有报告实验室事件、伤害、事故、职业相关疾病以及潜在危险的政策和程序，符合国家和地方对事故报告的规定要求。

7.23.2 所有事故报告应形成书面文件并存档（包括所有相关活动的记录和证据等文件）。适用时， 报告应包括事实的详细描述、原因分析、影响范围、后果评估、采取的措施、所采取措施有效性的追踪、预防类似事件发生的建议及改进措施等。

7.23.3 事故报告（包括采取的任何措施）应提交实验室管理层和安全委员会评审，适用时，还应提交更高管理层评审。

7.23.4 实验室任何人员不得隐瞒实验室活动相关的事件、伤害、事故、职业相关疾病以及潜在危险，应按国家规定上报。

1. 住房和城乡建设部：生物安全实验室建筑技术规范

1 总 则

1.0.1为使生物安全实验室在设计、施工和验收方面满足实验室生物安全防护要求，制定本规范。

1.0.2本规范适用于新建、改建和扩建的生物安全实验室的设计、施工和验收。

1.0.3生物安全实验室的建设应切实遵循物理隔离的建筑技术原则，以生物安全为核心，确保实验人员的安全和实验室周围环境的安全，并应满足实验对象对环境的要求，做到实用、经济。生物安全实验室所用设备和材料应有符合要求的合格证、检验报告，并在有效期之内。属于新开发的产品、工艺，应有鉴定证书 或试验证明材料。

1.0.4生物安全实验室的设计、施工和验收除应执行本规范的 规定外，尚应符合国家现行有关标准的规定。

2 术 语

2.0.1一级屏障primary barrier

操作者和被操作对象之间的隔离，也称一级隔离。

2.0.2二级屏障secondary barrier

生物安全实验室和外部环境的隔离，也称二级隔离。

2.0.3生物安全实验室biosafety laboratory

通过防护屏障和管理措施，达到生物安全要求的微生物实验室和动物实验室。包括主实验室及其辅助用房。

2.0.4实验室防护区laboratory containment area

是指生物风险相对较大的区域，对围护结构的严密性、气流流向等有要求的区域。

2.0.5实验室辅助工作区non-contamination zone

实验室辅助工作区指生物风险相对较小的区域，也指生物安全实验室中防护区以外的区域。

2.0.6主实验室main room

是生物安全实验室中污染风险最高的房间，包括实验操作间、动物饲养间、动物解剖间等，主实验室也称核心工作间。

2.0.7缓冲间buffer room

设置在被污染概率不同的实验室区域间的密闭室。需要时，可设置机械通风系统，其门具有互锁功能，不能同时处于开启状态。

2.0.8独立通风笼具individually ventilated cage (IVC)

一种以饲养盒为单位的独立通风的屏障设备，洁净空气分别送人各独立笼盒使饲养环境保持一定压力和洁净度，用以避免环境污染动物〔正压）或动物污染环境（负压），一切实验操作均需要在生物安全柜等设备中进行。该设备用于饲养清洁、无特定病原体或感染（负压）动物。

2.0.9动物隔离设备animal isolated equipment

是指动物生物安全实验室内饲育动物采用的隔离装置的统称。该设备的动物饲育内环境为负压和单向气流，以防止病原体外泄至环境并能有效防止动物逃逸。常用的动物隔离设备有隔离器、层流柜等。

2.0.10气密门airtight door

气密门为密闭门的一种，气密门通常具有一体化的门扇和门框，采用机械压紧装置或充气密封圈等方法密闭缝隙。

2.0.11活毒废水waste water of biohazard

被有害生物因子污染了的有害废水。

2.0.12洁净度7级cleanliness class 7

空气中大于等于0. 5;.im的尘粒数大于35200粒／m3到小于等于352000粒／m3，大于等于1¦Ìm的尘粒数大于8320粒/m3到小于等于83200粒／m3，大于等于5¦Ìm的尘粒数大于293粒/m3到小于等于2930粒/m30

2.0.13洁净度8级cleanliness Class 8

空气中大于等于0.5拌m的尘粒数大于352000粒／m3到小于等于3520000粒／m3，大于等于1拜m的尘粒数大于83200粒/m3到小于等于832000粒/m3，大于等于5拌m的尘粒数大于2930粒／m3到小于等于29300粒/m3.

2.0.14静态at-rest

实验室内的设施已经建成，工艺设备已经安装，通风空调系统和设备正常运行，但无工作人员操作且实验对象尚未进人时的状态。

2.0.15综合性能评定comprehensive performance judgment

对已竣工验收的生物安全实验室的工程技术指标进行综合检测和评定。

3生物安全实验室的分级、分类和技术指标

3.1生物安全实验室的分级

3.1.1生物安全实验室可由防护区和辅助工作区组成。

3.1.2根据实验室所处理对象的生物危害程度和采取的防护措施，生物安全实验室分为四级。微生物生物安全实验室可采用BSL-1、BSI- 2、BSL- 3、BSI-4表示相应级别的实验室；动物生物安全实验室可采用ABSL-, ABSL-2, ABSL- 3, ABSL-4表示相应级别的实验室。生物安全实验室应按表3.1.1进行分级。

3.2生物安全实验室的分类

3.2.1生物安全实验室根据所操作致病性生物因子的传播途径可分为a类和b类。a类指操作非经空气传播生物因子的实验室；b类指操作经空气传播生物因子的实验室。bl类生物安全实验室指可有效利用安全隔离装置进行操作的实验室；b2类生物安全实验室指不能有效利用安全隔离装置进行操作的实验室。

3.2.2四级生物安全实验室根据使用生物安全柜的类型和穿着防护服的不同，可分为生物安全柜型和正压服型两类，并可符合表3.2. 2的规定。

3.3生物安全实验室的技术指标

3.3.1二级生物安全实验室宜实施一级屏障和二级屏障，三级、四级生物安全实验室应实施一级屏障和二级屏障。

3.3.2生物安全主实验室二级屏障的主要技术指标应符合表3.3.2的规定。

3.3.3三级和四级生物安全实验室其他房间的主要技术指标应符合表3.3.3的规定。

3.3.4当房间处于值班运行时，在各房间压差保持不变的前提下，值班换气次数可低于本规范表3.3. 2和表3.3.3中规定的数值。

3.3.5对有特殊要求的生物安全实验室，空气洁净度级别可高于本规范表3.3.2和表3.3.3的规定，换气次数也应随之提高。

4建筑、装修和结构

4. 1建筑要求

4.1.1生物安全实验室的位置要求应符合表4.1.1的规定。

4.1.2生物安全实验室应在人口处设置更衣室或更衣柜。

4.1.3 BSL-3中a类实验室防护区应包括主实验室、缓冲间等，缓冲间可兼作防护服更换间；辅助工作区应包括清洁衣物更换间、监控室、洗消间、淋浴间等；BS卜3中bl类实验室防护区应包括主实验室、缓冲间、防护服更换间等。辅助工作区应包括清洁衣物更换间、监控室、洗消间、淋浴间等。主实验室不宜直接与其他公共区域相邻。

4.1.4 ABSI二3实验室防护区应包括主实验室、缓冲间、防护服更换间等，辅助工作区应包括清洁衣物更换间、监控室、洗消间等。

4.1.5四级生物安全实验室防护区应包括主实验室、缓冲间、外防护服更换间等，辅助工作区应包括监控室、清洁衣物更换间等；设有生命支持系统四级生物安全实验室的防护区应包括主实验室、化学淋浴间、外防护服更换间等．化学淋浴间可兼作缓

冲间。

4.1.6 ABSL-3中的b2类实验室和四级生物安全实验室宜独立于其他建筑。

4.1.7三级和四级生物安全实验室的室内净高不宜低于2. 6m,三级和四级生物安全实验室设备层净高不宜低于2.2m,

4.1.8三级和四级生物安全实验室人流路线的设置，应符合空气洁净技术关于污染控制和物理隔离的原则。

4.1.9 ABS卜4的动物尸体处理设备间和防护区污水处理设备间应设缓冲间。

4.1.10设置生命支持系统的生物安全实验室，应紧邻主实验室设化学淋浴间。

4.1.11三级和四级生物安全实验室的防护区应设置安全通道和紧急出口，并有明显的标志。

4.1.12三级和四级生物安全实验室防护区的围护结构宜远离建筑外墙，主实验室宜设置在防护区的中部。四级生物安全实验室建筑外墙不宜作为主实验室的围护结构。

4.1.13三级和四级生物安全实验室相邻区域和相邻房间之间应根据需要设置传递窗，传递窗两门应互锁，并应设有消毒灭菌装置，其结构承压力及严密性应符合所在区域的要求；当传递不能灭活的样本出防护区时，应采用具有熏蒸消毒功能的传递窗或药液传递箱。

4.1.14二级生物安全实验室应在实验室或实验室所在建筑内配备高压灭菌器或其他消毒灭菌设备；三级生物安全实验室应在防护区内设置生物安全型双扉高压灭菌器，主体一侧应有维护空间；四级生物安全实验室主实验室应设置生物安全型双扉高压灭菌器，主体所在房间应为负压。

4.1.15三级和四级生物安全实验室的生物安全柜和负压解剖台应布置于排风口附近，并应远离房间门。

4.1.16 ABSL3, ABSI二4产生大动物尸体或数量较多的小动物尸体时，宜设置动物尸体处理设备。动物尸体处理设备的投放口宜设置在产生动物尸体的区域。动物尸体处理设备的投放口宜高出地面或设置防护栏杆。

4 . 2 装修要求

4.2.1三级和四级生物安全实验室应采用无缝的防滑耐腐蚀地面，踢脚宜与墙面齐平或略缩进不大于2mm-3mm。地面与墙面的相交位置及其他围护结构的相交位置，宜作半径不小于30mm的圆弧处理。

4.2.2三级和四级生物安全实验室墙面、顶棚的材料应易于清洁消毒、耐腐蚀、不起尘、不开裂、光滑防水，表面涂层宜具有抗静电性能。

4.2.3一级生物安全实验室可设带纱窗的外窗；没有机械通风系统时，ABSL-2中的a类、bi类和BSL-2生物安全实验室可设外窗进行自然通风，且外窗应设置防虫纱窗；ABSIL-2中b2类、三级和四级生物安全实验室的防护区不应设外窗，但可在内墙上设密闭观察窗，观察窗应采用安全的材料制作。

4.2.4生物安全实验室应有防止节肢动物和啮齿动物进入和外逃的措施。

4.2.5二级、三级、四级生物安全实验室主人口的门和动物饲养间的门、放置生物安全柜实验间的门应能自动关闭，实验室门应设置观察窗，并应设置门锁。当实验室有压力要求时，实验室的门宜开向相对压力要求高的房间侧。缓冲间的门应能单向锁定。ABSL-3中b2类主实验室及其缓冲间和．四级生物安全实验室主实验室及其缓冲间应采用气密门。

4.2.6生物安全实验室的设计应充分考虑生物安全柜、动物隔

离设备、高压灭菌器、动物尸体处理设备、污水处理设备等设备的尺寸和要求，必要时应留有足够的搬运孔洞，以及设置局部隔离、防振、排热、排湿设施。

4.2.7三级和四级生物安全实验室防护区内的顶棚上不得设置检修口。

4.2.8二级、三级、四级生物安全实验室的人口，应明确标示出生物防护级别、操作的致病性生物因子、实验室负责人姓名、紧急联络方式等，并应标示出国际通用生物危险符号（图4.2.8)。生物危险符号应按图4.2.8绘制，颜色应为黑色，背景为黄色。 4 . 3 结构要求

4.3.1生物安全实验室的结构设计应符合现行国家标准《建筑结构可靠度设计统一标准》GB50068的有关规定。三级生物安全实验室的结构安全等级不宜低于一级，四级生物安全实验室的结构安全等级不应低于一级。

4.3.2生物安全实验室的抗震设计应符合现行国家标准《建筑抗震设防分类标准》GB50223的有关规定。三级生物安全实验室抗震设防类别宜按特殊设防类，四级生物安全实验室抗震设防类别应按特殊设防类。

4.3.3生物安全实验室的地基基础设计应符合现行国家标准《建筑地基基础设计规范》GB50007的有关规定。三级生物安全实验室的地基基础宜按甲级设计，四级生物安全实验室的地基基础应按甲级设计。

4.3.4三级和四级生物安全实验室的主体结构宜采用混凝土结构或砌体结构体系。

4.3.5三级和四级生物安全实验室的吊顶作为技术维修夹层时，其吊顶的活荷载不应小于0.75kN/m2，对于吊顶内特别重要的设备宜做单独的维修通道。

5空调、通风和净化

5 . 1 一般规定

5.1.1生物安全实验室空调净化系统的划分应根据操作对象的危害程度、平面布置等情况经技术经济比较后确定，并应采取有效措施避免污染和交叉污染。空调净化系统的划分应有利于实验室消毒灭菌、自动控制系统的设置和节能运行。

5.1.2生物安全实验室空调净化系统的设计应考虑各种设备的热湿负荷。

5.1.3生物安全实验室送、排风系统的设计应考虑所用生物安全柜、动物隔离设备等的使用条件。

5.1.4生物安全实验室可按表5.1.4的原则选用生物安全柜。 5.1.5二级生物安全实验室中的a类和bl类实验室可采用带循环风的空调系统。二级生物安全实验室中的b2类实验室宜采用全新风系统，防护区的排风应根据风险评估来确定是否需经高效空气过滤器过滤后排出。

5.1.6三级和四级生物安全实验室应采用全新风系统。

5.1.7三级和四级生物安全实验室主实验室的送风、排风支管和排风机前应安装耐腐蚀的密闭阀，阀门严密性应与所在管道严密性要求相适应。

5.1.8三级和四级生物安全实验室防护区内不应安装普通的风机盘管机组或房间空调器。

5.1.，三级和四级生物安全实验室防护区应能对排风高效空气过滤器进行原位消毒和检漏。四级生物安全实验室防护区应能对送风高效空气过滤器进行原位消毒和检漏。

5.1.10生物安全实验室的防护区宜临近空调机房。

5.1.11生物安全实验室空调净化系统和高效排风系统所用风机应选用风压变化较大时风量变化较小的类型。

5 . 2 送风系统

5.2.1空气净化系统至少应设置粗、中、高三级空气过滤，并应符合下列规定：

1第一级是粗效过滤器，全新风系统的粗效过滤器可设在空调箱内，对于带回风的空调系统，粗效过滤器宜设置在新风口或紧靠新风口处。

第二级是中效过滤器，宜设置在空气处理机组的正压段。

第三级是高效过滤器，应设置在系统的末端或紧靠末端，不应设在空调箱内。

4全新风系统宜在表冷器前设置一道保护用的中效过滤器。

5.2.2送风系统新风口的设置应符合下列规定：

1新风口应采取有效的防雨措施。

2新风口处应安装防鼠、防昆虫、阻挡绒毛等的保护网，且易于拆装。

3新风口应高于室外地面2. 5m以上，并应远离污染源。

5.3．排风系统

5.3.1三级和四级生物安全实验室排风系统的设置应符合下列规定：

1排风必须与送风连锁，排风先于送风开启，后于送风关闭。

2主实验室必须设置室内排风口，不得只利用生物安全柜或其他负压隔离装置作为房间排风出口。

3 bl类实验室中可能产生污染物外泄的设备必须设置带高效空气过滤器的局部负压排风装置，负压排风装置应具有原位检漏功能。

4不同级别、种类生物安全柜与排风系统的连接方式应按表5.3. 1选用。

5动物隔离设备与排风系统的连接应采用密闭连接或设置局部排风罩。

6排风机应设平衡基座，并应采取有效的减振降噪措施。 5.3.2三级和四级生物安全实验室防护区的排风必须经过高效过滤器过滤后排放。

5.3.3三级和四级生物安全实验室排风高效过滤器宜设置在室内排风口处或紧邻排风口处，三级生物安全实验室防护区有特殊要求时可设两道高效过滤器。四级生物安全实验室防护区除在室内排风口处设第一道高效过滤器外，还应在其后串联第二道高效过滤器。防护区高效过滤器的位置与排风口结构应易于对过滤器进行安全更换和检漏。

5.3.4三级和四级生物安全实验室防护区排风管道的正压段不应穿越房间，排风机宜设置于室外排风口附近。

5.3.5三级和四级生物安全实验室防护区应设置备用排风机，备用排风机应能自动切换．切换过程中应能保持有序的压力梯度和定向流。

5.3.6三级和四级生物安全实验室应有能够调节排风或送风以维持室内压力和压差梯度稳定的措施。

5.3.7三级和四级生物安全实验室防护区室外排风口应设置在主导风的下风向，与新风口的直线距离应大于12m，并应高于所在建筑物屋面2m以上。三级生物安全实验室防护区室外排风口与周围建筑的水平距离不应小于20m,

5.3.8 ABS卜4的动物尸体处理设备间和防护区污水处理设备间的排风应经过高效过滤器过滤。 5 . 4 气流组织

5.4.1三级和四级生物安全实验室各区之间的气流方向应保证由辅助工作区流向防护区，辅助工作区与室外之间宜设一间正压缓冲室。

5.4.2三级和四级生物安全实验室内各种设备的位置应有利于气流由被污染风险低的空间向被污染风险高的空间流动，最大限度减少室内回流与涡流。

5.4.3生物安全实验室气流组织宜采用上送下排方式，送风口和排风口布置应有利于室内可能被污染空气的排出。饲养大动物生物安全实验室的气流组织可采用上送上排方式。

5.4.4在生物安全柜操作面或其他有气溶胶产生地点的上方附近不应设送风口。

5.4.5高效过滤器排风口应设在室内被污染风险最高的区域，不应有障碍。

5.4.6气流组织上送下排时，高效过滤器排风口下边沿离地面不宜低于。.lm，且不宜高于0. 15m；上边沿高度不宜超过地面之上0. 6m。排风口排风速度不宜大于lm/s。

5.5空调净化系统的部件与材料

5.5.1送、排风高效过滤器均不得使用木制框架。三级和四级生物安全实验室防护区的高效过滤器应耐消毒气体的侵蚀，防护区内淋浴间、化学淋浴间的高效过滤器应防潮。三级和四级生物安全实验室高效过滤器的效率不应低于现行国家标准《高效空气过滤器》GB/T13554中的B类。

5.5.2要消毒的通风管道应采用耐腐蚀、耐老化、不吸水、易消毒灭菌的材料制作，并应为整体焊接。

5.5.3排风机外侧的排风管上室外排风口处应安装保护网和防雨罩

5.5.4空调设备的选用应满足下列要求：

1.不应采用淋水式空气处理机组。当采用表面冷却器时，通过盘管所在截面的气流速度不宜大于2.Om/s,

2各级空气过滤器前后应安装压差计，测量接管应通畅，安装严密。

3宜选用干蒸汽加湿器。

4加湿设备与其后的过滤段之间应有足够的距离。

5在空调机组内保持1000Pa的静压值时，箱体漏风率不 应大于2%.

6消声器或消声部件的材料应能耐腐蚀、不产尘和不易附着灰尘

7 送、排风系统中的中效、高效过滤器不应重复使用。

6给水排水与气体供应

6 . 1 一般规定

6.1.1生物安全实验室的给水排水干管、气体管道的干管，应敷设在技术夹层内。生物安全实验室防护区应少敷设管道，与本区域无关管道不应穿越。引人三级和四级生物安全实验室防护区内的管道宜明敷。

6.1.2给水排水管道穿越生物安全实验室防护区围护结构处应设可靠的密封装置，密封装置的严密性应能满足所在区域的严密性要求。

6.1.3进出生物安全实验室防护区的给水排水和气体管道系统应不渗漏、耐压、耐温、耐腐蚀。实验室内应有足够的清洁、维护和维修明露管道的空间。

6.1.4生物安全实验室使用的高压气体或可燃气体，应有相应的安全措施。

6.1.5化学淋浴系统中的化学药剂加压泵应一用一备，并应设置紧急化学淋浴设备，在紧急情况下或设备发生故障时使用。 6 . 2 给 水

6.2.1生物安全实验室防护区的给水管道应采取设置倒流防止器或其他有效的防止回流污染的装置，并且这些装置应设置在辅助工作区。

6.2.2 ABSL3和四级生物安全实验室宜设置断流水箱，水箱容积宜按一天的用水量进行计算。

6.2.3三级和四级生物安全实验室防护区的给水管路应以主实验室为单元设置检修阀门和止回阀。

6.2.4一级和二级生物安全实验室应设洗手装置，并宜设置在靠近实验室的出口处。三级和四级生物安全实验室的洗手装置应设置在主实验室出口处，对于用水的洗手装置的供水应采用非手动开关。

6.2.5二级、三级和四级生物安全实验室应设紧急冲眼装置。一级生物安全实验室内操作刺激或腐蚀性物质时，应在30m内设紧急冲眼装置，必要时应设紧急淋浴装置。

6.2.6 ABSL-3和四级生物安全实验室防护区的淋浴间应根据工艺要求设置强制淋浴装置。

6.2.7大动物生物安全实验室和需要对笼具、架进行冲洗的动物实验室应设必要的冲洗设备。

6.2.8三级和四级生物安全实验室的给水管路应涂上区别于一般水管的醒目的颜色。

6.2.9室内给水管材宜采用不锈钢管、铜管或无毒塑料管等，管道应可靠连接。 6 . 3 排 水

6. 3. 1三级和四级生物安全实验室可在防护区内有排水功能要求的地面设置地漏，其他地方不宜设地漏。大动物房和解剖间等处的密闭型地漏内应带活动网框，活动网框应易于取放及

清理。

6.3.2三级和四级生物安全实验室防护区应根据压差要求设置存水弯和地漏的水封深度；构造内无存水弯的卫生器具与排水管道连接时，必须在排水口以下设存水弯；排水管道水封处必须保证充满水或消毒液。

6.3.3三级和四级生物安全实验室防护区的排水应进行消毒灭菌处理。

6.3.4三级和四级生物安全实验室的主实验室应设独立的排水支管，并应安装阀门。

6.3.5活毒废水处理设备宜设在最低处，便于污水收集和检修。

6.3.6 ABSL-2防护区污水的处理装置可采用化学消毒或高温灭菌方式。三级和四级生物安全实验室防护区活毒废水的处理装置应采用高温灭菌方式。应在适当位置预留采样口和采样操作空间。

6.3.7生物安全实验室防护区排水系统上的通气管口应单独设置，不应接人空调通风系统的排风管道。三级和四级生物安全实验室防护区通气管口应设高效过滤器或其他可靠的消毒装置，同时应使通气管口四周的通风良好。

6.3.8三级和四级生物安全实验室辅助工作区的排水，应进行监测．并应采取适当处理措施，以确保排放到市政管网之前达到排放要求。

6.3.9三级和四级生物安全实验室防护区排水管线宜明设，并与墙壁保持一定距离便于检查维修。

6.3.10三级和四级生物安全实验室防护区的排水管道宜采用不锈钢或其他合适的管材、管件。排水管材、管件应满足强度、温度、耐腐蚀等性能要求。

6.3.11四级生物安全实验室双扉高压灭菌器的排水应接人防护区废水排放系统。 6 . 4 气体供应

6.4.1生物安全实验室的专用气体宜由高压气瓶供给，气瓶宜设置于辅助工作区，通过管道输送到各个用气点，并应对供气系统进行监测。

6.4.2所有供气管穿越防护区处应安装防回流装置，用气点应根据工艺要求设置过滤器。

6.4.3三级和四级生物安全实验室防护区设置的真空装置，应有防止真空装置内部被污染的措施；应将真空装置安装在实验室内。

6.4.4正压服型生物安全实验室应同时配备紧急支援气罐，紧急支援气罐的供气时间不应少于60min/人。

6.4.5供操作人员呼吸使用的气体的压力、流量、含氧量、温度、湿度、有害物质的含量等应符合职业安全的要求。

6.4.6充气式气密门的压缩空气供应系统的压缩机应备用，并应保证供气压力和稳定性符合气密门供气要求。

7 电 气

7 . 1 配 电

7.1.1生物安全实验室应保证用电的可靠性。二级生物安全实验室的用电负荷不宜低于二级。

7.1.2 BSL-3实验室和ABSL-3中的a类和bl类实验室应按一级负荷供电，当按一级负荷供电有困难时．应采用一个独立供电电源．且特别重要负荷应设置应急电源；应急电源采用不间断电源的方式时，不间断电源的供电时间不应小于30min；应急电源采用不间断电源加自备发电机的方式时，不间断电源应能确保自备发电设备启动前的电力供应。

7.1.3 A]略L-3中的b2类实验室和四级生物安全实验室必须按一级负荷供电，特别重要负荷应同时设置不间断电源和自备发电设备．作为应急电源．不间断电源应能确保自备发电设备启动前的电力供应。

7.1.4生物安全实验室应设专用配电箱。三级和四级生物安全实验室的专用配电箱应设在该实验室的防护区外。

7.1.5生物安全实验室内应设置足够数量的固定电源插座，重要设备应单独回路配电，且应设置漏电保护装置。

7.1.6管线密封措施应满足生物安全实验室严密性要求。三级和四级生物安全实验室配电管线应采用金属管敷设，穿过墙和楼板的电线管应加套管或采用专用电缆穿墙装置，套管内用不收缩、不燃材料密封。

7 . 2 照 明

7.2.1三级和四级生物安全实验室室内照明灯具宜采用吸顶式密闭洁净灯，并宜具有防水功能。

7.2.2三级和四级生物安全实验室应设置不少于30min的应急照明及紧急发光疏散指示标志。

7.2.3三级和四级生物安全实验室的人口和主实验室缓冲间人口处应设置主实验室工作状态的显示装置。 7 . 3 自动控制

7.3.1空调净化自动控制系统应能保证各房间之间定向流方向的正确及压差的稳定。

7.3.2三级和四级生物安全实验室的自控系统应具有压力梯度、温湿度、连锁控制、报警等参数的历史数据存储显示功能，自控系统控制箱应设于防护区外。

7.3.3三级和四级生物安全实验室自控系统报，信号应分为重要参数报苷和一般参数报替。重要参数报替应为声光报普和显示报替，一般参数报，应为显示报警。三级和四级生物安全实验室应在主实验室内设置紧急报借按钮。

7.3.4三级和四级生物安全实验室应在有负压控制要求的房间人口的显著位置，安装显示房间负压状况的压力显示装置。

7.3.5自控系统应预留接口。

7.3.6三级和四级生物安全实验室空调净化系统启动和停机过程应采取措施防止实验室内负压值超出围护结构和有关设备的安全范围。

7.3.7三级和四级生物安全实验室防护区的送风机和排风机应设置保护装置，并应将保护装置报警信号接人控制系统。

7.3.8三级和四级生物安全实验室防护区的送风机和排风机宜设置风压差检测装置，当压差低于正常值时发出声光报警。

7.3.，三级和四级生物安全实验室防护区应设送排风系统正常运转的标志，当排风系统运转不正常时应能报警。备用排风机组应能自动投人运行，同时应发出报警信号。

7.3.10三级和四级生物安全实验室防护区的送风和排风系统必须可靠连锁，空调通风系统开机顺序应符合本规范第5.3. 1条的要求。

7.3.11当空调机组设置电加热装置时应设置送风机有风检测装置，并在电加热段设置监测温度的传感器，有风信号及温度信号应与电加热连锁。

7.3.12三级和四级生物安全实验室的空调通风设备应能自动和手动控制，应急手动应有优先控制权，且应具备硬件连锁功能。

7.3.13四级生物安全实验室防护区室内外压差传感器采样管应配备与排风高效过滤器过滤效率相当的过滤装置。

7.3.14三级和四级生物安全实验室应设置监测送风、排风高效过滤器阻力的压差传感器。

7.3.15在空调通风系统未运行时，防护区送风、排风管上的密闭阀应处于常闭状态。 7 . 4 安全防范

7.4.1四级生物安全实验室的建筑周围应设置安防系统。三级和四级生物安全实验室应设门禁控制系统。

7.4.2三级和四级生物安全实验室防护区内的缓冲间、化学淋浴间等房间的门应采取互锁措施。

7.4.3三级和四级生物安全实验室应在互锁门附近设置紧急手动解除互锁开关。中控系统应具有解除所有门或指定门互锁的功能。

7.4.4三级和四级生物安全实验室应设闭路电视监视系统。

7.4.5生物安全实验室的关键部位应设置监视器，需要时，可实时监视并录制生物安全实验室活动情况和生物安全实验室周围J清况。监视设备应有足够的分辨率，影像存储介质应有足够的数据存储容量。

7 . 5 通 信

7.5.1三级和四级生物安全实验室防护区内应设置必要的通信设备。

7.5.2三级和四级生物安全实验室内与实验室外应有内部电话或对讲系统。安装对讲系统时，宜采用向内通话受控、向外通话非受控的选择性通话方式。

8 消 防

8.0.1二级生物安全实验室的耐火等级不宜低于二级。

8.0.2三级生物安全实验室的耐火等级不应低于二级。四级生物安全实验室的耐火等级应为一级。

8.0.3四级生物安全实验室应为独立防火分区。三级和四级生物安全实验室共用一个防火分区时，其耐火等级应为一级。

8.0.4生物安全实验室的所有疏散出口都应有消防疏散指示标志和消防应急照明措施。

8.0.5三级和四级生物安全实验室吊顶材料的燃烧性能和耐火极限不应低于所在区域隔墙的要求。三级和四级生物安全实验室与其他部位隔开的防火门应为甲级防火门。

8.0.6生物安全实验室应设置火灾自动报警装置和合适的灭火器材。

8.0.7三级和四级生物安全实验室防护区不应设置自动喷水灭火系统和机械排烟系统，但应根据需要采取其他灭火措施。

8.0.8独立于其他建筑的三级和四级生物安全实验室的送风、排风系统可不设置防火阀。

8.0.9三级和四级生物安全实验室的防火设计应以保证人员能尽快安全疏散、防止病原微生物扩散为原则，火灾必须能从实验室的外部进行控制，使之不会蔓延。

9 施 工 要 求

9 . 1 一 般 规 定

9.1.1生物安全实验室的施工应以生物安全防护为核心。三级和四级生物安全实验室施工应同时满足洁净室施工要求。

9.1.2生物安全实验室施工应编制施工方案。

9.1.3各道施工程序均应进行记录，验收合格后方可进行下道工序施工。

9.1.4施工安装完成后，应进行单机试运转和系统的联合试运转及调试，作好调试记录，并应编写调试报告。

9 . 2 筑 装 修

9.2.1建筑装修施工应做到墙面平滑、地面平整、不易附着灰尘。

9.2.2三级和四级生物安全实验室围护结构表面的所有缝隙应采取可靠的措施密封。

9.2.3三级和四级生物安全实验室有压差梯度要求的房间应在合适位置设测压孔，平时应有密封措施。

9.2.4生物安全实验室中各种台、架、设备应采取防倾倒措施，相互之间应保持一定距离。当靠地靠墙放置时，应用密封胶将靠地靠墙的边缝密封。

9.2.5气密门宜直接与土建墙连接固定，与强度较差的围护结构连接固定时，应在围护结构上安装加强构件。

9.2.6气密门两侧、顶部与围护结构的距离不宜小于200mm,

9.2.7气密门门体和门框宜采用整体焊接结构，门体开闭机构宜设置有可调的铰链和锁扣。

9 . 3 空调净化

9.3.1空调机组的基础对地面的高度不宜低于200mm.

9.3.2空调机组安装时应调平，并作减振处理。各检查门应平整．密封条应严密。正压段的门宜向内开，负压段的门宜向外开。表冷段的冷凝水排水管上应设置水封和阀门。

9.3.3送、排风管道的材料应符合设计要求，加工前应进行清洁处理，去掉表面油污和灰尘。

9.3.4风管加工完毕后，应擦拭干净，并应采用薄膜把两端封住，安装前不得去掉或损坏。

9.3.5技术夹层里的任何管道和设备穿过防护区时，贯穿部位应可靠密封。灯具箱与吊顶之间的孔洞应密封不漏。

9.3.6送、排风管道宜隐蔽安装。

9.3.7送、排风管道咬口连接的咬口缝均应用胶密封。

9.3.8各类调节装置应严密，调节灵活，操作方便。

9.3.9三级和四级生物安全实验室的排风高效过滤装置，应符合国家现行有关标准的规定，直到现场安装时方可打开包装。排风高效过滤装置的室内侧应有保护高效过滤器的措施。

9.4 实验室设备

9.4.1生物安全柜、负压解剖台等设备在搬运过程中，不应横倒放置和拆卸，宜在搬人安装现场后拆开包装。

9.4.2生物安全柜和负压解剖台背面、侧面与墙的距离不宜小于300mm，顶部与吊顶的距离不应小于300mm,

9.4.3传递窗、双扉高压灭菌器、化学淋浴间等设施与实验室围护结构连接时，应保证箱体的严密性。

9.4.4传递窗、双扉高压灭菌器等设备与轻体墙连接时，应在连接部位采取加固措施。

9.4.5三级和四级生物安全实验室防护区内的传递窗和药液传递箱的腔体或门扇应整体焊接成型。

9.4.6具有熏蒸消毒功能的传递窗和药液传递箱的内表面不应使用有机材料。

9.4.7生物安全实验室内配备的实验台面应光滑、不透水、耐腐蚀、耐热和易于清洗。

9.4.8生物安全实验室的实验台、架、设备的边角应以圆弧过渡，不应有突出的尖角、锐边、沟槽。

10检测和验收

10. 1工程检测

10.1.1三级和四级生物安全实验室工程应进行工程综合性能全面检测和评定，并应在施工单位对整个工程进行调整和测试后进行。对于压差、洁净度等环境参数有严格要求的二级生物安全实验室也应进行综合性能全面检测和评定。

10.1.2有下列情况之一时，应对生物安全实验室进行综合性能全面检测并按本规范附录A进行记录：

1竣工后，投人使用前。

2停止使用半年以上重新投人使用。

3进行大修或更换高效过滤器后。

4一年一度的常规检测。

10.1.3有生物安全柜、隔离设备等的实验室，首先应进行生物安全柜、动物隔离设备等的现场检测，确认性能符合要求后方可进行实验室性能的检测。

10.1.4检测前应对全部送、排风管道的严密性进行确认。对于b2类的三级生物安全实验室和四级生物安全实验室的通风空调系统，应根据对不同管段和设备的要求，按现行国家标准《洁净室施工及验收规范》GB50591的方法和规定进行严密性试验。

10.1.5三级和四级生物安全实验室工程静态检测的必测项目应按表10. 1.5的规定进行。 10.1.6围护结构的严密性检测和评价应符合下列规定：

1围护结构严密性检测方法应按现行国家标准《洁净室施工及验收规范》GB50591和《实验室生物安全通用要求》GB19489的有关规定进行，围护结构的严密性应符合本规范表3.3.2的要求。

2 ABSL-3中b2类的主实验室应采用恒压法检测。

3四级生物安全实验室的主实验室应采用压力衰减法检测，有条件的进行正、负压两种工况的检测。 4对于BSL-3和ABSL3中a类、bl类实验室可采用目测及烟雾法检测。

10.1.7排风高效过滤器检漏的检测和评价应符合下列规定：

1对于三级和四级生物安全实验室防护区内使用的所有排风高效过滤器应进行原位扫描法检漏。检漏用气溶胶可采用大气尘或人工尘，检漏采用的仪器包括粒子计数器或光度计。

2对于既有实验室以及异型高效过滤器，现场确实无法扫描时，可进行高效过滤器效率法检漏。

3检漏时应同时检测并记录过滤器风量，风量不应低于实际正常运行工况下的风量。

4采用大气尘以及粒子计数器对排风过滤器直接扫描检漏时，过滤器上游粒径大于或等于0. 5拌m的含尘浓度不应小于4000pc/IJ，可采用的方法包括开启实验室各房门，保证实验室与室外相通，并关闭送风，只开排风，或关闭送排风系统，局部采用正压检漏风机。此时对于第一道过滤器，超过3pc/L，即判断为泄漏。具体方法应符合现行国家标准《洁净室施工及验收规范）))GB50591的有关规定。

5当大气尘浓度不能满足要求时，可采用人工尘，过滤器上游采用人工尘作为检漏气溶胶时，应采取措施保证过滤器上游人工尘气溶胶的均匀和稳定，并应进行验证，具体验证方法应符

合本规范附录D的规定。 6采用人工尘光度计扫描法检漏时，应按现行国家标准《洁净室施工及验收规范》GB50591的有关规定执行。且当采样探头对准被测过滤器出风面某一点静止检测时，测得透过率高于

0.01%，即认为该点为漏点。

7进行高效过滤器效率法检漏时，在过滤器上游引人人工尘，在下游进行测试，过滤器下游采样点所处断面应实现气溶胶均匀混合，过滤效率不应低于”.9900。具体方法应符合本规范

附录D的规定。 10.1.8送风高效过滤器检漏的检测和评价应符合下列规定：

三级生物安全实验中的b2类实验室和四级生物安全实验室所有防护区内使用的送风高效过滤器应进行原位检漏，其余类型实验室的送风高效过滤器采用抽检。

2检漏方法和评价标准应符合现行国家标准《洁净室施工及验收规范》GB50591的有关规定，并宜采用大气尘和粒子计数器直接扫描法。

10.1.9气流方向检测和评价应符合下列规定：

1可采用目测法，在关键位置采用单丝线或用发烟装置测定气流流向。

2评价标准：气流流向应符合本规范第5.4.2条的要求。

10.1.10静压差、送风量、洁净度级别、温度、相对湿度、噪声、照度等室内环境参数的检测方法和要求应符合现行国家标准《洁净室施工及验收规范》GB50591的有关规定。

10.1.11在生物安全实验室防护区使用的排风高效过滤器单元的严密性应符合现行国家标准《实验室生物安全通用要求》GB19489的有关规定，并应采用压力衰减法进行检测。

10. 1.12生物安全实验室应进行工况验证检测，有多个运行工况时，应分别对每个工况进行工程检测，并应验证工况转换时系统的安全性，除此之外还包括系统启停、备用机组切换、备用电源切换以及电气、自控和故障报警系统的可靠性验证。

10.1.13竣工验收的检测可由施工单位完成，但不得以竣工验收阶段的调整测试结果代替综合性能全面评定。

10.1.14三级和四级生物安全实验室投人使用后，应按本章要求进行每年例行的常规检测。 10.2生物安全设备的现场检测

10.2.1需要现场进行安装调试的生物安全设备包括生物安全柜、动物隔离设备、Ivc、负压解剖台等。有下列情况之一时，应对该设备进行现场检测并按本规范附录B进行记录：

1生物安全实验室竣工后，投人使用前，生物安全柜、动物隔离设备等已安装完毕。

2生物安全柜、动物隔离设备等被移动位置后。

3生物安全柜、动物隔离设备等进行检修后。

4生物安全柜、动物隔离设备等更换高效过滤器后。

5生物安全柜、动物隔离设备等一年一度的常规检测。 10.2.2新安装的生物安全柜、动物隔离设备等，应具有合格的出厂检测报告，并应现场检测合格且出具检测报告后才可使用。

10.2.3生物安全柜、动物隔离设备等的现场检测项目应符合表10.2.3的要求，其中第1项～5项中有一项不合格的不应使用。对现场具备检测条件的、从事高风险操作的生物安全柜和动物隔离设备应进行高效过滤器的检漏，检漏方法应按生物安全实验室高效过滤器的检漏方法执行。 10.2.4垂直气流平均风速检测应符合下列规定：

检测方法：对于II级生物安全柜等具备单向流的设备，在送风高效过滤器以下0. 15m处的截面上，采用风速仪均匀布点测量截面风速。测点间距不大于0. 15m，侧面距离侧壁不大于

0. lm，每列至少测量3点，每行至少测量5点。评价标准：平均风速不低于产品标准要求。

10.2.5工作窗口的气流流向检测应符合下列规定：检测方法：可采用发烟法或丝线法在工作窗口断面检测，检测位置包括工作窗口的四周边缘和中间区域。评价标准：工作窗口断面所有位置的气流均明显向内，无外逸，且从工作窗口吸人的气流应直接吸人窗口外侧下部的导流格栅内，无气流穿越工作区。

10.2.6工作窗口的气流平均风速检测应符合下列规定：检测方法：1风量罩直接检测法：采用风量罩测出工作窗口风量，再计算出气流平均风速。2风速仪直接检测法：宜在工作窗口外接等尺寸辅助风管，用风速仪测量辅助风管断面风速，或采用风速仪直接测量工作窗口断面风速，采用风速仪直接测量时，每列至少测量3点，至少测量5列，每列间距不大于0.15m. 3风速仪间接检测法：将工作窗口高度调整为8cm高，在窗口中间高度均匀布点，每点间距不大于0.15m，计算工作窗口风量，计算出工作窗口正常高度（通常为20cm或25cm）下

的平均风速。评价标准：工作窗口断面上的平均风速值不低于产品标准要求。

10.2.7工作区洁净度检测应符合下列规定：

检测方法：采用粒子计数器在工作区检测。粒子计数器的采样口置于工作台面向上0. 2m高度位置对角线布置，至少测量5点。

评价标准：工作区洁净度应达到5级。 10.2.8噪声检测应符合下列规定：

检测方法：对于生物安全柜、动物隔离设备等应在前面板中

心向外0. 3m，地面以上1. lm处用声级计测量噪声。对于必须

和实验室通风系统同时开启的生物安全柜和动物隔离设备等，有

条件的，应检测实验室通风系统的背景噪声，必要时进行检测值

修正。

评价标准：噪声不应高于产品标准要求。

10.2.，照度检测应符合下列规定：

检测方法：沿工作台面长度方向中心线每隔0. 3m设置一个测量点。与内壁表面距离小于0.15m时，不再设置测点。

评价标准：平均照度不低于产品标准要求。

10.2.10高效过滤器的检漏应符合下列规定：

检测方法：在高效过滤器上游引人大气尘或发人工尘，在过滤器下游采用光度计或粒子计数器进行检漏，具备扫描检漏条件的，应进行扫描检漏，无法扫描检漏的，应检测高效过滤器

效率。

评价标准：对于采用扫描检漏高效过滤器的评价标准同生物安全实验室高效过滤器的检漏；对子不能进行扫描检漏，而采用检测高效过滤器过滤效率的，其整体透过率不应超过0.005%.

10.2.11l级生物安全柜和动物隔离设备等非单向流送风设备的送风量检测应符合下列规定：

检测方法：在送风高效过滤器出风面10cm-15cm处或在进风口处测风速，计算风量。

评价标准：不低于产品设计值。

10.2.12 1[级生物安全柜和动物隔离设备箱体静压差检测应符合下列规定：

检测方法：测量正常运转状态下，箱体对所在实验室的相对负压。

评价标准：不低于产品设计值。

10.2.13工级生物安全柜和动物隔离设备严密性检测应符合下列规定：

检测方法：采用压力衰减法，将箱体抽真空或打正压，观察一定时间内的压差衰减，记录温度和大气压变化，计算衰减率。

评价标准：严密性不低于产品设计值。

10.2.14 I级生物安全柜、动物隔离设备、手套箱式解剖台的手套口风速检测应符合下列规定：

检测方法：人为摘除一只手套，在手套口中心检测风速。

评价标准：手套口中心风速不低于0. 7m/s.

10.2.15生物安全柜在有条件时，宜在现场进行箱体的漏泄检测，生物安全柜漏电检测，接地电阻检测。

10.2.16生物安全柜的安装位置应符合本规范第9.4.2条中的

相关要求。

10.2.17有下列情况之一时，需要对活毒废水处理设备、高压灭菌锅、动物尸体处理设备等进行检测。

1实验室竣工后，投人使用前，设备安装完毕。

2设备经过检修后。

3设备更换阀门、安全阀后。

4设备年度常规检测。

10.2.18活毒废水处理设备、高压灭菌锅、动物尸体处理设备

等带有高效过滤器的设备应进行高效过滤器的检漏，且检测方法应符合本规范第10.1.7条的规定。

10.2.19活毒废水处理设备、动物尸体处理设备等产生活毒废水的设备应进行活毒废水消毒灭菌效果的验证。

10.2.20活毒废水处理设备、高压灭菌锅、动物尸体处理设备等产生固体污染物的设备应进行固体污染物消毒灭菌效果的验证。

1 0 . 3工程验收

10.3.1生物安全实验室的工程验收是实验室启用验收的基础，根据国家相关规定，生物安全实验室须由建筑主管部门进行工程验收合格，再进行实验室认可验收，生物安全实验室工程验收评价项目应符合附录C的规定。

10.3.2工程验收的内容应包括建设与设计文件、施工文件和综合性能的评定文件等。

10.3.3在工程验收前，应首先委托有资质的工程质检部门进行工程检测。

10.3.4工程验收应出具工程验收报告。生物安全实验室应按本规范附录C规定的验收项目逐项验收，并应根据下列规定作出

1. 国家卫生健康委员会：国家卫生计生委关于发布《病原微生物实验室生物安全通用准则》等5项卫生行业标准的通告

1 范围

本标准规定了病原微生物实验室生物安全防护的基本原则、分级和基本要求。

本标准适用于开展微生物相关的研究、教学、检测、诊断等活动实验室。

2 术语与定义

下列术语和定义适用于本文件。

2.1

实验室生物安全 laboratory biosafety

实验室的生物安全条件和状态不低于容许水平，可避免实验室人员、来访人员、社区及环境受到不可接受的损害，符合相关法规、标准等对实验室生物安全责任的要求。

2.2

风险 risk

危险发生的概率及其后果严重性的综合。

2.3

风险评估 risk assessment

评估风险大小以及确定是否可接受的全过程。

2.4

风险控制 risk control

为降低风险而采取的综合措施。

2.5

个体防护装备 personal protective equipment； PPE

防止人员个体受到生物性、化学性或物理性等危险因子伤害的器材和用品。

2.6

生物安全柜 biosafety cabinet； BSC

具备气流控制及高效空气过滤装置的操作柜，可有效降低病原微生物或生物实验过程中产生的有害气溶胶对操作者和环境的危害。

2.7

气溶胶 aerosols

悬浮于气体介质中的粒径一般为0.001 μm～100 μm的固态或液态微小粒子形成的相对稳定的分散体系。

2.8

生物安全实验室 biosafety laboratory

通过防护屏障和管理措施，达到生物安全要求的病原微生物实验室。

2.9

实验室防护区 laboratory containment area

实验室的物理分区，该区域内生物风险相对较大，需对实验室的平面设计、围护结构的密闭性、气流，以及人员进入、个体防护等进行控制的区域。

2.10

实验室辅助工作区 non-contamination zone

是指生物风险相对较小的区域，也指生物安全实验室中防护区以外的区域。

2.11

核心工作间 core area

是生物安全实验室中开展实验室活动的主要区域，通常是指生物安全柜或动物饲养和操作间所在的房间。

2.12

加强型生物安全二级实验室 enhanced biosafety level 2 laboratory

在普通型生物安全二级实验室的基础上，通过机械通风系统等措施加强实验室生物安全防护要求的实验室。

2.13

事故 accident

造成人员及动物感染、伤害、死亡， 或设施设备损坏， 以及其他损失的意外情况。

2.14

事件 incident

导致或可能导致事故的情况。

2.15

高效空气过滤器（HEPA 过滤器） high efficiency particulate air filter

通常以0.3 μm微粒为测试物，在规定的条件下滤除效率高于99.97%的空气过滤器。

2.16

气锁 air lock

具备机械送排风系统、整体消毒灭菌条件、化学喷淋（适用时）和压力可监控的气密室，其门具有互锁功能，不能同时处于开启状态。

3 病原微生物危害程度分类

根据病原微生物的传染性、感染后对个体或者群体的危害程度，将病原微生物分为四类：

a) 第一类病原微生物，是指能够引起人类或者动物非常严重疾病的微生物，以及我国尚未发现或者已经宣布消灭的微生物；

b) 第二类病原微生物，是指能够引起人类或者动物严重疾病，比较容易直接或者间接在人与人、动物与人、动物与动物间传播的微生物；

c) 第三类病原微生物，是指能够引起人类或者动物疾病，但一般情况下对人、动物或者环境不构成严重危害，传播风险有限，实验室感染后很少引起严重疾病，并且具备有效治疗和预防措施的微生物；

d) 第四类病原微生物，是指在通常情况下不会引起人类或者动物疾病的微生物。

注1： 第一类、第二类病原微生物统称为高致病性病原微生物。

4 实验室生物安全防护水平分级与分类

4.1 分级

4.1.1 根据实验室对病原微生物的生物安全防护水平，并依照实验室生物安全国家标准的规定，将实验室分为一级（ Biosafety Level 1,BSL-1）、二级（ BSL-2）、三级（ BSL-3）、四级（ BSL-4）。

4.1.2 生物安全防护水平为一级的实验室适用于操作在通常情况下不会引起人类或者动物疾病的微生物。

4.1.3 生物安全防护水平为二级的实验室适用于操作能够引起人类或者动物疾病，但一般情况下对人、动物或者环境不构成严重危害，传播风险有限，实验室感染后很少引起严重疾病，并且具备有效治疗和预防措施的微生物。 按照实验室是否具备机械通风系统，将 BSL-2 实验室分为普通型 BSL-2 实验室、加强型 BSL-2 实验室。

4.1.4 生物安全防护水平为三级的实验室适用于操作能够引起人类或者动物严重疾病，比较容易直接或者间接在人与人、动物与人、动物与动物间传播的微生物。

4.1.5 生物安全防护水平为四级的实验室适用于操作能够引起人类或者动物非常严重疾病的微生物，我国尚未发现或者已经宣布消灭的微生物。

4.2 分类

4.2.1 以 BSL-1、 BSL-2、 BSL-3、 BSL-4 表示仅从事体外操作的实验室的相应生物安全防护水平。

4.2.2 以 ABSL-1（ Animal Biosafety Level 1, ABSL-1） 、 ABSL-2、 ABSL-3、 ABSL-4 表示包括从事动物活体操作的实验室的相应生物安全防护水平。

4.2.3 动物生物安全实验室分为从事脊椎动物和无脊椎动物实验活动的实验室。

4.2.4 根据实验活动、采用的个体防护装备和基础隔离设施的不同，实验室分为：

a) 操作通常认为非经空气传播致病性生物因子的实验室；

b) 可有效利用安全隔离装置（如： II 级生物安全柜） 操作常规量经空气传播致病性生物因子的实验室；

c) 不能有效利用安全隔离装置操作常规量经空气传播致病性生物因子的实验室；

d) 利用具有生命支持系统的正压服操作常规量经空气传播致病性生物因子的实验室；

e) 利用具有Ⅲ级生物安全柜操作常规量经空气传播致病性生物因子的实验室。

5 风险评估与风险控制

5.1 总则

实验室应建立并维持风险评估和风险控制制度，应明确实验室持续进行风险识别、风险评估和风险控制的具体要求（参见附录A） 。

5.2 风险识别

当实验活动涉及致病性生物因子时，应识别但不限于5.2.a)至5.2.j） 所述的风险因素：

a) 实验活动涉及致病性生物因子的已知或未知的特性，如：

1) 危害程度分类；

2) 生物学特性；

3) 传播途径和传播力；

4) 感染性和致病性：易感性、宿主范围、致病所需的量、潜伏期、临床症状、病程、预后等；

5) 与其他生物和环境的相互作用、相关实验数据、流行病学资料；

6) 在环境中的稳定性；

7) 预防、治疗和诊断措施，包括疫苗、治疗药物与感染检测用诊断试剂。

b) 涉及致病性生物因子的实验活动，如：

1) 菌（毒）种及感染性物质的领取、转运、保存、销毁等；

2) 分离、培养、鉴定、制备等操作；

3) 易产生气溶胶的操作，如离心、研磨、振荡、匀浆、超声、接种、冷冻干燥等；

4) 锐器的使用，如注射针头、解剖器材、玻璃器皿等。

c) 实验活动涉及到遗传修饰生物体（ GMOs）时，应考虑重组体引起的危害。

d) 涉及致病性生物因子的动物饲养与实验活动：

1) 抓伤、咬伤；

2) 动物毛屑、呼吸产生的气溶胶；

3) 解剖、采样、检测等；

4) 排泄物、分泌物、组织/器官/尸体、垫料、废物处理等；

5) 动物笼具、器械、控制系统等可能出现故障。

e) 感染性废物处置过程中的风险：

1) 废物容器、包装、标识；

2) 收集、消毒、储存、运输等；

3) 感染性废物的泄露；

4) 灭菌的可靠性；

5) 设施外人群可能接触到感染性废物的风险。

f) 实验活动安全管理的风险，包括但不限于：

1) 消除、减少或控制风险的管理措施和技术措施，及采取措施后残余风险或带来的新风险；

2) 运行经验和风险控制措施，包括与设施、设备有关的管理程序、操作规程、维护保养规程

等的潜在风险；

3) 实施应急措施时可能引起的新的风险。

g) 涉及致病性生物因子实验活动的相关人员：

1) 专业及生物安全知识、操作技能；

2) 对风险的认知；

3) 心理素质；

4) 专业及生物安全培训状况；

5) 意外事件/事故的处置能力；

6) 健康状况；

7) 健康监测、医疗保障及医疗救治；

8) 对外来实验人员安全管理及提供的保护措施。

h) 实验室设施、设备：

1) 生物安全柜、离心机、摇床、培养箱等；

2) 废物、废水处理设施、设备；

3) 个体防护装备；

适用时，包括：

1) 防护区的密闭性、压力、温度与气流控制；

2) 互锁、密闭门以及门禁系统；

3) 与防护区相关联的通风空调系统及水、电、气系统等；

4) 安全监控和报警系统；

5) 动物饲养、操作的设施设备；

6) 菌（毒）种及样本保藏的设施设备；

7) 防辐射装置；

8) 生命支持系统、正压防护服、化学淋浴装置等。

i) 实验室生物安保制度和安保措施，重点识别所保藏的或使用的致病性生物因子被盗、滥用和恶意释放的风险。

j) 已发生的实验室感染事件的原因分析。

5.3 风险评估

5.3.1 风险评估应以国家法律、法规、标准、规范，以及权威机构发布的指南、数据等为依据。对已识别的风险进行分析，形成风险评估报告。

5.3.2 风险评估应由具有经验的不同领域的专业人员（不限于本机构内部的人员）进行。

5.3.3 实验室应在 5.2 的基础上， 并结合但不限于以下情况进行风险评估：

a) 病原体生物学特性或防控策略发生变化时；

b) 开展新的实验活动或变更实验活动（包括设施、设备、人员、活动范围、规程等）；

c) 操作超常规量或从事特殊活动；

d) 本实验室或同类实验室发生感染事件、感染事故；

e) 相关政策、法规、标准等发生改变。

5.4 风险评估报告

5.4.1 风险评估报告的内容至少应包括：实验活动（项目计划）简介、评估目的、评估依据、评估方法/程序、评估内容、评估结论。

5.4.2 风险评估报告应注明评估时间及编审人员。

5.4.3 风险评估报告应经实验室设立单位批准。

5.5 风险控制

5.5.1 依据风险评估结论采取相应的风险控制措施。

5.5.2 采取风险控制措施时宜首优先考虑控制风险源，再考虑采取其他措施降低风险。

6 实验室设施和设备要求

6.1 实验室设计原则和基本要求

6.1.1 实验室选址、设计和建造应符合国家和地方建设规划、生物安全、环境保护和建筑技术规范等

规定和要求。

6.1.2 实验室的设计应保证对生物、化学、辐射和物理等危险源的防护水平控制在经过评估的可接受程度，防止危害环境。

6.1.3 实验室的建筑结构应符合国家有关建筑规定。

6.1.4 在充分考虑生物安全实验室地面、墙面、顶板、管道、橱柜等在消毒、清洁、防滑、防渗漏、防积尘等方面特殊要求的基础上，从节能、环保、安全和经济性等多方面综合考虑，选用适当的符合国家标准要求的建筑材料。

6.1.5 实验室的设计应充分考虑工作方便、流程合理、人员舒适等问题。

6.1.6 实验室内温度、湿度、照度、噪声和洁净度等室内环境参数应符合工作要求，以及人员舒适性、卫生学等要求。

6.1.7 实验室的设计、在满足工作要求、安全要求的同时，应充分考虑节能和冗余。

6.1.8 实验室的走廊和通道应不妨碍人员和物品通过。

6.1.9 应设计紧急撤离路线，紧急出口处应有明显的标识。

6.1.10 房间的门根据需要安装门锁，门锁应便于内部快速打开。

6.1.11 实验室应根据房间或实验间在用、停用、消毒、维护等不同状态时的需要，采取适当的警示和进入限制措施，如警示牌、警示灯、警示线、门禁等。

6.1.12 实验室的安全保卫应符合国家相关部门对该级别实验室的安全管理规定和要求。

6.1.13 应根据生物材料、样本、药品、化学品和机密资料等被误用、被盗和被不正当使用的风险评估，采取相应的物理防范措施。

6.1.14 应有专门设计以确保存储、转运、收集、处理和处置危险物料的安全。

6.2 BSL-1 实验室

6.2.1 应为实验室仪器设备的安装、 清洁和维护、 安全运行提供足够的空间。

6.2.2 实验室应有足够的空间和台柜等摆放实验室设备和物品。

6.2.3 在实验室的工作区外应当有存放外衣和私人物品的设施，应将个人服装与实验室工作服分开放置。

6.2.4 进食、饮水和休息的场所应设在实验室的工作区外。

6.2.5 实验室墙壁、 顶板和地板应当光滑、易清洁、防渗漏并耐化学品和消毒剂的腐蚀。地面应防滑，不得在实验室内铺设地毯。

6.2.6 实验室台（桌）柜和座椅等应稳固和坚固，边角应圆滑。实验台面应防水，并能耐受中等程度的热、有机溶剂、酸碱、消毒剂及其他化学剂。

6.2.7 应根据工作性质和流程合理摆放实验室设备、台柜、物品等，避免相互干扰、交叉污染，并应不妨碍逃生和急救。台（桌）柜和设备之间应有足够的间距，以便于清洁。

6.2.8 实验室应设洗手池，水龙头开关宜为非手动式，宜设置在靠近出口处。

6.2.9 实验室的门应有可视窗并可锁闭，并达到适当的防火等级，门锁及门的开启方向应不妨碍室内人员逃生。

6.2.10 实验室可以利用自然通风，开启窗户应安装防蚊虫的纱窗。如果采用机械通风，应避免气流流向导致的污染和避免污染气流在实验室之间或与其他区域之间串通而造成交叉污染。

6.2.11 应保证实验室内有足够的照明，避免不必要的反光和闪光。

6.2.12 实验室涉及刺激性或腐蚀性物质的操作，应在 30 m 内设洗眼装置，风险较大时应设紧急喷淋装置。

6.2.13 若涉及使用有毒、刺激性、挥发性物质，应配备适当的排风柜（罩）。

6.2.14 若涉及使用高毒性、放射性等物质，应配备相应的安全设施设备和个体防护装备，应符合国家、地方的相关规定和要求。

6.2.15 若使用高压气体和可燃气体，应有安全措施，应符合国家、地方的相关规定和要求。

6.2.16 应有可靠和足够的电力供应，确保用电安全。

6.2.17 应设应急照明装置，同时考虑合适的安装位置，以保证人员安全离开实验室。

6.2.18 应配备足够的固定电源插座，避免多台设备使用共同的电源插座。应有可靠的接地系统，应在关键节点安装漏电保护装置或监测报警装置。

6.2.19 应满足实验室所需用水。

6.2.20 给水管道应设置倒流防止器或其他有效的防止回流污染的装置；给排水系统应不渗漏，下水应有防回流设计。

6.2.21 应配备适用的应急器材，如消防器材、意外事故处理器材、急救器材等。

6.2.22 应配备适用的通讯设备。

6.2.23 必要时，可配备适当的消毒、灭菌设备。

6.3 BSL-2 实验室

6.3.1 普通型 BSL-2 实验室

6.3.1.1 适用时，应符合 6.2 的要求。

6.3.1.2 实验室主入口的门、放置生物安全柜实验间的门应可自动关闭；实验室主入口的门应有进入

控制措施。

6.3.1.3 实验室工作区域外应有存放备用物品的条件。

6.3.1.4 应在实验室或其所在的建筑内配备压力蒸汽灭菌器或其他适当的消毒、灭菌设备，所配备的消毒、灭菌设备应以风险评估为依据。

6.3.1.5 应在实验室工作区配备洗眼装置，必要时，应在每个工作间配备洗眼装置。

6.3.1.6 应在操作病原微生物及样本的实验区内配备二级生物安全柜。

6.3.1.7 应按产品的设计、使用说明书的要求安装和使用生物安全柜。

6.3.1.8 如果使用管道排风的生物安全柜，应通过独立于建筑物其他公共通风系统的管道排出。

6.3.1.9 实验室入口应有生物危害标识，出口应有逃生发光指示标识。

6.3.2 加强型 BSL-2 实验室

6.3.2.1 适用时， 应符合 6.3.1 的要求。

6.3.2.2 加强型 BSL-2 实验室应包含缓冲间和核心工作间。

6.3.2.3 缓冲间可兼作防护服更换间。必要时，可设置准备间和洗消间等。

6.3.2.4 缓冲间的门宜能互锁。如果使用互锁门，应在互锁门的附近设置紧急手动互锁解除开关。

6.3.2.5 实验室应设洗手池；水龙头开关应为非手动式，宜设置在靠近出口处。

6.3.2.6 采用机械通风系统，送风口和排风口应采取防雨、防风、防杂物、防昆虫及其他动物的措施，送风口应远离污染源和排风口。排风系统应使用高效空气过滤器。

6.3.2.7 核心工作间内送风口和排风口的布置应符合定向气流的原则，利于减少房间内的涡流和气流死角。

6.3.2.8 核心工作间气压相对于相邻区域应为负压，压差宜不低于 10 Pa。在核心工作间入口的显著位置，应安装显示房间负压状况的压力显示装置。

6.3.2.9 应通过自动控制措施保证实验室压力及压力梯度的稳定性，并可对异常情况报警。

6.3.2.10 实验室的排风应与送风连锁，排风先于送风开启，后于送风关闭。

6.3.2.11 实验室应有措施防止产生对人员有害的异常压力，围护结构应能承受送风机或排风机异常时导致的空气压力载荷。

6.3.2.12 核心工作间温度 18 ℃～26 ℃，噪音应低于 68 dB。

6.3.2.13 实验室内应配置压力蒸汽灭菌器，以及其他适用的消毒设备

6.4 BSL-3 实验室

6.4.1 要求

适用时，应符合 6.3 的要求。

6.4.2 平面布局

6.4.2.1 实验室应在建筑物中自成隔离区或为独立建筑物，应有出入控制。

6.4.2.2 实验室应明确区分辅助工作区和防护区。防护区中直接从事高风险操作的工作间为核心工作间，人员应通过缓冲间进入核心工作间。

6.4.2.3 对于操作通常认为非经空气传播致病性生物因子的实验室，实验室辅助工作区应至少包括监控室和清洁衣物更换间；防护区应至少包括缓冲间及核心工作间。

6.4.2.4 对于可有效利用安全隔离装置（如：生物安全柜）操作常规量经空气传播致病性生物因子的实验室，实验室辅助工作区应至少包括监控室、清洁衣物更换间和淋浴间；防护区应至少包括防护服更换间、缓冲间及核心工作间。实验室核心工作间不宜直接与其他公共区域相邻。

6.4.2.5 可根据需要安装传递窗。如果安装传递窗，其结构承压力及密闭性应符合所在区域的要求，以保证围护结构的完整性，并应具备对传递窗内物品表面进行消毒的条件。

6.4.2.6 应充分考虑生物安全柜、双扉压力蒸汽灭菌器等大设备进出实验室的需要，实验室应设有尺寸足够的设备门。

6.4.3 围护结构

6.4.3.1 实验室宜按甲类建筑设防，耐火等级应符合相关标准要求。

6.4.3.2 实验室防护区内围护结构的内表面应光滑、耐腐蚀、不开裂、防水，所有缝隙和贯穿处的接缝都应可靠密封， 应易清洁和消毒。

6.4.3.3 实验室防护区内的地面应防渗漏、完整、光洁、防滑、耐腐蚀、不起尘。

6.4.3.4 实验室内所有的门应可自动关闭，需要时，应设观察窗；门的开启方向不应妨碍逃生。

6.4.3.5 实验室内所有窗户应为密闭窗，玻璃应耐撞击、防破碎。

6.4.3.6 实验室及设备间的高度应满足设备的安装要求，应有维修和清洁空间。

6.4.3.7 实验室防护区的顶棚上不得设置检修口等。

6.4.3.8 在通风系统正常运行状态下，采用烟雾测试法检查实验室防护区内围护结构的严密性时，所有缝隙应无可见泄漏。

6.4.4 通风空调系统

6.4.4.1 应安装独立的实验室送排风系统，确保在实验室运行时气流由低风险区向高风险区流动，同时确保实验室空气通过 HEPA 过滤器过滤后排出室外。

6.4.4.2 实验室空调系统的设计应充分考虑生物安全柜、离心机、二氧化碳培养箱、冰箱、压力蒸汽灭菌器、紧急喷淋装置等设备的冷、热、湿负荷。

6.4.4.3 实验室防护区房间内送风口和排风口的布置应符合定向气流的原则，利于减少房间内的涡流和气流死角；送排风应不影响其他设备的正常功能，在生物安全柜操作面或其他有气溶胶发生地点的上方不得设送风口。

6.4.4.4 不得循环使用实验室防护区排出的空气，不得在实验室防护区内安装分体空调等在室内循环处理空气的设备。

6.4.4.5 应按产品的设计要求和使用说明安装生物安全柜和其排风管道系统。

6.4.4.6 实验室的送风应经过初效、中效过滤器和 HEPA 过滤器过滤。

6.4.4.7 实验室防护区室外排风口应设置在主导风的下风向，与新风口的直线距离应大于 12 m，并应高于所在建筑的屋面 2 m 以上，应有防风、防雨、防鼠、防虫设计，但不应影响气体向上空排放。

6.4.4.8 HEPA 过滤器的安装位置应尽可能靠近送风管道（在实验室内的送风口端）和排风管道（在实验室内的排风口端）。

6.4.4.9 应可以在原位对排风 HEPA 过滤器进行消毒和检漏。

6.4.4.10 如在实验室防护区外使用高效过滤器单元，其结构应牢固，应能承受 2 500 Pa 的压力；高效过滤器单元的整体密封性应达到在关闭所有通路并维持腔室内的温度稳定的条件下，若使空气压力维持在 1 000 Pa 时，腔室内每分钟泄漏的空气量应不超过腔室净容积的 0.1%。

6.4.4.11 应在实验室防护区送风和排风管道的关键节点安装密闭阀，必要时，可完全关闭。

6.4.4.12 实验室的排风管道应采用耐腐蚀、耐老化、不吸水的材料制作，宜使用不锈钢管道。密闭阀与实验室防护区相通的送风管道和排风管道应牢固、气密、易消毒，管道的密封性应达到在关闭所有通路并维持管道内的温度稳定的条件下，若使空气压力维持在 500Pa 时，管道内每分钟泄漏的空气量应不超过管道内净容积的 0.2%。

6.4.4.13 排风机应一用一备。应尽可能减少排风机后排风管道正压段的长度，该段管道不应穿过其他房间。

6.4.5 供水与供气系统

6.4.5.1 应在实验室防护区靠近实验间出口处设置非手动洗手设施；如果实验室不具备供水条件，应设非手动手消毒装置。

6.4.5.2 应在实验室的给水与市政给水系统之间设防回流装置或其他有效的防止倒流污染的装置，且这些装置应设置在防护区外，宜设置在防护区围护结构的边界处。

6.4.5.3 进出实验室的液体和气体管道系统应牢固、不渗漏、防锈、耐压、耐温（冷或热）、耐腐蚀。应有足够的空间清洁、维护和维修实验室内暴露的管道，应在关键节点安装截止阀、防回流装置或 HEPA过滤器等。

6.4.5.4 如果有供气（液）罐等，应放在实验室防护区外易更换和维护的位置，安装牢固，不应将不相容的气体或液体放在一起。

6.4.5.5 如果有真空装置，应有防止真空装置的内部被污染的措施；不应将真空装置安装在实验场所之外。

6.4.6 污物处理及消毒系统

6.4.6.1 应在实验室防护区内设置符合生物安全要求的压力蒸汽灭菌器。宜安装生物安全型的双扉压力蒸汽灭菌器，其主体应安装在易维护的位置，与围护结构的连接之处应可靠密封。

6.4.6.2 对实验室防护区内不能使用压力蒸汽灭菌的物品应有其他消毒、灭菌措施。

6.4.6.3 压力蒸汽灭菌器的安装位置不应影响生物安全柜等安全隔离装置的气流。

6.4.6.4 可根据需要设置传递物品的渡槽。如果设置传递物品的渡槽，应使用强度符合要求的耐腐蚀性材料，并方便更换消毒液；渡槽与围护结构的连接之处应可靠密封。

6.4.6.5 地面液体收集系统应有防液体回流的装置。

6.4.6.6 进出实验室的液体和气体管道系统应牢固、不渗漏、防锈、耐压、耐温（冷或热）、耐腐蚀。排水管道宜明设，并应有足够的空间清洁、维护和维修实验室内暴露的管道。在发生意外的情况下，为减少污染范围，利于设备的检修和维护，应在关键节点安装截止阀。

6.4.6.7 实验室防护区内如果有下水系统，应与建筑物的下水系统完全隔离；下水应直接通向本实验室专用的污水处理系统。

6.4.6.8 所有下水管道应有足够的倾斜度和排量，确保管道内不存水；管道的关键节点应按需要安装防回流装置、存水弯（深度应适用于空气压差的变化）或密闭阀门等；下水系统应符合相应的耐压、耐热、耐化学腐蚀的要求， 安装牢固，无泄漏，便于维护、清洁和检查。

6.4.6.9 实验室排水系统应单独设置通气口，通气口应设 HEPA 过滤器或其他可靠的消毒装置，同时应保证通气口处通风良好。如通气口设置 HEPA 过滤器，则应可以在原位对 HEPA 过滤器进行消毒和检漏。

6.4.6.10 实验室应以风险评估为依据，确定实验室防护区污水（包括污物）的消毒方法；应对消毒效果进行监测，确保每次消毒的效果。

6.4.6.11 实验室辅助区的污水应经处理达标后方可排放市政管网处。

6.4.6.12 应具备对实验室防护区、设施设备及与其直接相通的管道进行消毒的条件。

6.4.6.13 应在实验室防护区可能发生生物污染的区域（如生物安全柜、离心机附近等）配备便携的消毒装置，同时应备有足够的适用消毒剂。当发生意外时，及时进行消毒处理。

6.4.7 电力供应系统

6.4.7.1 电力供应应按一级负荷供电，满足实验室的用电要求，并应有冗余。

6.4.7.2 生物安全柜、送风机和排风机、照明、自控系统、监视和报警系统等应配备不间断备用电源，电力供应至少维持 30 min。

6.4.7.3 应在实验室辅助工作区安全的位置设置专用配电箱，其放置位置应考虑人员误操作的风险、恶意破坏的风险及受潮湿、水灾侵害等风险。

6.4.8 照明系统

6.4.8.1 实验室核心工作间的照度应不低于 350 lx，其他区域的照度应不低于 200 lx，宜采用吸顶式密闭防水洁净照明灯。

6.4.8.2 应避免过强的光线和光反射。

6.4.8.3 应设应急照明系统以及紧急发光疏散指示标识。

6.4.9 自控、监视与报警系统

6.4.9.1 实验室自动化控制系统应由计算机中央控制系统、通讯控制器和现场执行控制器等组成。应具备自动控制和手动控制的功能，应急手动应有优先控制权，且应具备硬件联锁功能。

6.4.9.2 实验室自动化控制系统应保证实验室防护区内定向气流的正确及压力压差的稳定。

6.4.9.3 实验室通风系统联锁控制程序应先启动排风，后启动送风；关闭时，应先关闭送风及密闭阀，后关排风及密闭阀。

6.4.9.4 通风系统应与Ⅱ级 B 型生物安全柜、排风柜（罩）等局部排风设备连锁控制，确保实验室稳定运行，并在实验室通风系统开启和关闭过程中保持有序的压力梯度。

6.4.9.5 当排风系统出现故障时，应先将送风机关闭，待备用排风机启动后，再启动送风机，避免实验室出现正压。

6.4.9.6 当送风系统出现故障时，应有效控制实验室负压在可接受范围内，避免影响实验室人员安全、生物安全柜等安全隔离装置的正常运行和围护结构的安全。

6.4.9.7 应能够连续监测送排风系统 HEPA 过滤器的阻力。

6.4.9.8 应在有压力控制要求的房间入口的显著位置，安装显示房间压力的装置。

6.4.9.9 中央控制系统应可以实时监控、 记录和存储实验室防护区内压力、压力梯度、温度、湿度等有控制要求的参数，以及排风机、送风机等关键设施设备的运行状态、电力供应的当前状态等。应设置历史记录档案系统，以便随时查看历史记录，历史记录数据宜以趋势曲线结合文本记录的方式表达。

6.4.9.10 中央控制系统的信号采集间隔时间应不超过 1 min，各参数应易于区分和识别。

6.4.9.11 实验室自控系统报警应分为一般报警和紧急报警。一般报警为过滤器阻力的增大、温湿度偏离正常值等， 暂时不影响安全，实验活动可持续进行的报警；紧急报警指实验室出现正压、压力梯度持续丧失、风机切换失败、停电、火灾等， 对安全有影响，应终止实验活动的报警。一般报警应为显示报警，紧急报警应为声光报警和显示报警，可以向实验室内外人员同时显示紧急警报， 应在核心工作间内设置紧急报警按钮。

6.4.9.12 核心工作间的缓冲间的入口处应有指示核心工作间工作状态的装置，必要时，设置限制进入核心工作间的连锁机制。

6.4.9.13 实验室应设电视监控，在关键部位设置摄像机，可实时监视并录制实验室活动情况和实验室周围情况。监视设备应有足够的分辨率和影像存储容量。

6.4.10 实验室通讯系统

6.4.10.1 实验室防护区内应设置向外部传输资料和数据的传真机或其他电子设备。

6.4.10.2 监控室和实验室内应安装语音通讯系统。如果安装对讲系统，宜采用向内通话受控、向外通话非受控的选择性通话方式。

6.4.11 实验室门禁管理系统

6.4.11.1 实验室应有门禁管理系统，应保证只有获得授权的人员才能进入实验室，并能够记录人员出入。

6.4.11.2 实验室应设门互锁系统，应在互锁门的附近设置紧急手动解除互锁开关，需要时，可立即解除门的互锁。

6.4.11.3 当出现紧急情况时，所有设置互锁功能的门应能处于可开启状态。

6.4.12 参数要求

6.4.12.1 实验室的围护结构应能承受送风机或排风机异常时导致的空气压力载荷。

6.4.12.2 适用于 4.2.4 a)实验室， 其核心工作间的气压（负压）与室外大气压的压差值应不小于 30 Pa，与相邻区域的压差（负压）应不小于 10 Pa；对于可有效利用安全隔离装置操作常规量经空气传播致病性生物因子的实验室， 其核心工作间的气压（负压）与室外大气压的压差值应不小于 40 Pa，与相邻区域的压差（负压）应不小于 15 Pa。

6.4.12.3 实验室防护区各房间的最小换气次数应不小于 12 次/h。

6.4.12.4 实验室的温度宜控制在 18 ℃～26 ℃范围内。

6.4.12.5 正常情况下，实验室的相对湿度宜控制在 30%～70%范围内；消毒状态下，实验室的相对湿度应能满足消毒的技术要求。

6.4.12.6 在安全柜开启情况下，核心工作间的噪声应不大于 68 dB。

6.4.12.7 实验室防护区的静态洁净度应不低于 8 级水平。

6.5 BSL-4 实验室

6.5.1 类型

6.5.1.1 BSL-4 实验室分为正压服型实验室和安全柜型实验室。

6.5.1.2 在安全柜型实验室中，所有微生物的操作均在Ⅲ级生物安全柜中进行。在正压服型实验室中，工作人员应穿着配有生命支持系统的正压防护服。

6.5.1.3 适用时，应符合 6.4 的要求。

6.5.2 平面布局

6.5.2.1 实验室应在建筑物中自成隔离区或为独立建筑物，应有出入控制。

6.5.2.2 BSL-4 实验室防护区应至少包括核心工作间、缓冲间、外防护服更换间等，外防护服更换间应为气锁，辅助工作区应包括监控室、清洁衣物更换间等。

6.5.2.3 正压服型 BSL-4 实验室的防护区应包括核心工作间、化学淋浴间、外防护服更换间等，化学淋浴间应为气锁，可兼作缓冲间，辅助工作区应包括监控室、清洁衣物更换间等。

6.5.3 围护结构

6.5.3.1 实验室防护区的围护结构应尽量远离建筑外墙。

6.5.3.2 实验室的核心工作间应尽可能设置在防护区的中部。

6.5.3.3 实验室防护区围护结构的气密性应达到在关闭受测房间所有通路并保持房间内温度稳定的条件下，当房间内的空气压力上升到 500 Pa 后， 20 min 内自然衰减的气压小于 250 Pa。

6.5.3.4 可根据需要安装传递窗。如果安装传递窗，其结构承压力及密闭性应符合所在区域的要求；需要时，应配备符合气锁要求并具备消毒条件的传递窗。

6.5.4 通风空调系统

6.5.4.1 实验室的排风应经过两级 HEPA 过滤器处理后排放。

6.5.4.2 应可以在原位对送、排风 HEPA 过滤器进行消毒和检漏。

6.5.5 生命支持系统

6.5.5.1 正压服型实验室应同时配备紧急支援气罐，紧急支援气罐的供气时间应不少于 60 min/人。

6.5.5.2 生命支持系统应有不间断备用电源，连续供电时间应不少于 60 min。

6.5.5.3 供呼吸使用的气体的压力、流量、含氧量、温度、湿度、有害物质的含量等应符合职业安全

的要求。

6.5.5.4 生命支持系统应具备必要的报警装置。

6.5.5.5 根据工作情况，进入实验室的工作人员配备满足工作需要的合体的正压防护服，实验室应配备正压防护服检漏器具和维修工具。

6.5.6 污物处理及消毒系统

6.5.6.1 应在实验室的核心工作间内配备生物安全型压力蒸汽灭菌器；如果配备双扉压力蒸汽灭菌器，其主体所在房间的室内气压应为负压，并应设在实验室防护区内易更换和维护的位置。

6.5.6.2 化学淋浴消毒装置应在无电力供应的情况下仍可以使用，消毒液储存器的容量应满足所有情况下对消毒使用量的需求。

6.5.6.3 实验室防护区内所有需要运出实验室的物品或其包装的表面应经过可靠灭菌，符合安全要求。

6.5.7 参数要求

6.5.7.1 实验室防护区内所有区域的室内气压应为负压，实验室核心工作间的气压（负压）与室外大气压的压差值应不小于 60 Pa，与相邻区域的压差（负压）应不小于 25 Pa。

6.5.7.2 安全柜型实验室应在Ⅲ级生物安全柜或相当的安全隔离装置内操作致病性生物因子；同时应具备与安全隔离装置配套的物品传递设备以及生物安全型压力蒸汽灭菌器。

6.6 动物实验室

6.6.1 ABSL－1 实验室

6.6.1.1 实验室选址、 设计和建造应符合国家和地方建设规划、生物安全、环境保护和建筑技术规范等规定和要求。

6.6.1.2 围护结构的空间配置、强度要求等应与所饲养的动物种类相适应。

6.6.1.3 动物饲养环境与设施条件应符合实验动物微生物等级要求。

6.6.1.4 实验室应分为动物饲养间和实验操作间等部分，必要时，应具备动物检疫室。

6.6.1.5 动物饲养间和实验操作间的室内气压相对外环境宜为负压，不得循环使用动物实验室排出的空气。

6.6.1.6 如果安装窗户，所有窗户应密闭；需要时，窗户外部应装防护网。

6.6.1.7 实验室应与建筑物内的其他域相对隔离或独立。

6.6.1.8 实验室的门应有可视窗，应安装为向里开启。

6.6.1.9 门应能够自动关闭，需要时，可以上锁。

6.6.1.10 实验室的工作表面应能良好防水和易于消毒。如果有地面液体收集系统，应设防液体回流装置，存水弯应有足够的深度。

6.6.1.11 应设置洗手池或手消毒装置，宜设置在出口处。

6.6.1.12 应设置适合、良好的实验动物饲养笼具或护栏，防止动物逃逸、损毁；应可以对动物笼具进行清洗和消毒。

6.6.1.13 饲养笼具除考虑安全要求外还应考虑对动物福利的要求。

6.6.1.14 动物尸体及相关废物的处置设施和设备应符合国家相关规定的要求。

6.6.1.15 动物尸体及组织应做无害化处理，废物应彻底灭菌后方可排出。

6.6.1.16 实验室应具备常用个人防护物品，如防动物面罩等； 动物解剖等特殊防护用品，如防切割手套等。

6.6.2 ABSL-2 实验室

6.6.2.1 适用时，应符合 6.3 和 6.6.1 的要求。

6.6.2.2 动物饲养间和实验操作间应在出入口处设置缓冲间。

6.6.2.3 应设置非手动洗手装置或手消毒装置，宜设置在出口处。

6.6.2.4 应在实验室或其邻近区域配备压力蒸汽灭菌器。

6.6.2.5 送风应经 HEPA 过滤器过滤后进入实验室。

6.6.2.6 实验室功能上分为能有效利用安全隔离装置控制病原微生物的实验室和不能有效利用安全隔离装置控制病原微生物的实验室。

6.6.2.7 从事可能产生有害气溶胶的动物实验活动应在能有效利用安全隔离装置控制病原微生物的实验室内进行；排气应经 HEPA 过滤器过滤后排出。

6.6.2.8 动物饲养间和实验操作间的室内气压相对外环境应为负压，气体应直接排放到其所在的建筑物外。

6.6.2.9 适用时，如大量动物实验、病原微生物致病性较强、传播力较大、动物可能增强病原毒力或毒力回复时的活动，宜在能有效利用安全隔离装置控制病原微生物的实验室内进行；排气应经 HEPA 过滤器过滤后排出。

6.6.2.10 当不能满足 6.6.2.9 时或在不能有效利用安全隔离装置控制病原微生物的实验室进行一般感染性动物实验时，应使用 HEPA 过滤器过滤动物饲养间排出的气体。

6.6.2.11 实验室防护区室外排风口应设置在主导风的下风向，与新风口的直线距离应大于 12 m，并应高于所在建筑的屋面 2 m 以上，应有防风、防雨、防鼠、防虫设计，但不影响气体向上空排放。

6.6.2.12 污水、污物等应消毒处理，并应对消毒效果进行检测，以确保达到排放要求。

6.6.2.13 实验室应提供有效的、两种以上的消毒、灭菌方法。

6.6.3 ABSL－3 实验室

6.6.3.1 适用时，应符合 6.6.2 的要求。

6.6.3.2 根据动物物种和病原危害程度要求，应在实验室防护区设淋浴间，需要时，应设置强制淋浴装置。

6.6.3.3 必要时，实验室应设置动物准备间、动物传递窗、动物走廊。

6.6.3.4 动物饲养间和实验操作间属于核心工作间。入口和出口，均应设置缓冲间。

6.6.3.5 动物饲养间和实验操作间应尽可能设在整个实验室的中心部位，不应直接与其他公共区域相邻。

6.6.3.6 动物饲养间和动物操作间应安装监视设备和通讯设备。

6.6.3.7 适用于 4.2.4 b)验室的防护区应至少包括淋浴间、防护服更换间、缓冲间及核心工作间。核心工作间应包括动物饲养间和实验操作间，如解剖间。

6.6.3.8 当不能有效利用安全隔离装置饲养动物时，应根据进一步的风险评估确定实验室的生物安全防护要求。

6.6.3.9 适用于 4.2.4 a)和 4.2.4 b)的核心工作间气压（负压）与室外大气压的压差值应不小于 60Pa，与相邻区域的压差（负压）应不低于 15 Pa。

6.6.3.10 适用于 4.2.4 c)的核心工作间（ 动物饲养间和实验操作间） 的缓冲间应为气锁， 并具备能有效控制的防护服或传递物品的表面进行消毒的条件。

6.6.3.11 适用于 4.2.4 c)的核心工作间（ 动物饲养间和实验操作间） ，应有严格限制进入的门禁措施。

6.6.3.12 适用于 4.2.4 c)的核心工作间（ 动物饲养间和实验操作间） ，应可以在原位送风 HEPA 过滤器进行消毒和检漏；应根据风险评估的结果， 确定动物饲养间排风是否需要经过两级 HEPA 过滤器的过滤。

6.6.3.13 适用于 4.2.4 c)的核心工作间（ 动物饲养间和实验操作间） 的气压（负压）与室外大气压的压差值应不小于 80 Pa，与相邻区域的压差（负压）应不低于 25 Pa。

6.6.3.14 适用于 4.2.4 c)的核心工作间（ 动物饲养间和实验操作间） 及其缓冲间的气密性应达到在关闭受测房间所有通路并维持房间内的温度在设计范围上限的条件下，若使空气压力维持在 250 Pa 时，房间内每小时泄漏的空气量应不超过受测房间净容积的 10%。

6.6.3.15 送风机、排风机均一用一备。

6.6.3.16 实验室内应配备便携式消毒装置，并应备有足够的适用消毒剂，及时对污染进行处理。

6.6.3.17 应有对动物尸体和废物进行灭菌，对动物笼具进行清洁和消毒的装置，需要时，对所有物品或其包装的表面在运出实验室前进行清洁和消毒。

6.6.3.18 应在风险评估的基础上，适当处理防护区内淋浴间的污水，并应对消毒效果进行监测，以确保达到排放要求。

6.6.3.19 实验室应提供适合、优良的个人防护物品。可重复使用时，应能进行有效消毒。

6.6.4 ABSL－4 实验室

6.6.4.1 适用时，应符合 6.6.3 的要求。

6.6.4.2 淋浴间应设置强制淋浴装置。

6.6.4.3 根据实验活动和动物种类，实验室应提供良好的实验服和适合的个体防护装备。

6.6.4.4 动物饲养间的缓冲间应为气锁。

6.6.4.5 应有严格限制进入动物饲养间的门禁措施。

6.6.4.6 动物饲养间和实验操作间的气压（负压）与室外大气压的压差值应不小于 100 Pa；与相邻区域气压的压差（负压）应不低于 25 Pa。

6.6.4.7 动物饲养间和实验操作间及其缓冲间的气密性应达到在关闭受测房间所有通路并保持房间内温度稳定的条件下，当房间内的空气压力上升到 500 Pa 后， 20 min 内自然衰减的压力小于 250 Pa。

6.6.4.8 应有装置和技术对所有物品或其包装的表面在运出动物饲养间前进行清洁和消毒。

6.6.4.9 应有对动物尸体、组织、代谢物、标本及相关废物进行彻底消毒和灭菌的装备， 应严格按相关要求进行处置。必要时，进行两次消毒、灭菌。

6.6.5 无脊椎动物实验室

6.6.5.1 根据动物种类危害和病原危害，防护水平应根据国家相关主管部门的规定和风险评估的结果确定。

6.6.5.2 实验室的建造、功能区分应充分考虑动物特性和实验活动，能重点实现控制动物本身的危害或可能从事病原感染的双重危害。

6.6.5.3 实验室应具备有效控制动物逃逸、藏匿等的防护装置。

6.6.5.4 从事节肢动物（特别是可飞行、快爬或跳跃的昆虫）的实验活动，应采取以下适用的措施（但不限于）：

a) 应通过缓冲间进入动物饲养间或操作间，缓冲间内应配备适用的捕虫器和灭虫剂；

b) 应在所有关键的可开启的门窗、所有通风管道的关键节点安装防节肢动物逃逸的纱网；

c) 应在不同区域饲养、操作未感染和已感染节肢动物；

d) 应具备动物饲养间或操作间、缓冲间密闭和进行整体消毒的条件；应设喷雾式杀虫装置；

e) 应设制冷温装置，需要时，可以通过减低温度及时降低动物的活动能力；

f) 应有机制或装置确保水槽和存水弯管等设备内的液体或消毒液不干涸；

g) 应配备消毒、灭菌设备和技术，能对所有实验后废弃动物、尸体、废物进行彻底消毒、灭菌处

理；

h) 应有机制监测和记录会飞、爬、跳跃的节肢动物幼虫和成虫的数量；

i) 应配备适用于放置装蜱螨容器的油碟；应具备操作已感染或潜在感染的节肢动物的低温盘；

j) 应具备带双层网的笼具以饲养或观察已感染或潜在感染的逃逸能力强的节肢动物；

k) 应具备适用的生物安全柜或相当的安全隔离装置以操作已感染或潜在感染的节肢动物；

l) 应设置高清晰监视器和通讯设备，动态监控动物的活动。

7 实验室生物安全管理要求

7.1 管理体系

7.1.1 实验室设立单位应有明确的法律地位，生物安全三级、四级实验室应具有从事相关活动的资格。

7.1.2 实验室的设立单位应成立生物安全委员会及实验动物使用管理委员会（适用时），负责组织专家对实验室的设立和运行进行监督、咨询、指导、评估（包括实验室运行的生物安全风险评估和实验室生物安全事故的处置）。

7.1.3 实验室设立单位的法定代表人负责本单位实验室的生物安全管理，建立生物安全管理体系，落实生物安全管理责任部门或责任人；定期召开生物安全管理会议，对实验室生物安全相关的重大事项做出决策；批准和发布实验室生物安全管理体系文件。

7.1.4 实验室生物安全管理责任部门负责组织制定和修订实验室生物安全管理体系文件；对实验项目进行审查和风险控制措施的评估；负责实验室工作人员的健康监测的管理；组织生物安全培训与考核，并评估培训效果；监督生物安全管理体系的运行落实。

7.1.5 实验室负责人为实验室生物安全第一责任人，全面负责实验室生物安全工作。负责实验项目计划、方案和操作规程的审查（参见附录 B） ；决定并授权人员进入实验室；负责实验室活动的管理；纠

正违规行为并有权做出停止实验的决定。指定生物安全负责人，赋予其监督所有活动的职责和权力，包括制定、维持、监督实验室安全计划的责任，阻止不安全行为或活动的权力。

7.1.6 与实验室生物安全管理有关的关键职位均应指定职务代理人。

7.2 人员管理

7.2.1 实验室应配备足够的人力资源以满足实验室生物安全管理体系的有效运行，并明确相关部门和人员的职责。

7.2.2 实验室管理人员和工作人员应熟悉生物安全相关政策、法律、法规和技术规范，有适合的教育背景、工作经历，经过专业培训，能胜任所承担的工作；实验室管理人员还应具有评价、纠正和处置违反安全规定行为的能力。

7.2.3 建立工作人员准入及上岗考核制度，所有与实验活动相关的人员均应经过培训，经考核合格后取得相应的上岗资质；动物实验人员应持有有效实验动物上岗证及所从事动物实验操作专业培训证明。

7.2.4 实验室或者实验室的设立单位应每年定期对工作人员培训（包括岗前培训和在岗培训） ，并对培训效果进行评估。

7.2.5 从事高致病性病原微生物实验活动的人员应每半年进行一次培训，并记录培训及考核情况。

7.2.6 实验室应保证工作人员充分认识和理解所从事实验活动的风险，必要时，应签署知情同意书。

7.2.7 实验室工作人员应在身体状况良好的情况下进入实验区工作。若出现疾病、疲劳或其他不宜进行实验活动的情况，不应进入实验区。

7.2.8 实验室设立单位应该与具备感染科的综合医院建立合作机制，定期组织在医院进行工作人员体检，并进行健康评估，必要时，应进行预防接种。

7.2.9 实验室工作人员出现与其实验活动相关的感染临床症状或者体征时，实验室负责人应及时向上级主管部门和负责人报告，立即启动实验室感染应急预案。由专车、专人陪同前往定点医疗机构就诊。并向就诊医院告知其所接触病原微生物的种类和危害程度。

7.2.10 应建立实验室人员（包括实验、管理和维保人员）的技术档案、健康档案和培训档案，定期评估实验室人员承担相应工作任务的能力；临时参与实验活动的外单位人员应有相应记录。

7.2.11 实验室人员的健康档案应包括但不限于：

a) 岗位风险说明及知情同意书（必要时）；

b) 本底血清样本或特定病原的免疫功能相关记录；

c) 预防免疫记录（适用时）；

d) 健康体检报告；

e) 职业感染和职业禁忌症等资料；

f) 与实验室安全相关的意外事件、事故报告等。

7.3 菌（毒）种及感染性样本的管理

7.3.1 实验室菌（毒）种及感染性样本保存、使用管理，应依据国家生物安全的有关法规，制定选择、购买、采集、包装、运输、转运、接收、查验、使用、处置和保藏的政策和程序。

7.3.2 实验室应有 2 名工作人员负责菌（毒）种及感染性样本的管理。

7.3.3 实验室应具备菌（毒）种及感染性样本适宜的保存区域和设备。

7.3.4 保存区域应有消防、防盗、监控、报警、通风和温湿度监测与控制等设施；保存设备应有防盗和温度监测与控制措施。高致病性病原微生物菌（毒）种及感染性样本的保存应实行双人双锁。

7.3.5 保存区域应有菌（毒）种及感染性样本检查、交接、包装的场所和生物安全柜等设备。

7.3.6 保存菌（毒）种及感染性样本容器的材质、质量应符合安全要求，不易破碎、爆裂、泄露。

7.3.7 保存容器上应有牢固的标签或标识，标明菌（毒）种及感染性样本的编号、日期等信息。

7.3.8 菌（毒）种及感染性样本在使用过程中应有专人负责，入库、出库及销毁应记录并存档。

7.3.9 实验室应当将在研究、教学、检测、诊断、生产等实验活动中获得的有保存价值的各类菌（毒）

种或感染性样本送交保藏机构进行鉴定和保藏。

7.3.10 高致病性病原微生物相关实验活动结束后，应当在 6 个月内将菌（毒）种或感染性样本就地销毁或者送交保藏机构保藏。

7.3.11 销毁高致病性病原微生物菌（毒）种或感染性样本时应采用安全可靠的方法，并应当对所用方法进行可靠性验证。销毁工作应当在与拟销毁菌（毒）种相适应的生物安全实验室内进行，由两人共同操作，并应当对销毁过程进行严格监督和记录。

7.3.12 病原微生物菌（毒）种或感染性样本的保存应符合国家有关保密要求。

7.4 设施设备运行维护管理

7.4.1 实验室应有对设施设备（包括个体防护装备）管理的政策和运行维护保养程序，包括设施设备性能指标的监控、日常巡检、安全检查、定期校准和检定、定期维护保养等（参见附录 C） 。

7.4.2 实验室设施设备性能指标应达到国家相关标准的要求和实验室使用的要求。

7.4.3 设施设备应由经过授权的人员操作和维护。

7.4.4 设施设备维护、修理、报废等需移出实验室，移出前应先进行消毒去污染。

7.4.5 如果使用防护口罩、防护面罩等个体呼吸防护装备，应做个体适配性测试。

7.4.6 应依据制造商的建议和使用说明书使用和维护实验室设施设备，说明书应便于有关人员查阅。

7.4.7 应在设备显著部位标示其唯一编号、校准或验证日期、下次校准或验证日期、准用或停用状态。

7.4.8 应建立设施设备档案，内容应包括（但不限于）：

a) 制造商名称、型式标识、系列号或其他唯一性标识；

b) 验收标准及验收记录；

c) 接收日期和启用日期；

d) 接收时的状态（新品、使用过、修复过）；

e) 当前位置；

f) 制造商的使用说明或其存放处；

g) 维护记录和年度维护计划；

h) 校准（验证）记录和校准（验证）计划；

i) 任何损坏、故障、改装或修理记录；

j) 服务合同；

k) 预计更换日期或使用寿命；

l) 安全检查记录。

7.4.9 实验室所有设备、仪器，未经实验室负责人许可不得擅自移动。

7.4.10 实验室内的所有物品（包括仪器设备和实验室产品等）， 应经过消毒处理后方可移出该实验室。

7.4.11 实验室应在电力供应有保障、设施和设备运转正常情况下使用。

7.4.12 应实时监测实验室通风系统过滤器阻力，当影响到实验室正常运行时应及时更换。

7.4.13 生物安全柜、压力蒸汽灭菌器、动物隔离设备等应由具备相应资质的机构按照相应的检测规程进行检定。实验室应有专门的程序对服务机构及其服务进行评估并备案。

7.4.14 高效空气过滤器应由经过培训的专业人员进行更换，更换前应进行原位消毒，确认消毒合格后，按标准操作流程进行更换。新高效空气过滤器，应进行检漏，确认合格后方可使用。

7.4.15 应根据实验室使用情况对防护区进行消毒。

7.4.16 如安装紫外灯，应定期监测紫外灯的辐射强度。

7.4.17 应定期对压力蒸汽灭菌器等消毒、灭菌设备进行效果监测与验证（参见附录 D） 。

7.5 实验室活动的管理

7.5.1 实验活动应依法开展，并符合有关主管部门的相关规定。

7.5.2 实验室的设立单位及其主管部门负责实验室日常活动的管理，承担建立健全安全管理的制度，检查、维护实验设施、设备，控制实验室感染的职责。

7.5.3 实验室应有计划、申请、批准、实施、监督和评估实验活动的制度和程序。

7.5.4 实验活动应在与其防护级别相适应的生物安全实验室内开展。

7.5.5 一级和二级生物安全实验室应当向设区的市级人民政府卫生计生主管部门备案；三级和四级生物安全实验室应当通过实验室国家认可，并向所在地的县（区）级人民政府环境保护主管部门和公安部门备案。

7.5.6 三级和四级生物安全实验室从事高致病性病原微生物实验活动， 应取得国家卫生和计生行政主管部门颁发的《高致病性病原微生物实验室资格证书》。

7.5.7 取得《高致病性病原微生物实验室资格证书》的三级和四级生物安全实验室需要从事某种高致病性病原微生物或者疑似高致病性病原微生物实验活动的，还应当报省级以上卫生和计生行政主管部门批准。

7.5.8 二级生物安全实验室从事高致病性病原微生物实验室活动除应满足《人间传染的病原微生物名录》对实验室防护级别的要求外还应向省级卫生和计生行政主管部门申请。

7.5.9 实验室使用我国境内未曾发现的高致病性病原微生物菌（毒）种或样本和已经消灭的病原微生物菌（毒）种或样本、《人间传染的病原微生物名录》规定的第一类病原微生物菌（毒）种或样本、或国家卫生和计划生育委员会规定的其他菌（毒）种或样本，应当经国家卫生和计划生育委员会批准；使用其他高致病性菌（毒）种或样本，应当经省级人民政府卫生计生行政主管部门批准；使用第三、四类病原微生物菌（毒）种或样本，应当经实验室所在法人机构批准。

7.5.10 实验活动应当严格按照实验室技术规范、操作规程进行。实验室负责人应当指定专人监督检查实验活动。

7.5.11 从事高致病性病原微生物相关实验活动应当有 2 名以上的工作人员共同进行。从事高致病性病原微生物相关实验活动的实验室工作人员或者其他有关人员，应当经实验室负责人批准。

7.5.12 在同一个实验室的同一个独立安全区域内，只能同时从事一种高致病性病原微生物的相关实验活动。

7.5.13 实验室应当建立实验档案，记录实验室使用情况和安全监督情况。实验室从事高致病性病原微生物相关实验活动的实验档案保存期不得少于 20 年。

7.6 生物安全监督检查

7.6.1 实验室的设立单位及其主管部门应当加强对实验室日常活动的管理，定期对有关生物安全规定的落实情况进行检查。

7.6.2 实验室应建立日常监督、定期自查和管理评审制度，及时消除隐患，以保证实验室生物安全管理体系有效运行，每年应至少系统性地检查一次，对关键控制点可根据风险评估报告适当增加检查频率。

7.6.3 实验室应制定监督检查计划，应将高致病性病原微生物菌（毒）种和样本的操作、菌（毒）种及样本保管、实验室操作规范、实验室行为规范、废物处理等作为监督的重点，同时检查风险控制措施的有效性，包括对实验人员的操作、设备的使用、新方法的引入以及大量样本检测等内容。

7.6.4 对实验活动进行不定期监督检查，对影响安全的主要要素进行核查,以确保生物安全管理体系运行的有效性。

7.6.5 实验室监督检查的内容包括但不限于：

a) 病原微生物菌（毒）种和样本操作的规范性；

b) 菌（毒）种及样本保管的安全性；

c) 设施设备的功能和状态；

d) 报警系统的功能和状态；

e) 应急装备的功能及状态；

f) 消防装备的功能及状态；

g) 危险物品的使用及存放安全；

h) 废物处理及处置的安全；

i) 人员能力及健康状态；

j) 安全计划的实施；

k) 实验室活动的运行状态；

l) 不符合规定操作的及时纠正；

m) 所需资源是否满足工作要求；

n) 监督检查发现问题的整改情况。

7.6.6 为保证实验室生物安全监督检查工作的质量，应依据事先制定适用于不同工作领域的核查表实施。

7.6.7 当发现不符合规定的工作、发生事件或事故时，应立即查找原因并评估后果；必要时，停止工作。在监督检查过程中发现的问题要立即采取纠正措施，并监控所取得的效果，以确保所发现的问题得以有效解决。

7.7 消毒和灭菌

7.7.1 实验室应根据操作的病原微生物种类、污染的对象和污染程度等选择适宜的消毒和灭菌方法，以确保消毒效果。

7.7.2 实验室根据菌（毒）种、生物样本及其他感染性材料和污染物，可选用压力蒸汽灭菌方法或有效的化学消毒剂处理。实验室按规定要求做好消毒与灭菌效果监测。

7.7.3 实验使用过的防护服、一次性口罩、手套等应选用压力蒸汽灭菌方法处理。

7.7.4 医疗废物等应经压力蒸汽灭菌方法处理后再按相关实验室废物处置方法处理。

7.7.5 动物笼具可经化学消毒或压力蒸汽灭菌处理，局部可用消毒剂擦拭消毒处理。

7.7.6 实验仪器设备污染后可用消毒液擦拭消毒。必要时，可用环氧乙烷、甲醛熏蒸消毒。

7.7.7 生物安全柜、工作台面等在每次实验前后可用消毒液擦拭消毒。

7.7.8 污染地面可用消毒剂喷洒或擦拭消毒处理。

7.7.9 感染性物质等溢洒后，应立即使用有效消毒剂处理。

7.7.10 实验人员需要进行手消毒时，应使用消毒剂擦拭或浸泡消毒，再用肥皂洗手、流水冲洗。

7.7.11 选用的消毒剂、消毒器械应符合国家相关规定。

7.7.12 实验室应确保消毒液的有效使用，应监测其浓度，应标注配制日期、有效期及配制人等。

7.7.13 实施消毒的工作人员应佩戴个体防护装备。

7.8 实验废物处置

7.8.1 实验室废物处理和处置的管理应符合国家或地方法规和标准的要求。

7.8.2 实验室废物处置应由专人负责。

7.8.3 实验室废物的处置应符合《医疗废物管理条例》的规定。实验室废物的最终处置应交由经当地环保部门资质认定的医疗废物处理单位集中处置。

7.8.4 实验室废物的处置应有书面记录，并存档。

7.9 实验室感染性物质运输

7.9.1 实验室应制定感染性及潜在感染性物质运输的规定和程序，包括在实验室内传递、实验室所在机构内部转运及机构外部的运输，应符合国家和国际规定的要求。感染性物质的国际运输还应依据并遵守国家出入境的相关规定。

7.9.2 实验室应确保具有运输资质和能力的人员负责感染性及潜在感染性物质运输。

7.9.3 感染性及潜在感染性物质运输应以确保其属性、防止人员感染及环境污染的方式进行，并有可靠的安保措施。必要时，在运输过程中应备有个体防护装备及有效消毒剂。

7.9.4 感染性及潜在感染性物质应置于被证实和批准的具有防渗漏、防溢洒的容器中运输。

7.9.5 机构外部的运输，应按照国家、 国际规定及标准使用具有防渗漏、防溢洒、防水、防破损、防外泄、耐高温、耐高压的三层包装系统，并应有规范的生物危险标签、标识、警告用语和提示用语等。

7.9.6 应建立并维持感染性及潜在感染性物质运输交接程序，交接文件至少包括其名称、性质、数量、交接时包装的状态、交接人、收发交接时间和地点等，确保运输过程可追溯。

7.9.7 感染性及潜在感染性物质的包装以及开启，应当在符合生物安全规定的场所中进行。运输前后均应检查包装的完整性，并核对感染性及潜在感染性物质的数量。

7.9.8 高致病性病原微生物菌（毒）种或样本的运输,应当按照国家有关规定进行审批。地面运输应有专人护送，护送人员不得少于两人

7.9.9 应建立感染性及潜在感染性物质运输应急预案。运输过程中被盗、被抢、丢失、泄漏的，承运单位、护送人应当立即采取必要的处理和控制措施，并按规定向有关部门报告。

7.10 应急预案和意外事故的处置

7.10.1 实验室应制定应急预案和意外事故的处置程序，包括生物性、化学性、物理性、放射性等意外事故，以及火灾、水灾、冰冻、地震或人为破坏等突发紧急情况等。

7.10.2 应急预案应至少包括组织机构、应急原则、人员职责、应急通讯、个体防护、 应对程序、应急设备、撤离计划和路线、污染源隔离和消毒、人员隔离和救治、现场隔离和控制、风险沟通等内容。

7.10.3 在制定的应急预案中应包括消防人员和其他紧急救助人员。在发生自然灾害时，应向救助人员告知实验室建筑内和∕或附近建筑物的潜在风险，只有在受过训练的实验室工作人员的陪同下，其他人员才能进入相关区域。

7.10.4 应急预案应得到实验室设立单位管理层批准。实验室负责人应定期组织对预案进行评审和更新。

7.10.5 从事高致病性病原微生物相关实验活动的实验室制定的实验室感染应急预案应向所在地的省、自治区、直辖市卫生主管部门备案。

7.10.6 实验室应对所有人员进行培训， 确保人员熟悉应急预案。每年应至少组织所有实验室人员进行一次演练。

7.10.7 实验室应根据相关法规建立实验室事故报告制度。

7.10.8 实验室发生意外事故，工作人员应按照应急预案迅速采取控制措施，同时应按制度及时报告，任何人员不得瞒报。

7.10.9 事故现场紧急处理后，应及时记录事故发生过程和现场处置情况。

7.10.10 实验室负责人应及时对事故作出危害评估并提出下一步对策。对事故经过和事故原因、责任进行调查分析，形成书面报告。报告应包括事故的详细描述、原因分析、影响范围、预防类似事件发生的建议及改进措施。所有事故报告应形成档案文件并存档。

7.10.11 事故报告应经所在机构管理层、生物安全委员会评估。

7.11 实验室生物安全保障

7.11.1 实验室设立单位应建立健全安全保卫制度，采取有效的安全措施，以防止病原微生物菌（毒）种及样本丢失、被窃、滥用、误用或有意释放。实验室发生高致病性病原微生物菌（毒）种或样本被盗、被抢、丢失、泄漏的，应当依照相关规定及时进行报告。

7.11.2 实验室设立单位根据实验室工作内容以及具体情况，进行风险评估，制定生物安全保障规划，进行安全保障培训；调查并纠正实验室生物安全保障工作中的违规情况。

7.11.3 从事高致病性病原微生物相关实验活动的实验室应向当地公安机关备案，接受公安机关对实验室安全保卫工作的监督指导。

7.11.4 应建立高致病性病原微生物实验活动的相关人员综合评估制度，考察上述人员在专业技能、身心健康状况等方面是否胜任相关工作。

7.11.5 建立严格的实验室人员出入管理制度。

7.11.6 适用时，应按照国家有关规定建立相应的保密制度。
