# Supplementary material for: Mining and quantitative evaluation of the laboratory biosafety policy in China
Source: PLoS One. 2025 Aug 12;20(8):e0328923. doi: 10.1371/journal.pone.0328923 (PMC12342282; doi:10.1371/journal.pone.0328923)
Supplement: S2 Appendix — (DOCX) [file pone.0328923.s002.docx]

**Appendix**

**S2 137 laboratory biosafety management policies included in the analysis**

| **No** | **Policy Document** | **Issuing Date** | **Issuing Department** |
| --- | --- | --- | --- |
| 1 | Law of the People's Republic of China on Animal Epidemic Prevention | 3-Jul-97 | Member of the Standing Committee of the National People's Congress |
| 2 | Regulation on the Bio-safety Management of Patgogenic Microbe Labs | 12-Nov-04 | The State Council |
| 3 | Measures for the Construction Review of High Level Pathogenic Microbiology Laboratory | 24-Jun-11 | Ministry of Science and Technology |
| 4 | Biosecurity Law of the People's Republic of China | 17-Oct-20 | Member of the Standing Committee of the National People's Congress |
| 5 | Measures for biosafety environmental management of pathogenic microorganism laboratories | 1-May-06 | Ministry of Ecology and Environment |
| 6 | Measures for the Administration and Approval of Biosafety Management in Laboratories Handling Highly Pathogenic Animal Pathogens | 20-May-05 | Ministry of Agriculture and Rural Affairs |
| 7 | Measures for the Administration and Approval of Biosafety in Laboratories and Experimental Activities Involving Highly Pathogenic Microorganisms of Human Infection | 15-Aug-06 | National Health Commission |
| 8 | Notice on issurance of the 14th Five-Year Plan for National Health | 27-Apr-22 | Office of the State Council |
| 9 | Measures for the Preservation and Management of Animal Pathogenic Microorganisms (Bacterial and Viral Strains) | 26-Nov-08 | Ministry of Agriculture and Rural Affairs |
| 10 | Guiding Opinions on reforming and improving the Comprehensive Supervision System of the medical and health industry | 18-Jul-18 | Office of the State Council |
| 11 | Opinions on Strengthening the Safety Protection of Personnel in the Prevention and Control of Infectious Diseases | 6-Jan-15 | Office of the State Council |
| 12 | Notice on Promulgation of Several Policies for Promoting the Accelerated Development of the Biological Industry | 2-Jun-09 | Office of the State Council |
| 13 | Measures for the Administration of Institutions Preserving Pathogenic Microorganisms (Toxins) of Human Infections | 16-Jun-09 | National Health Commission |
| 14 | Notice on Forwarding the "Eleventh Five-Year Plan" for the Development of the Biological Industry of the National Development and Reform Commission | 8-Apr-07 | Office of the State Council |
| 15 | Notice on Issuing the Regulations on the Main Responsibilities, Internal Organizations, and Staffing of the National Health and Family Planning Commission | 9-Jun-13 | Office of the State Council |
| 16 | Notice on the Issuance of the "Twelfth Five-Year Plan" for the Development of Health Services | 8-Oct-12 | The State Council |
| 17 | Notice on Forwarding the Planning of Vaccine Supply System Construction of the National Development and Reform Commission and Other Departments | 22-Dec-11 | Office of the State Council |
| 18 | Regulations on the Transportation of Highly Pathogenic Microorganisms (Toxins) or Samples that Can Infect Humans | 28-Dec-05 | National Health Commission |
| 19 | Notice on the Issuance of the Regulations on the Main Responsibilities, Internal Organizations and Personnel Establishment of the Ministry of Health | 10-Jul-08 | Office of the State Council |
| 20 | Notice on the Issuance of the Plan for the Development of the Biological Industry | 29-Dec-12 | The State Council |
| 21 | Guiding Opinions on Strengthening the Registration of First-level and Second-level Animal Pathogenic Microorganism Laboratories | 17-Jan-24 | Ministry of Agriculture and Rural Affairs |
| 22 | Notice on Strengthening the Bio-safety Administration of Animal Pathogenic Microbe Laboratories | 9-Feb-20 | Ministry of Agriculture and Rural Affairs, Ministry of Education, Ministry of Science and Technology, National Health Commission, General Administration of Customs, National Forestry and Grassland Administration, Chinese Academy of Science |
| 23 | Notice on the Establishment of the Fourth National Expert Committee on Biosafety of Pathogenic Microorganism Laboratories | 7-Sep-21 | National Health Commission, Ministry of Agriculture and Rural Affairs |
| 24 | Notice on Further Strengthening the Biosafety Management of Animal Pathogenic Microorganism Laboratories | 11-May-21 | Ministry of Agriculture and Rural Affairs |
| 25 | Notice on Carrying Out the Special Inspection of Biosafety in National Animal Pathogenic Microorganism Laboratories in 2020 | 18-May-20 | Ministry of Agriculture and Rural Affairs |
| 26 | Notice on the Replacement of the National Expert Committee for Biosafety Evaluation of Human Pathogenic Microorganisms Laboratories | 17-Jan-21 | National Health Commission |
| 27 | Notice on the Issuance of the "Guidance on Laboratory Biosafety for Novel Coronavirus (Second Edition)" | 23-Jan-20 | National Health Commission |
| 28 | Notice on the Establishment of the Expert Committee for Biosafety Evaluation of Animal Pathogenic Microorganisms Laboratories under the Ministry of Agriculture and Rural Affairs | 26-Dec-19 | Ministry of Agriculture and Rural Affairs |
| 29 | Notice on Carrying out the Special Inspection of Biosafety in Animal Pathogenic Microorganism Laboratories in 2016 | 20-Jun-16 | Ministry of Agriculture and Rural Affairs |
| 30 | Notice on Strengthening the Review of the Construction of High-level Pathogenic Microorganism Laboratories | 3-Aug-11 | Ministry of Science and Technology |
| 31 | Notice on Further Improving the Supervision of Biosafety in Animal Pathogenic Microorganism Laboratories | 8-Mar-16 | Ministry of Agriculture and Rural Affairs |
| 32 | Notice on Organizing and Conducting On-site Environmental Inspections of Pathogenic Microorganism Laboratories | 21-Jun-07 | Ministry of Ecology and Environment |
| 33 | Notice on Carrying out the Special Inspection of Biosafety in National Animal Pathogenic Microorganism Laboratories in 2018 | 12-Apr-18 | Ministry of Agriculture and Rural Affairs |
| 34 | Veterinary Laboratory Biosafety Guidelines | 15-Oct-03 | Ministry of Agriculture and Rural Affairs |
| 35 | Notice on Further Improving the Biosafety Management of Pathogenic Microorganism Laboratories | 5-Sep-06 | National Health Commission |
| 36 | Notice on the Release of the "Service Guide for Administrative Examination and Approval of High-level Pathogenic Microorganism Laboratory Construction Review" | 15-Jun-15 | Ministry of Science and Technology |
| 37 | Notice on the Issuance of the "Working Procedures for the Approval of Qualifications for Highly Pathogenic Microorganism Laboratories" | 15-May-07 | National Health Commission |
| 38 | Notice on Conducting the Special Inspection of Biosafety in National High-Level Animal Pathogenic Microorganism Laboratories in 2019 | 12-Apr-19 | Ministry of Agriculture and Rural Affairs |
| 39 | Notice on the Appointment of Members of the Biosafety Review Committee for High-Level Pathogenic Microorganism Laboratories | 9-May-12 | Ministry of Science and Technology |
| 40 | Notice on biosafety supervision after the cancellation of the "Approval of highly pathogenic Microorganism Laboratory activity Qualification" | 31-Oct-17 | National Health Commission |
| 41 | Notice on issues related to the environmental impact assessment qualification of pathogenic microorganism laboratory project | 14-Feb-06 | Ministry of Ecology and Environment |
| 42 | Notice on the supervision work after the cancellation of qualification identification of highly pathogenic microorganisms experimental activities | 1-Nov-17 | Ministry of Agriculture and Rural Affairs |
| 43 | Notice on Further Regulating the Approval Process for Experimental Activities Involving Highly Pathogenic Animal Pathogens | 12-Dec-08 | Ministry of Agriculture and Rural Affairs |
| 44 | Guiding Opinions on Improving the Resource Sharing of High-Level Animal Biosafety Laboratories | 2-Jun-22 | Ministry of Agriculture and Rural Affairs |
| 45 | Notice on the issuance of Implementation Guidelines for Regional Novel Coronavirus nucleic acid Testing Organizations (third edition) | 18-Mar-22 | The Joint Prevention and Control Mechanism of the State Council |
| 46 | Notice on the issuance of the 14th Five-Year Health Standardization Work Plan | 11-Jan-22 | National Health Commission |
| 47 | Notice on the issuance of the "14th Five-Year Plan" for Bioeconomic Development | 20-Dec-21 | National Development and Reform Commission |
| 48 | Notice on the establishment of the National Expert Committee on the Preservation and Management of animal Pathogenic Microorganisms (viruses) | 3-Jun-21 | Ministry of Agriculture and Rural Affairs |
| 49 | Notice on issuing Guidelines on the Routine Prevention and Control of COVID-19 in Blood Stations | 25-Mar-21 | National Health Commission, Ministry of National Defense of the People's Republic of China |
| 50 | Notice on "Matters Related to the Construction and Management of Biosafety Level III Laboratories" | 27-Jun-03 | Ministry of Science and Technology |
| 51 | Notice on the issuance of the Management Measures for Large-scale Novel Coronavirus Nucleic Acid Testing Laboratories (Trial) | 17-Feb-21 | The Joint Prevention and Control Mechanism of the State Council |
| 52 | Notice on the issuance of the Working Manual of Novel Coronavirus Nucleic Acid Detection in Medical Institutions (Trial Second edition) | 28-Dec-20 | The Joint Prevention and Control Mechanism of the State Council |
| 53 | Notice on issuing the Guidelines for the Prevention and Control of COVID-19 in Blood Stations in Autumn and Winter | 13-Nov-20 | National Health Commission, Ministry of National Defense of the People's Republic of China |
| 54 | Urgent Notice on Strengthening SARS virus strains, centralized management of human specimens, and ensuring the biosafety of virus laboratories and custodian units | 17-Dec-03 | National Health Commission |
| 55 | Notice on the issuance of Technical Guidelines for the Construction of Inspection Laboratories in Medical and Health Institutions (for Trial Implementation) | 4-Sep-20 | National Health Commission, National Development and Reform Commission |
| 56 | Notice on the issuance of the Work Plan for Further Promoting Capacity Building for Nucleic Acid Detection of the Novel Coronavirus | 27-Aug-20 | The Joint Prevention and Control Mechanism of the State Council |
| 57 | Notice on issuing the Interim Measures for the Administration of Medical Laboratory Testing | 1-Aug-20 | The Joint Prevention and Control Mechanism of the State Council |
| 58 | Notice on the issuance of the Working Manual of Novel Coronavirus Nucleic Acid Detection in Medical Institutions (Trial) | 10-Jul-20 | The Joint Prevention and Control Mechanism of the State Council |
| 59 | Notice on Further Strengthening Laboratory Biosafety supervision and Management in the routine prevention and control of the novel coronavirus (COVID-19) epidemic | 6-Jul-20 | National Health Commission |
| 60 | Notice on quality control of nucleic acid testing of the novel coronavirus under normal epidemic prevention and control | 1-Jun-20 | The Joint Prevention and Control Mechanism of the State Council |
| 61 | Notice on further COVID-19 testing during the epidemic period | 18-Apr-20 | The Joint Prevention and Control Mechanism of the State Council |
| 62 | Notice on issuing the Guiding Opinions on Further Strengthening the Construction of Internal Control in Public Hospitals | 18-Dec-23 | Ministry of Finance, National Health Commission, National Healthcare Security Administration, State Administration of Traditional Chinese Medicine |
| 63 | Notice on Strengthening the Management of National African Swine Fever Related Laboratories | 22-Apr-19 | Ministry of Agriculture and Rural Affairs |
| 64 | Notice on the issuance of Technical Guidelines for the Prevention and Control of Novel Coronavirus Infection in Medical Institutions (Third edition) | 8-Sep-21 | The Joint Prevention and Control Mechanism of the State Council |
| 65 | Notice on the issuance of "Key Points of Veterinary Work 2018" | 14-Feb-18 | Ministry of Agriculture and Rural Affairs |
| 66 | Notice on the issuance of the "Guidelines for the Implementation of the Organization of the New Coronavirus Nucleic Acid Testing for All Staff (Second edition)" | 8-Sep-21 | The Joint Prevention and Control Mechanism of the State Council |
| 67 | Notice on the issuance of the Special Inspection Plan for the Prevention and Treatment of Infectious Diseases and the Supervision and Law Enforcement of Infection Prevention and Control in TCM Medical Institutions | 15-Sep-17 | State Administration of Traditional Chinese Medicine |
| 68 | Notice on the issuance of the High-level Biosafety Laboratory System Construction Plan (2016-2025) | 8-Nov-16 | National Development and Reform Commission, Ministry of Science and Technology |
| 69 | Notice on issuing the Guidelines for the Organization and Implementation of the New Coronavirus Nucleic Acid Testing for All Staff | 7-Feb-21 | The Joint Prevention and Control Mechanism of the State Council |
| 70 | Notice on biosafety supervision of highly pathogenic microorganism research projects | 15-Jul-16 | National Health Commission |
| 71 | Notice on Further strengthening the management of the National Veterinary Reference Laboratory | 7-Apr-16 | Ministry of Agriculture and Rural Affairs |
| 72 | Notice on improving the prevention and control of COVID-19 in maternal and child health care institutions in autumn and winter | 22-Oct-20 | National Health Commission |
| 73 | Notice on encouraging CDC institutions to carry out nucleic acid testing services for the novel coronavirus | 11-Sep-20 | The Joint Prevention and Control Mechanism of the State Council |
| 74 | Notice on the issuance of the "Health Supervision Work standards for the Prevention and Treatment of Infectious Diseases" | 14-Jul-14 | National Health Commission |
| 75 | Notice on the routine prevention and control of traditional Chinese medicine | 15-May-20 | State Administration of Traditional Chinese Medicine |
| 76 | Notice on the investigation of Biosafety in laboratories of food and drug inspection and testing institutions | 20-Jun-14 | National Medical Products Administration |
| 77 | Notice on strengthening the medical treatment of severe cases of pneumonia infected by novel coronavirus | 22-Jan-20 | National Health Commission |
| 78 | Reply to Recommendation No. 7088 of the Second Session of the 13th National People's Congress | 15-Aug-19 | Ministry of Agriculture and Rural Affairs |
| 79 | Notice on Strengthening supervision of experimental activities of "Swine influenza" A/H1N1 virus | 30-Apr-09 | Ministry of Agriculture and Rural Affairs |
| 80 | Notice on the issuance of the Ministry of Agriculture 2009 Veterinary Work Points | 1-Feb-09 | Ministry of Agriculture and Rural Affairs |
| 81 | Notice on transport of animal strains, samples, disease materials, etc | 29-Nov-08 | Civil Aviation Administration of China |
| 82 | Notice on Doing a Good Job in the Field of Veterinary Medicine During the 2018 Spring Festival | 1-Feb-18 | Ministry of Agriculture and Rural Affairs |
| 83 | Notice on the issuance of Guidelines for Hospital Management Evaluation (2008 edition) | 13-May-08 | National Health Commission |
| 84 | Notice on the medical treatment of human infection with H7N9 bird flu | 24-Jan-17 | National Health Commission |
| 85 | Notice on Strengthening the work of Agricultural Production Safety | 10-May-08 | Ministry of Agriculture and Rural Affairs |
| 86 | Notice on issuing key points of health and family planning work in 2017 | 13-Jan-17 | National Health Commission |
| 87 | Notice on prevention of safety risks in the field of veterinary medicine | 17-Dec-16 | Ministry of Agriculture and Rural Affairs |
| 88 | Opinions on Implementing Key actions to develop modern agriculture | 13-Mar-07 | Ministry of Agriculture and Rural Affairs |
| 89 | Notice on the issuance of the Key Points of Veterinary Work in 2016 | 25-Jan-16 | Ministry of Agriculture and Rural Affairs |
| 90 | Notice on the issuance of the Key Points of Health and Family Planning Work in 2016 | 19-Jan-16 | National Health Commission |
| 91 | CNAS-CL05 Criteria for Accreditation of Laboratory Biosafety | 1-Jun-06 | China National Accreditation Service for Conformity Assessment |
| 92 | Notice on the Issuance of the "National Plan for the Elimination of Small Ruminant Peste des Petits Ruminants (PPR) (2016-2020)" | 24-Dec-15 | Ministry of Agriculture and Rural Affairs |
| 93 | Notice on building laboratory biosafety capacity in primary health institutions | 6-Feb-06 | National Health Commission |
| 94 | Notice on the issuance of "Key Points of Health and Family Planning Work in 2015" | 14-Jan-15 | National Health Commission |
| 95 | Notice on the issuance of the Ministry of Health and Agriculture on the Cooperation Mechanism for the Prevention and Treatment of Zoonotic Infectious Diseases | 20-Sep-05 | National Health Commission |
| 96 | Urgent Notice on Strengthening the research and management of Highly Pathogenic Microorganisms | 30-May-05 | Ministry of Education, Ministry of Science and Technology, Ministry of Agriculture and Rural Affairs |
| 97 | Highly pathogenic animal pathogenic microorganism species or sample transport packaging specification | 24-May-05 | Ministry of Agriculture and Rural Affairs |
| 98 | Notice on the issuance of the Guidelines for Hospital Management Evaluation (Trial) | 17-Mar-05 | National Health Commission |
| 99 | Notice on the comparison of laboratory testing capacity of veterinary system in 2014 | 28-Mar-14 | Ministry of Agriculture and Rural Affairs |
| 100 | Notice on the issuance of the Key Points of Health Work 2005 | 24-Jan-05 | National Health Commission |
| 101 | Notice on Further Improving the Medical Treatment of Human Infections with H7N9 Avian Influenza | 10-Apr-13 | National Health Commission |
| 102 | Notice on Strengthening the Prevention and Control of Human Infections with H7N9 Avian Influenza | 3-Apr-13 | National Health Commission |
| 103 | Notice on Carrying Out the Comparison of Laboratory Testing Capabilities in the Veterinary System in 2013 | 15-Mar-13 | Ministry of Agriculture and Rural Affairs |
| 104 | Notice on the Issuance of the "Measures for the Administration of Clinical Laboratories in Medical Institutions" | 27-Feb-06 | National Health Commission |
| 105 | Notice on the Issuance of Key Points for Health Work in 2012 | 30-Jan-12 | National Health Commission |
| 106 | Notice on the Issuance of the "Key Points of Veterinary Work in the Ministry of Agriculture for 2010" | 10-Feb-10 | Ministry of Agriculture and Rural Affairs |
| 107 | Notice on the Issuance of the Key Points of Health Work for 2010 | 1-Jan-10 | National Health Commission |
| 108 | Notice on preparations for the prevention and treatment of influenza A (H1N1) pandemic with traditional Chinese medicine | 17-Sep-09 | State Administration of Traditional Chinese Medicine |
| 109 | Notice on preparations for medical treatment of influenza A (H1N1) pandemic | 8-Sep-09 | National Health Commission |
| 110 | Notice on the Issuance of Key Points of Health Work for 2009 | 24-Jan-09 | National Health Commission |
| 111 | Notice on the Issuance of the "Opinions on the Counterpart Support Work for Disease Prevention and Control in the Wenchuan Earthquake Disaster Area" | 7-Aug-08 | National Health Commission |
| 112 | Notice on Further Ensuring Effective Supply and Quality Safety of Agricultural Products During the Olympic Games | 21-Jul-08 | Ministry of Agriculture and Rural Affairs |
| 113 | Notice on the Issuance of Guidelines for Prevention and Control of Six Infectious Diseases Including Ebola Hemorrhagic Fever and Clinical Diagnosis and Treatment Protocols | 12-Jul-08 | National Health Commission |
| 114 | Notice on the Issuance of the Key Points of Health Work in 2008 | 23-Jan-08 | National Health Commission |
| 115 | Notice on the Issuance of the "Strategy for Prevention and Control of Acute Infectious Diseases Outbreaks" | 20-Jun-07 | National Health Commission |
| 116 | Notice on the Issuance of the "Key Points of Scientific and Technological Work for Social Development (2006-2010)" | 24-Apr-07 | Ministry of Science and Technology |
| 117 | Opinions on Implementing the Several Opinions of the Central Committee of the Communist Party of China and The State Council on Promoting the Construction of a New Socialist Countryside | 18-Jan-06 | Ministry of Agriculture and Rural Affairs |
| 118 | Notice on the issuance of the Key Points of Health Work in 2006 | 31-Dec-05 | National Health Commission |
| 119 | Notice on the issuance of the National Work Plan for the Prevention and Control of SARS and Influenza in the Winter and Spring of 2004-2005 | 28-Oct-04 | National Health Commission |
| 120 | Notice on the Issuance of Regulations for the Management of Human Sample Resources for Infectious Atypical Pneumonia | 18-Jun-03 | National Health Commission |
| 121 | Notice on Strengthening the Environmental Protection Management of SARS Prevention and Control Construction Projects | 14-May-03 | Ministry of Ecology and Environment |
| 122 | Interim Measures for the management of the preservation, use and infection of animal models of infectious Atypical pneumonia virus | 6-May-03 | Ministry of Science and Technology, National Health Commission, National Medical Products Administration, Ministry of Ecology and Environment |
| 123 | Interim Administrative Measures for the Research Laboratory of SARS virus | 6-May-03 | Ministry of Science and Technology, National Health Commission, Ministry of Ecology and Environment, National Medical Products Administration |
| 124 | Law of the People's Republic of China on Prevention and Treatment of Infectious Diseases | 21-Feb-89 | Member of the Standing Committee of the National People's Congress |
| 125 | Law of the People's Republic of China on the Prevention and Control of Environment Pollution Caused by Solid Wastes | 30-Oct-95 | Member of the Standing Committee of the National People's Congress |
| 126 | Regulations for the Administration of Affairs Concerning Experimental Animals | 31-Oct-88 | The State Council |
| 127 | Regulations on the Administration of Medical Wastes | 16-Jun-03 | The State Council |
| 128 | Notice on the Guidance on Effectively Implementing the International Health Regulations 2005 to Accelerate the Building of Core Capacity for Public Health Emergency Response | 24-Jul-13 | Office of the State Council |
| 129 | Notice on Further improving the biosafety management of COVID-19 laboratories | 17-Jan-23 | National Health Commission |
| 130 | Detailed Rules for the Designation of Institutions for the Preservation of Pathogenic Microorganisms (Toxins) of Human Infectious Diseases | 12-May-11 | National Health Commission |
| 131 | Measures for the Management of Medical Waste in Medical and Health Institutions | 15-Oct-03 | National Health Commission |
| 132 | Notice on the issuance of Laboratory Biosafety Accreditation Rules (CNAS-RL05.2016) and other accreditation specification documents | 1-Feb-16 | China National Accreditation Service for Conformity Assessment |
| 133 | Public Security Industry Standard of the People's Republic of China, Part I: High-level Pathogenic Microorganism Laboratories | 28-Dec-22 | Ministry of Public Security |
| 134 | Requirements for Biosafety in Mobile Laboratories | 11-Sep-15 | State General Administration of the People’s Republic of China for Quality Supervision and Inspection and Quarantine, Standardization Administration of the People's Republic of China |
| 135 | General Requirements for Laboratory Biosafety | 5-Apr-04 | State General Administration of the People’s Republic of China for Quality Supervision and Inspection and Quarantine, Standardization Administration of the People's Republic of China |
| 136 | Announcement on the release of the national standard "Biosafety Laboratory building Technical Code" | 3-Aug-04 | Ministry of Housing and Urban-Rural Development, State General Administration of the People’s Republic of China for Quality Supervision and Inspection and Quarantine |
| 137 | Announcement on the Issuance of Five Health Industry Standards, Including the "General Guidelines for Biosafety in Pathogenic Microorganism Laboratories" | 3-Nov-02 | National Health Commission |
