# Supplementary material for: Mining and quantitative evaluation of the laboratory biosafety policy in China
Source: PLoS One. 2025 Aug 12;20(8):e0328923. doi: 10.1371/journal.pone.0328923 (PMC12342282; doi:10.1371/journal.pone.0328923)
Supplement: Attachment — Submitted filename: Response to Reviewers.docx [file pone.0328923.s004.docx]

**Response**

Dear Editor and Reviewers,

Thank you very much for your careful review of our manuscript entitled " Mining and quantitative evaluation of the laboratory biosafety policy in China". We are sincerely grateful for your thoughtful suggestions and critical comments, which have greatly contributed to the improvement of our work. The feedback has provided valuable direction not only for the revision of this manuscript, but also for our broader research efforts.

We have revised the manuscript accordingly, with all changes highlighted in red in the updated version. A detailed, point-by-point response to each comment is presented below.

Reviewer #1: Biosafety management system is not only worth studying in theory, but also in practice. Therefore, the topic of this paper deserves full affirmation. This paper is generally good, from the research method, data application and research content, are commendable. In terms of language and text, it is also necessary to optimize, and it is best to find professional institutions or people for native language modification. In the summary, we should briefly explain the countermeasures and suggestions. The countermeasures and suggestions in the last part of the article can be supplemented with corresponding discussions, like 4 and 5 in a simple sentence, which is a little irresponsible.

**Answer:**

Thank you very much for your suggestion. As for language, we tried our best to improve the manuscript and made some changes to the manuscript. These changes will not influence the content and framework of the paper. And here we did not list the changes but marked in red and blue in the revised paper.

Regarding the suggestions, we have fully considered the reviewers' comments and refined the originally proposed suggestions. In terms of strengthening communication among departments, the original suggestion was to accelerate the formation of a biosafety coordination mechanism, improve the information-sharing mechanism among departments, and establish an efficient working mechanism. Considering the feasibility of the suggestions, we have supplemented the content of regularly convening cross-departmental joint meetings in the revised version to enhance the feasibility of the suggestions.

In terms of the policy formulation process, we have mainly made revisions to the language expression without changing the content.

Regarding t Enhance supervision of biosafety level 1 and 2 (BSL-1/2) laboratories., considering that the filing system is an important policy for the management of Level 1 and 2 laboratories and that the current main issue lies in the differences in procedures and effectiveness across provinces and cities, we have adjusted the sequence of the two originally proposed suggestions.

In terms of leveraging the power of industry associations, the original version only briefly mentioned the rational use of non-governmental forces without clarifying how to bring their roles into play. The revised version has supplemented this content.

Regarding the original suggestion to set phased goals for long-term policies, considering that this involves issues related to policy-making procedures, we have deleted this item. The final version was as follows.

*1. Strengthen communication among different departments. A clear coordination mechanism and a standardized information-sharing platform should be established for laboratory biosafety. Regular cross-institutional joint meetings can facilitate communication and interaction among departments, ensuring timely transmission and exchange of relevant information. This approach enables effective management and prevents the issuance of duplicate policies across different departments.*

*2. Optimize* *the policy formulation process. Before drafting new policies, a comprehensive review of existing policies should be conducted, accompanied by expert discussions, and evidence-based investigations to ensure systemic coherence, logical consistency, and non-redundancy from a holistic perspective. In addition, when issuing policies, efforts should be made to minimize reliance on notifications as the primary format. Converting select notifications into formal documents would enhance their systematicity, stability, sustainability, and enforceability.*

*3.* *Enhance supervision of biosafety level 1 and 2 (BSL-1/2) laboratories. A laboratory registration system has been established at the national level for biosafety level 2 laboratories; however, during implantation, registration format, procedures and validity period varies across provinces and cities, and the review process of the management part is also inconsistent[33]. Therefore, there is an urgent need to convene expert panels to develop standardized and implementable registration and management criteria to ensure consistency in the management of low-level biosafety laboratories. Additionally, it is recommended to enhance regulatory measures by formulating corresponding policies—for example, encouraging the adoption of digital technologies in laboratory biosafety management and establishing a unified digital information management system to enable laboratory monitoring, early warning, and post-incident response.*

*4. Harnessing the power of industry associations. Under the national philosophy of multi-stakeholder collaboration, it is crucial to strategically engage industry forces, particularly by actively guiding industry associations to deeply participate in the development of biosafety quality control standards. This involvement serves to standardize the implementation of laboratory biosafety management protocols. Additionally, regular expert-led inspections of laboratory biosafety can be conducted to monitor and facilitate policy adherence.*

Journal Requirements 1. Please ensure that your manuscript meets PLOS ONE's style requirements, including those for file naming. The PLOS ONE style templates can be found at

https://journals.plos.org/plosone/s/file?id=wjVg/PLOSOne_formatting_sample_main_body.pdf and

https://journals.plos.org/plosone/s/file?id=ba62/PLOSOne_formatting_sample_title_authors_affiliations.pdf.

**Answer:**

Thank you very much for your suggestion. I have already done that.

Journal Requirements 2. PLOS requires an ORCID iD for the corresponding author in Editorial Manager on papers submitted after December 6th, 2016. Please ensure that you have an ORCID iD and that it is validated in Editorial Manager. To do this, go to ‘Update my Information’ (in the upper left-hand corner of the main menu), and click on the Fetch/Validate link next to the ORCID field. This will take you to the ORCID site and allow you to create a new iD or authenticate a pre-existing iD in Editorial Manager.

**Answer:**

Thank you very much for your suggestion. I have already done that.

Journal Requirements 3. Please remove your figures from within your manuscript file, leaving only the individual TIFF/EPS image files, uploaded separately. These will be automatically included in the reviewers’ PDF.

**Answer:**

Thank you very much for your suggestion. I have already done that.

Journal Requirements 4. Please review your reference list to ensure that it is complete and correct. If you have cited papers that have been retracted, please include the rationale for doing so in the manuscript text, or remove these references and replace them with relevant current references. Any changes to the reference list should be mentioned in the rebuttal letter that accompanies your revised manuscript. If you need to cite a retracted article, indicate the article’s retracted status in the References list and also include a citation and full reference for the retraction notice.

**Answer:**

Thank you very much for your suggestion. I have already done that.

We appreciate for Editors/Reviewers’ warm work and hope that the correction will meet with approval.
